# Obligate autotrophy at the thermodynamic limit of life in a new acetogenic bacterium

**DOI:** 10.3389/fmicb.2023.1185739

**Published:** 2023-05-12

**Authors:** Evgenii N. Frolov, Alexander G. Elcheninov, Alexandra V. Gololobova, Stepan V. Toshchakov, Andrei A. Novikov, Alexander V. Lebedinsky, Ilya V. Kublanov

**Affiliations:** ^1^Federal Research Center of Biotechnology, Winogradsky Institute of Microbiology, Russian Academy of Sciences, Moscow, Russia; ^2^Kurchatov Center for Genome Research, National Research Center “Kurchatov Institute”, Moscow, Russia; ^3^Department of Physical and Colloid Chemistry, Gubkin University, Moscow, Russia

**Keywords:** acetogenesis, obligate autotrophy, acetogenic bacterium, the Wood-Ljungdahl pathway, Ech complex, electron-bifurcating hydrogenase, *Aceticella*

## Abstract

One of the important current issues of bioenergetics is the establishment of the thermodynamic limits of life. There is still no final understanding of what is the minimum value of the energy yield of a reaction that is sufficient to be used by an organism (the so-called “biological quantum of energy”). A reasonable model for determination of the minimal energy yield would be microorganisms capable of living on low-energy substrates, such as acetogenic prokaryotes. The most prominent metabolic feature of acetogens is autotrophic growth with molecular hydrogen and carbon dioxide as the substrates, which is hardly competitive in environments. Most probably, that is why only facultative autotrophic acetogens have been known so far. Here, we describe the first obligately autotrophic acetogenic bacterium *Aceticella autotrophica* gen. nov., sp. nov., strain 3443-3Ac^T^. Phylogenetically, the new genus falls into a monophyletic group of heterotrophic bacteria of the genera *Thermoanaerobacterium*, *Thermoanaerobacter*, and *Caldanaerobacter* (hereinafter referred to as TTC group), where the sole acetogenic representative has so far been the facultatively autotrophic *Thermoanaerobacter kivui*. *A. autotrophica* and *T. kivui* both are acetogens employing energy-converting hydrogenase (Ech-acetogens) that are likely to have inherited the acetogenesis capacity vertically from common ancestor. However, their acetogenic machineries have undergone different adjustments by gene replacements due to horizontal gene transfers from different donors. Obligate autotrophy of *A. autotrophica* is associated with the lack of many sugar transport systems and carbohydrate catabolism enzymes that are present in other TTC group representatives, including *T. kivui*.

## 1. Introduction

Acetogenic bacteria are a specialized group of strictly anaerobic microorganisms that are able to produce acetate from two molecules of carbon dioxide (CO_2_) via the Wood–Ljungdahl pathway (WLP) during anaerobic respiration. Their lithotrophic growth occurs according to the equation:

4H_2_+2CO_2_ = CH_3_COOH+2H_2_O (ΔG_0_′ = −104 kJ).

Under environmental conditions, the free energy change of the reaction allows for the synthesis of only a fraction of an ATP mole, and acetogens are thus a paradigm for microbial life under extreme energy limitation (Schuchmann and Müller, 2014).

Currently, over 100 acetogens, representing 28 bacterial genera, have been isolated from diverse habitats (Drake et al., 2008; Allen et al., 2010; Zhilina et al., 2012; Poehlein et al., 2013; Gerritsen et al., 2014; Lawson et al., 2016; Mao et al., 2021). While most acetogenic isolates are in the phylum Firmicutes, they also include Spirochaetes like *Treponema azotonutricum* and *Treponema primitia*, Desulfobacterota like *Desulfotignum phosphitoxidans*, and Acidobacteria like *Holophaga foetida* (hereafter, unless otherwise indicated, taxonomy is according to Bergey’s Manual of Systematics of Archaea and Bacteria).^1^ Among Archaea, only for *Methanosarcina acetivorans* and *Archaeoglobus fulgidus* and only with the low-potential CO as the electron donor was the energy conservation via acetogenesis reported (Rother and Metcalf, 2004; Henstra et al., 2007; Schöne et al., 2022). In addition, acetogenesis has been suggested for some uncultivated Bacteria like ‘*Candidatus* Adiutrix intracellularis’ (Ikeda-Ohtsubo et al., 2016) and Archaea, e.g., members of the phyla Bathyarchaeota and Lokiarchaeota and anaerobic methanotrophic archaea ANME-2a (Evans et al., 2015; He et al., 2016; Orsi et al., 2020; Yang et al., 2020; Loh et al., 2021; Deb and Das, 2022).

The most prominent metabolic feature of acetogens is chemolithoautotrophic growth with H_2_ as the energy source and HCO_3_^–^/CO_2_ as the carbon source and electron acceptor. However, acetogens have to grow under highly competitive conditions, since, in anaerobic environments methanogens and sulfate-reducing prokaryotes are usually the dominant hydrogenotrophs, outcompeting acetogens due to the greater energy yield (Ragsdale and Pierce, 2008) and a lower H_2_ threshold (Le Van et al., 1998). Most probably, that is why only facultative autotrophic acetogens have been known so far. Heterotrophic capabilities of acetogens are quite flexible, they are known to utilize a vast variety of organic substrates like sugars, alcohols, organic acids, aldehydes, and aromatic compounds. Acetogens can also use a variety of alternative electron acceptors, like nitrate, nitrite, thiosulfate, sulfate, Fe(III), perchlorate, dimethylsulfoxide, fumarate, and others (Ragsdale and Pierce, 2008). The ability of acetogenic bacteria to form acetate from many organic substrates trophically links them to methanogens that can use acetate as a substrate. This makes acetogens an essential part of anaerobic food webs (Schuchmann and Müller, 2014). Metabolic flexibility is believed to be a key capability of acetogens, explaining the almost ubiquitous distribution of acetogenic bacteria in anoxic environments: soils, freshwater and marine sediments, subsurface samples, alkaline and high-salt environments, hot springs, sludge, and the intestinal tracts of many animals, including humans and termites (Ragsdale and Pierce, 2008; Schuchmann and Müller, 2014). Thus, acetogens play an important part in the global carbon cycle, although they never represent a numerically significant part of microbial communities.

The catabolism of acetogens is usually regarded as modular. The first module consists of electron-donating reactions (the oxidative part of acetogenesis), the second module includes the reductive reactions of acetogenesis (the Wood-Ljungdahl pathway), while the third module connects redox cofactor recycling to energy conservation and comprises a membrane-bound respiration system involving Rnf (ferredoxin: NAD+ oxidoreductase termed after *Rhodobacter* nitrogen fixation complex) or Ech (energy-converting hydrogenase) complex and ATP synthase (Schuchmann and Müller, 2014, 2016; Basen and Müller, 2017). In the first module, organic or inorganic substrates are oxidized by donating electrons to ferredoxin (Fd) and NAD^+^. It is important to note that the largest thermodynamic barrier in acetogenesis is the reduction of CO_2_ to CO in the carbonyl branch of WLP. This redox couple has a very low standard redox potential (E_0_′ = –520 mV, Thauer et al., 1977). Therefore acetogens must employ low potential ferredoxin, and there are ferredoxins that are known to have redox potentials of –500 mV and even lower (Bar-Even, 2013). During chemoorganoheterotrophic growth (Kuhns et al., 2020) a few enzymes such as tungsten-containing aldehyde:ferredoxin oxidoreductase (Heider et al., 1995) or pyruvate:ferredoxin oxidoreductase (Blamey and Adams, 1993) can replenish the low potential reduced ferredoxin, oxidized in the WLP carbonyl branch. However, during chemolithoautotrophic growth with hydrogen, low potential ferredoxin has to be reduced with hydrogen as the electron donor, but this reaction is endergonic (Schuchmann and Müller, 2014) since the standard redox potential of the couple H^+^/H_2_ is –414 mV (Thauer et al., 1977). This energy barrier is overcome by means of flavin-based electron bifurcation, where the endergonic reduction of low potential ferredoxin is coupled to the exergonic reduction of NAD^+^ (Buckel and Thauer, 2018; Müller et al., 2018). In acetogens, this reaction is catalyzed by the soluble electron-bifurcating hydrogenase (Schuchmann and Müller, 2012, 2014). Ferredoxin and NAD^+^ are reduced simultaneously in a 1/1 stoichiometry in an overall exergonic reaction (ΔG_0_′ = –11 kJ per mole, Herrmann et al., 2008; Schuchmann and Müller, 2012; Buckel and Thauer, 2013; Wang et al., 2013). Therefore, the oxidative module of acetogenesis yields NADH and reduced ferredoxin. Part of this ferredoxin is consumed in the second, reductive acetogenesis module (specifically, in the WLP carbonyl branch), and the remaining ferredoxin is used to generate a chemiosmotic gradient in the third module of acetogenesis (Schuchmann and Müller, 2014).

It is a unique property of acetogenic microorganisms to use the WLP not only as the carbon fixation pathway in anabolism but also as the terminal electron sink. How the WLP can be coupled to the synthesis of ATP has been thoroughly studied in *Acetobacterium woodii*, which has a simple respiratory chain consisting of a ferredoxin: NAD^+^ oxidoreductase (Rnf complex) and a Na^+^-dependent F_1_F_0_-ATP synthase (Fritz and Müller, 2007; Biegel and Müller, 2010; Hess et al., 2013). The Rnf complex generates a transmembrane electrochemical Na^+^ gradient, which fuels a Na^+^-dependent ATP synthase. Another model organism for the study of acetogenesis, *Clostridium ljungdahlii*, also contains an Rnf complex, but the chemiosmotic ATP synthesis in this organism seems to be driven by protons (Köpke et al., 2010; Tremblay et al., 2012). However, the Rnf complex is not present in every acetogen, and in the case of its absence its role is accomplished by the energy-converting hydrogenases (Ech complexes), detected in the Rnf-free acetogens like *Thermoanaerobacter kivui, Thermacetogenium phaeum* and acetogenic representatives of the genus *Moorella* (Hess et al., 2014; Schuchmann and Müller, 2014; Basen and Müller, 2017; Schoelmerich and Müller, 2019). Energy conservation by a hydrogenase-dependent chemiosmotic mechanism has been studied in detail for the thermophilic acetogenic bacterium *Thermoanaerobacter kivui* (Hess et al., 2014; Schoelmerich and Müller, 2019). The operation of the Ech complex of *T. kivui* leads to the translocation of H^+^ and Na^+^ across the membrane, and the H^+^ gradient is harnessed for energy conservation by the H^+^-dependent F_1_F_0_-ATP synthase. Thus, acetogenic bacteria have been classified into “Rnf-acetogens” and “Ech-acetogens” (Schuchmann and Müller, 2014).

In this work we isolated the first obligately autotrophic acetogenic bacterium *Aceticella autotrophica* gen. nov., sp. nov., strain 3443-3Ac^T^, and described its metabolic properties, focusing on its energy metabolism and the likely causes for inability to grow heterotrophically.

## 2. Materials and methods

### 2.1. Enrichment, isolation, and physiological studies

Strain 3443-3Ac^T^ was isolated from a sample of sediments collected from the terrestrial hot spring Kaskadny at East Thermal Field, Uzon Caldera, Kamchatka, Russia (N54° 30.026′ E160° 00.374′, elevation 658 m) in August 2015. The thermal fluid parameters were 58°C, pH 3.1 and Eh +90 mV. The indigenous microbial community of the hot spring Kaskadny was described previously (Merkel et al., 2017). Samples were taken anaerobically, placed into tightly stoppered bottles and transported to the laboratory. An enrichment culture was initiated by inoculating the sample [10% (w/v)] into anaerobically prepared sterilized (by autoclaving at 121°C for 1 h) liquid medium of the following composition (g L^–1^): NH_4_Cl, 0.33; KCl, 0.33; MgCl_2_ ⋅2H_2_O, 0.33; CaCl_2_ ⋅6H_2_O, 0.33; KH_2_PO_4_, 0.33; trace element solution (Kevbrin and Zavarzin, 1992), 1 ml L^–1^; vitamin solution (Wolin et al., 1963), 1 ml L^–1^. Sodium sulfide (0.5 g L^–1^) was used as a reducing agent. Resazurin was added as a redox indicator in a concentration of 1.0 mg L^–1^. To adjust the pH of the medium to 6.0, 2N NaOH was used. The medium was dispensed in 5-ml aliquots into 17-ml Hungate tubes; the head space was filled with H_2_/CO_2_ (4:1, 152 kPa). After 4 days of incubation at 50°C, a pronounced production of acetate accompanied by microbial growth was observed. The culture was purified using the dilution-to-extinction technique, and the resulting isolate was designated as strain 3443-3Ac^T^.

Cell growth and cell morphology were monitored using an Olympus CX-41 phase-contrast microscope. Negatively stained cells and fine structure of the cells were studied using a JEM-100 electron microscope as described previously (Bonch-Osmolovskaya et al., 1990).

Growth experiments were performed in triplicates using Hungate tubes with the medium of the same composition as used for the pure culture isolation. To determine optimal growth conditions, strain 3443-3Ac^T^ was cultivated anaerobically at various temperatures (22–65°C) and pH values 4.3–7.5. The pH in the medium was adjusted with solutions of HCl (3N) or NaOH (2N). To test the reaction of the new isolate to NaCl concentration, it was anaerobically cultivated at NaCl concentrations from 0 to 30 g L^–1^ with intervals of 5 g L^–1^. To determine strain 3443-3Ac^T^ Na^+^-dependence sodium-deficient medium was prepared by replacing sodium sulfide with 3 mM cysteine-HCl.

The utilization of substrates other than H_2_ was tested with 100% CO_2_-filled headspace at 50°C and pH 6.0. Growth experiments for determination of CO consumption were performed exactly as described for *Thermoanaerobacter kivui* by Weghoff and Müller (2016). The utilization of CO was tested with 10, 20, 30, 40, 50, 60, 70, and 100% CO in CO_2_-filled headspace. Electron acceptors were tested in the medium under H_2_/CO_2_ atmosphere or in the medium containing glucose, acetate, pyruvate, ethanol, methanol, or formate under CO_2_-filled headspace at 50°C and pH 6.0. Soluble substrates and electron acceptors were added from sterile anaerobic stock solutions before inoculation. Insoluble substrates and electron acceptors were added directly into the tubes with liquid medium prior to sterilization. Utilization of substrates or electron acceptors was monitored by increase in optical density due to microbial growth and by decrease of electron donor or acceptor concentration in the medium during growth.

Strain 3443-3Ac^T^ was deposited in the DSMZ (German Collection of Microorganisms and Cell Cultures) and VKM (All-Russian Collection of Microorganisms) under accession numbers DSM 108286 and VKM B-3415, respectively.

### 2.2. Analytical methods

Sulfide was measured colorimetrically with N,N-dimethyl-*p*-phenylenediamine (Trüper and Schlegel, 1964). Sulfate, nitrate, nitrite, perchlorate, and chloride were analyzed with a Stayer liquid chromatograph (Aquilon) equipped with an IonPack AS4-ASC column (Dionex) and conductivity detector; the eluent was bicarbonate (1.36 mM)/carbonate (1.44 mM) at a flow rate of 1.5 ml min^–1^. H_2_ and CO_2_ were analyzed with Chromatec Crystal 5000.2 gas chromatograph (Chromatec) equipped with a NaX zeolite 60/80 mesh 3 m × 2 mm column (Chromatec) for H_2_ and an Hayesep Q 80/100 mesh 3 m × 2 mm column (Chromatec) for CO_2_. Acetate, formate, propionate, butyrate, methanol, ethanol, propanol, isopropanol, butanol, isobutanol were assayed using two methods. The first method was the same gas chromatograph with the flame ionization detector (FID) and capillary column Optima FFAPplus 0.25 μm × 0.32 mm × 30 m (Macherey-Nagel) with argon as the carrier gas. Separation was carried out with temperature programming. For gas chromatography, the samples (0.2 ml) were pre-treated by centrifugation at 12600 × *g* for 2 min, followed by acidification of clear supernatants with 5 M formic acid to the pH of 2.0. The detection limit of the method was 0.2 mM. The second method for the determination of volatile fatty acids and alcohols was HPLC on a Stayer gradient chromatograph (JSC Aquilon, Russia) equipped with a 7.8 mm × 300 mm Resex ROA column. The eluent was 0.5 mN HNO_3_; the flow rate was 1.5 mL/min. A conductometer was used as the detector.

Cellular fatty acid (CFA) profiles were determined by GC-MS (Thermo Scientific Trace GC Ultra DSQ II, HP-5MS column, E_*I*_ 70 eV) of methyl ester derivatives prepared from 10 mg of freeze-dried biomass treated by anhydrous HCl/MeOH (Slobodkina et al., 2020). Polar lipids were determined by 2D-TLC as described earlier (Slobodkina et al., 2013). Respiratory lipoquinones were extracted with cold acetone from cells disrupted by grinding in liquid N_2_ and further separated by thin-layer chromatography (TLC). The excised bands were analyzed by tandem mass spectrometry (LCQ ADVANTAGE MAX) and the compounds were identified by their ionized masses.

### 2.3. Activity assays in cell extract

Cells of strain 3443-3Ac^T^ were suspended in 50 mM Tris⋅HCl buffer (pH 7.5) containing 120 mM KCl. The cells lysate was obtained using the ultrasonic disintegrator Soniprep 150 Plus (150 W, 23 kHz). The lysate was centrifuged for 30 min (12,100 g; 4°C), and the supernatant (cell extract) was stored in anoxic conditions (flushed with sterile 100% CO_2_) at +4°C until use (not more than 3 days). The protein concentration, measured using Qubit Protein Assay Kit (Thermo Fisher Scientific, USA), was 1.87 mg ml^–1^.

Glucose and cellobiose consumption by strain 3443-3Ac^T^ cell extract was determined using a DNS assay (Miller, 1959). D-glucose and D-cellobiose solutions at various (50–500 μg/ml) concentrations were used to plot a calibration curve for DNS assay. The reaction mixtures (700 μl) contained 70 μl of 10 mM glucose or cellobiose in a TrisHCl buffer (50 mM, pH 7.5) and 630 μl of cell extract (the final concentration of sugars in solution was 1 mM). 1 mM glucose or cellobiose solution in TrisHCl buffer as well as cell extract without added sugars were used as controls. All controls and experiments were set in three replications. The reaction mixtures were incubated at the strain 3443-3Ac^T^ optimal growth temperature (50°C) for 9 h followed by determination of reducing sugars formation by DNS assay (Miller, 1959).

Hexokinase activity was measured spectrophotometrically using a coupled assay with glucose 6-phosphate dehydrogenase. The reaction rate was followed by the production of NADPH at 340 nm. The assay mixture (2.7 ml) contained 50 mM TrisHCl, pH 8.5; 25 mM NaCl; 2 mM MgCl_2_; 2 mM ATP; 3 mM glucose; 0.8 mM NADP^+^; and 1 U glucose-6-phosphate dehydrogenase from baker’s yeast (*S. cerevisiae*) type VII, 3.2 M ammonium sulfate suspension (Cáceres et al., 2003). The reaction was started by the addition of strain 3443-3Ac^T^ cell extract (300 μl). The same reaction mixtures (3 ml) without the cell extract or substrate were used as the control assays.

### 2.4. Genome sequencing and assembly

The extraction of genomic DNA was performed as described by Park (2007). The genomic DNA was sonicated with a CovarisS2 device with parameters adjusted to obtain 400 bp fragments. Then DNA fragments were used for the preparation of fragment libraries using the NEBNext^®^ Ultra™ II DNA Library Prep Kit for Illumina^®^ (E7645, NEB) according to the manufacturer’s instructions. The library was sequenced on the Illumina HiSeq2500. As a result, 6,593,610 single end reads were obtained (in total 1,648.4 Mb). For nanopore sequencing, the DNA was re-purified using MagAttract HMW DNA Kit (Qiagen, Germany) to enrich high molecular weight DNA fraction and remove co-purified contaminants. Genomic library for nanopore sequencing was prepared with Rapid Barcoding Kit (SQK-RBK004, Oxford Nanopore Technologies, UK). Sequencing was performed with FLO-MIN-106D flow cell (R9.4.1). Basecalling was performed using Guppy basecaller v2.3.5 with flipflop model and resulted in 128,379 reads with a median read length of 611 nucleotides (total of about 167.4 Mb).

Primary *de novo* assembly was obtained by Canu assembler v. 1.9, using only long nanopore reads (Koren et al., 2017). Polishing of the primary assembly was performed both with raw Nanopore reads by Nanopolish (Loman et al., 2015) and correction of the polished assembly with Illumina reads by Pilon (Walker et al., 2014). As a result an assembly consisting of 1 linear contig of approximately 2.24 Mbp total length was obtained. For the assembly improvement, hybrid *de novo* assembly was made by SPAdes assembler version 3.13.0 (Prjibelski et al., 2020) using both long and short reads, resulting in 2.3 Mbp total assembly with N50 of 1,678,036 bp. Manual curation and visualization of two assemblies, performed with CLC genomic workbench 3.10.0 (Qiagen, Germany), allowed one circular chromosome of 2,267,618 bp length to be obtained. Start of the chromosome was set to origin of replication, predicted by DoriC 10.0 web server (Luo and Gao, 2019). Genome sequence, as well as related project information and sample details, were deposited in NCBI database under accession numbers CP060096, PRJNA647162, and SAMN15577649, respectively. The genome sequence was also deposited in IMG with genome ID 2860381621.

### 2.5. Genome annotation

Primary genome annotation was performed by Prokaryotic Genome Annotation Pipeline (PGAP, Tatusova et al., 2016) during the process of genome submission to NCBI submission portal. Additional annotations aimed to improve predictions of protein function were performed using IMG/MER System (Chen et al., 2017) and Rapid Annotation using Subsystem Technology web server (Brettin et al., 2015) and manual curation of key metabolic genes. Refining of the automated annotations and other predictions were done manually as described by Toshchakov et al. (2015). Prediction of mobile elements was performed with *isescan* package (Xie and Tang, 2017), identification and prediction of phage-related genes was done with PHASTer server (Arndt et al., 2016). Prediction of genomic islands (GI) was performed by IslandViewer 4 web tool (Bertelli et al., 2017). Additional annotation of defense systems and biosynthetic gene clusters was performed by defense-finder tool (Tesson et al., 2022) and antiSMASH (Blin et al., 2019) web server, respectively.

Gene gain and loss that occurred during the evolution of *Thermoanaerobacterium*, *Thermoanaerobacter*, *Caldanaerobacter* phylogenetic cluster was analyzed using Count (Csurös, 2010). Short version of phylogenetic tree based on bac120 (see below) was used as a reference tree. All the proteins involved in the gene gain/loss analysis were listed as their COG identifiers, assigned by the IMG during annotation. Rates in Count were optimized with default parameters.

### 2.6. Phylogenetic and phylogenomic analyses

Phylogenetic analysis based on the bac120 set of conserved proteins (Parks et al., 2017) was performed as follows. All available genomes of representatives of Bergey’s-based order *Thermoanaerobacterales* and a large set of reference genomes affiliated to GTDB-based p_Firmicutes_A phylum (containing closest relatives of strain 3443-3Ac^T^: *Thermoanaerobacterium*, *Thermoanaerobacter*, *Caldanaerobacter* spp.) and to GTDB-based p_Firmicutes phylum (as an outgroup), as well as the genome of *Melioribacter roseus* P3M-2^T^ (as a root) were downloaded from Genbank. The protein sequences were identified and aligned with the GTDB-tk v.1.3.0 (Parks et al., 2018). The resulting alignment was treated using the Gblock v.0.91b with the gentlest parameters and half gap elimination (Castresana, 2000). The phylogenetic analysis was performed in the RAxML v. 8.2.12 (Stamatakis, 2014) with the PROTGAMMAILG model of amino acid substitution. Local support values were 1,000 rapid bootstrap replications.

For studies of the evolutionary origin of acetogenic metabolism in our isolate, we constructed phylogenetic trees of proteins involved in acetogenesis. As a source of reference proteins, we chose representative genomes of GTDB rs202 (47,894 genomes). GTDB places emphasis on inclusion of as much high-quality sequence data as possible and on systematization, i.e., provision of full taxonomic assignments from species to domains using uniform approaches of genome-based phylogenetic classification (Parks et al., 2018, 2020). The GTDB taxonomy is in reasonable accord with traditional taxonomy accepted by microbiologists, and the two taxonomies have been gradually getting closer. GTDB representative genomes represent all groups of prokaryotes, both cultured and yet uncultured, that the GTDB team considers to be species based on the phylogenetic criteria applied (one genome for each GTDB species). Thus, GTDB representative genomes present a compact and reasonably adequate sampling of prokaryotic diversity.

Representative genomes of GTDB rs202 (47,894 genomes) were downloaded from GTDB repository^2^ to create a local database for tblastn from NCBI-BLAST+ package (Camacho et al., 2009), operated at default values of most parameters (the default for *e*-value is 10). For the -num_alignments and -num_descriptions options, the value of 50,000 was set.

The queries for tblastn were the proteins of our strain 3443-3Ac^T^ and/or the proteins of *T.kivui*. The RnfD and RnfC queries were Awo_c22050 and Awo_c22060 from *Acetobacterium woodii*. For convenience of further analysis of gene co-occurrence and co-location, the queries were usually concatenated (up to 17 subqueries separated by strings of 100 “x” letters). The cut-off values for identification of tblastn hits were usually 25% identity with a particular subquery at 80% coverage. In case of the catalytic subunit of energy-converting hydrogenase (EchE), the identity cut-off value was 28% and there was an additional requirement for the presence of two CxxC nickel binding motifs to distinguish the ECH catalytic subunits from homologous subunits of membrane-bound oxidoreductases (Vignais and Billoud, 2007). Hit sequences extracted from tblastn output with home-made software (see below) were checked for validity of gene identification by submitting them to HydDB (Søndergaard et al., 2016) for EchE (9 of 4,275 sequences had to be discarded) or HMMSCAN (Potter et al., 2018). An additional confirmation of the validity of gene identification was the expected clustering of certain hits in the genome (although particular organization of the gene clusters could vary).

The analysis of tblastn output (namely, the complete set of alignments provided by tblastn was used) was performed by a home-made software, whose earlier version was successfully used by us to analyze genomic contexts of CO dehydrogenase genes in microbial genomes (Techtmann et al., 2012). The software provided a report on gene co-location (within a user specified distance, 15 kb by default), and also on a tighter co-location of genes (0.5 kb). The software also extracted hit sequences directly from the tblastn output and printed them to files. The names of the extracted sequences were formulated so as to include information on the host genome GTDB taxonomic string and the genomic context revealed. These sequences were used to construct Neighbor Joining (NJ) and Maximum Likelihood (ML) trees. The protein sequences extracted from tblastn alignments somewhat varied in their completeness (default query coverage requirement was 80%); however, the variable terminal regions that escaped analysis would have been anyway neglected in the process of tree construction, where the parameters that we used included deletion of alignment positions with coverage below 95%).

The phylogenetic trees of proteins involved in acetogenesis were constructed as follows. For “total” trees, all sequences of the target proteins extracted from the output of tblastn against the database of GTDB rs202 representative genomes were aligned with MAFFT version 7 (Katoh et al., 2019), and NJ and/or ML trees were constructed with MEGA6 (Tamura et al., 2013). For smaller trees, 25 best hits of *A.autotrophica*’s proteins and 25 best hits of *T.kivui*’s proteins were taken after tblastn in representative genomes of GTDB rs202. Homologous proteins of TTC group representatives from the same database were added in cases where the same enzymatic activity could be assumed. Redundant proteins were discarded with CD-HIT (Fu et al., 2012) at 100% cut-off value. The ML trees with 100 bootstrap replicates were constructed with MEGA6 after alignment with built-in ClustalW.

Since GTDB taxonomy is firmly phylogeny-grounded, the inclusion of the GTDB taxonomic string in sequence names in trees allows one to do without juxtaposition of a phylogenetic tree constructed for a particular protein with a tree showing the phylogeny of the core genes of the host organism (since the information on the host phylogeny is provided by the sequence name). Conclusions on vertical inheritance or HGT events can be drawn without such juxtaposition.

## 3. Results and discussion

### 3.1. Physiological characterization of strain 3443-3Ac^T^

Strain 3443-3Ac^T^ was isolated from a sample of sediments collected from the terrestrial hot spring Kaskadny at East Thermal Field, Uzon Caldera, Kamchatka, Russia (N54° 30.026′ E160° 00.374′, elevation 658 m). The isolation was performed in anaerobic medium in the presence of H_2_ as the energy source and HCO_3_^–^/CO_2_ as the carbon source and electron acceptor.

Cells of strain 3443-3Ac^T^ were rod-shaped, 0.5–0.7 μm in diameter and 1.5–5 μm in length; occurred singly, in pairs, or in chains depending on the growth phase and growth conditions. No motility was observed. The cells examined in negatively stained specimens (Supplementary Figure 1A) did not exhibit flagella. Strain 3443-3Ac^T^ formed round terminal endospores after prolonged (10 days) incubation at 50°C. The percentage of cells with spores, though, was no more than 1–2%. Gram-stain reaction was positive and ultrathin sections of the cells (Supplementary Figure 1B) revealed a Gram-positive cell wall type.

**FIGURE 1:**
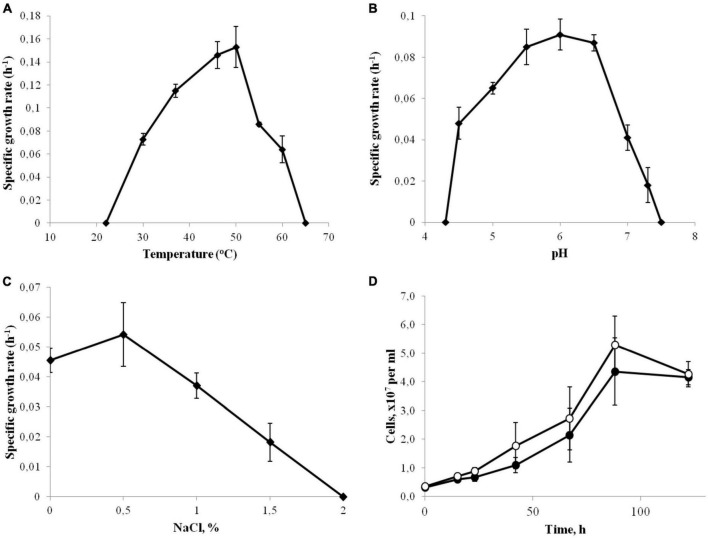
Effect of temperature **(A)**, pH **(B)** and NaCl concentrations **(C)** on the growth rate of strain 3443-3Ac^T^. **(D)** Effect of Na^+^ on the growth of strain 3443-3Ac^T^; cultures grown on H_2_ and CO_2_ were passaged in Na^+^-enriched (●) and Na^+^-deficient (°) minimal medium, fifth passage is shown. Error bars indicate standard deviations of biological replicates (*n* = 3).

Strain 3443-3Ac^T^ was a strictly anaerobic bacterium growing at low-potential redox conditions: no growth occurred in a sodium sulfide-free medium. The temperature range for growth was 30–60°C, with an optimum at 46–50°C (Figure 1A). No growth was detected at 65°C or above, as well as at 22°C or below after incubation for 10 days. The pH range of growth was 4.5–7.3, with an optimum at pH 6.0 (Figure 1B). Growth was not observed at and below pH 4.3 and at and above pH 7.5. The isolate grew at NaCl concentrations of up to 1.5% NaCl (Figure 1C), but optimal growth was observed on the basal medium, where the concentrations of Na^+^ and Cl^–^ were 0.1 and 0.06%, respectively. No growth was observed at and above 2.0% NaCl (w/v). In order to check for Na^+^ dependence of strain 3443-3Ac^T^ during autotrophic growth, cells were cultivated in five consecutive passages in a Na^+^-depleted minimal medium (the contaminating amount of Na^+^ was less than 100 μM) or a Na^+^-enriched medium containing 70 mM NaCl. The growth rate was similar in all passages indicating that Na^+^ concentration did not affect growth. The growth rate and yield in the fifth passage is shown on Figure 1D. Strain 3443-3Ac^T^ was able to grow in a vitamin-free medium; however, the growth yield and rate were about 30% lower in this case. The doubling time under the optimal growth conditions (60°C, pH 6.0, no additional NaCl) was 4.5 h.

**FIGURE 2:**
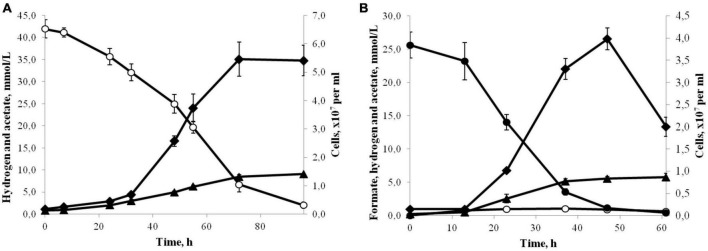
**(A)** Growth (♦) and production of acetate (▲) by strain 3443-3Ac^T^ in medium under H_2_(°)/CO_2_ at 50°C and pH 6.0. **(B)** Growth (♦) and production of acetate (▲) and H_2_(°) by strain 3443-3Ac^T^ in medium with formate (●) at 50°C and pH 6.0. Error bars indicate standard deviations of biological replicates (*n* = 3).

Strain 3443-3Ac^T^ grew chemolithoautotrophically, optimally with H_2_ as the energy source and HCO_3_^–^/CO_2_ as the carbon source and electron acceptor (Figure 2A). Acetate was the only product of the growth. The isolate also used formate (25 mM) as the energy source, but the growth yield was lower compared to growth on H_2_ (Figure 2B). The main end products during growth with formate were acetate and CO_2_, but small amounts of hydrogen were also formed. Formate was converted to acetate (and CO_2_) with a ratio of 4.2:1. The hydrogen yield reached 1 mmol L^–1^ culture in the exponential phase of growth, but decreased to 0.5 mmol L^–1^ in the stationary phase. Growth experiments for determination of CO consumption were performed exactly as described for *Thermoanaerobacter kivui* by Weghoff and Müller (2016). The cells were pre-grown on H_2_ and CO_2_ and transferred to the medium with 10% CO in the gas phase (the gas phase contained CO_2_ and did not contain H_2_). By increasing the CO concentration in 10% increments, it was possible to adapt strain 3443-3Ac^T^ to grow on up to 50% CO (the gas phase contained also 50% CO_2_). Acetate was the main product of the growth on CO, but small amounts of hydrogen were also formed. Strain 3443-3Ac^T^ was unable to utilize yeast extract, beef extract, peptone, pectin, dextrin, starch, cellulose, xylan (0.2, 1.0, or 2.0 g L^–1^ each), glucose, fructose, galactose, mannose, arabinose, rhamnose, xylose, ribose, trehalose, cellobiose, sucrose, lactose, maltose, raffinose, sorbitol, mannitol (0.5, 2.0 or 5.0 g L^–1^ each), acetate, lactate, pyruvate, malate, propionate, butyrate, fumarate, succinate, citrate, ethanol, propanol, glycerol, methanol (5, 20, or 40 mM each), 2-methoxyphenol, 3,4-dimethoxybenzoate, or 2-methoxybenzoate (1, 5, or 10 mM each). With H_2_/CO_2_ (80:20) or formate (20 mM), strain 3443-3Ac^T^ did not use nitrate, sulfate, perchlorate, fumarate, Fe (III) citrate (10 mM each), nitrite, sulfite (both 5 mM), or O_2_ (2 or 5%) as the electron acceptors. With H_2_/CO_2_ (80:20) or formate (20 mM) sulfide formation was observed on medium with thiosulfate (10 mM) or elemental sulfur (10.0 g L^–1^), but acetate was the main product of growth, and growth rate and cell yield were the same as in the absence of these acceptors. No growth was observed on glucose (1.0 g L^–1^) in the presence of thiosulfate (10 mM) or elemental sulfur (10.0 g L^–1^).

To confirm the obligatorily autotrophic nature of strain 3443-3Ac^T^, we measured the consumption of glucose and cellobiose in its cell extracts (Supplementary Table 1). Glucose and cellobiose were chosen because both sugars can be viewed as starting points of glycolysis [substrates for hexokinase or a phosphotransferase system (PTS)], and because all of the genes encoding glycolysis/gluconeogenesis enzymes, including hexokinase, and a gene of cellulose-phosphorylase (GH94) domain-containing protein but not PTS genes were found in the genome of strain 3443-3Ac^T^ (see Supplementary Table 2 and section “3.6. The likely causes for the inability of strain 3443-3AcT to grow heterotrophically”). Moreover, both glucose and cellobiose are used by all of the closest relatives of strain 3443-3Ac^T^ [representatives of the genera *Thermoanaerobacterium*, *Thermoanaerobacter*, *Caldanaerobacter*, *Calorimonas*, and *Caldanaerobius* (see section “3.2. Phylogenetic position and taxonomic status of strain 3443-3Ac^T^”)]. No consumption of glucose or cellobiose by the cell extract of strain 3443-3Ac^T^ was observed. The lack of cellobiose consumption is consistent with the results of strain 3443-3Ac^T^ genome analysis, which showed almost total absence of PTS genes, as well as of beta-glucosidase encoding genes (see section “3.6. The likely causes for the inability of strain 3443-3AcT to grow heterotrophically”). The presence of a hexokinase gene (Supplementary Table 2), prompted us to look for the respective activity in strain 3443-3Ac^T^ cell extract, and this activity was not detected. Altogether, the results of the tests with cell extracts support our conclusion about the obligatorily autotrophic nature of strain 3443-3Ac^T^.

Analysis of cellular fatty acids revealed iso-C_15:0_ (52.8% of the total fatty acids) and C_16:0_ (40.4%) as the major components. The following fatty acids were also detected: iso-C_17:0_ (4.8%), C_18:0_ (0.9%), iso-C_16:0_ (0.8%), and anteiso-C_17:0_ (0.4%) (Supplementary Table 3). Polar lipids of strain 3443-3Ac^T^ included four unidentified phospholipids and an unknown aminophospholipid (possibly, similar to phosphatidylethanolamine, but with larger R_f_1 and lower R_f_2 values). Thin-layer chromatography coupled to mass spectrometry (TLC-MS) revealed no respiratory quinones.

### 3.2. Phylogenetic position and taxonomic status of strain 3443-3Ac^T^

The genome of strain 3443-3Ac^T^ (see below) contains four almost identical (99.8–100%) 16S rRNA genes. BLAST search revealed 93–94% sequence identity to homologous genes of representatives of the genus *Thermoanaerobacterium*, and 86–88% sequence identity to those of *Thermoanaerobacter* spp. and *Caldanaerobacter* spp.

In the latest version (as of April 2022) of *Bergey’s Manual of Systematics of Archaea and Bacteria (BMSAB)* (see text footnote 3), the genus *Thermoanaerobacterium* is assigned to *Thermoanaerobacterales* Incertae Sedis Family III, whereas the genera *Thermoanaerobacter* and *Caldanaerobacter* are in the family *Thermoanaerobacteraceae* within the order *Thermoanaerobacterales*, which in addition include 8 and 12 other genera, respectively. The development of phylogenomic analyses based on ribosomal and other conserved protein sequences opens new windows into microbial taxonomy. In the GTDB (Release 07-RS207) bacterial taxonomy, which is based on concatenated alignment of 120 ubiquitous, single-copy proteins, covering hundreds of thousands genomes (Parks et al., 2018, 2020), only three genera *Thermoanaerobacterium*, *Thermoanaerobacter*, and *Caldanaerobacter* comprise the family f_Thermoanaerobacteraceae within the order o_Thermoanaerobacterales. The structure of higher taxa in GTDB is strongly different from that in the more traditional *BMSAB* taxonomy, and, whereas the GTDB o_Thermoanaerobacterales is within class c_Thermoanaerobacteria in phylum p_ Firmicutes_A, most of the genera assigned to order *Thermoanaerobacterales* in *BMSAB* fall into other higher taxa in GTDB, such as class c_Thermosediminibacteria and various classes within phylum p_Firmicutes_B.

**FIGURE 3:**
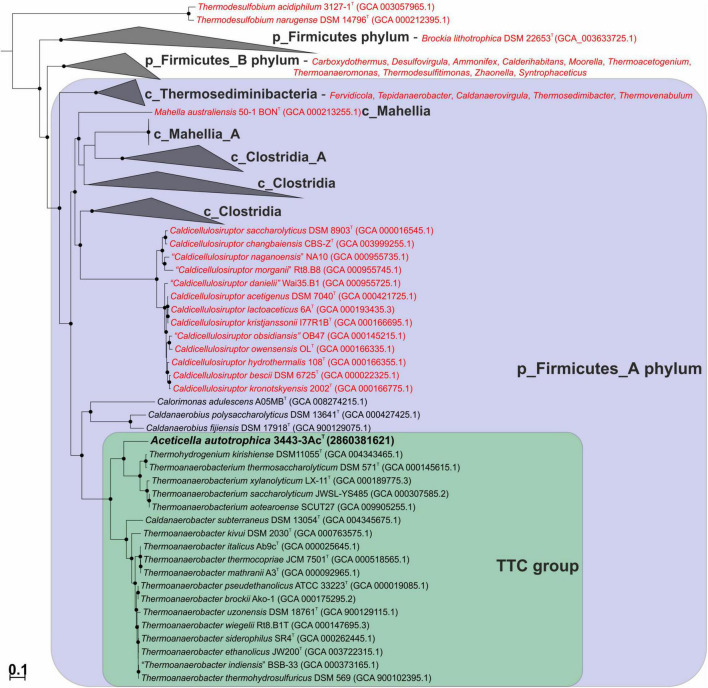
Maximum-likelihood phylogenetic tree, based on the bac120 set of conserved proteins, showing position of strain 3443-3Ac^T^ (in bold) among the relatives. *Thermoanaerobacterales* species that have been misclassified and should be affiliated to other taxa are in red. Conversely, *Thermohydrogenium kirishiense* should be reclassified as *Thermoanaerobacterium* species. The branch lengths correspond to the number of substitutions per site according to the corrections associated with the model used. The black circles at nodes indicate that the corresponding support values are above 50%. *Melioribacter roseus* P3M-2^T^ (not shown) was used as an outgroup.

We constructed a phylogenetic tree based on the bac120 set of conserved proteins for a collection of bacteria that included our strain 3443-3Ac^T^, all of the *BMSAB* order *Thermoanaerobacterales* representatives with available genomes, and representatives of the GTDB phylums p_Firmicutes and p_Firmicutes_A (see Methods for further details). The tree that we constructed (Figure 3) agreed on the whole with the GTDB phylogenetic taxonomy and showed that our strain 3443-3Ac^T^ belonged to a monophyletic clade that included *Thermoanaerobacterium*, *Thermoanaerobacter* and *Caldanaerobacter* representatives (hereafter, TTC group, corresponding to the GTDB family f_Thermoanaerobacteraceae), as well as representatives of the genera *Calorimonas* and *Caldanaerobius*. Strain 3443-3Ac^T^ formed a separate genus-level branch within the TTC cluster.

Thus, based on phenotypic (Table 1), genotypic and phylogenetic characteristics of our isolate, strain 3443-3Ac^T^ is considered to represent a new species of a new genus, for which the name *Aceticella autotrophica* gen. nov., sp. nov. is proposed.

**TABLE 1:** The principal characteristics distinguishing the genus *Aceticella* gen. nov. from the genera *Thermoanaerobacterium* [data from Yamamoto et al. (1998), Romano et al. (2010), Shang et al. (2013), Onyenwoke and Wiegel (2015), and Lopez et al. (2017)], *Thermoanaerobacter* [data from Wagner et al. (2008), Tomas et al. (2013), and Onyenwoke and Wiegel (2015)], and *Caldanaeobacter* [data from Kim et al. (2001), Kozina et al. (2010), and Fardeau et al. (2015)].

Property	*Aceticella* (1)	*Thermoanaerobacterium* (9)	*Thermoanaerobacter* (16)	*Caldanaerobacter* (2)
Isolation source	Hot springs	Geothermal sites, hot springs, canned food, dung samples	Geothermal sites, hydrothermal vent, hot springs, water and mud samples from thermal spas, geothermally heated oil samples, extraction juices of beet sugar factories, mesobiotic mud and soil, sugar refinery, household dust, compost of camel feces, spoiled meat, thermophilic reactor fed with household solid waste from a biogas plant	Hot spring, submarine hydrothermal vent, geothermal water, oil well
Cell morphology	Rod-shaped cells occur singly, in pairs or in chains depending on the growth phase and growth conditions.	Rod-shaped cells occur singly, in pairs or in chains depending on the growth phase and growth conditions	Rod-shaped cells occur singly, in pairs or in chains depending on the growth phase and growth conditions. Asymmetric cell division leads to formation of coccoid cells. Stationary growth phase cells are pleomorphic, and some form filaments.	Rod-shaped cells occur singly, in pairs or in chains depending on the growth phase and growth conditions. Cells sometimes branch.
Cell wall	Gram-positive cell-wall structure and Gram-stain- positive	Cells have Gram-positive cell-wall structure, but are Gram-stain-variable	Cells have Gram-positive cell-wall structure, but are Gram-stain-variable	Cells have Gram-positive cell-wall structure, but are Gram-stain-variable
Motility	–	+	V	V
Spores	+	V	V	V
Growth temperature (°C)				
Range	30–60	35–75	30–85	40–85
Optimum	50	50–60	55–71	65–75
Growth pH				
Range	4.5–7.3	3.8 – 8.5	4.0–9.9	4.5–9.2
Optimum	6.0	5.2 –7.8	5.8–8.5	6.5–7.5
NaCl for growth (%, w/v)				
Range	0–1.5	0–3.0	0–4.5	0–4.0
Optimum	0	0.1–2.5	0.5–1	0–2.5
Chemoorganoheterotro phic growth	–	+	+	+
Chemolithoheterotrophic growth	–	nd	V	V
Chemolithoautotrophic growth	+	–	V*	–
Substrate utilization				
Glucose	–	+	+	+
Fructose	–	V	+	+
Galactose	–	V	V	+
Mannose	–	+	+	+
Xylose	–	+	V	V
Cellobiose	–	+	V	+
Lactose	–	V	V	+
Maltose	–	+	V	+
Sucrose	–	V	V	V
Pectin	–	V	V	nd
Starch	–	V	V	+
Xylan	–	+	V	V
Cellulose	–	–	V	nd
Fermentation end products from hexoses	NA	Ethanol, acetate, lactate, H_2_ and CO_2_ (In some instances, butyrate and butanol are also formed)	Ethanol, acetate, lactate, H_2_ and CO_2_ (In some instances, butyrate, isobutyrate, propionate and succinate are also formed)	Acetate, H_2_, CO_2_,L-alanine, ethanol and lactate (In some instances, propionate, isobutyrate and isovalerate are also formed)
Gelatin hydrolysis	–	V	V	–
Electron acceptors				
Sulfite	–	V	V	V
Sulfur	+	V	V	V
Thiosulfate	+	+	+	+
Nitrate	–	V	V	V
Acetogenesis capacity	+	–	V**	–
DNA G+C content (mol%)	33.0	29–36.1	30–38	33–41

Numbers of species are indicated in parenthesis. +, positive; –, negative; V, characteristic varies among different species; nd, not determined; NA, not applicable.

*Chemolithoautotrophic growth was shown only for *Thermoanaerobacter kivui* (H_2_+CO_2_ and CO+CO_2_).

**Acetogenesis capacity was shown only for *Thermoanaerobacter kivui*.

### 3.3. General genome properties, genome mobility, and genomic islands

Hybrid sequencing and *de novo* genome assembly allowed the full sequence of the 3443-3Ac^T^ circular chromosome to be obtained. The chromosome length was 2,267,618 nucleotides. *In silico* determined G+C content of the genomic DNA of strain 3443-3Ac^T^ was 33.0 mol%. Automatic gene calling and annotation resulted in the prediction of 2234 total genes including 2093 protein-coding, 72 RNA genes, and 69 pseudogenes.

**FIGURE 4:**
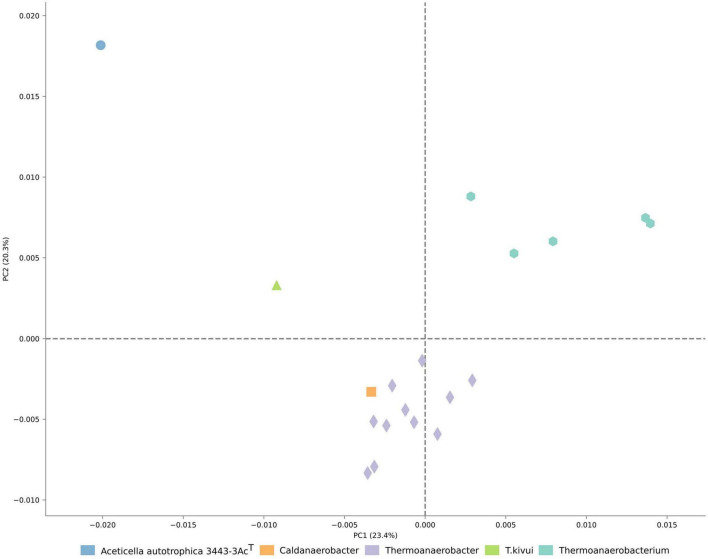
Ordination of the distribution of clusters of orthologous genes in proteomes of TTC representatives.

The analysis of COG distribution in the proteome of strain 3443-3Ac^T^ with comparison to TTC group representatives, performed with STAMP software (Parks et al., 2014), showed remote positioning of strain 3443-3Ac^T^ relative to other TTC group genera (Figure 4), reflecting its metabolic peculiarities.

Analysis of genome mobility-related features showed that strain 3443-3Ac^T^ can be characterized by the largest proportion of IS-element-related genes among TTC group representatives taken for comparative analysis (Supplementary Figure 2). Analysis of phage-related genes showed at least two incomplete prophage regions of 6.9 and 11.4 kb, possessing 75 and 62% of phage-associated genes, respectively (Supplementary Figure 3).

In accordance with the high level of predicted genome mobility, analysis of laterally acquired genomic regions revealed 13 genomic islands (GI), spanning over 330 kb (Supplementary Figure 3 and Supplementary material 2). The COG functional categories distribution showed that these laterally transferred loci were specifically enriched with mobile elements and genes involved in defense mechanisms (Supplementary material 3). Specifically, GI2 and GI3 possessed two Cas operons of types IB and IIIB, respectively. GI4 includes two restriction-modification systems of classes I and II (ACETAC_01575-ACETAC_01585 and ACETAC_01520-ACETAC_01530, respectively). GI5 possesses a gene for homodimeric lactococcal bacteriocin - lactococcin 972 (Martínez et al., 1996). GI6 codes for a four-gene Wadjet system (Doron et al., 2018), GI7 possesses phosphorothioation-sensing bacterial defense system SSpBCDE (Xiong et al., 2020). The large GI10 includes several genes for bacteriocin-processing C39 peptidases (Kanonenberg et al., 2013), as well as bacteriocin genes and s toxin/antitoxin systems (Supplementary material 2).

Search for CRISPR-Cas genomic immunity modules performed with cctyper web server (Russel et al., 2020) discovered five Cas operons; however, only one of them (IB type, ACETAC_00735- ACETAC_00770), possessing full adaptation and interference modules, as well as adjacent CRISPR repeat, seems to be functional (Supplementary Figure 4 and Supplementary Table 4). Others were either heterogeneous, located in the genomic islands (see above), or disrupted by IS-element mediated rearrangements.

### 3.4. Genomic determinants of acetogenesis in strain 3443-3Ac^T^

**FIGURE 5:**
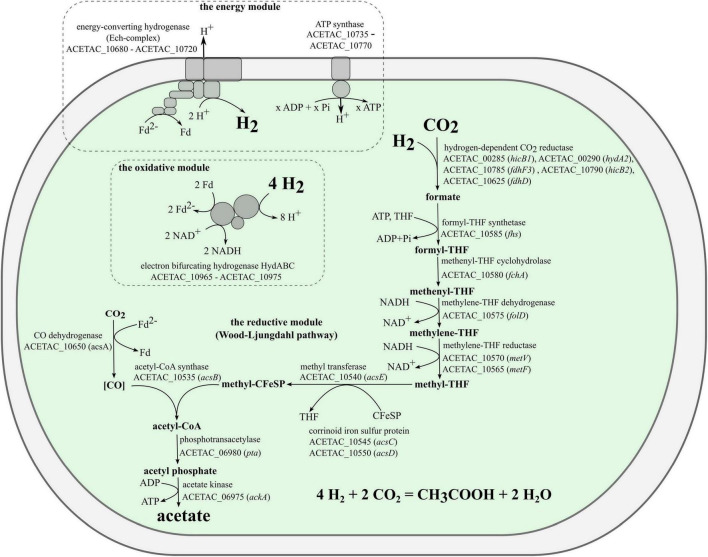
Model for acetogenesis on hydrogen in strain 3443-3Ac^T^.

The genome of strain 3443-3Ac^T^ encoded all the proteins (Supplementary Table 5) needed for chemolithoautotrophic acetogenic growth with hydrogen (Figure 5). The electron-donating reactions (the first module of acetogenesis) are implemented by the soluble electron-bifurcating [FeFe]-hydrogenase (HydABC complex, ACETAC_10965–ACETAC_10975), which is homologous (47–58% amino acid sequence identity of subunits) to the biochemically characterized bifurcating hydrogenases of the acetogenic bacteria *Acetobacterium woodii* and *Moorella thermoacetica* (Schuchmann and Müller, 2012; Wang et al., 2013), and still more similar (79–84% identity of subunits) to the HydABC of the acetogenic *Thermoanaerobacter kivui*, for which the properties have been deduced based on the homology with the aforementioned proteins from *A. woodii* and *M. thermoacetica* (Hess et al., 2014). Apart from the HydABC complex and HydA2, a component of the hydrogen-dependent CO_2_ reductase (see below), there are no other uptake hydrogenases encoded in the genome of strain 3443-3Ac^T^. As for H_2_-producing hydrogenases, the genome contains genes for Ni-containing membrane-bound energy-converting hydrogenase, which generates ion-motive force in the course of reduction of protons to molecular hydrogen (see below).

Electrons coming from molecular hydrogen are transferred by the electron-bifurcating hydrogenase to the universal electron carriers NAD^+^ and ferredoxin. A portion of the reduced ferredoxin is used in the reductive reactions of Wood-Ljungdahl pathway (the second module of acetogenesis), and the remaining portion is used to generate a chemiosmotic gradient (the third module). One CO_2_ molecule is reduced to a methyl group in the methyl branch of the WLP and another CO_2_ molecule is reduced to CO in the carbonyl branch. The first step in the methyl branch is the reduction of CO_2_ to formate. We predict that in strain 3443-3Ac^T^ this reaction is carried out by a hydrogen-dependent CO_2_ reductase (HDCR), an enzyme discovered in *Acetobacterium woodii* (Schuchmann and Müller, 2013) and then biochemically characterized also in *Thermoanaerobacter kivui* (Schwarz et al., 2018). This enzyme complex was shown to catalyze the reduction of CO_2_ with electrons coming directly from molecular hydrogen. The complex includes a hydrogenase subunit and a formate dehydrogenase subunit, which are connected by two electron-transferring subunits (Schuchmann and Müller, 2013; Schwarz et al., 2018). In both *Acetobacterium woodii* and *Thermoanaerobacter kivui*, the four genes encoding subunits of HDCR are located within one gene cluster. Both gene clusters also include a gene annotated as *fdhD*, encoding a protein with unknown but probably regulatory function. In the genome of strain 3443-3Ac^T^ the genes that we predict to encode HDCR subunits are not located in a single gene cluster. Genome analysis revealed the presence of one gene for selenium-free formate dehydrogenase, *fdhF1* (ACETAC_00565), and two genes for selenium-containing formate dehydrogenases: *fdhF2* (ACETAC_08370/ACETAC_08380) and *fdhF3* (ACETAC_10785). Of these, the *fdhF2* gene is disrupted by a transposon, whereas *fdhF1* and *fdhF3* encode proteins 76–77% identical to the only formate dehydrogenase of *Thermoanaerobacter kivui*. A gene encoding an electron transfer protein *hycB2* (ACETAC_10790) precedes the *fdhF3* gene. The hydrogenase module of HDCR is encoded by the hydrogenase subunit gene *hydA2* (ACETAC_00290) and the nearby electron-transferring subunit gene *hycB1* (ACETAC_00285). The aforementioned *fdhD* gene of the unknown relevance to HDCR is also present in strain 3443-3Ac^T^ genome (ACETAC_10625), although apart from the apparently relevant genes. Despite the weakly pronounced clustering of the HDCR genes homologs in strain 3443-3Ac^T^ genome, we predict the HDCR function based on the high identity values of the respective proteins with the subunits of the biochemically characterized HDCR of *Thermoanaerobacter kivui* (73–82%, Supplementary Table 5).

**FIGURE 6:**
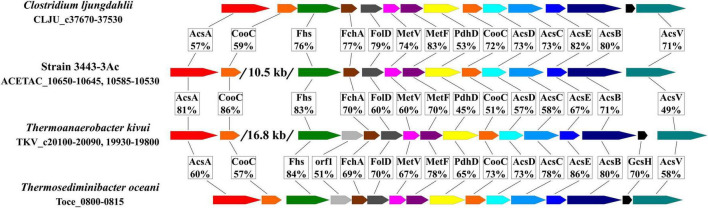
Comparison of the WLP gene cluster of strain 3443-3Ac^T^ with the gene clusters in *Clostridium ljungdahlii*, *Thermoanaerobacter kivui* and *Thermosediminibacter oceani* in terms of gene order and amino acid sequence identity of the encoded proteins. AcsA, CO dehydrogenase; CooC, maturation factor of the CO dehydrogenase; Fhs, formyl-THF synthetase, FchA, methenyl-THF cyclohydrolase; FolD, methylene-THF dehydrogenase; MetV and MetF, methylene-THF reductase; PdhD, dihydrolipoamide dehydrogenase; AcsD and AcsC, corrinoid iron sulfur protein; AcsE, methyl transferase; AcsB, acetyl-CoA synthase; GcsH, protein H of a glycine cleavage system; AcsV, acetyl-CoA synthase corrinoid activation protein.

Other genes encoding functions of the WLP (with notable exception of the CO dehydrogenase gene *acsA*) are located in the 3443-3Ac^T^ genome in one gene cluster (hereafter, the WLP gene cluster, Figure 6 and Supplementary Table 5), *acsA* being located 10.5 kb upstream. Very similar gene clusters occur in the genomes of many representatives of the class c_Clostridia orders o_Clostridiales and o_Peptostreptococcales and of the class c_Thermosediminibacteria (GTDB taxonomy), but in the genomes of those bacteria the cluster includes the CO dehydrogenase gene *acsA* (Figure 6). Most similar to our strain’s WLP gene cluster in terms of its organization is the gene cluster of *Thermoanaerobacter kivui*, which, apart from strain 3443-3Ac^T^, is the only TTC group member that has WLP genomic determinants. In the *Thermoanaerobacter kivui*’s genome, *acsA* (one of the two *Thermoanaerobacter kivui*’s CO dehydrogenase genes) also occurs beyond the WLP gene cluster 16.8 kb upstream (Figure 6). The genome of strain 3443-3Ac^T^ also contains two CO dehydrogenase genes, and their phylogenies (see below) suggests that it is ACETAC_10650 that is involved in the WLP in strain 3443-3Ac^T^ (and thus should be termed *acsA*). The ACETAC_02795 gene is located far away from the WLP gene cluster and encodes a CO dehydrogenase with a 96% amino acid sequence identity with the biochemically characterized monofunctional CO dehydrogenase CooS of *Thermoanaerobacter kivui* (Schwarz et al., 2020; Jain et al., 2021). Thus, the ACETAC_02795 product is apparently involved in CO oxidation during growth on that substrate.

At the final acetogenesis stage, phosphotransacetylase (*pta* - ACETAC_06980) and acetate kinase (*ackA* - ACETAC_06975) catalyze the conversion of acetyl-CoA to acetate with ATP synthesis from ADP + P_i_. However, this substrate-level phosphorylation reaction does not solve the net problem of energy conservation since the ATP molecule is spent at the stage of formate activation by formyl-tetrahydrofolate (THF) synthetase (*fhs* - ACETAC_10585) in the methyl branch of WLP.

For energy conservation, acetogenic bacteria use a two-modular respiration system comprising either Rnf complex or Ech complex generating the ion-motive force (IMF) and an ATP synthase using IMF for ATP synthesis. Inspection of the genome showed that strain 3443-3Ac^T^ does not possess an Rnf complex, but has a gene cluster that we predict to encode subunits of a Ni-containing energy-converting hydrogenase (Ech complex, ACETAC_10680 - ACETAC_10720). This gene cluster comprises 9 genes (Supplementary Table 5) and is highly similar (in terms of encoded proteins sequence identity, gene order and phylogeny) to one of the two Ech-encoding gene clusters found in the genome of *Thermoanaerobacter kivui*, namely, to that encoded by TKV_c01230 - TKV_c01310 (Schoelmerich and Müller, 2019). The identity of homologous subunits of these Ech complexes of strain 3443-3Ac^T^ and *Thermoanaerobacter kivui* is 70–92%. An interesting point, supporting the vertical inheritance of the cluster, is the presence in both of these gene clusters of genes encoding proteins (ACETAC_10695, 74% identical to TKV_c01280, and ACETAC_10690, 80% identical to TKV_c01290) whose homologs have never been reported to occur as subunits in any hydrogenases studied and that have never been noticed to occur in any other gene clusters recognized to encode hydrogenases.

The Ech complex of strain 3443-3Ac^T^ is apparently the only candidate for the mechanism of coupling electron transfer with ion translocation in this bacterium: the genome of 3443-3Ac^T^ does not encode an Rnf complex or a NADH-quinone oxidoreductase. Moreover, neither quinones were found by TLC-MS nor quinone biosynthetic pathways were revealed during the genome analysis. Genes encoding the subunits of a F_1_F_O_ ATP synthase occur in the genome of strain 3443-3Ac^T^ as a single gene cluster (ACETAC_10735 - ACETAC_10770) located upstream of the *ech* gene cluster (Supplementary Table 5). The ATP synthase subunit *c* of strain 3443-3Ac^T^ (encoded by *atpE* - ACETAC_10765), like that of *Thermoanaerobacter kivui* (Hess et al., 2014), does not exhibit the Q…..ES/T Na+ binding motif and features the E55 proton-binding site. Thus, we predict that strain 3443-3Ac^T^ uses a respiration system comprising an Ech complex and an H^+^-dependent ATP synthase for energy conservation.

### 3.5. Genomic determinants of formate and carbon monoxide metabolism in strain 3443-3Ac^T^

The first step in formate metabolism is its uptake, but the genome of strain 3443-3Ac^T^ lacked genes encoding known formate transporters (PF01226, Form_Nir_trans). Recently it has been shown that formate transporter is not essential for growth of *A. woodii* on a high formate concentration (50–500 mM) at pH 7.0. *A. woodii* mutant with deleted formate transporter was capable to grow under these conditions because part of the formate was in the form of uncharged formic acid (Moon et al., 2021), which can diffuse across membranes (Falke et al., 2010). At the optimal concentration of 200 mM and pH 7.0 (pKa_formic acid_ = 3.75), 0.1 mM of the substrate was in the form of formic acid, and 199.9 mM was present as formate (Moon et al., 2021). Regrettably, growth of *A. woodii* mutant with deleted formate transporter gene has not been tested at formate concentrations below 50 mM. Meanwhile, strain 3443-3Ac^T^ grew on 25 mM formate at lower pH (pH 5.8) and 50*^o^*C, which implies that 0.2 mM of the substrate was in the form of formic acid, allowing us to assume that the uptake of formate in strain 3443-3Ac^T^ is indeed due to diffusion of formic acid through the membrane.

Strain 3443-3Ac^T^ converted formate to acetate (and CO_2_, which was not measured) with a ratio of 4.2:1.0 (Figure 2B). This is comparable to the ratio of 4.4:1.0 obtained for *A. woodii* (Moon et al., 2021). We suppose that the pathway of acetogenesis from formate in strain 3443-3Ac^T^ is similar to the pathway postulated for *A. woodii* (Moon et al., 2021) with an important distinction that the Ech complex operates instead of the Rnf complex. Of four molecules of formate metabolized, three are oxidized to CO_2_ and H_2_ by HDCR, whereas one molecule of formate is converted to formyl-THF, reduced further to methyl-THF in the methyl branch of the WLP (Supplementary Figure 5). In the carbonyl branch, CO_2_ is reduced to CO, which is bound to the methyl group by the CODH/ACS complex, generating acetyl-CoA, further converted to acetate. Balance of reducing equivalents is achieved by the interplay of HDCR, bifurcating hydrogenase HydABC, and Ech complex (Supplementary Figure 5): HydABC oxidizes the H_2_ produced by HDCR and Ech, thus forming reduced ferredoxin and NADH, and Ech oxidizes part of the reduced ferredoxin with the formation of H_2_ and generation of a chemiosmotic gradient.

The key enzyme in CO utilization is carbon monoxide dehydrogenase. The genome of strain 3443-3Ac^T^ contains two CO dehydrogenase genes (see above). Based on the high sequence identities of the CO dehydrogenases of strain 3443-3Ac^T^ with the biochemically characterized CO dehydrogenases of *Thermoanaerobacter kivui*, we predict that the ACETAC_10650 gene is the *acsA*, encoding the CO dehydrogenase component of the CODH/ACS complex, whereas the ACETAC_02795 gene is to be termed *cooS* and it encodes a monofunctional CO dehydrogenase. Thus, CO is oxidized by CooS, reducing ferredoxin, reoxidized then by the Ech complex with the formation of H_2_ and generation of chemiosmotic gradient (Supplementary Figure 6). We assume that part of the H_2_ formed is used by HDCR to reduce CO_2_ to formate, and the other part is oxidized by the bifurcating hydrogenase HydABC to produce NADH, required by the WLP methyl branch. CO and formate are further converted to acetate via the WLP.

The proposed metabolic schemes of strain 3443-3Ac^T^ growth on either formate or CO suggest balance of reducing equivalents without production of hydrogen or consumption of exogenous CO_2_. However, in both cases H_2_ is an essential intracellular intermediate, and due to its high diffusivity, part of it should be prone to escape the cell. Actually, small hydrogen production was observed both on formate and CO in our experiments, as it was observed during the growth on CO of *T. kivui* (Weghoff and Müller, 2016), for which an analogous metabolic scheme has been proposed (Schoelmerich and Müller, 2019).

It should be pointed out that the proposed schemes of strain 3443-3Ac^T^ metabolism during growth on H_2_+CO_2_, HCOOH, and CO (Figure 5 and Supplementary Figures 5, 6) suggest operation of the same enzymatic machinery, with the distinctions being the involvement of CooS in the growth on CO and the opposite direction of HDCR operation in case of growth on formate.

### 3.6. The likely causes for the inability of strain 3443-3Ac^T^ to grow heterotrophically

All representatives of the genera *Thermoanaerobacterium*, *Thermoanaerobacter* and *Caldanaerobacter* (TTC group) are chemoorganoheterotrophic bacteria able to grow by fermenting sugars. Strain 3443-3Ac^T^, is thus an exception, being, according to our knowledge, the first obligately autotrophic representative of the TTC group. Genome analysis showed that genes coding for glycolysis/gluconeogenesis enzymes are present in genomes of all representatives the TTC group, including strain 3443-3Ac^T^ (Supplementary Table 2).

**FIGURE 7:**
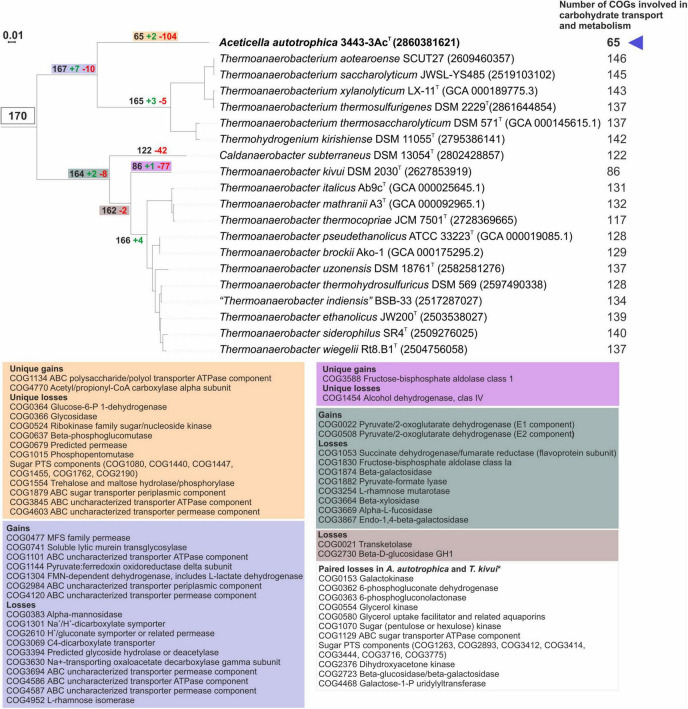
Analysis of gains and losses of genes involved in carbohydrate transport and metabolism in genomes of TTC group members (including strain 3443-3Ac^T^). Colored boxes specify list of gained/lost genes and corresponding nodes on the phylogenetic tree. *Genes that were lost in parallel by strain 3443-3Ac^T^ and *Thermoanaerobacter kivui* but are retained in other TCC group members. All protein functions are given according to official COG names list.

To decipher the inability of strain 3443-3Ac^T^ to grow on carbohydrates, genes gain and loss analysis within the TTC phylogenetic cluster was performed. Comparison of the detected full COG sets (Supplementary Figure 7) and COG sets involved in carbohydrate transport and metabolism (Figure 7) showed a drastic decrease in the number of carbohydrate metabolism-related genes in strain 3443-3Ac^T^ (Figure 7 and Supplementary material 4), while total number of COGs was similar to those in other representatives of the TTC cluster. The single acetogen in the TTC group (apart from strain 3443-3Ac^T^), *Thermoanaerobacter kivui* DSM 2030, had a similar profile of gene losses, but in *T.kivui*’s case the losses do not happen to be critical for the ability to utilize sugars. To reveal these critical losses, we compared unique gains/losses of *Aceticella autotrophica* with those of *T*. *kivui* among COGs involved in carbohydrate transport and metabolism, and also payed attention to paired losses among these COGs (i.e., losses that were parallel in these two organisms but did not occur in other TTC group members). Overall, strain 3443-3Ac^T^ had much more unique losses compared to *T*. *kivui*, and these losses mainly affected putative sugar-specific or uncharacterized transport systems, glycoside hydrolases and some other sugar-active enzymes. Among paired losses of strain 3443-3Ac^T^ and *T*. *kivui*, there were genes encoding various sugar-specific transporters, such as phosphotransferase system (PTS) transporters; sugar-specific adenosine triphosphate-binding cassette (ABC) transporters; and other permeases. As for the PTS, *T*. *kivui* genome encoded two complete PTSs consisting of IICB-IIA and IIA-IIC-IIB components while in the genome of strain 3443-3Ac^T^ only a gene fragment of a PTS putative EIIB component (ACETAC_00175) was found, indicating the PTS loss might be one of the losses critical to make 3443-3Ac^T^ an obligate autotroph. Interestingly, a gene encoding a cellulose-phosphorylase (GH94) domain-containing protein is present in the genomes of both strains as well as in other TTC members, which may be another indication of an operational PTS in strain 3443-3Ac^T^ ancestors. Additional paired losses included genes encoding enzymes involved in carbohydrate catabolism: sugar kinases, isomerases and transferases required for carbohydrate interconversions before entry into central catabolic pathways. Enzymes of galactose catabolism - galactokinase and galactose-1-phosphate uridylyltransferase - were also among paired losses. Oxidative branch of pentose phosphate pathway has undergone impairment in both organisms, due to the loss of phosphogluconolactonase, decarboxylating 6-phosphogluconate dehydrogenase and, in strain 3443-3Ac^T^, also of glucose-6-P 1-dehydrogenase (a unique loss of the strain).

Thus, we suggest that the inability of strain 3443-3Ac^T^ to use sugars as carbon and energy sources is associated with the loss of sugar transport systems and enzymes involved in carbohydrate catabolism.

### 3.7. On the likely evolutionary origin of acetogenic metabolism in the TTC phylogenetic group

Strains 3443-3Ac^T^ and *Thermoanaerobacter kivui* DSM 2030^T^ (the single strain of the *T. kivui* species) are the only two organisms among members of the TTC group that possess WLP and exhibit acetogenesis capacity. Thus, a question arises as to whether the WLP and the acetogenesis capacity were acquired by strain 3443-3Ac^T^ and *Thermoanaerobacter kivui* via horizontal transfer events or were inherited vertically from a common ancestor. The latter scenario implies losses of acetogenesis determinants in other species of the TTC group.

Neither of acetogenesis determinants is acetogenesis-specific. Instead, as discussed in the section “1. Introduction,” the acetogenesis capacity requires the presence of a particular combination of enzymes and enzymatic complexes each of which may be involved in other processes. This required combination includes (1) WLP enzymes, (2) an enzymatic complex coupling ion translocation with electron transfer from ferredoxin to a low-potential acceptor such as H+ or NAD+ (Ech-hydrogenase or Rnf, respectively), and (3) an enzymatic machinery for the production of reduced ferredoxin, the donor for Ech-hydrogenase or for Rnf. Mere presence of WLP enzymes may mean just the presence of a pathway of autotrophic CO_2_ fixation coupled to a mechanism of energy generation different from those employed in acetogenesis or be indicative of acetate oxidation capacity. Nevertheless, it seems appropriate to consider the evolutionary origin of acetogenesis in strain 3443-3Ac^T^ beginning with tracing the evolution of WLP determinants, first of all, of the WLP-specific carbon monoxide dehydrogenase/acetyl-CoA synthase (CODH/ACS) enzymatic complex, whose role in the case of acetogenesis is the reduction of CO_2_ to CO and further condensation of CO with methyl group, resulting in acetyl-CoA production. This complex occurs in both bacteria and archaea; in bacteria, the encoding genes are termed *acsABCDE*.

To trace the origin of the specific key determinants of WLP in strain 3443-3Ac^T^ and *T. kivui*, the only representatives of the TTC group in which these genes have been found, we constructed phylogenetic trees for their AcsABCDE proteins. Four of these five trees (those of AcsBCDE) exhibited similar topologies (Supplementary Figures 8–12 and Supplementary material 5): the proteins of 3443-3Ac^T^ occurred within clades formed by proteins belonging to representatives of the GTDB orders o_Clostridiales and o_ Peptostreptococcales within class c_Clostridia, whereas proteins of *T.kivui* were found among proteins of class c_Thermosediminibacteria members. This suggests that the ancestors of strain 3443-3Ac^T^ and *T. kivui* acquired the *acsBCDE* genes independently via HGT from different donors.

As discussed above, in strain 3443-3Ac^T^ and *T. kivui*, as well as in many representatives of the class c_Clostridia orders o_Clostridiales and o_Peptostreptococcales and of class c_Thermosediminibacteria (GTDB taxonomy), the *acsBCDE* genes occur in a common cluster (the WLP cluster) with some accessory genes and with genes responsible for the WLP methyl branch (Figure 6 and Supplementary material 5). Phylogenetic trees that we constructed for the products of these genes (Supplementary Figures 13–20) showed topologies similar to those of the AcsBCDE proteins: the proteins of strain 3443-3Ac^T^ were among proteins of c_Clostridia, whereas the proteins of *T.kivui* were among or close to proteins of c_Thermosediminibacteria. Thus, the WLP gene clusters were likely acquired by (ancestors of) strain 3443-3Ac^T^ and *T. kivui* as entire units via independent multigenic transfer events. Certain differences between the topologies of particular trees are likely to result from subsequent gene replacements via HGT across shorter phylogenetic distances. The members of the TTC group seem prone to ready acquisition of genetic information, as evidenced by demonstration of wide occurrence of natural competence among *Thermoanaerobacter* and *Thermoanaerobacterium* species (Shaw et al., 2010; Zeldes et al., 2023).

Given this phylogeny of the specific key determinants of WLP in strain 3443-3Ac^T^ and *T. kivui* and lack of WLP in other TTC group members, it seemed natural to infer two independent events of *de novo* emergence of WLP and thus acetogenesis capacity in strain 3443-3Ac^T^ and *T. kivui*. However, further genomic analysis revealed facts that prompted us to also consider another scenario for the presence of WLP and acetogenesis capacity in the two strains and to choose this scenario as a more likely one. Namely, the WLP gene clusters currently occurring in the genomes of strain 3443-3Ac^T^ and *T. kivui* may have resulted from replacements of a vertically inherited original WLP gene cluster that was functional in an ancestor of TTC. The facts indicating the pre-existence of WLP are as follows:

(1) The strain 3443-3Ac^T^ and *T. kivui*’s *acsA* (CO dehydrogenase) genes exhibit unusual location in the genomes (they occur beyond the WLP gene clusters, Figure 6), and the phylogeny of these genes differs from the phylogeny of the WLP gene clusters and complies with vertical inheritance from a common ancestor: in CO-dehydrogenase tree, the ACETAC_10650 and TKV_c20100 proteins of strain 3443-3Ac^T^ and *T. kivui* appeared as closest relatives, forming a separate lineage between CODHs of c_Clostridia and c_Thermosediminibacteria recognized as AcsAs due to clustering with AcsB in the genomes (Supplementary Figures 21, 22 and Supplementary material 6).

(2) Further support for the idea that WLP as a biochemical phenomenon was acquired by strain 3443-3Ac^T^ and *T. kivui* from a common ancestor comes from examination of phylogeny of formate dehydrogenases and of the enzyme complexes that are obligately required by WLP only when it operates as a part of acetogenesis process. Formate dehydrogenase genes (Supplementary Figure 23) and the gene clusters encoding bifurcating hydrogenase (Supplementary Figure 24) and energy-converting hydrogenase (Supplementary Figure 25) were apparently inherited by strain 3443-3Ac^T^ and *T. kivui* vertically from a common ancestor. This conclusion is based on their occurrence in distinctly separated two-member clades in phylogenetic trees and their pairwise identity values in the range of 75–85% (the AAI value between strain 3443-3Ac^T^ and *T. kivui* is 67% for complete proteomes and 74% for the 120 conserved proteins used for phylogenetic analysis). Such identity values well agree with vertical inheritance from a common ancestor.

(3) The genomes of strain 3443-3Ac^T^ and *T. kivui* encode proteins representing all 18 COGs required for biosynthesis of cobalamin, the cofactor necessary for WLP. The number of COGs of this set represented in other members of the TTC group varied from 7 to 16. This occurrence pattern agrees with the hypothesis about the presence of acetogenesis capacity in the common ancestor of the TTC group and its loss in most of the modern TTC species, with the loss rate varying in lineages.

Our conclusions are in accordance with the results of analysis of the entire evolutionary history of the CODH/ACS-encoding genes performed by Adam et al. (2018), who arrived at the conclusion that these genes were usually inherited as a single unit, their inheritance mostly followed vertical pattern, and their phylogeny can be traced to LUCA. As for Firmicutes sensu Bergey’s (corresponding to nine GTDB phyla, from p_Firmicutes to p_Firmicutes_H), their *acsAB* genes were inferred to be monophyletic, but their *acsCDE* genes were demonstrated to have a deviating evolutionary history in a particular Firmicutes lineage corresponding to GTDB phylum Firmicutes_A, where the *acsCDE* genes do not ascend to LUCA directly but are thought to result from replacement of native (vertically inherited) *acsCDE* genes by *acsCDE* genes coming from *Nitrospirae* (Adam et al., 2018). However, this replacement of particular details of the WLP mechanism cannot prevent one from assuming that, in Firmicutes, the WLP as biochemical phenomenon is monophyletic, was inherited vertically and likely ascends to LUCA. This is however quite a general overview of the evolutionary pattern, and the question is whether within particular lineages of Firmicutes the WLP could have been lost and later reacquired via horizontal transfer(s) of the necessary determinants. Based on the results of our analysis, such “loss and reacquisition” pattern does not anyway seem applicable to the case of our strain and *T. kivui*. We hypothesize that the WLP pathways in strain 3443-3Ac^T^ and *T. kivui* have not arisen independently *de novo* via horizontal gene transfers; instead, the capacity was inherited vertically from a common ancestor. Horizontal gene transfers did play a role, but this role consisted in replacement of details of the evolutionarily persisting mechanism, which has been lost by other TTC group members, or, looking at a different angle, has not been inherited by them from the ancestral pangenome.

## 4. Conclusion

Here, we described the first obligately autotrophic acetogenic bacterium *Aceticella autotrophica* gen. nov., sp. nov., strain 3443-3Ac^T^, isolated from a terrestrial hot spring of Uzon Caldera, Kamchatka. *A. autotrophica* is capable of acetogenic growth on H_2_/CO_2_, CO, or formate and is unable to grow heterotrophically, which makes it unique among acetogenic microorganisms. So far only facultative autotrophic acetogens have been known, and their metabolic versatility has been considered as the characteristic trait related to the low competitiveness of autotrophic acetogenesis in environmental conditions, where it occurs at the thermodynamic limit. We show that the inability of *A. autotrophica* to grow heterotrophically is associated with the loss of sugar-specific transport systems and many enzymes involved in carbohydrate catabolism. In ecological and evolutionary terms, the ability to survive in nature via obligately autotrophic acetogenic growth might be due to peculiarities of energy-converting mechanisms. However, our genomic analysis showed that *A. autotrophica*’s energy metabolism apparently involves cooperation of energy-converting hydrogenase, bifurcating hydrogenase, Wood-Ljungdahl pathway enzymes, and proton-dependent ATP synthase, i.e., the mechanism already described recently in so-called Ech-acetogens. Further studies are to show whether *A. autotrophica* has acquired yet unraveled additional mechanisms of energy conversion or peculiarities of enzyme kinetics allowing it to live as an obligatory acetogen.

### 4.1. Description of *Aceticella* gen. nov.

*Aceticella* (A.ce.ti.cel’la. L.n. *acetum* vinegar; L.n. *cella* cell; N.L. fem. n. *Aceticella* vinegar cell).

Cells are rod-shaped. Cell wall of Gram-positive type. Spore-forming. Moderately thermophilic. Obligate chemolithoautotrophic anaerobe, growing by acetogenesis with H_2_, formate, or CO as the energy source and HCO_3_^–^/CO_2_ as the electron acceptor. Acetate is the only end product of the acetogenic growth on H_2_. The main end product during acetogenic growth with formate or CO is acetate, but small amounts of hydrogen are also formed. In the presence of thiosulfate or elemental sulfur sulfide formation is observed, but acetate also is the main product of the growth. The type species is *Aceticella autotrophica*.

### 4.2. Description of *Aceticella autotrophica* sp. nov.

*Aceticella autotrophica* (au.to.tro’phi.ca. Gr. pron. *autos* self; Gr. adj. *trophikos* one who feeds; N.L. fem. adj. *autotrophica* autotrophic).

Cells are rod-shaped, 0.5–0.7 μm in diameter and 1.5–5 μm in length, growing singly, in pairs or in chains depending on the growth phase and growth conditions. Cells are non-motile. Spore-forming. Moderately thermophilic. Growth occurs between 30 and 60°C, with an optimum at 46–50°C. The pH range for growth is 4.5–7.3, with an optimum pH of 6.0. Growth does not occur above a NaCl concentration of 1.5% (w/v). Growth is not Na^+^-dependent. Cells are capable to grow in vitamin-free medium. Obligate chemolithoautotrophic anaerobe, growing by acetogenesis with H_2_ as the energy source and HCO_3_^–^/CO_2_ as the carbon source and the electron acceptor. Acetate is the only end product of the growth on H_2_ and HCO_3_^–^/CO_2_. Formate or CO can also be used as the energy source. The main end product during growth with formate or CO is acetate, but small amounts of hydrogen are also formed. No growth occurs with yeast extract, beef extract, peptone, pectin, dextrin, starch, cellulose, xylan, glucose, fructose, galactose, mannose, arabinose, rhamnose, xylose, ribose, trehalose, cellobiose, sucrose, lactose, maltose, raffinose, sorbitol, mannitol, acetate, lactate, pyruvate, malate, propionate, butyrate, fumarate, succinate, citrate, ethanol, propanol, glycerol, methanol, 2-methoxyphenol, 3,4-dimethoxybenzoate, or 2-methoxybenzoate. Nitrate, sulfate, perchlorate, fumarate, Fe (III) citrate, nitrite, sulfite, or O_2_ are not used as the electron acceptors. Sulfide formation is observed on the medium with thiosulfate or elemental sulfur, but acetate is the main product of the growth. In the presence of thiosulfate or elemental sulfur, growth rate and cell yield are the same as in the absence of these compounds. The G+C content of genomic DNA is 33 mol %. The major cellular fatty acid is iso-C_15:0_ and C_16:0_. Minor components are iso-C_17:0_, C_18:0_, iso-C_16:0_, and anteiso-C_17:0_. Membranes polar lipids include four unidentified phospholipids and unknown aminophospholipid. No respiratory quinones were detected. The type strain is 3443-3Ac^T^ (=DSM 108286^T^ = VKM B-3415^T^), isolated from a sample of sediments collected from the terrestrial hot spring Kaskadny at East Thermal Field, Uzon Caldera, Kamchatka, Russia (N54° 30.026′ E160° 00.374′, elevation 658 m).

## Data availability statement

Strain 3443-3Ac^T^ was deposited in the DSMZ (German Collection of Microorganisms and Cell Cultures) and VKM (All-Russian Collection of Microorganisms) under accession numbers: DSM 108286 and VKM B-3415, respectively. Genome sequence, as well as related project information and sample details, were deposited in NCBI database under accession numbers: CP060096, PRJNA647162, and SAMN15577649, respectively. The genome sequence was also deposited in IMG with genome ID 2860381621.

## Author contributions

EF, AL, and IK: conceptualization. EF, AG, ST, and AE: data curation. IK: funding acquisition. EF, AL, AG, ST, AN, and AE: investigation. EF and IK: project administration and supervision. EF, AG, ST, and AN: resources. EF, AL, ST, AE, and IK: visualization. EF and AL: writing – original draft preparation. All authors contributed to the formal analysis, methodology, validation, and writing – review and editing and article and approved the submitted version.

## Footnotes

1
https://onlinelibrary.wiley.com/doi/book/10.1002/9781118960608

2
https://data.gtdb.ecogenomic.org/
