# Supplementary material for: Obligate autotrophy at the thermodynamic limit of life in a new acetogenic bacterium
Source: Front Microbiol. 2023 May 12;14:1185739. doi: 10.3389/fmicb.2023.1185739 (PMC10213532; doi:10.3389/fmicb.2023.1185739)
Supplement: Supplementary file 1 [file Data_Sheet_1.DOCX]

Supplementary Material 1

Obligate autotrophy at the thermodynamic limit of life in a new acetogenic bacterium

Evgenii N. Frolov*, Alexander G. Elcheninov, Alexandra V. Gololobova, Stepan V. Toshchakov, Andrei A. Novikov, Alexander V. Lebedinsky, Ilya V. Kublanov

*** Correspondence:** Evgenii N. Frolov: evgenii_frolov_89@mail.ru

**Table S1**. Consumption of glucose and cellobiose by cell extract of strain 3443-3Ac^T^.

|  | **Sugar* consumption, mM** | | | |
| --- | --- | --- | --- | --- |
|  | **Replicate 1** | **Replicate 2** | **Replicate 3** | **Mean** |
| Control: Buffer + Glucose | 0,022 | 0,160 | 0,110 | 0,097 |
| Control: Buffer + Cellobiose | 0,090 | 0,060 | 0,060 | 0,070 |
| Sample: Cell extract + Glucose | 0,045 | 0,081 | 0,120 | 0,082 |
| Sample: Cell extract + Cellobiose | 0,058 | 0,116 | 0,098 | 0,091 |
| Control: Cell extract | 0,072 | 0,069 | 0,074 | 0,071 |

* glucose or cellobiose, depending on the experiment

The initial sugar concentration was 1 mM. Incubation time, 9 hours. Reducing sugars were measured using DNSA method. Glucose and cellobiose concentrations were calculated using glucose or cellobiose calibration curves, respectively. Sugar consumption was calculated from the difference in sugar concentration before and after incubation.

**Table S2.** *A. autotrophica* strain 3443-3Ac^T^ genes encoding enzymes of the Embden-Meyerhof-Parnas pathway.

| **Locus tags** | **Gene name** | **Predicted function** | **Best BlastP hit*** | **% identity** | **Score** | **E-value** | **COGs and Pfam domains** |
| --- | --- | --- | --- | --- | --- | --- | --- |
| ACETAC_09365 | *glcK* | Hexokinase | *Thermoanaerobacterium* sp. RBIITD | 68.35 | 424 | 4e-146 | COG1940  pfam00480 |
| ACETAC_04395 | *pgi* | Glucose-6-phosphate isomerase | *Thermoanaerobacterium* sp. RBIITD | 80.71 | 772 | 0.0 | COG0166  pfam00342 |
| ACETAC_04665 | *pfkA* | 6-phosphofructokinase | *Thermoanaerobacter kivui* | 84.42 | 560 | 0.0 | COG0205  pfam00365 |
| ACETAC_09275 | *fba* | Aldolase | *Thermoanaerobacterium* sp. RBIITD | 86.88 | 506 | 6e-180 | COG0191  pfam01116 |
| ACETAC_02625 | *tpiA* | Triose-phosphate isomerase | *Thermoanaerobacterium* sp. RBIITD | 80.24 | 389 | 6e-135 | COG0149  pfam00121 |
| ACETAC_02615 | *gap1* | Glyceraldehyde-3-phosphate dehydrogenase | *Thermoanaerobacterium* sp. RBIITD | 87.76 | 614 | 0.0 | COG0057  pfam00044 pfam02800 |
| ACETAC_04405 | *gap2* | NADP-dependent glyceraldehyde-3-phosphate dehydrogenase | *Planctomycetacea*e bacterium | 70.43 | 828 | 0.0 | COG1012  pfam00171 |
| ACETAC_02620 | *pgk* | Phosphoglycerate kinase | *Thermoanaerobacterium* sp. RBIITD | 85.24 | 689 | 0.0 | COG0126  pfam00162 |
| ACETAC_02630 | *gpmI* | Phosphoglycerate matase | *Thermoanaerobacterium* sp. RBIITD | 83.53 | 890 | 0.0 | COG0696  pfam01676 pfam06415 |
| ACETAC_02635 | *eno* | Enolase | *Thermoanaerobacterium* sp. RBIITD | 89.49 | 792 | 0.0 | COG0148  pfam00113 pfam03952 |
| ACETAC_04670 | *pyk* | Piruvate kinase | *Thermoanaerobacterium* sp. RBIITD | 82.16 | 979 | 0.0 | COG0469 pfam00224 pfam00391 pfam02887 |

**Table S3.**  Comparative composition of CFA in strain 3443-3Ac^T^ and its closest phylogenetic relatives from the order *Thermoanaerobacterales*: 1, *Thermoanaerobacterium thermosaccharolyticum* (Yamamoto et al., 1998); 2, *Thermoanaerobacterium thermosulfurigenes* (Yamamoto et al., 1998); 3, *Thermoanaerobacterium butyriciformans* (Lopez et al., 2017); 4, *Thermoanaerobacterium thermostercoris* (Lopez et al., 2017); 5, *Thermoanaerobacterium aotearoense* (Lopez et al., 2017); 6, *Thermoanaerobacter thermohydrosulfuricus* (Yamamoto et al., 1998); 7, *Thermoanaerobacter thermocopriae* (Yamamoto et al., 1998); 8, *Thermoanaerobacter uzonensis* (Wagner et al., 2008); 8, *Thermoanaerobacter sulfurigignens* (Wagner et al., 2008); 10, *Thermoanaerobacter pentosaceus* (Tomas et al., 2013); 11, *Caldanaerobacter subterraneus* subsp. *yonseiensis* (Kim et al., 2001). Compounds above 5% are in bold. tr - trace amounts.

| **Fatty acid** | **3443-3Ac**  **(current study)** | **1** | **2** | **3** | **4** | **5** | **6** | **7** | **8** | **9** | **10** | **11** |
| --- | --- | --- | --- | --- | --- | --- | --- | --- | --- | --- | --- | --- |
| **10:0** | - | - | - | 0.10 | - | - | - | - | - | - | - | - |
| **11:0 iso** | - | - | - | 0.11 | - | 0.96 | - | - | - | - | 0.59 | - |
| **12:0** | - | - | - | 1.76 | - | - | - | - | - | - | - | - |
| **13:0 iso** | - | - | tr | 0.32 | - | 0.66 | - | tr | 1.40 | 0.90 | 1.38 | - |
| **13:0 anteiso** | - | - | - | 0.13 | - | - | - | - | - | - | - | - |
| **14:0** | - | **32.50** | tr | **24.35** | - | 1.94 | 0.50 | tr | 1.70 | - | - | - |
| **14:0 iso** |  | - | tr | - | - | - | - | - | - | - | - | - |
| **14:0 iso3OH** | - | - | - | 1.40 | **8.60** | **11.34** | - | - | - | - | **15.79** | **6.00** |
| **14:0 2OH** | - | - | - | - | - | 1.19 | - | - | - | - | 1.35 | - |
| **15:0** | - | - | 4.2 | - | - | - | **7.6** | **5.0** | **11.80** | **18.30** | - | - |
| **15:0 iso** | **52.80** | **21.6** | **43.4** | **32.59** | **49.93** | **49.77** | **47.3** | **54.1** | **53.50** | **57.70** | **40.39** | **42.00** |
| **15:0 anteiso** | - | - | - | **5.39** | **6.75** | **9.19** | - | - | **5.30** | **6.10** | **5.94** | **5.00** |
| **15:0 iso 3OH** | - | - | - | - | - | 1.21 | - | - | - | - | - | - |
| **16:0** | **40.40** | **25.50** | **12.70** | **14.56** | **20.09** | **9.16** | **11.2** | 1.1 | **7.30** | 2.20 | - | **7.00** |
| **16:0 iso** | 0.80 | - | 4.60 | 0.10 | - | - | 3.1 | 1.6 | - | - | **7.42** | - |
| **16:0 N alcohol** | - | - | - | 2.45 | - | - | - | - | - | - | - | - |
| **16:0 10-methyl** | - | - | - | - | - | - | - | - | **7.3** | **5.5** | - | - |
| **17:0** | - | tr | 3.60 | - | - | - | - | **10.3** | - | - | - | - |
| **17:0 iso** | 4.80 | **16.70** | **23.30** | **10.46** | **9.86** | **9.62** | **23.3** | **24.7** | 2.8 | **5.7** | **15.51** | **11.00** |
| **17:0 anteiso** | 0.40 | - | - | 4.50 | 3.49 | 3.44 | - | - | - | - | 4.25 | **5.00** |
| **18:0** | 0.90 | 1.50 | 1.20 | 0.45 | - | 0.83 | 1.2 | 1.8 | **5.0** | - | - | - |
| **18:0 iso** | - | 1.30 | 4.00 | - | - | - | 2.1 | 0.5 | - | - | - | - |
| **16:1** | - | - | - | - | - | - | - | - | - | - | - | - |
| **18:1** | - | tr | 2.6 | - | - | - | 3.7 | - | 3.9 | 3.6 | - | **6.00** |

**Table S4.** Cas-operons detected in the genome of *A. autotrophica* strain 3443-3Ac^T^ by *cctyper* web server.

| **#** | **Start** | **End** | **Prediction** | **Complete_Interference** | **Complete_Adaptation** | **Best_type** | **Best_score** | **Genes** |
| --- | --- | --- | --- | --- | --- | --- | --- | --- |
| 1 | 233848 | 256737 | I-A | 0% | 100% | I-A | 7 | Csx1, Csx2, Csx1, Cas4_5, Cas1, Cas2 |
| 2 | 148672 | 156457 | I-B | 100% | 100% | I-B | 22 | Cas8b2, Cas7, Cas5, Cas3,Cas4, Cas1, Cas2, Cas6 |
| 3 | 308782 | 318243 | III-B | 100% | NA | III-B | 16 | Csx1,Cmr1,Cas10,Cmr3, Csm6, Cmr4, Cmr5, Cmr6 |
| 4 | 200224 | 210348 | I-B | 100% | 0% | I-B | 14 | Cas6,Cas8b1,Cas7b, TnsB,Cas5,Cas3 |
| 5 | 877968 | 886771 | III-D | 50% | NA | III-D | 10 | Csx1,Cas10,Csm3,Csm3, Csx19,Csm3 |

**Table S5.** *A. autotrophica* strain 3443-3Ac^T^ genes encoding enzymes of the Wood-Ljungdahl pathway, electron-bifurcating hydrogenase, energy-converting hydrogenase (Ech complex) and ATP synthase.

| **Locus tags** | **Gene name** | **Predicted function** | **Best BlastP hit*** | **% identity** | **Score** | **E-value** | **COGs and Pfam domains** |
| --- | --- | --- | --- | --- | --- | --- | --- |
| **The oxidative part of acetogenesis** | | | | | | | |
| ACETAC_10975 | *hydA1* | Electron-bifurcating hydrogenase (HydABC) | *Thermoanaerobacter kivui* | 79.33 | 943 | 0.0 | COG4624  pfam00037  pfam02256  pfam02906  pfam10588  pfam12837  pfam13510 |
| ACETAC_10970 | *hydB* |  | *Thermoanaerobacter kivui* | 83.68 | 1080 | 0.0 | COG1894  pfam01257  pfam01512  pfam10531  pfam10589  pfam12838 |
| ACETAC_10965 | *hydC* |  | *Thermoanaerobacter kivui* | 79.41 | 282 | 5e-95 | COG1905  pfam01257 |
| **The reductive part of acetogenesis (Wood-Ljungdahl pathway)** | | | | | | | |
| ACETAC_00285 | *hycB1* | Hydrogen-dependent CO_2_ reductase  (HDCR) | *Thermoanaerobacter kivui* | 78.01 | 323 | 9e-111 | COG1142  pfam12798  pfam12800  pfam13247 |
| ACETAC_00290 | *hydA2* |  | *Thermoanaerobacter kivui* | 82.39 | 816 | 0.0 | COG4624  pfam00037  pfam02256  pfam02906 |
| ACETAC_10625 | *fdhD* |  | *Thermoanaerobacter kivui* | 62.59 | 352 | 8e-120 | COG1526  pfam02634 |
| ACETAC_00565 | *fdhF1* |  | *Thermoanaerobacter kivui* | 77.32 | 1213 | 0.0 | COG3383  pfam00384  pfam01568  pfam04879 |
| ACETAC_08370/08380 | *fdhF2* |  | *Clostridium magnum* | 81.02 | 1234 | 0.0 | COG3383  pfam00384  pfam01568  pfam04879 |
| ACETAC_10785 | *fdhF3* |  | *Thermoanaerosceptrum fracticalcis* | 76.85 | 1194 | 0.0 | COG3383  pfam00384  pfam01568  pfam04879 |
| ACETAC_10790 | *hycB2* |  | *Thermoanaerobacter kivui* | 72.89 | 244 | 9e-180 | COG1142  pfam12798  pfam13247 |
| ACETAC_10585 | *fhs* | Formyl-THF synthetase | *Thermoanaerobacter kivui* | 82.65 | 926 | 0.0 | COG2759  pfam01268 |
| ACETAC_10580 | *fchA* | Methenyl-THF cyclohydrolase | *Haloimpatiens lingqiaonensis* | 78.57 | 299 | 2e-100 | COG3404  pfam04961 |
| ACETAC_10575 | *folD* | Methylene-THF dehydrogenase | *Clostridium carboxidivorans* | 81.34 | 479 | 3e-169 | COG0190  pfam00763  pfam02882 |
| ACETAC_10570 | *metV* | Methylene-THF reductase | *Clostridium scatologenes* | 75.46 | 347 | 5e-119 | COG-  pfam12225 |
| ACETAC_10565 | *metF* |  | *Clostridium ragsdalei* | 83.96 | 506 | 8e-180 | COG0685  pfam02219 |
| ACETAC_10540 | *acsE* | Methyl transferase | *Clostridium scatologenes* | 85.06 | 464 | 3e-164 | COG1410  pfam00809 |
| ACETAC_10550 | *acsD* | Corrinoid iron sulfur protein | *Clostridium magnum* | 75.96 | 499 | 2e-176 | COG2069  pfam03599 |
| ACETAC_10545 | *acsC* |  | *Clostridium magnum* | 75.95 | 723 | 0.0 | COG1456  pfam03599  pfam04060 |
| ACETAC_10535 | *acsB* | Acetyl-CoA synthase | *Clostridium luticellarii* | 80.93 | 1184 | 0.0 | COG1614  pfam03598 |
| ACETAC_10650 | *acsA* | CO dehydrogenase | *Thermoanaerobacter kivui* | 81.05 | 1067 | 0.0 | COG1151  pfam03063 |
| ACETAC_02795 | *cooS* |  | *Thermoanaerobacter kivui* | 95.69 | 1207 | 0.0 | COG1151  pfam03063 |
| ACETAC_06980 | *pta* | Phosphotransacetylase | *Thermoanaerobacter* sp. | 80.07 | 452 | 1e-157 | COG0280  pfam01515 |
| ACETAC_06975 | *ackA* | Acetate kinase | *Thermoanaerobacterium* sp. RBIITD | 78.95 | 639 | 0.0 | COG0282  pfam00871 |
| **Energy-conserving** **reactions of acetogenesis** | | | | | | | |
| ACETAC_10720 | *echA* | Energy-converting hydrogenase  (Ech complex) | *Thermoanaerobacter kivui* | 72.26 | 1108 | 0.0 | COG0651  pfam00361 |
| ACETAC_10715 | *echB* |  | *Thermoanaerobacter kivui* | 81.39 | 525 | 0.0 | COG0650  pfam00146 |
| ACETAC_10710 | *echC* |  | *Thermoanaerobacter kivui* | 92.41 | 273 | 3e-92 | COG3260  pfam01058 |
| ACETAC_10705 | *echF* |  | *Thermoanaerobacter kivui* | 80.35 | 299 | 1e-101 | COG1143  pfam13187  pfam13237 |
| ACETAC_10700 | *hycB3* |  | *Thermoanaerobacter kivui* | 85.89 | 296 | 1e-100 | COG0437  pfam12837  pfam13247 |
| ACETAC_10695 | - |  | *Thermoanaerobacter kivui* | 69.93 | 227 | 9e-74 | COG-  pfam- |
| ACETAC_10690 | - |  | *Thermoanaerobacter kivui* | 83.83 | 296 | 9e-101 | COG-  pfam- |
| ACETAC_10685 | *echD* |  | *Thermoanaerobacter kivui* | 70.62 | 248 | 2e-81 | COG-  pfam00329 |
| ACETAC_10680 | *echE* |  | *Thermoanaerobacter kivui* | 76.67 | 601 | 0.0 | COG3261  pfam00346  pfam00374 |
| ACETAC_10735 | *atpC* | ATP synthase  epsilon chain | *Thermoanaerobacterium xylanolyticum* | 61.48 | 167 | 2e-50 | COG0355  pfam00401  pfam02823 |
| ACETAC_10740 | *atpD* | ATP synthase  beta chain | *Thermoanaerobacterium* sp. RBIITD | 88.29 | 853 | 0.0 | COG0055  pfam00006  pfam02874 |
| ACETAC_10745 | *atpG* | ATP synthase  gamma chain | *Thermoanaerobacterium aotearoense* | 71.34 | 427 | 2e-148 | COG0224  pfam00231 |
| ACETAC_10750 | *atpA* | ATP synthase  alpha chain | *Thermoanaerobacterium* sp. RBIITD | 81.15 | 858 | 0.0 | COG0056  pfam00006  pfam00306  pfam02874 |
| ACETAC_10755 | *atpH* | ATP synthase delta chain | *Thermoanaerobacterium* sp. RBIITD | 56.18 | 184 | 3e-56 | COG0712  pfam00213 |
| ACETAC_10760 | *atpF* | ATP synthase F0 sector subunit b | *Thermoanaerobacterium* sp. RBIITD | 74.23 | 237 | 2e-77 | COG0711  pfam00430 |
| ACETAC_10765 | *atpE* | ATP synthase F0 sector subunit c | *Thermoanaerobacterium thermosaccharolyticum* | 89.13 | 84 | 2e-19 | COG0636  pfam00137 |
| ACETAC_10770 | *atpB* | ATP synthase F0 sector subunit a | *Thermoanaerobacterium* sp. RBIITD | 71.89 | 286 | 9e-95 | COG0356  pfam00119 |


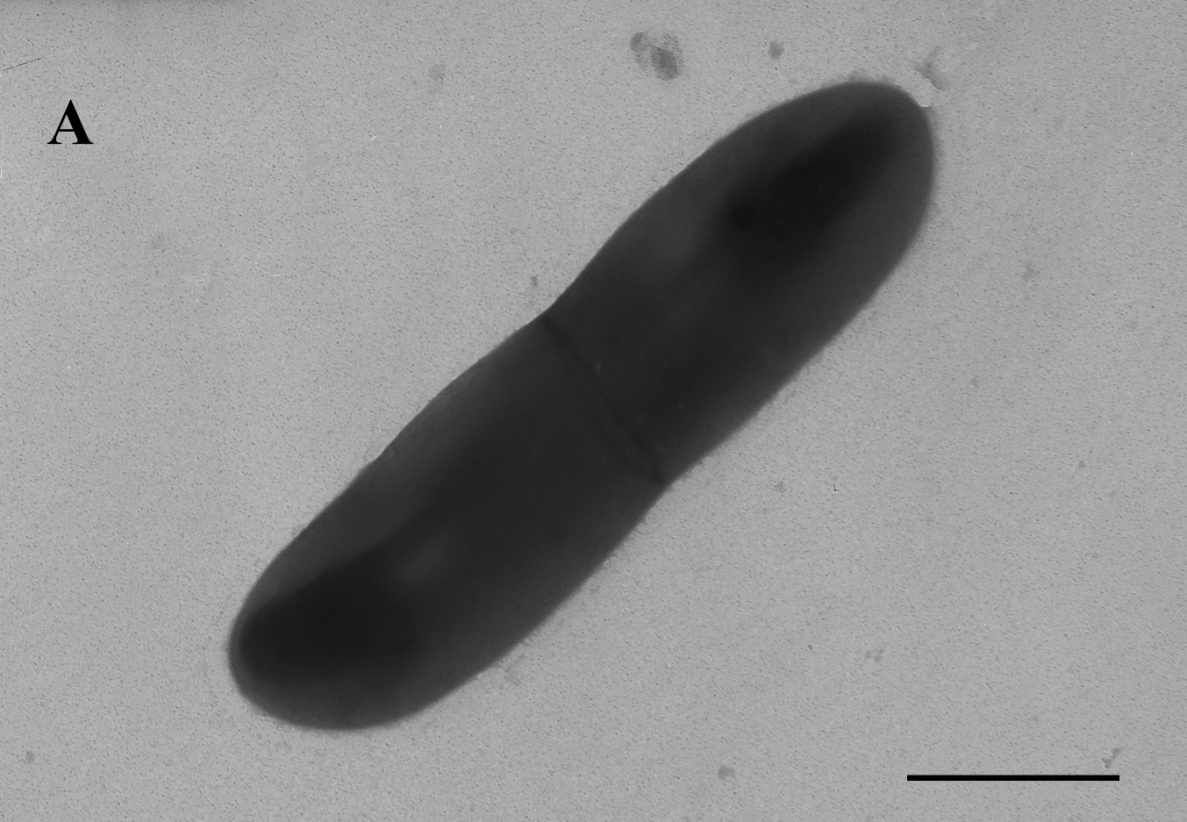

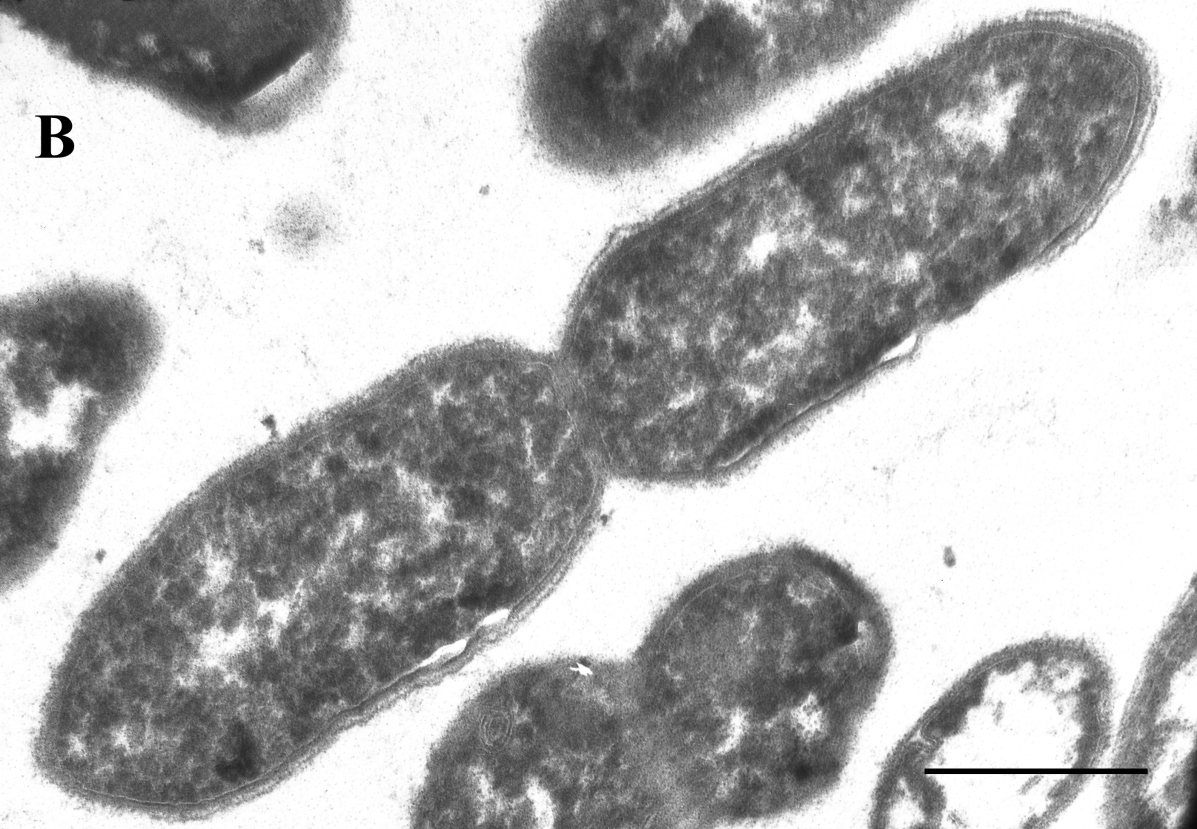


**Figure S1.** Cell morphology and ultrastructure of *A. autotrophica* strain 3443-3Ac^T^. (A) Electron micrograph showing overall cell morphology; bar, 0.5 µm. (B) Ultrathin section showing cell wall structure; bar, 0.5 µm.


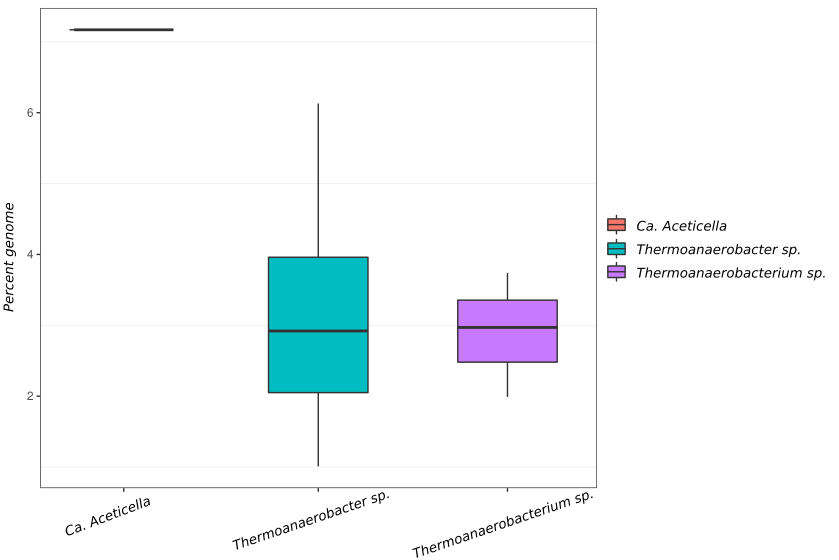


**Figure S2.** Boxplot of the proportions of the *A. autotrophica* strain 3443-3a^T^ genome, occupied by mobile genetic elements, predicted by isescan package (Xie and Tang, 2017).

**
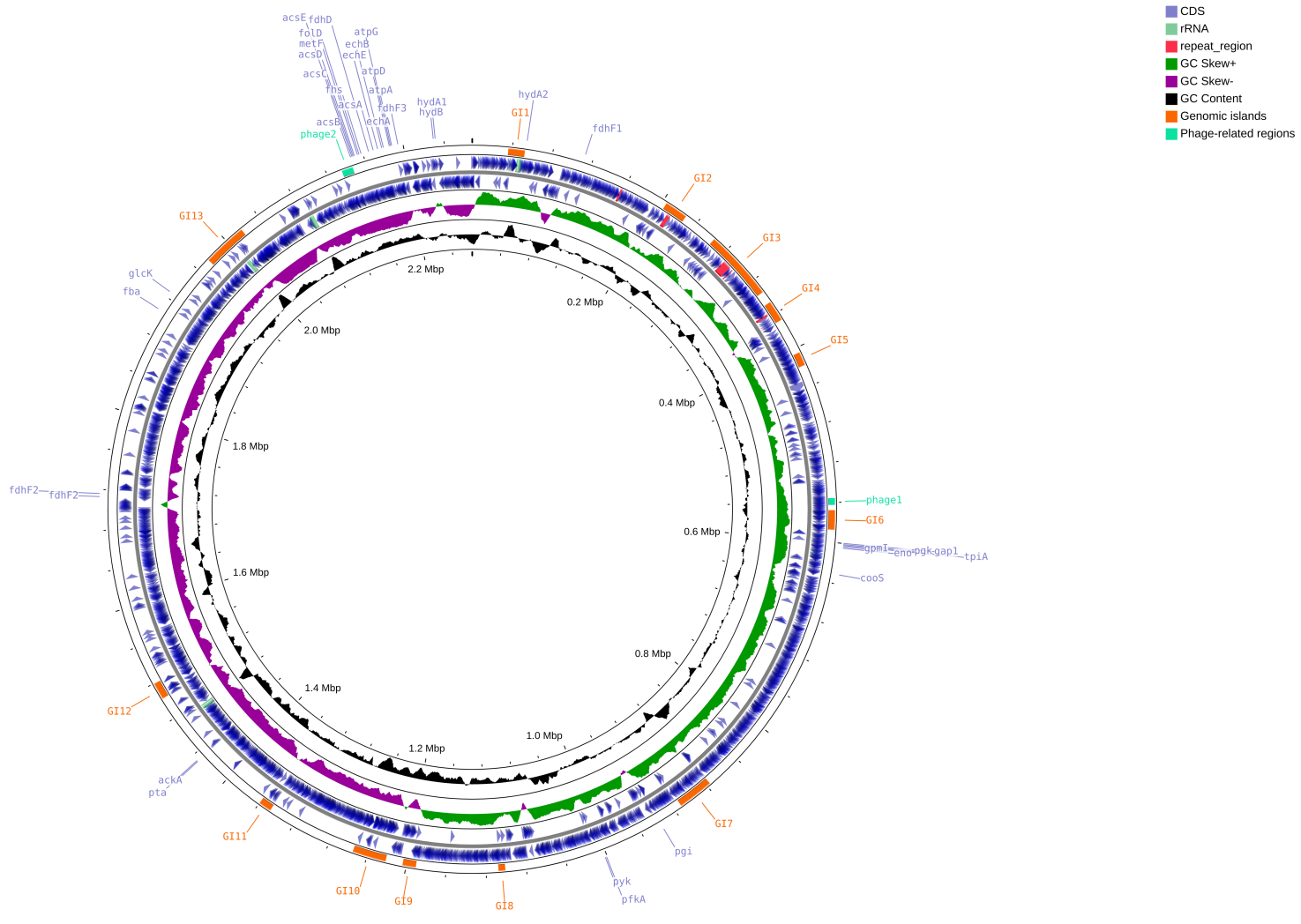
**

**Figure S3.** Circular map of the chromosome of *A. autotrophica* strain 3443-3a^T^. From inner to outer ring: (1) genomic coordinates; (2) GC-content; (3) GC-skew; (4) CDSs of lagging strand; (4) CDSs on leading strand; (5) Genomic islands and phage-related regions. Positions of key metabolic genes mentioned in the paper are marked by text labels.


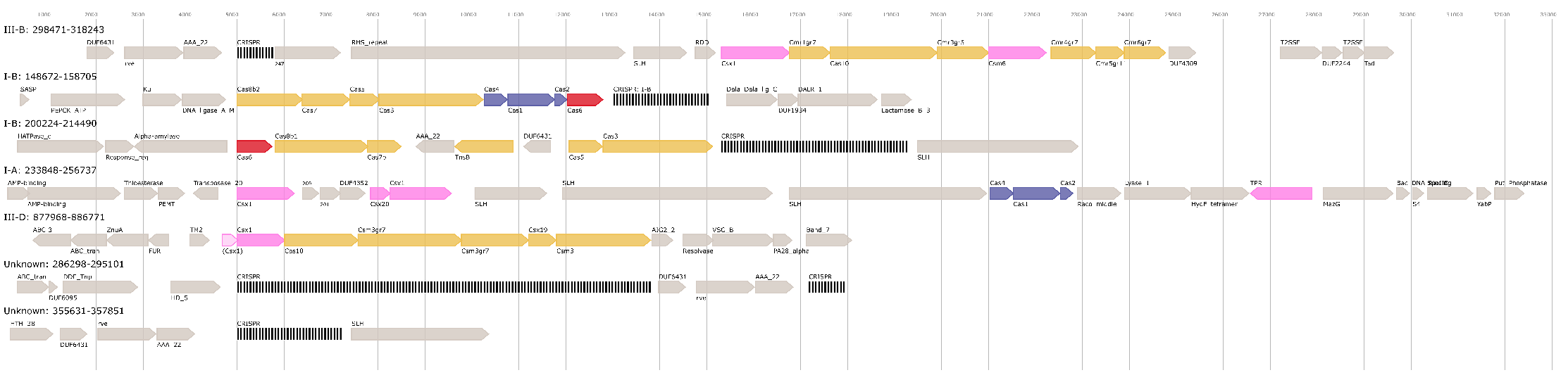


**Figure S4.** Schematic representation of CRISPR-Cas loci detected by *cctyper* web server in the genome of *A. autotrophica*. CRISPR repeats are shown as black bars.

**
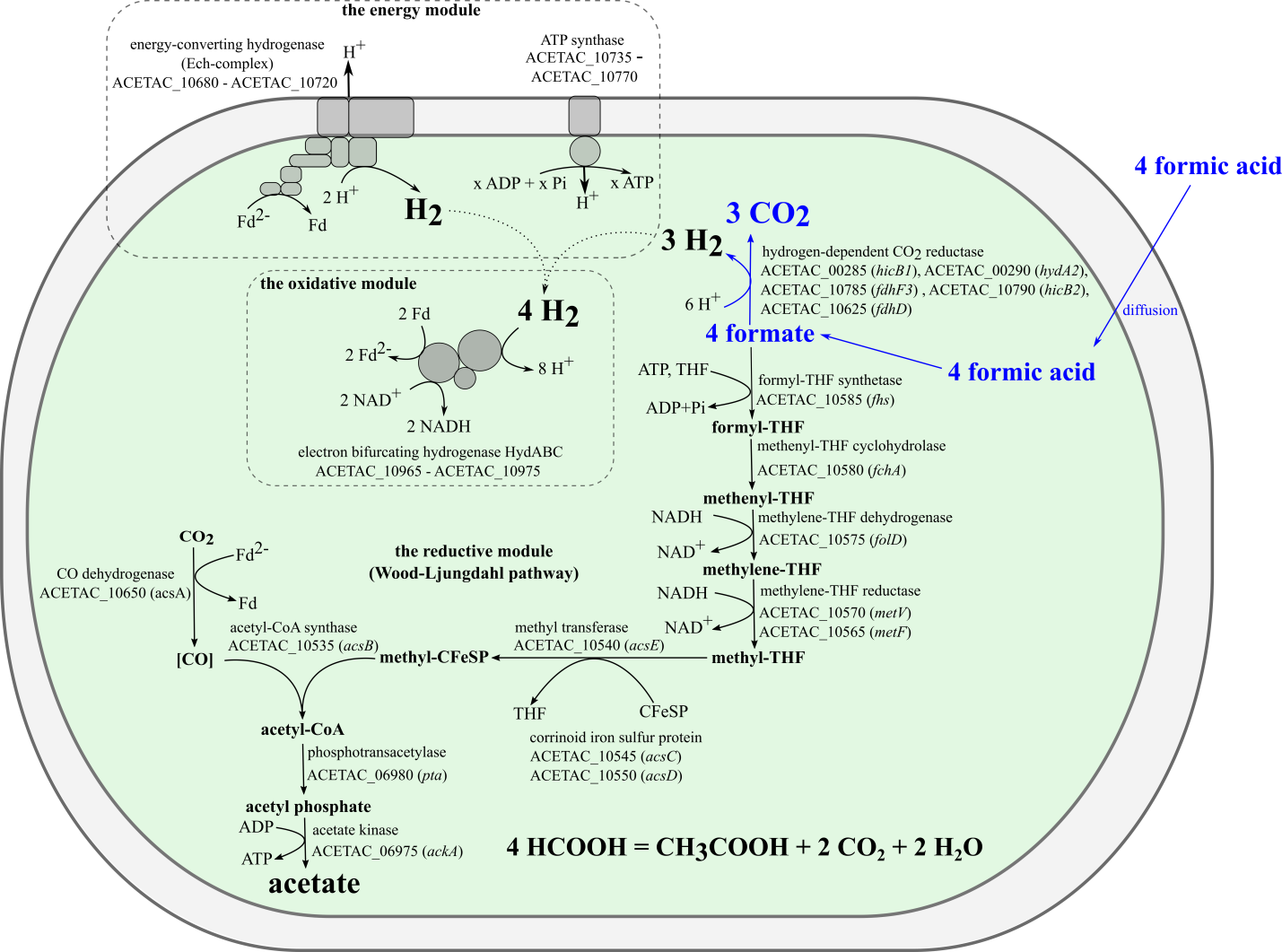
**

**Figure S5.** Acetogenesis on formate in *A. autotrophica* strain 3443-3Ac^T^.


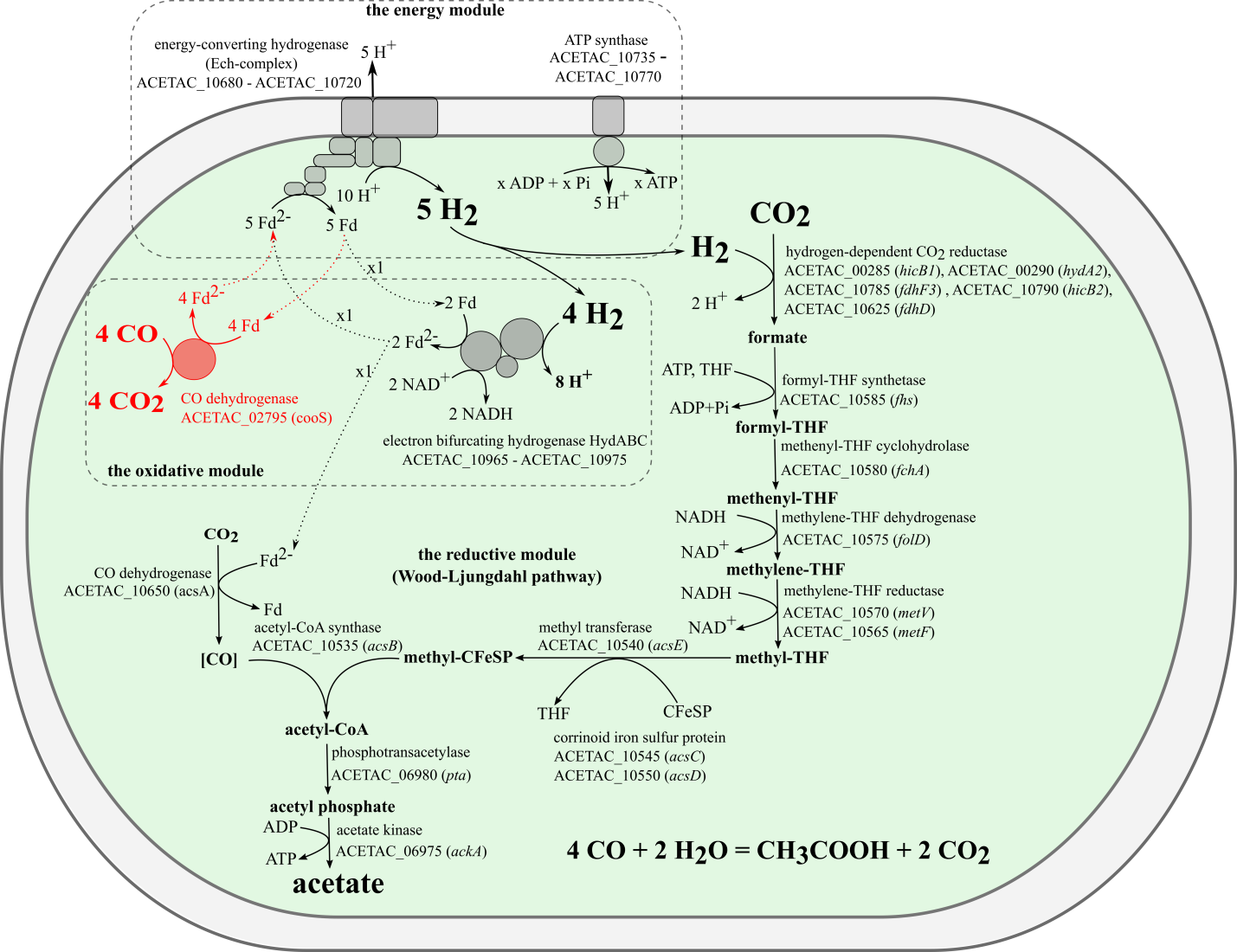


**Figure S6.** Acetogenesis on СO in *A. autotrophica* strain 3443-3Ac^T^.

**
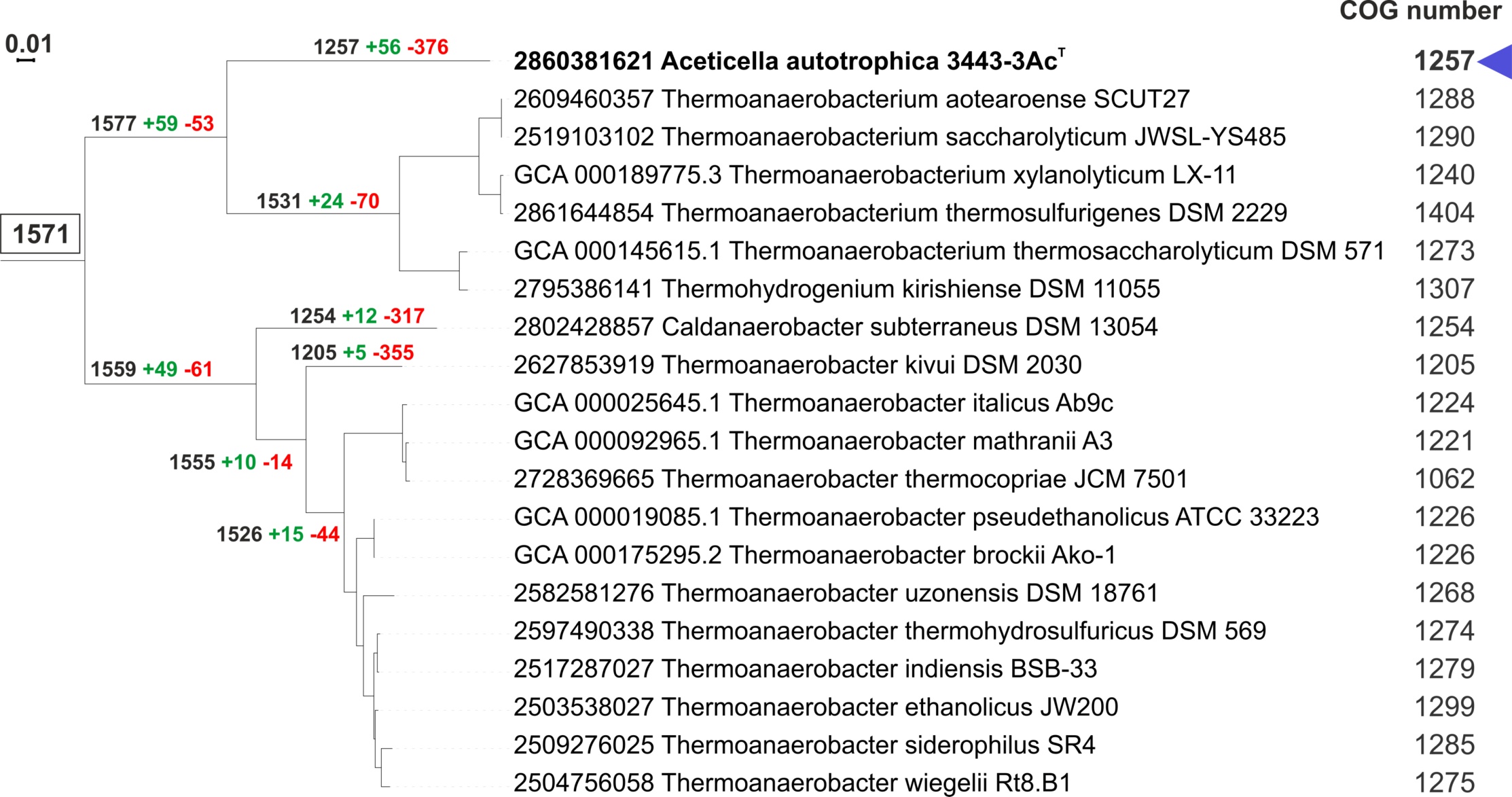
**

**Figure S7.** Analysis of total gene gains and gene losses in genome of *A. autotrophica* strain 3443-3Ac^T^ (in bold) and other members of TTC group.

**Figure S8.** A subtree of acetyl-CoA synthase AscB ML tree (the entire tree is shown in Supplementary Material 5). The entire tree was constructed for all 1654 AscBs (acetyl-CoA synthases of bacterial type) found in representative genomes of GTDB rs202 (47,894 genomes). The subtree represents a clade (having a bootstrap support of 99%) which is dominated by AcsBs from representatives of p_Firmicutes_A c_Clostridia and includes virtually all AcsBs from this GTDB taxon (with a few exceptions apparently due to HGT events). In the subtree, the effects of HGT events can also be noted when comparing the tree topology with the GTDB phylogeny-grounded taxonomy.

**
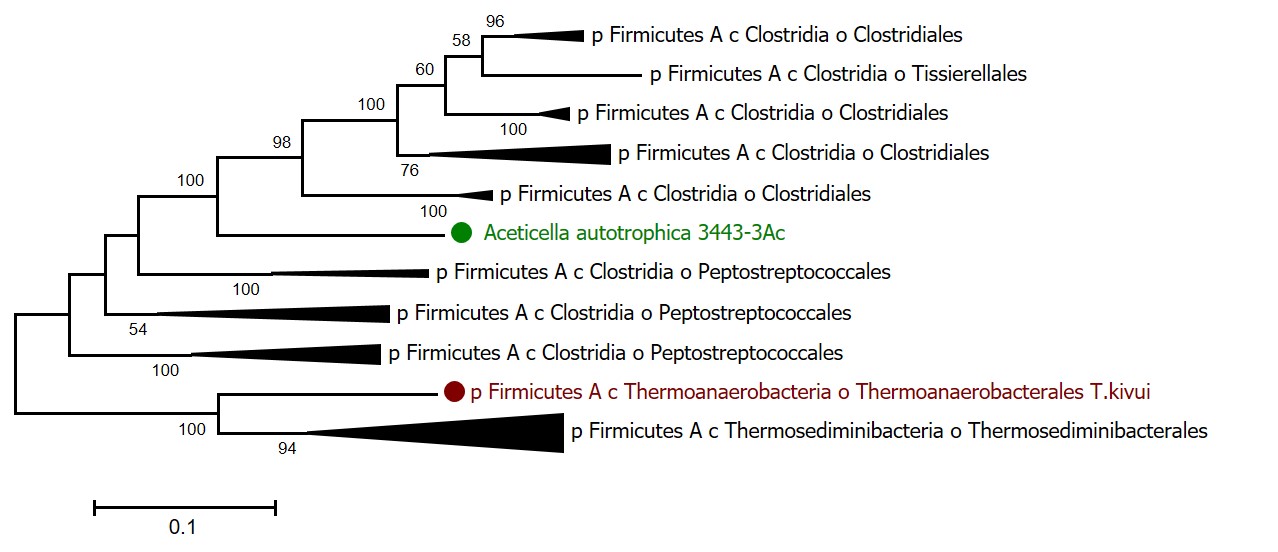
**

**Figure S9.** AcsB, acetyl-CoA synthase. 25 best hits of *A. autotrophica*’s protein and 25 best hits of *T. kivui*’s protein were taken after blast in representative genomes of GTDB rs202. Redundant proteins were discarded with CD-HIT at 100% cut-off value. The ML tree with 100 bootstrap replicates was constructed with MEGA-6 after alignment with built-in ClustalW. Bootstrap values lower than 50 are not shown.


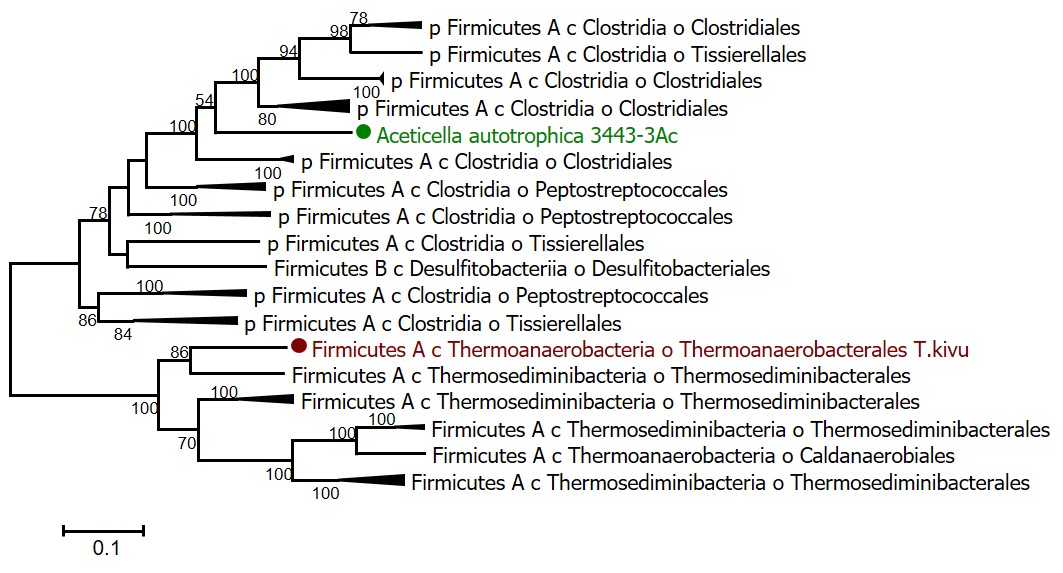


**Figure S10.** AcsC, corrinoid iron sulfur protein large subunit. 25 best hits of *A. autotrophica*’s protein and 25 best hits of *T. kivui*’s protein were taken after blast in representative genomes of GTDB rs202. Redundant proteins were discarded with CD-HIT at 100% cut-off value. The ML tree with 100 bootstrap replicates was constructed with MEGA-6 after alignment with built-in ClustalW. Bootstrap values lower than 50 are not shown.


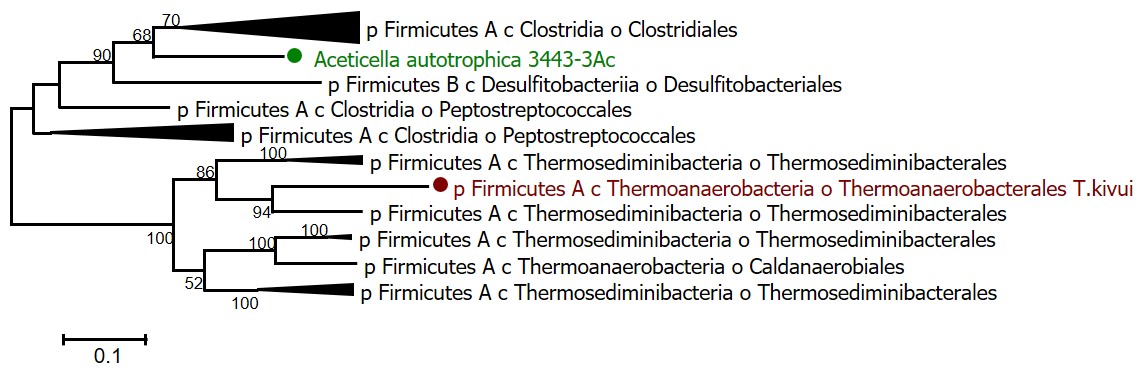


**Figure S11.** AcsD, corrinoid iron sulfur protein small subunit.25 best hits of *A. autotrophica*’s protein and 25 best hits of *T. kivui*’s protein were taken after blast in representative genomes of GTDB rs202. Redundant proteins were discarded with CD-HIT at 100% cut-off value. The ML tree with 100 bootstrap replicates was constructed with MEGA-6 after alignment with built-in ClustalW. Bootstrap values lower than 50 are not shown.


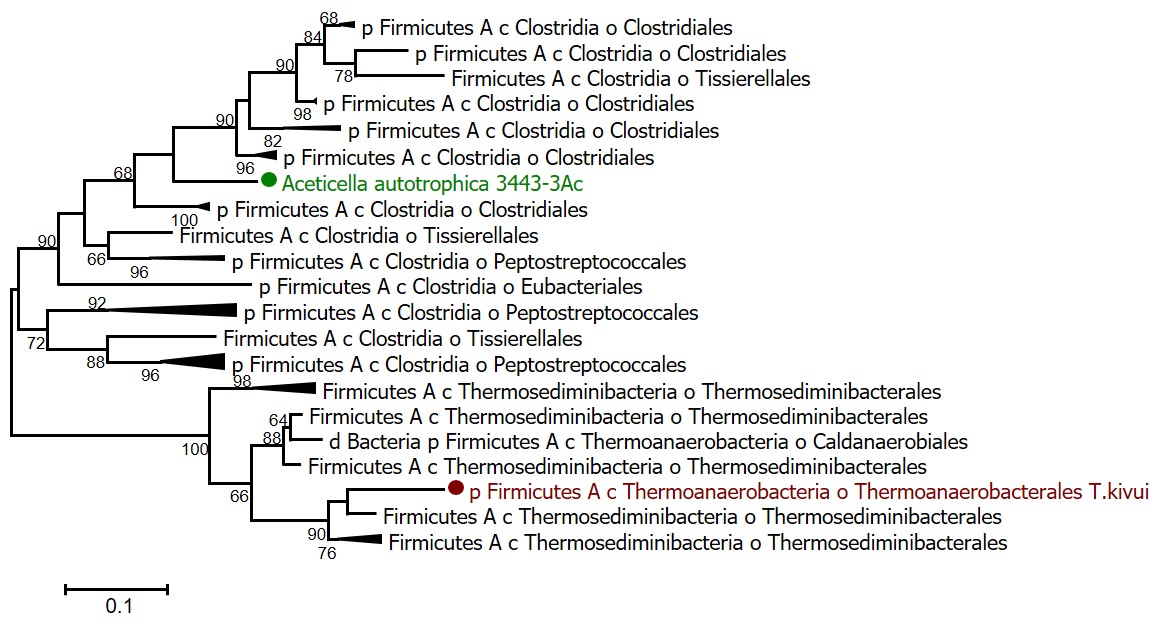


**Figure S12.** AcsE, methyl transferase. 25 best hits of *A. autotrophica*’s protein and 25 best hits of *T. kivui*’s protein were taken after blast in representative genomes of GTDB rs202. Redundant proteins were discarded with CD-HIT at 100% cut-off value. The ML tree with 100 bootstrap replicates was constructed with MEGA-6 after alignment with built-in ClustalW. Bootstrap values lower than 50 are not shown.


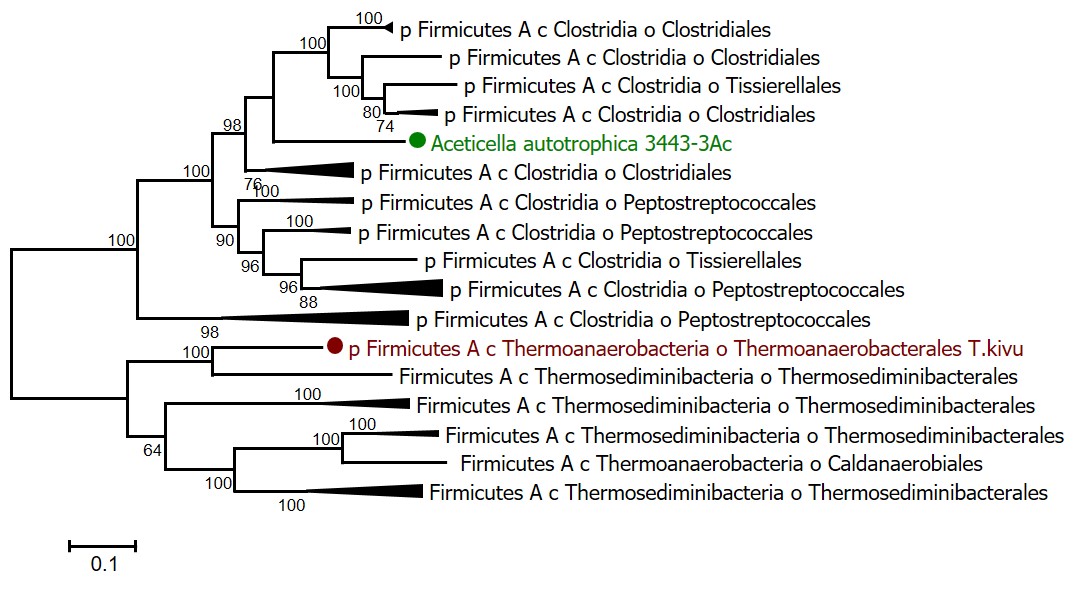


**Figure S13.** AcsV, acetyl-CoA synthase corrinoid activation protein. 25 best hits of *A. autotrophica*’s protein and 25 best hits of *T. kivui*’s protein were taken after blast in representative genomes of GTDB rs202. Redundant proteins were discarded with CD-HIT at 100% cut-off value. The ML tree with 100 bootstrap replicates was constructed with MEGA-6 after alignment with built-in ClustalW. Bootstrap values lower than 50 are not shown.

**
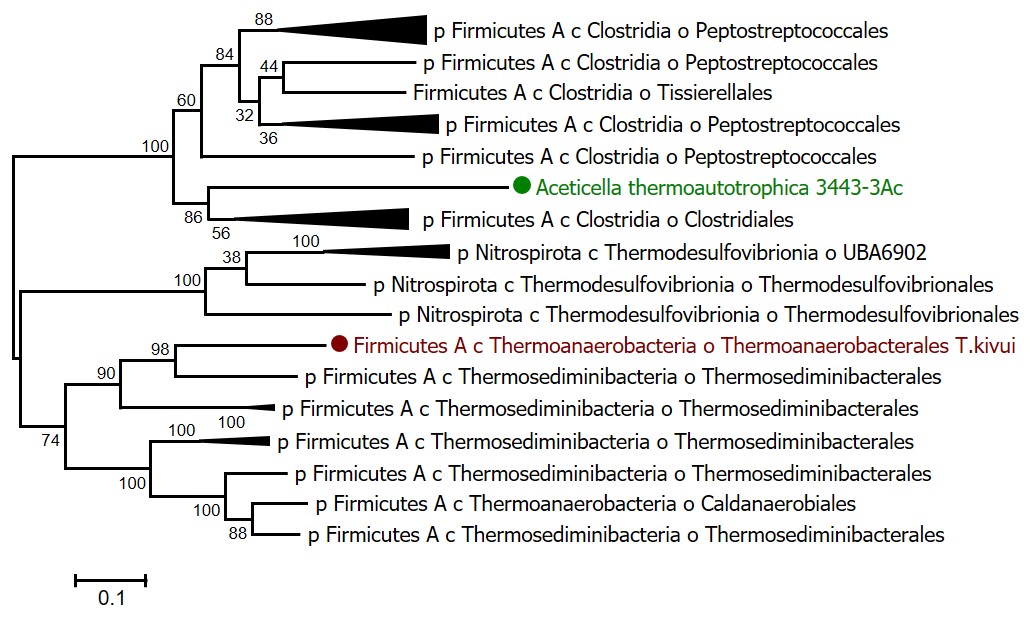
**

**Figure S14.** PdhD, dihydrolipoamide dehydrogenase. 25 best hits of *A. autotrophica*’s protein and 25 best hits of *T. kivui*’s protein were taken after blast in representative genomes of GTDB rs202. Redundant proteins were discarded with CD-HIT at 100% cut-off value. The ML tree with 100 bootstrap replicates was constructed with MEGA-6 after alignment with built-in ClustalW. Bootstrap values lower than 50 are not shown.

**
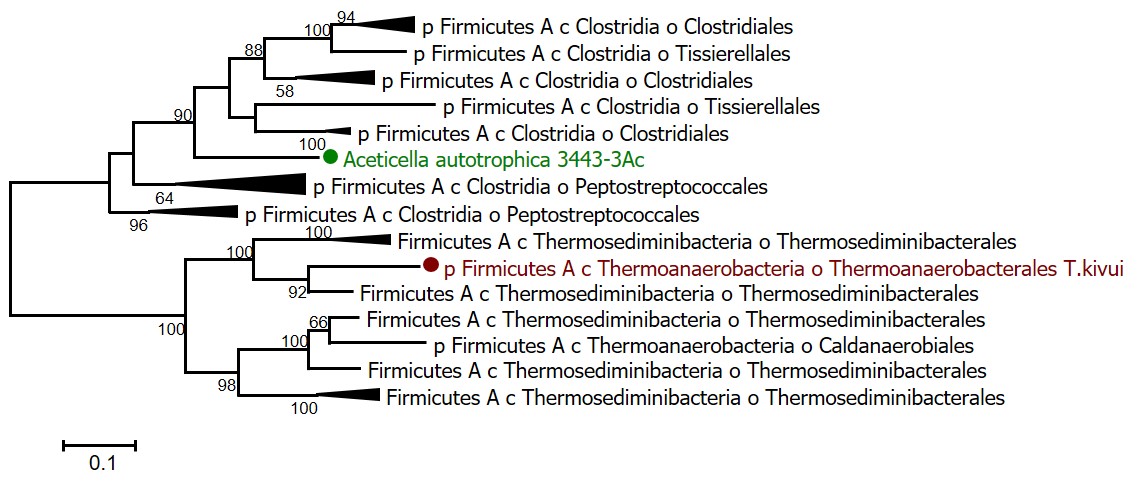
**

**Figure S15.** CooC2, maturation factor of the CO dehydrogenase. 25 best hits of *A. autotrophica*’s protein and 25 best hits of *T. kivui*’s protein were taken after blast in representative genomes of GTDB rs202. Redundant proteins were discarded with CD-HIT at 100% cut-off value. The ML tree with 100 bootstrap replicates was constructed with MEGA-6 after alignment with built-in ClustalW. Bootstrap values lower than 50 are not shown.

**
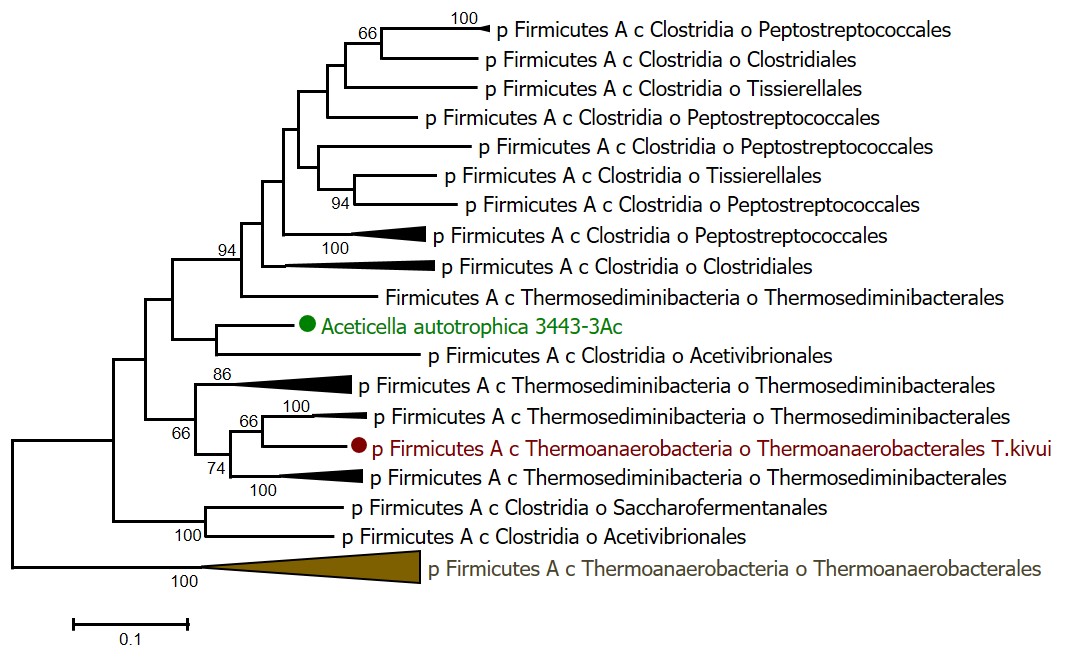
**

**Figure S16.** Fhs, formyl-THF synthetase. 25 best hits of *A. autotrophica*’s protein and 25 best hits of *T. kivui*’s protein were taken after blast in representative genomes of GTDB rs202. Redundant proteins were discarded with CD-HIT at 100% cut-off value. The ML tree with 100 bootstrap replicates was constructed with MEGA-6 after alignment with built-in ClustalW. Bootstrap values lower than 50 are not shown.

**
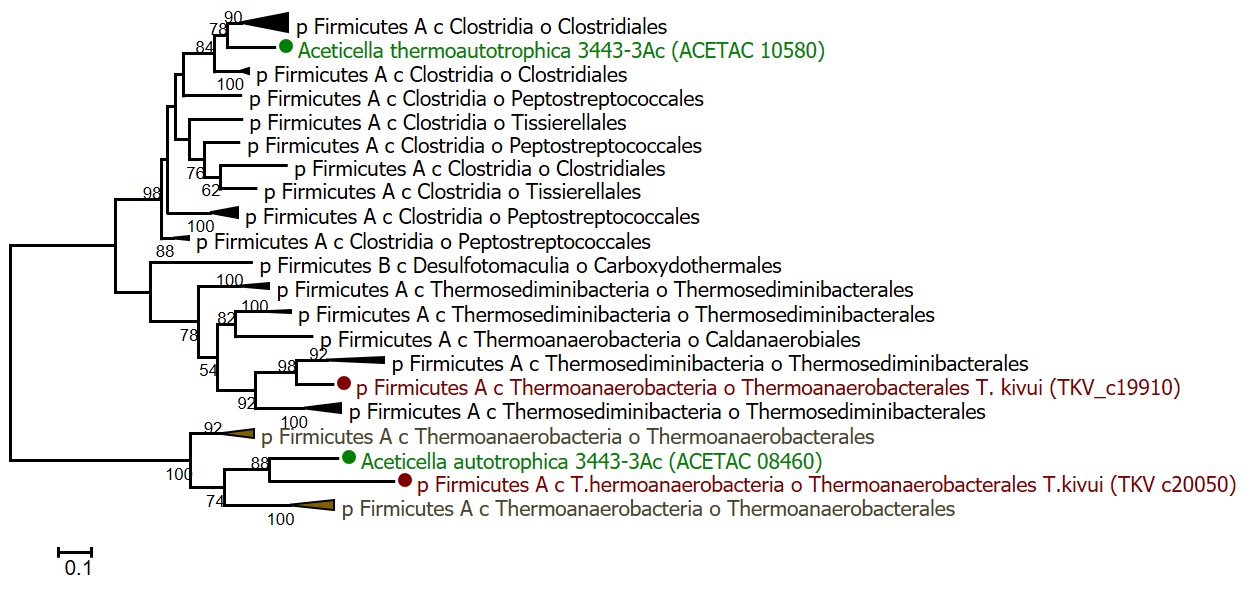
**

**Figure S17.** FchA, methenyl-THF cyclohydrolase. 25 best hits of *A. autotrophica*’s protein and 25 best hits of *T. kivui*’s protein were taken after blast in representative genomes of GTDB rs202. Homologous proteins of TTC group representatives from the same database were added because the same enzymatic activity could be assumed. Redundant proteins were discarded with CD-HIT at 100% cut-off value. The ML tree with 100 bootstrap replicates was constructed with MEGA-6 after alignment with built-in ClustalW. Bootstrap values lower than 50 are not shown.

**
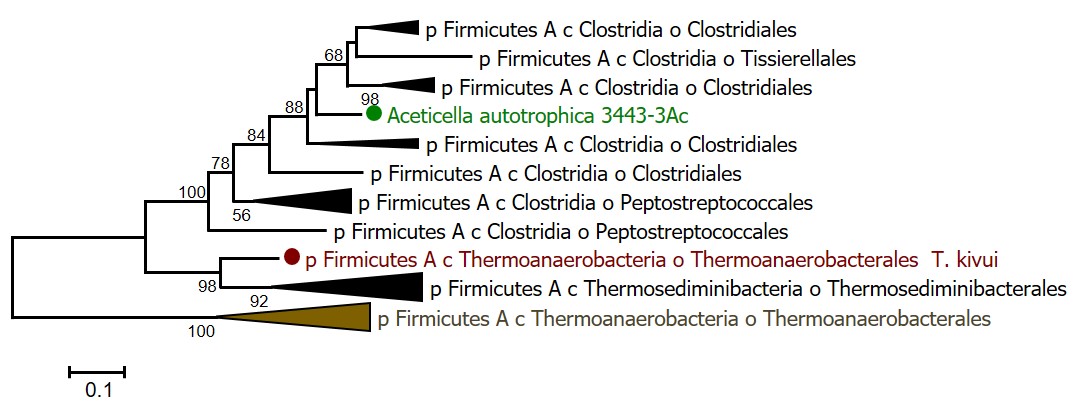
**

**Figure S18.** FolD, methylene-THF dehydrogenase. 25 best hits of *A. autotrophica*’s protein and 25 best hits of *T. kivui*’s protein were taken after blast in representative genomes of GTDB rs202. Redundant proteins were discarded with CD-HIT at 100% cut-off value. The ML tree with 100 bootstrap replicates was constructed with MEGA-6 after alignment with built-in ClustalW. Bootstrap values lower than 50 are not shown.

**
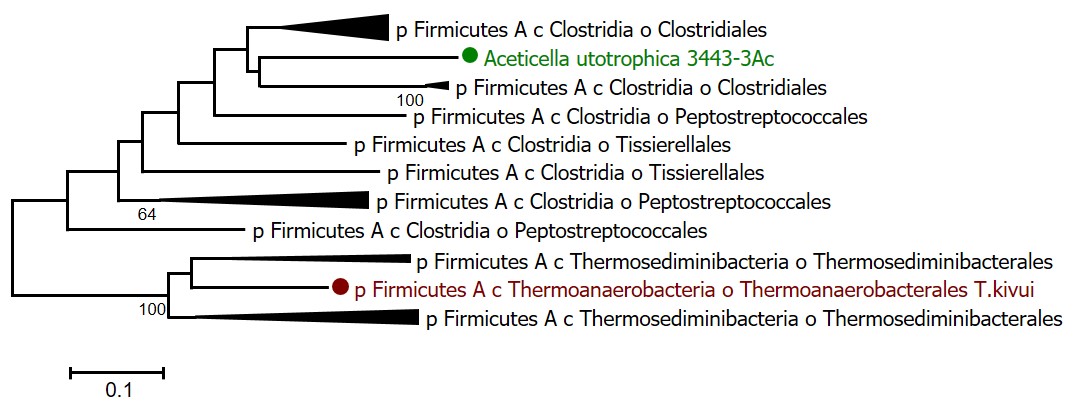
**

**Figure S19.** MetV subunit of methylene-THF reductase. 25 best hits of *A. autotrophica*’s protein and 25 best hits of *T. kivui*’s protein were taken after blast in representative genomes of GTDB rs202. Redundant proteins were discarded with CD-HIT at 100% cut-off value. The ML tree with 100 bootstrap replicates was constructed with MEGA-6 after alignment with built-in ClustalW. Bootstrap values lower than 50 are not shown.

**
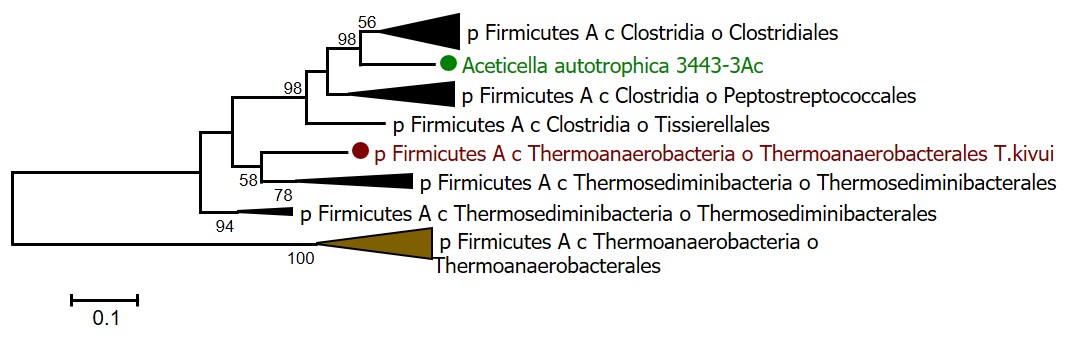
**

**Figure S20.** MetF subunit of methylene-THF reductase. 25 best hits of *A. autotrophica*’s protein and 25 best hits of *T. kivui*’s protein were taken after blast in representative genomes of GTDB rs202. Redundant proteins were discarded with CD-HIT at 100% cut-off value. The ML tree with 100 bootstrap replicates was constructed with MEGA-6 after alignment with built-in ClustalW. Bootstrap values lower than 50 are not shown.

**Figure S21.** A subtree of a NJ tree of Ni- CO dehydrogenases of bacterial type, termed AcsA or CooS as dependent on the function. The entire tree is shown in emf file Fig. Supplementary Material 6. The entire tree was constructed for all 3553 bacterial-type Ni-CO dehydrogenases found in representative genomes of GTDB rs202 (47,894 genomes). The subtree represents a clade (having a bootstrap support of 93%) which is dominated by AcsAs (as judged by clustering in genomes with AscB, shown in emf file Supplementary Material 5) from representatives of p_Firmicutes_A c_Clostridia and includes virtually all AcsAs from this GTDB taxon (with a few exceptions apparently due to HGT events). In the subtree, the effects of HGT events can also be noted when comparing the tree topology with the GTDB phylogeny-grounded taxonomy.

**Figure S22.** Another subtree of the NJ tree of Ni- CO dehydrogenases of bacterial type, shown in emf file Supplementary Material 6. The subtree represents a clade (having a bootstrap support of 99%) which is dominated by CooSes (as judged by lack of clustering in genomes with AscB, shown in emf file Supplementary Material 5) from representatives of p_Firmicutes_A c_Clostridia and includes virtually all CooSes from this GTDB taxon (with a few exceptions apparently due to HGT events). In the subtree, the effects of HGT events can also be noted when comparing the tree topology with the GTDB phylogeny-grounded taxonomy.


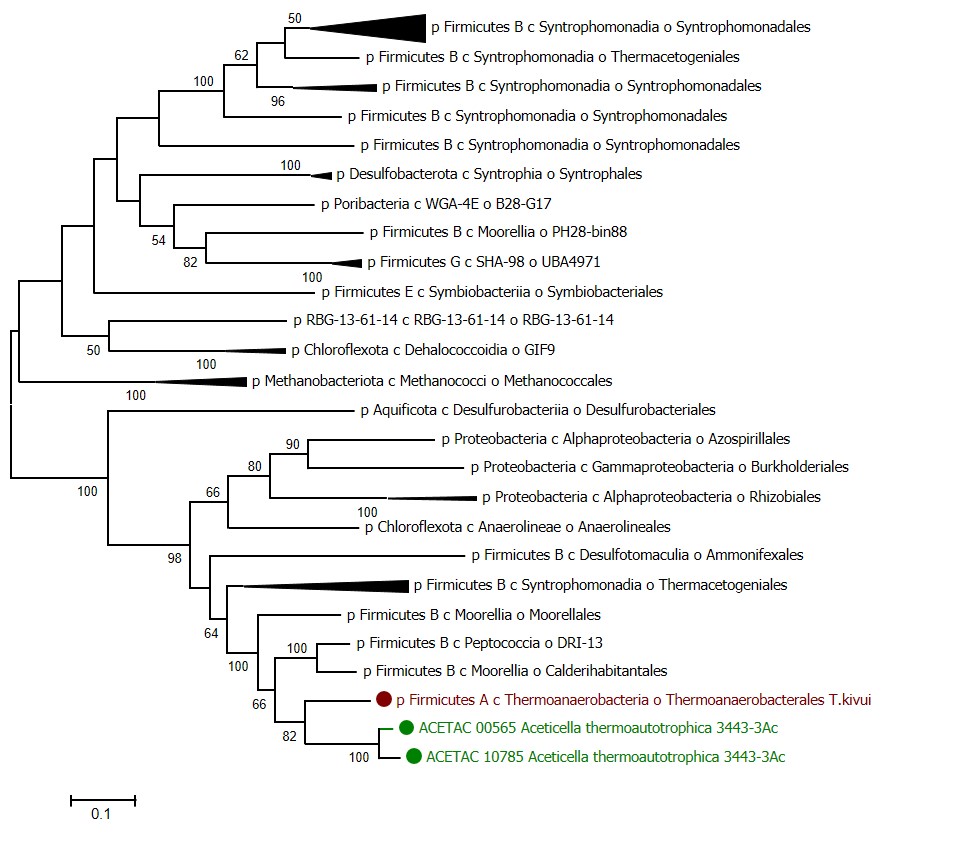


**Figure S23.** ML tree of formate dehydrogenase FdhF. 25 best hits of *A. autotrophica*’s protein and 25 best hits of *T. kivui*’s protein were taken after blast in representative genomes of GTDB rs202. Redundant proteins were discarded with CD-HIT at 100% cut-off value. The ML tree with 100 bootstrap replicates was constructed with MEGA-6 after alignment with built-in ClustalW. Bootstrap values lower than 50 are not shown.

**Figure S24.** ML tree of HydA, the catalytic subunit of bifurcating Fe,Fe-hydrogenase. 25 best hits of *A. autotrophica*’s protein and 25 best hits of *T. kivui*’s protein were taken after blast in representative genomes of GTDB rs202. Redundant proteins were discarded with CD-HIT at 100% cut-off value. The ML tree with 100 bootstrap replicates was constructed with MEGA-6 after alignment with built-in ClustalW. Bootstrap values lower than 50 are not shown. Most of the hydA genes represented in the tree occurred in the gene clusters hydCBA, typical of biochemically characterized bifurcating Fe,Fe-hydrogenases.


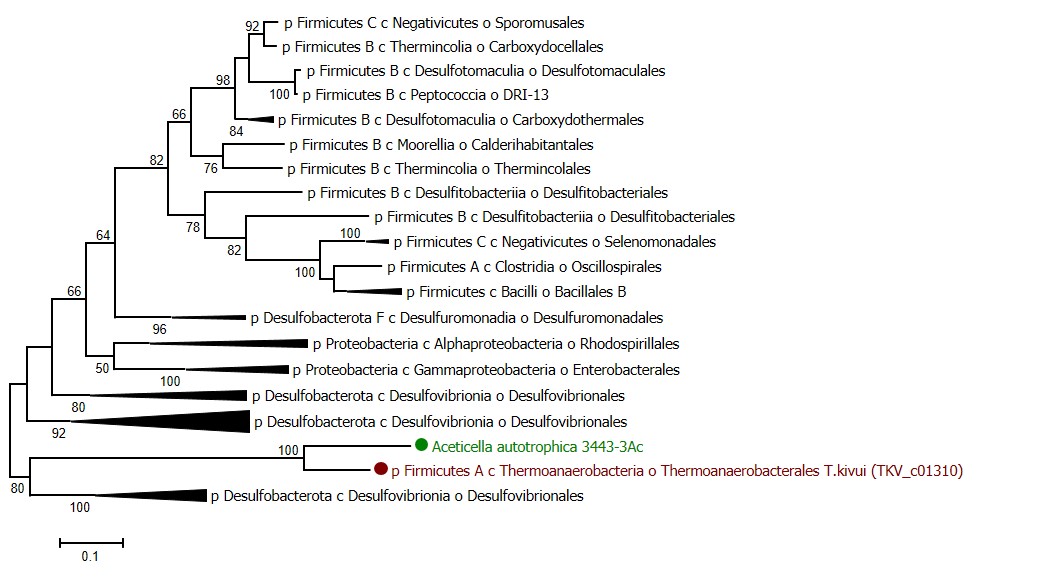


**Figure S25.** ML tree of EchE, the catalytic subunit of energy-converting hydrogenase. 25 best hits of *A. autotrophica*’s ACETAC_10680 protein and 25 best hits of *T. kivui*’s TKV_c01310 protein were taken after blast in representative genomes of GTDB rs202. Redundant proteins were discarded with CD-HIT at 100% cut-off value. The ML tree with 100 bootstrap replicates was constructed with MEGA-6 after alignment with built-in ClustalW. Bootstrap values lower than 50 are not shown. Of the two energy-converting hydrogenases of *T. kivui*, represented (by the catalytic subunit TKV_c01310) is the one closely related to the *A. autotrophica*’s enzymatic complex.

**Supplementary Material 2.** Genomic islands table.

**Supplementary Material 3.** COG functional categories distribution table.

**Supplementary Material 4.** Carbohydrate metabolism related COG sets found in genomes of TTC members.

**Supplementary Material 5.** Acetyl-CoA synthase AscB ML tree. The tree was constructed for all 1654 AscBs (acetyl-CoA synthases of bacterial type) found in representative genomes of GTDB rs202 (47,894 genomes). The entire ML tree with 100 bootstrap replicates was constructed with MEGA-6 after alignment with MAFFT version 7. *A. autorophica* and *T. kivui* are in green and brown font, resepectively. In the tree, protein names include assembly accessions, taxonomic position of the organism in GTDB rs202 (phylum, class, and order affiliation), and the organization of gene clusters around *acsB* genes. Gene designations (in alphabetic order) are as follows: *acsA*, CO dehydrogenase gene clustered with *acsB* gene; *acsB*, acetyl-CoA synthase gene; *acsC*, gene for corrinoid iron sulfur protein large subunit; *acsD*, gene for corrinoid iron sulfur protein small subunit; *acsE*, methyl transferase gene; *acsV*, gene for acetyl-CoA synthase corrinoid activation protein; COG1810, gene of unknown function, present in some WLP gene clusters; *cooC*, gene for maturation factor of the CO dehydrogenase; *fhs*, formyl-THF synthetase gene; *fchA*, methenyl-THF cyclohydrolase gene; *folD*, methylene-THF dehydrogenase gene; *gcsH*, gene for protein H of a glycine cleavage system; *metF*, geme fpr methylene-THF reductase MetF subunit; *metV*, gene for methylene-THF reductase metV subunit; *pdhD*, dihydrolipoamide dehydrogenase gene; 1X, 2X etc. designate N*500-nt gaps in which genes may be present that could not be identified by the method employed.

**Supplementary Material 6.** NJ tree of Ni- CO dehydrogenases of bacterial type, termed AcsA or CooS as dependent on the function. The tree was constructed for all 3553 bacterial-type Ni- CO dehydrogenases found in representative genomes of GTDB rs202 (47,894 genomes). The NJ tree with 100 bootstrap replicates was constructed with MEGA-6 after alignment with MAFFT version 7. *A. autoro*phica and *T. kivui* are in green and brown font, resepectively. In the tree, protein names include assembly accessions, taxonomic position of the organism in GTDB rs202 (phylum, class, and order affiliation), and the statement (if valid) on the presence of a closely located (within a 15-kb distance) *acsB* gene (‘*acsB*-c’ mark at the beginning of the protein name).
