# Supplementary material for: Obligate autotrophy at the thermodynamic limit of life in a new acetogenic bacterium
Source: Front Microbiol. 2023 May 12;14:1185739. doi: 10.3389/fmicb.2023.1185739 (PMC10213532; doi:10.3389/fmicb.2023.1185739)
Supplement: Supplementary file 5 [file Data_Sheet_5.PDF]

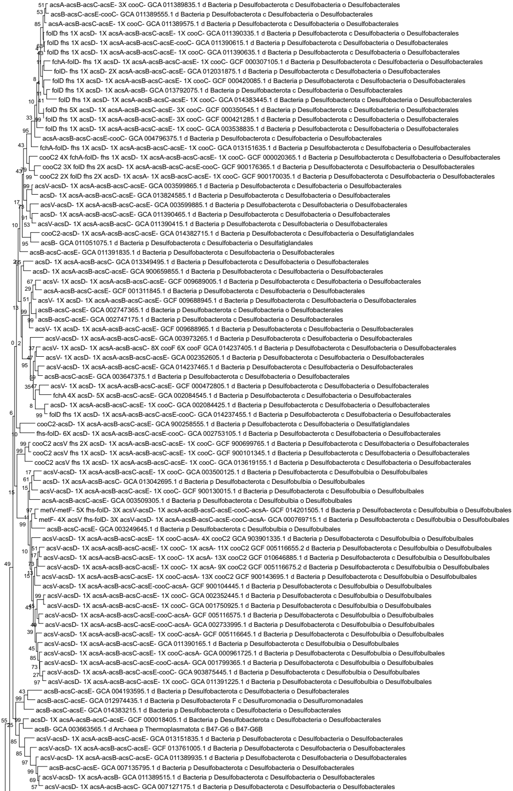
acsA-acsB-acsC-acsE- 3X cooC- GCA 011389835.1 d Bacteria p Desulfobacterota c Desulfobacteria o Desulfobacterales
acsB-acsC-acsE-cooC- GCA 011389555.1 d Bacteria p Desulfobacterota c Desulfobacteria o Desulfobacterales
acsA-acsB-acsC-acsE- 1X cooC- GCA 011389575.1 d Bacteria p Desulfobacterota c Desulfobacteria o Desulfobacterales
folD fhs 1X acsD- 1X acsA-acsB-acsC-acsE- 1X cooC- GCA 011390335.1 d Bacteria p Desulfobacterota c Desulfobacteria o Desulfobacterales
folD fhs 1X acsD- 1X acsA-acsB-acsC-acsE-cooC- GCA 011390615.1 d Bacteria p Desulfobacterota c Desulfobacteria o Desulfobacterales
folD fhs 1X acsD- 1X acsA-acsB-acsC-acsE- 1X cooC- GCA 011390635.1 d Bacteria p Desulfobacterota c Desulfobacteria o Desulfobacterales
fchA-folD- fhs 1X acsD- 1X acsA-acsB-acsC-acsE- 1X cooC- GCF 000307105.1 d Bacteria p Desulfobacterota c Desulfobacteria o Desulfobacterales
folD- fhs 1X acsD- 2X acsA-acsB-acsC-acsE- GCA 012031875.1 d Bacteria p Desulfobacterota c Desulfobacteria o Desulfobacterales
folD fhs 1X acsD- 1X acsA-acsB-acsC-acsE- 1X cooC- GCF 000420085.1 d Bacteria p Desulfobacterota c Desulfobacteria o Desulfobacterales
folD fhs 1X acsD- 1X acsA-acsB- GCA 013792075.1 d Bacteria p Desulfobacterota c Desulfobacteria o Desulfobacterales
folD fhs 1X acsD- 1X acsA-acsB-acsC-acsE- 1X cooC- GCA 014383445.1 d Bacteria p Desulfobacterota c Desulfobacteria o Desulfobacterales
folD fhs 5X acsD- 1X acsA-acsB-acsC-acsE- 3X cooC- GCF 000350545.1 d Bacteria p Desulfobacterota c Desulfobacteria o Desulfobacterales
folD fhs 1X acsD- 1X acsA-acsB-acsC-acsE- 3X cooC- GCF 000421285.1 d Bacteria p Desulfobacterota c Desulfobacteria o Desulfobacterales
folD fhs 1X acsD- 1X acsA-acsB-acsC-acsE- 1X cooC- GCA 003538835.1 d Bacteria p Desulfobacterota c Desulfobacteria o Desulfobacterales
acsA-acsB-acsC-acsE-cooC- GCA 004796375.1 d Bacteria p Desulfobacterota c Desulfobacteria o Desulfobacterales
fchA-folD- fhs 1X acsD- 1X acsA-acsB-acsC-acsE- 1X cooC- GCA 013151635.1 d Bacteria p Desulfobacterota c Desulfobacteria o Desulfobacterales
cooC2 4X fchA-folD- fhs 1X acsD- 1X acsA-acsB-acsC-acsE- 1X cooC- GCF 000020365.1 d Bacteria p Desulfobacterota c Desulfobacteria o Desulfobacterales
cooC2 3X folD fhs 2X acsD- 1X acsA-acsB-acsC-acsE-cooC- GCF 900176365.1 d Bacteria p Desulfobacterota c Desulfobacteria o Desulfobacterales
cooC2 2X folD fhs 2X acsD- 1X acsA- 1X acsB-acsC-acsE- 1X cooC- GCF 900170035.1 d Bacteria p Desulfobacterota c Desulfobacteria o Desulfobacterales
acsV-acsD- 1X acsA-acsB-acsC-acsE- GCA 003599865.1 d Bacteria p Desulfobacterota c Desulfobacteria o Desulfobacterales
acsD- 1X acsA-acsB-acsC-acsE- GCA 013824585.1 d Bacteria p Desulfobacterota c Desulfobacteria o Desulfobacterales
acsV-acsD- 1X acsA-acsB-acsC-acsE- GCA 003599885.1 d Bacteria p Desulfobacterota c Desulfobacteria o Desulfobacterales
acsD- 1X acsA-acsB-acsC-acsE- GCA 011390465.1 d Bacteria p Desulfobacterota c Desulfobacteria o Desulfobacterales
acsV-acsD- 1X acsA-acsB-acsC- GCA 011390415.1 d Bacteria p Desulfobacterota c Desulfobacteria o Desulfobacterales
cooC2-acsD- 1X acsA-acsB-acsC-acsE- GCA 014382715.1 d Bacteria p Desulfobacterota c Desulfobacteria o Desulfatiglandales
acsB- GCA 011051075.1 d Bacteria p Desulfobacterota c Desulfobacteria o Desulfatiglandales
acsB-acsC-acsE- GCA 011391835.1 d Bacteria p Desulfobacterota c Desulfobacteria o Desulfobacterales
acsD- 1X acsA-acsB-acsC- GCA 013349495.1 d Bacteria p Desulfobacterota c Desulfobacteria o Desulfobacterales
acsD- 1X acsA-acsB-acsC-acsE- GCA 900659855.1 d Bacteria p Desulfobacterota c Desulfobacteria o Desulfobacterales
acsV- 1X acsD- 1X acsA-acsB-acsC-acsE- GCF 009689005.1 d Bacteria p Desulfobacterota c Desulfobacteria o Desulfobacterales
acsA-acsB-acsC-acsE- GCF 001311845.1 d Bacteria p Desulfobacterota c Desulfobacteria o Desulfobacterales
acsV- 1X acsD- 1X acsA-acsB-acsC-acsE- GCF 009688945.1 d Bacteria p Desulfobacterota c Desulfobacteria o Desulfobacterales
acsB-acsC-acsE- GCA 002747365.1 d Bacteria p Desulfobacterota c Desulfobacteria o Desulfobacterales
acsB-acsC-acsE- GCA 002747175.1 d Bacteria p Desulfobacterota c Desulfobacteria o Desulfobacterales
acsV- 1X acsD- 1X acsA-acsB-acsC-acsE- GCF 009688965.1 d Bacteria p Desulfobacterota c Desulfobacteria o Desulfobacterales
acsV-acsD- 1X acsA-acsB-acsC-acsE- GCA 003973265.1 d Bacteria p Desulfobacterota c Desulfobacteria o Desulfobacterales
acsV- 1X acsD- 1X acsA-acsB-acsC- 8X cooF 6X cooF GCA 014237405.1 d Bacteria p Desulfobacterota c Desulfobacteria o Desulfobacterales
acsV- 1X acsD- 1X acsA-acsB-acsC-acsE- GCA 002352605.1 d Bacteria p Desulfobacterota c Desulfobacteria o Desulfobacterales
acsV-acsD- 1X acsA-acsB-acsC-acsE- GCA 014237465.1 d Bacteria p Desulfobacterota c Desulfobacteria o Desulfobacterales
acsB-acsC-acsE- GCA 003647375.1 d Bacteria p Desulfobacterota c Desulfobacteria o Desulfobacterales
acsV- 1X acsD- 1X acsA-acsB-acsC-acsE- GCF 000472805.1 d Bacteria p Desulfobacterota c Desulfobacteria o Desulfobacterales
fchA 4X acsD- 5X acsB-acsC-acsE- GCA 002084545.1 d Bacteria p Desulfobacterota c Desulfobacteria o Desulfobacterales
acsD- 1X acsA-acsB-acsC-acsE- 1X cooC- GCA 002084425.1 d Bacteria p Desulfobacterota c Desulfobacteria o Desulfobacterales
folD fhs 1X acsD- 1X acsA-acsB-acsC-acsE-cooC- GCA 014237455.1 d Bacteria p Desulfobacterota c Desulfobacteria o Desulfobacterales
cooC2-acsD- 1X acsA-acsB-acsC-acsE- GCA 900258555.1 d Bacteria p Desulfobacterota c Desulfobacteria o Desulfatiglandales
fhs-folD- 6X acsD- 1X acsA-acsB-acsC-acsE-cooC- GCA 002753105.1 d Bacteria p Desulfobacterota c Desulfobacteria o Desulfobacterales
cooC2 acsV fhs 2X acsD- 1X acsA-acsB-acsC-acsE- 1X cooC- GCF 900699765.1 d Bacteria p Desulfobacterota c Desulfobacteria o Desulfobacterales
cooC2 acsV fhs 1X acsD- 1X acsA-acsB-acsC-acsE- 1X cooC- GCF 900101345.1 d Bacteria p Desulfobacterota c Desulfobacteria o Desulfobacterales
cooC2 acsV fhs 1X acsD- 1X acsA-acsB-acsC-acsE- 1X cooC- GCA 013619155.1 d Bacteria p Desulfobacterota c Desulfobacteria o Desulfobacterales
acsV-acsD- 1X acsA-acsB-acsC-acsE- 1X cooC- GCA 003500125.1 d Bacteria p Desulfobacterota c Desulfobulbia o Desulfobulbales
acsD- 1X acsA-acsB-acsC- GCA 013042695.1 d Bacteria p Desulfobacterota c Desulfobulbia o Desulfobulbales
acsV-acsD- 1X acsA-acsB-acsC-acsE- 1X cooC- GCF 900130015.1 d Bacteria p Desulfobacterota c Desulfobulbia o Desulfobulbales
acsA-acsB-acsC-acsE- GCA 003509305.1 d Bacteria p Desulfobacterota c Desulfobulbia o Desulfobulbales
metV-metF- 5X fhs-folD- 3X acsV-acsD- 1X acsA-acsB-acsC-acsE-cooC-acsA- GCF 014201505.1 d Bacteria p Desulfobacterota c Desulfobulbia o Desulfobulbales
metF- 4X acsV fhs-folD- 3X acsV-acsD- 1X acsA-acsB-acsC-acsE-cooC-acsA- GCA 000769715.1 d Bacteria p Desulfobacterota c Desulfobulbia o Desulfobulbales
acsB-acsC-acsE- GCA 003249645.1 d Bacteria p Desulfobacterota c Desulfobulbia o Desulfobulbales
acsV-acsD- 1X acsA-acsB-acsC-acsE- 1X cooC-acsA- 4X cooC2 GCA 903901335.1 d Bacteria p Desulfobacterota c Desulfobulbia o Desulfobulbales
acsV-acsD- 1X acsA-acsB-acsC-acsE- 1X cooC- 1X acsA- 11X cooC2 GCF 005116655.2 d Bacteria p Desulfobacterota c Desulfobulbia o Desulfobulbales
acsV-acsD- 1X acsA-acsB-acsC-acsE- 1X cooC- 1X acsA- 13X cooC2 GCF 010646885.1 d Bacteria p Desulfobacterota c Desulfobulbia o Desulfobulbales
acsV-acsD- 1X acsA-acsB-acsC-acsE- 1X cooC- 1X acsA- 9X cooC2 GCF 005116675.2 d Bacteria p Desulfobacterota c Desulfobulbia o Desulfobulbales
acsV-acsD- 1X acsA-acsB-acsC-acsE- 1X cooC-acsA- 13X cooC2 GCF 900143695.1 d Bacteria p Desulfobacterota c Desulfobulbia o Desulfobulbales
acsV-acsD- 1X acsA-acsB-acsC-acsE-cooC-acsA- GCF 900104445.1 d Bacteria p Desulfobacterota c Desulfobulbia o Desulfobulbales
acsV-acsD- 1X acsA-acsB-acsC-acsE- 1X cooC- GCA 002352445.1 d Bacteria p Desulfobacterota c Desulfobulbia o Desulfobulbales
acsV-acsD- 1X acsA-acsB-acsC-acsE- 1X cooC- GCA 001750925.1 d Bacteria p Desulfobacterota c Desulfobulbia o Desulfobulbales
acsV-acsD- 1X acsA-acsB-acsC-acsE-cooC-acsA- GCF 005116575.1 d Bacteria p Desulfobacterota c Desulfobulbia o Desulfobulbales
acsV-acsD- 1X acsA-acsB-acsC-acsE-cooC-acsA- GCA 002733995.1 d Bacteria p Desulfobacterota c Desulfobulbia o Desulfobulbales
acsV-acsD- 1X acsA-acsB-acsC-acsE- 1X cooC-acsA- GCF 005116645.1 d Bacteria p Desulfobacterota c Desulfobulbia o Desulfobulbales
acsV-acsD- 1X acsA-acsB-acsC-acsE- GCA 011390165.1 d Bacteria p Desulfobacterota c Desulfobulbia o Desulfobulbales
acsV-acsD- 1X acsA-acsB-acsC-acsE- 1X cooC-acsA- GCA 000961725.1 d Bacteria p Desulfobacterota c Desulfobulbia o Desulfobulbales
acsV-acsD- 1X acsA-acsB-acsC-acsE-cooC-acsA- GCA 001799365.1 d Bacteria p Desulfobacterota c Desulfobulbia o Desulfobulbales
acsV-acsD- 1X acsA-acsB-acsC-acsE-cooC- GCA 903875445.1 d Bacteria p Desulfobacterota c Desulfobulbia o Desulfobulbales
acsV-acsD- 1X acsA-acsB-acsC-acsE- 1X cooC- GCA 011391225.1 d Bacteria p Desulfobacterota c Desulfobulbia o Desulfobulbales
acsB-acsC-acsE- GCA 004193595.1 d Bacteria p Desulfobacterota c Desulfobacteria o Desulfobacterales
acsB-acsC-acsE- GCA 012974435.1 d Bacteria p Desulfobacterota F c Desulfuromonadia o Desulfuromonadales
acsB-acsC-acsE- GCA 014383215.1 d Bacteria p Desulfobacterota c Desulfobacteria o Desulfobacterales
acsD- 1X acsA-acsB-acsC-acsE- GCF 000018405.1 d Bacteria p Desulfobacterota c Desulfobacteria o Desulfobacterales
acsB- GCA 003663565.1 d Archaea p Thermoplasmatota c B47-G6 o B47-G6B
acsV-acsD- 1X acsA-acsB-acsC-acsE- GCA 013151835.1 d Bacteria p Desulfobacterota c Desulfobacteria o Desulfobacterales
acsV-acsD- 1X acsA-acsB-acsC-acsE- GCF 013761005.1 d Bacteria p Desulfobacterota c Desulfobacteria o Desulfobacterales
acsV-acsD- 1X acsA-acsB-acsC-acsE- GCA 011389935.1 d Bacteria p Desulfobacterota c Desulfobacteria o Desulfobacterales
acsB-acsC-acsE- GCA 007135795.1 d Bacteria p Desulfobacterota c Desulfobacteria o Desulfobacterales
acsV-acsD- 1X acsA-acsB- GCA 011389515.1 d Bacteria p Desulfobacterota c Desulfobacteria o Desulfobacterales
acsV-acsD- 1X acsA-acsB-acsC- GCA 007127175.1 d Bacteria p Desulfobacterota c Desulfobacteria o Desulfobacterales

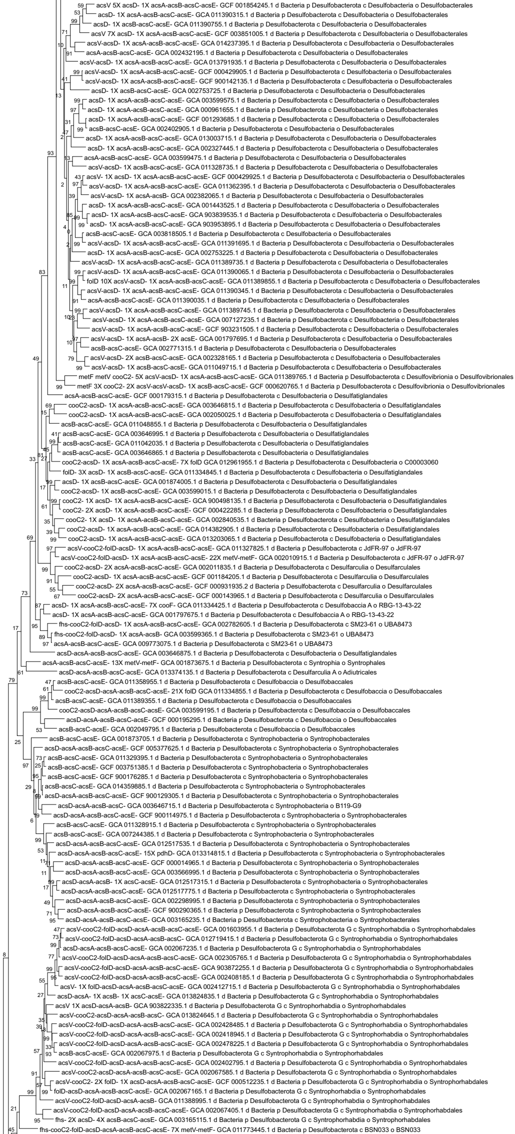
acsV 5X acsD- 1X acsA-acsB-acsC-acsE- GCF 001854245.1 d Bacteria p Desulfobacterota c Desulfobacteria o Desulfobacterales
acsD- 1X acsA-acsB-acsC-acsE- GCA 011390315.1 d Bacteria p Desulfobacterota c Desulfobacteria o Desulfobacterales
acsD- 1X acsB-acsC-acsE- GCA 011390755.1 d Bacteria p Desulfobacterota c Desulfobacteria o Desulfobacterales
acsV 7X acsD- 1X acsA-acsB-acsC-acsE- GCF 003851005.1 d Bacteria p Desulfobacterota c Desulfobacteria o Desulfobacterales
acsV-acsD- 1X acsA-acsB-acsC-acsE- GCA 014237395.1 d Bacteria p Desulfobacterota c Desulfobacteria o Desulfobacterales
acsA-acsB-acsC-acsE- GCA 002432195.1 d Bacteria p Desulfobacterota c Desulfobacteria o Desulfobacterales
acsV-acsD- 1X acsA-acsB-acsC-acsE- GCA 013791935.1 d Bacteria p Desulfobacterota c Desulfobacteria o Desulfobacterales
acsV-acsD- 1X acsA-acsB-acsC-acsE- GCF 000429905.1 d Bacteria p Desulfobacterota c Desulfobacteria o Desulfobacterales
acsV-acsD- 1X acsA-acsB-acsC-acsE- GCF 900142135.1 d Bacteria p Desulfobacterota c Desulfobacteria o Desulfobacterales
acsD- 1X acsB-acsC-acsE- GCA 002753725.1 d Bacteria p Desulfobacterota c Desulfobacteria o Desulfobacterales
acsD- 1X acsA-acsB-acsC-acsE- GCA 003599575.1 d Bacteria p Desulfobacterota c Desulfobacteria o Desulfobacterales
acsD- 1X acsA-acsB-acsC-acsE- GCA 000961655.1 d Bacteria p Desulfobacterota c Desulfobacteria o Desulfobacterales
acsD- 1X acsA-acsB-acsC-acsE- GCF 001293685.1 d Bacteria p Desulfobacterota c Desulfobacteria o Desulfobacterales
acsB-acsC-acsE- GCA 002402905.1 d Bacteria p Desulfobacterota c Desulfobacteria o Desulfobacterales
acsD- 1X acsA-acsB-acsC-acsE- GCA 013003715.1 d Bacteria p Desulfobacterota c Desulfobacteria o Desulfobacterales
acsD- 1X acsA-acsB-acsC-acsE- GCA 002327445.1 d Bacteria p Desulfobacterota c Desulfobacteria o Desulfobacterales
acsA-acsB-acsC-acsE- GCA 003599475.1 d Bacteria p Desulfobacterota c Desulfobacteria o Desulfobacterales
acsV-acsD- 1X acsB-acsC-acsE- GCA 011328735.1 d Bacteria p Desulfobacterota c Desulfobacteria o Desulfobacterales
acsV- 1X acsD- 1X acsA-acsB-acsC-acsE- GCF 000429925.1 d Bacteria p Desulfobacterota c Desulfobacteria o Desulfobacterales
acsV-acsD- 1X acsA-acsB-acsC-acsE- GCA 011362395.1 d Bacteria p Desulfobacterota c Desulfobacteria o Desulfobacterales
acsV-acsD- 1X acsA-acsB- GCA 002382065.1 d Bacteria p Desulfobacterota c Desulfobacteria o Desulfobacterales
acsD- 1X acsA-acsB-acsC-acsE- GCA 001443525.1 d Bacteria p Desulfobacterota c Desulfobacteria o Desulfobacterales
acsD- 1X acsA-acsB-acsC-acsE- GCA 903839535.1 d Bacteria p Desulfobacterota c Desulfobacteria o Desulfobacterales
acsD- 1X acsA-acsB-acsC-acsE- GCA 903953895.1 d Bacteria p Desulfobacterota c Desulfobacteria o Desulfobacterales
acsB-acsC-acsE- GCA 003818505.1 d Bacteria p Desulfobacterota c Desulfobacteria o Desulfobacterales
acsV-acsD- 1X acsA-acsB-acsC-acsE- GCA 011391695.1 d Bacteria p Desulfobacterota c Desulfobacteria o Desulfobacterales
acsD- 1X acsA-acsB-acsC-acsE- GCA 002753225.1 d Bacteria p Desulfobacterota c Desulfobacteria o Desulfobacterales
acsV-acsD- 1X acsA-acsB-acsC-acsE- GCA 011389735.1 d Bacteria p Desulfobacterota c Desulfobacteria o Desulfobacterales
acsV-acsD- 1X acsA-acsB-acsC-acsE- GCA 011390065.1 d Bacteria p Desulfobacterota c Desulfobacteria o Desulfobacterales
folD 10X acsV-acsD- 1X acsA-acsB-acsC-acsE- GCA 011389855.1 d Bacteria p Desulfobacterota c Desulfobacteria o Desulfobacterales
acsV-acsD- 1X acsA-acsB-acsC-acsE- GCA 011390345.1 d Bacteria p Desulfobacterota c Desulfobacteria o Desulfobacterales
acsA-acsB-acsC-acsE- GCA 011390035.1 d Bacteria p Desulfobacterota c Desulfobacteria o Desulfobacterales
acsV-acsD- 1X acsA-acsB-acsC-acsE- GCA 011389745.1 d Bacteria p Desulfobacterota c Desulfobacteria o Desulfobacterales
acsV-acsD- 1X acsA-acsB-acsC-acsE- GCA 007127235.1 d Bacteria p Desulfobacterota c Desulfobacteria o Desulfobacterales
acsV-acsD- 1X acsA-acsB-acsC-acsE- GCF 903231505.1 d Bacteria p Desulfobacterota c Desulfobacteria o Desulfobacterales
acsV-acsD- 1X acsA-acsB- 2X acsE- GCA 001797695.1 d Bacteria p Desulfobacterota c Desulfobacteria o Desulfobacterales
acsB-acsC-acsE- GCA 002771315.1 d Bacteria p Desulfobacterota c Desulfobacteria o Desulfobacterales
acsV-acsD- 2X acsB-acsC-acsE- GCA 002328165.1 d Bacteria p Desulfobacterota c Desulfobacteria o Desulfobacterales
acsV-acsD- 1X acsB-acsC-acsE- GCA 011049715.1 d Bacteria p Desulfobacterota c Desulfobacteria o Desulfobacterales
metF metV cooC2- 5X acsV-acsD- 1X acsA-acsB-acsC-acsE- GCA 011389765.1 d Bacteria p Desulfobacterota c Desulfovibrionia o Desulfovibrionales
metF 3X cooC2- 2X acsV-acsV-acsD- 1X acsB-acsC-acsE- GCF 000620765.1 d Bacteria p Desulfobacterota c Desulfovibrionia o Desulfovibrionales
acsA-acsB-acsC-acsE- GCF 000179315.1 d Bacteria p Desulfobacterota c Desulfobacteria o Desulfatiglandales
cooC2-acsD- 1X acsA-acsB-acsC-acsE- GCA 003646815.1 d Bacteria p Desulfobacterota c Desulfobacteria o Desulfatiglandales
cooC2-acsD- 1X acsA-acsB-acsC-acsE- GCA 002050025.1 d Bacteria p Desulfobacterota c Desulfobacteria o Desulfatiglandales
acsB-acsC-acsE- GCA 011048855.1 d Bacteria p Desulfobacterota c Desulfobacteria o Desulfatiglandales
acsB-acsC-acsE- GCA 003646995.1 d Bacteria p Desulfobacterota c Desulfobacteria o Desulfatiglandales
acsB-acsC-acsE- GCA 011042035.1 d Bacteria p Desulfobacterota c Desulfobacteria o Desulfatiglandales
acsB-acsC-acsE- GCA 003646865.1 d Bacteria p Desulfobacterota c Desulfobacteria o Desulfatiglandales
cooC2-acsD- 1X acsA-acsB-acsC-acsE- 7X folD GCA 012961955.1 d Bacteria p Desulfobacterota c Desulfobacteria o C00003060
folD- 3X acsD- 1X acsB-acsC-acsE- GCA 011334845.1 d Bacteria p Desulfobacterota c Desulfobacteria o Desulfatiglandales
acsD- 1X acsB-acsC-acsE- GCA 001874005.1 d Bacteria p Desulfobacterota c Desulfobacteria o Desulfatiglandales
cooC2-acsD- 1X acsB-acsC-acsE- GCA 003599015.1 d Bacteria p Desulfobacterota c Desulfobacteria o Desulfatiglandales
cooC2- 1X acsD- 1X acsA-acsB-acsC-acsE- GCA 900498135.1 d Bacteria p Desulfobacterota c Desulfobacteria o Desulfatiglandales
cooC2- 2X acsD- 1X acsA-acsB-acsC-acsE- GCF 000422285.1 d Bacteria p Desulfobacterota c Desulfobacteria o Desulfatiglandales
cooC2- 1X acsD- 1X acsA-acsB-acsC-acsE- GCA 002840535.1 d Bacteria p Desulfobacterota c Desulfobacteria o Desulfatiglandales
cooC2-acsD- 1X acsA-acsB-acsC-acsE- GCA 014382905.1 d Bacteria p Desulfobacterota c Desulfobacteria o Desulfatiglandales
cooC2-acsD- 1X acsA-acsB-acsC-acsE- GCA 013203065.1 d Bacteria p Desulfobacterota c Desulfobacteria o Desulfatiglandales
acsV-cooC2-folD-acsD- 1X acsA-acsB-acsC-acsE- GCA 011327825.1 d Bacteria p Desulfobacterota c JdFR-97 o JdFR-97
acsV-cooC2-folD-acsD- 1X acsA-acsB-acsC-acsE- 22X metV-metF- GCA 002010915.1 d Bacteria p Desulfobacterota c JdFR-97 o JdFR-97
cooC2-acsD- 2X acsA-acsB-acsC-acsE- GCA 002011835.1 d Bacteria p Desulfobacterota c Desulfarculia o Desulfarculales
cooC2-acsD- 1X acsA-acsB-acsC-acsE- GCF 001184205.1 d Bacteria p Desulfobacterota c Desulfarculia o Desulfarculales
cooC2-acsD- 2X acsA-acsB-acsC-acsE- GCF 000931935.2 d Bacteria p Desulfobacterota c Desulfarculia o Desulfarculales
cooC2-acsD- 2X acsA-acsB-acsC-acsE- GCF 000143965.1 d Bacteria p Desulfobacterota c Desulfarculia o Desulfarculales
acsD- 1X acsA-acsB-acsC-acsE- 7X cooF- GCA 011334425.1 d Bacteria p Desulfobacterota c Desulfobaccia A o RBG-13-43-22
acsD- 1X acsA-acsB-acsC-acsE- GCA 001797675.1 d Bacteria p Desulfobacterota c Desulfobaccia A o RBG-13-43-22
fhs-cooC2-folD-acsD- 1X acsA-acsB-acsC-acsE- GCA 002782605.1 d Bacteria p Desulfobacterota c SM23-61 o UBA8473
fhs-cooC2-folD-acsD- 1X acsA-acsB- GCA 003599365.1 d Bacteria p Desulfobacterota c SM23-61 o UBA8473
acsA-acsB-acsC-acsE- GCA 009773075.1 d Bacteria p Desulfobacterota c SM23-61 o UBA8473
acsD-acsA-acsB-acsC-acsE- GCA 003646875.1 d Bacteria p Desulfobacterota c Desulfobacteria o Desulfatiglandales
acsA-acsB-acsC-acsE- 13X metV-metF- GCA 001873675.1 d Bacteria p Desulfobacterota c Syntrophia o Syntrophales
acsD-acsA-acsB-acsC-acsE- GCA 013374135.1 d Bacteria p Desulfobacterota c Desulfarculia A o Adiutricales
acsB-acsC-acsE- GCA 011358955.1 d Bacteria p Desulfobacterota c Desulfobaccia o Desulfobaccales
cooC2-acsD-acsA-acsB-acsC-acsE- 21X folD GCA 011334855.1 d Bacteria p Desulfobacterota c Desulfobaccia o Desulfobaccales
acsB-acsC-acsE- GCA 011389355.1 d Bacteria p Desulfobacterota c Desulfobaccia o Desulfobaccales
cooC2-acsD-acsA-acsB-acsC-acsE- GCA 003599195.1 d Bacteria p Desulfobacterota c Desulfobaccia o Desulfobaccales
acsD-acsA-acsB-acsC-acsE- GCF 000195295.1 d Bacteria p Desulfobacterota c Desulfobaccia o Desulfobaccales
acsB-acsC-acsE- GCA 002049795.1 d Bacteria p Desulfobacterota c Desulfobaccia o Desulfobaccales
acsB-acsC-acsE- GCA 001873705.1 d Bacteria p Desulfobacterota c Syntrophobacteria o Syntrophobacterales
acsD-acsA-acsB-acsC-acsE- GCF 005377625.1 d Bacteria p Desulfobacterota c Syntrophobacteria o Syntrophobacterales
acsB-acsC-acsE- GCA 011329395.1 d Bacteria p Desulfobacterota c Syntrophobacteria o Syntrophobacterales
acsB-acsC-acsE- GCF 003751385.1 d Bacteria p Desulfobacterota c Syntrophobacteria o Syntrophobacterales
acsB-acsC-acsE- GCF 900176285.1 d Bacteria p Desulfobacterota c Syntrophobacteria o Syntrophobacterales
acsB-acsC-acsE- GCA 014359885.1 d Bacteria p Desulfobacterota c Syntrophobacteria o Syntrophobacterales
acsD-acsA-acsB-acsC-acsE- GCF 900129305.1 d Bacteria p Desulfobacterota c Syntrophobacteria o Syntrophobacterales
acsD-acsA-acsB-acsC- GCA 003646715.1 d Bacteria p Desulfobacterota c Syntrophobacteria o B119-G9
acsD-acsA-acsB-acsC-acsE- GCF 900114975.1 d Bacteria p Desulfobacterota c Syntrophobacteria o Syntrophobacterales
acsB-acsC-acsE- GCA 011328915.1 d Bacteria p Desulfobacterota c Syntrophobacteria o Syntrophobacterales
acsB-acsC-acsE- GCA 007244385.1 d Bacteria p Desulfobacterota c Syntrophobacteria o Syntrophobacterales
acsD-acsA-acsB-acsC-acsE- GCA 012517535.1 d Bacteria p Desulfobacterota c Syntrophobacteria o Syntrophobacterales
acsD-acsA-acsB-acsC-acsE- 15X pdhD- GCA 013314815.1 d Bacteria p Desulfobacterota c Syntrophobacteria o Syntrophobacterales
acsD-acsA-acsB-acsC-acsE- GCF 000014965.1 d Bacteria p Desulfobacterota c Syntrophobacteria o Syntrophobacterales
acsD-acsA-acsB-acsC-acsE- GCA 003566995.1 d Bacteria p Desulfobacterota c Syntrophobacteria o Syntrophobacterales
acsD-acsA-acsB- 1X acsC-acsE- GCA 012517315.1 d Bacteria p Desulfobacterota c Syntrophobacteria o Syntrophobacterales
acsD-acsA-acsB-acsC-acsE- GCA 012517775.1 d Bacteria p Desulfobacterota c Syntrophobacteria o Syntrophobacterales
acsD-acsA-acsB-acsC-acsE- GCA 002298995.1 d Bacteria p Desulfobacterota c Syntrophobacteria o Syntrophobacterales
acsD-acsA-acsB-acsC-acsE- GCF 900290365.1 d Bacteria p Desulfobacterota c Syntrophobacteria o Syntrophobacterales
acsD-acsA-acsB-acsC-acsE- GCA 003165235.1 d Bacteria p Desulfobacterota c Syntrophobacteria o Syntrophobacterales
acsV-cooC2-folD-acsD-acsA-acsB-acsC-acsE- GCA 001603955.1 d Bacteria p Desulfobacterota G c Syntrophorhabdia o Syntrophorhabdales
acsV-cooC2-folD-acsD-acsA-acsB-acsC- GCA 012719415.1 d Bacteria p Desulfobacterota G c Syntrophorhabdia o Syntrophorhabdales
acsD-acsA-acsB-acsC-acsE- GCA 002067235.1 d Bacteria p Desulfobacterota G c Syntrophorhabdia o Syntrophorhabdales
acsV-cooC2-folD-acsD-acsA-acsB-acsC-acsE- GCA 002305765.1 d Bacteria p Desulfobacterota G c Syntrophorhabdia o Syntrophorhabdales
acsV-cooC2-folD-acsD-acsA-acsB-acsC-acsE- GCA 903872255.1 d Bacteria p Desulfobacterota G c Syntrophorhabdia o Syntrophorhabdales
acsV-cooC2-folD-acsD-acsA-acsB-acsC-acsE- GCA 002408185.1 d Bacteria p Desulfobacterota G c Syntrophorhabdia o Syntrophorhabdales
acsV- 1X folD-acsD-acsA-acsB-acsC-acsE- GCA 002412715.1 d Bacteria p Desulfobacterota G c Syntrophorhabdia o Syntrophorhabdales
acsD-acsA- 1X acsB- 1X acsC-acsE- GCA 013824835.1 d Bacteria p Desulfobacterota G c Syntrophorhabdia o Syntrophorhabdales
acsV 1X acsD-acsA-acsB- GCA 903822335.1 d Bacteria p Desulfobacterota G c Syntrophorhabdia o Syntrophorhabdales
acsV-cooC2-acsD-acsA-acsB-acsC- GCA 013824645.1 d Bacteria p Desulfobacterota G c Syntrophorhabdia o Syntrophorhabdales
acsV-cooC2-folD-acsD-acsA-acsB-acsC-acsE- GCA 002428485.1 d Bacteria p Desulfobacterota G c Syntrophorhabdia o Syntrophorhabdales
acsV-cooC2-folD-acsD-acsA-acsB-acsC-acsE- GCA 002418945.1 d Bacteria p Desulfobacterota G c Syntrophorhabdia o Syntrophorhabdales
acsV-cooC2-folD-acsD-acsA-acsB-acsC-acsE- GCA 002478225.1 d Bacteria p Desulfobacterota G c Syntrophorhabdia o Syntrophorhabdales
acsB-acsC-acsE- GCA 002067975.1 d Bacteria p Desulfobacterota G c Syntrophorhabdia o Syntrophorhabdales
acsV-cooC2-folD-acsD-acsA-acsB-acsC-acsE- GCA 002402795.1 d Bacteria p Desulfobacterota G c Syntrophorhabdia o Syntrophorhabdales
acsV-cooC2-acsD-acsA-acsB-acsC-acsE- GCA 002067585.1 d Bacteria p Desulfobacterota G c Syntrophorhabdia o Syntrophorhabdales
acsV-cooC2- 2X folD- 1X acsD-acsA-acsB-acsC-acsE- GCF 000512235.1 d Bacteria p Desulfobacterota G c Syntrophorhabdia o Syntrophorhabdales
folD-acsD-acsA-acsB-acsC-acsE- GCA 002067165.1 d Bacteria p Desulfobacterota G c Syntrophorhabdia o Syntrophorhabdales
acsV-cooC2-folD-acsD-acsA-acsB- GCA 011388995.1 d Bacteria p Desulfobacterota G c Syntrophorhabdia o Syntrophorhabdales
acsV-cooC2-folD-acsD-acsA-acsB-acsC-acsE- GCA 002067405.1 d Bacteria p Desulfobacterota G c Syntrophorhabdia o Syntrophorhabdales
fhs- 2X acsD- 4X acsB-acsC-acsE- GCA 003165115.1 d Bacteria p Desulfobacterota G c Syntrophorhabdia o Syntrophorhabdales
fhs-cooC2-folD-acsD-acsA-acsB-acsC-acsE- 7X metV-metF- GCA 011773445.1 d Bacteria p Desulfobacterota c BSN033 o BSN033

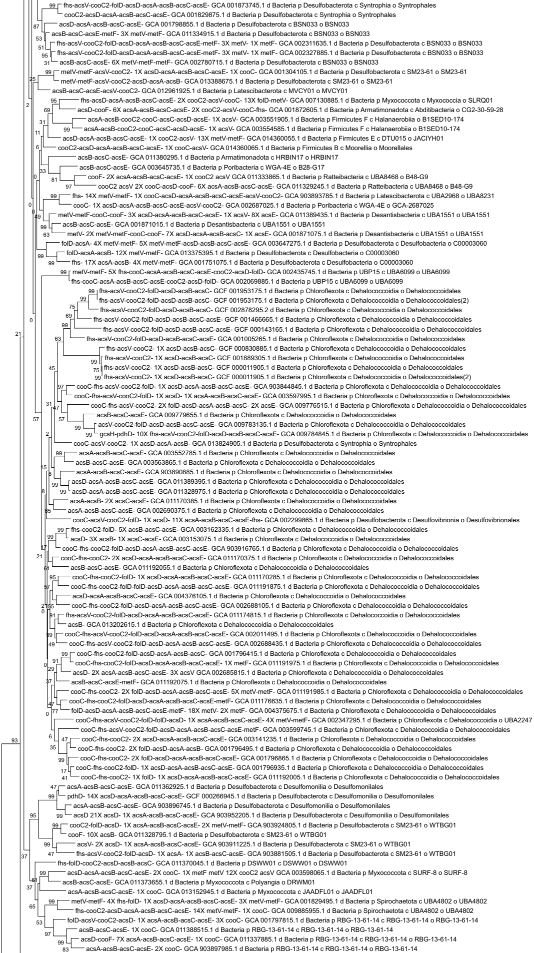
fhs-acsV-cooC2-folD-acsD-acsA-acsB-acsC-acsE- GCA 001873745.1 d Bacteria p Desulfobacterota c Syntrophia o Syntrophales
cooC2-acsD-acsA-acsB-acsC-acsE- GCA 001829875.1 d Bacteria p Desulfobacterota c Syntrophia o Syntrophales
acsD-acsA-acsB-acsC-acsE- GCA 001798855.1 d Bacteria p Desulfobacterota c BSN033 o BSN033
acsB-acsC-acsE-metF- 3X metV-metF- GCA 011334915.1 d Bacteria p Desulfobacterota c BSN033 o BSN033
fhs-acsV-cooC2-folD-acsD-acsA-acsB-acsC-acsE-metF- 3X metV- 1X metF- GCA 002311635.1 d Bacteria p Desulfobacterota c BSN033 o BSN033
fhs-acsV-cooC2-folD-acsD-acsA-acsB-acsC-acsE-metF- 3X metV- 1X metF- GCA 002327885.1 d Bacteria p Desulfobacterota c BSN033 o BSN033
acsB-acsC-acsE- 6X metV-metF-metF- GCA 002780715.1 d Bacteria p Desulfobacterota c BSN033 o BSN033
metV-metF-acsV-cooC2- 1X acsD-acsA-acsB-acsC-acsE- 1X cooC- GCA 001304105.1 d Bacteria p Desulfobacterota c SM23-61 o SM23-61
metV-metF-acsV-cooC2-acsD-acsA-acsB- GCA 013388675.1 d Bacteria p Desulfobacterota c SM23-61 o SM23-61
acsB-acsC-acsE-acsV-cooC2- GCA 012961925.1 d Bacteria p Latescibacterota c MVCY01 o MVCY01
fhs-acsD-acsA-acsB-acsC-acsE- 2X cooC2-acsV-cooC- 13X folD-metV- GCA 007130885.1 d Bacteria p Myxococcota c Myxococcia o SLRQ01
acsD-cooF- 6X acsA-acsB-acsC-acsE- 2X cooC2-acsV-cooC-fhs- GCA 001872605.1 d Bacteria p Armatimonadota c Abditibacteria o CG2-30-59-28
acsA-acsB-cooC2-cooC-acsC-acsD-acsE- 1X acsV- GCA 003551905.1 d Bacteria p Firmicutes F c Halanaerobiia o B1SED10-174
acsA-acsB-cooC2-cooC-acsC-acsD-acsE- 1X acsV- GCA 003554585.1 d Bacteria p Firmicutes F c Halanaerobiia o B1SED10-174
acsD-acsA-acsB-acsC-acsE- 1X cooC2-acsV- 13X metV-metF- GCA 014360055.1 d Bacteria p Firmicutes E c DTU015 o JACIYH01
cooC2-acsD-acsA-acsB-acsC-acsE- 1X cooC-acsV- GCA 014360065.1 d Bacteria p Firmicutes B c Moorellia o Moorellales
acsB-acsC-acsE- GCA 011380295.1 d Bacteria p Armatimonadota c HRBIN17 o HRBIN17
acsB-acsC-acsE- GCA 003645735.1 d Bacteria p Poribacteria c WGA-4E o B28-G17
cooF- 2X acsA-acsB-acsC-acsE- 1X cooC2 acsV GCA 011333865.1 d Bacteria p Ratteibacteria c UBA8468 o B48-G9
cooC2 acsV 2X cooC-acsD-cooF- 6X acsA-acsB-acsC-acsE- GCA 011329245.1 d Bacteria p Ratteibacteria c UBA8468 o B48-G9
fhs- 14X metV-metF- 1X cooC-acsD-acsA-acsB-acsC-acsE-acsV-cooC2- GCA 903893785.1 d Bacteria p Latescibacterota c UBA2968 o UBA8231
cooC- 1X acsD-acsA-acsB-acsC-acsE-acsV-cooC2- GCA 002687025.1 d Bacteria p Poribacteria c WGA-4E o GCA-2687025
metV-metF-cooC-cooF- 3X acsD-acsA-acsB-acsC-acsE- 1X acsV- 8X acsE- GCA 011389435.1 d Bacteria p Desantisbacteria c UBA1551 o UBA1551
acsB-acsC-acsE- GCA 001871015.1 d Bacteria p Desantisbacteria c UBA1551 o UBA1551
metV- 2X metV-metF-cooC-cooF- 7X acsD-acsA-acsB-acsC- 1X acsE- GCA 001871075.1 d Bacteria p Desantisbacteria c UBA1551 o UBA1551
folD-acsA- 4X metV-metF- 5X metV-metF-acsD-acsB-acsC-acsE- GCA 003647275.1 d Bacteria p Desulfobacterota c Desulfobacteria o C00003060
folD-acsA-acsB- 12X metV-metF- GCA 013375395.1 d Bacteria p Desulfobacterota c Desulfobacteria o C00003060
fhs- 17X acsA-acsB- 4X metV-metF- GCA 001751075.1 d Bacteria p Desulfobacterota c Desulfobacteria o C00003060
metV-metF- 5X fhs-cooC-acsA-acsB-acsC-acsE-cooC2-acsD-folD- GCA 002435745.1 d Bacteria p UBP15 c UBA6099 o UBA6099
fhs-cooC-acsA-acsB-acsC-acsE-cooC2-acsD-folD- GCA 002069885.1 d Bacteria p UBP15 c UBA6099 o UBA6099
fhs-acsV-cooC2-folD-acsD-acsB-acsC- GCF 001953175.1 d Bacteria p Chloroflexota c Dehalococcoidia o Dehalococcoidales
fhs-acsV-cooC2-folD-acsD-acsB-acsC- GCF 001953175.1 d Bacteria p Chloroflexota c Dehalococcoidia o Dehalococcoidales(2)
fhs-acsV-cooC2-folD-acsD-acsB-acsC- GCF 002878295.2 d Bacteria p Chloroflexota c Dehalococcoidia o Dehalococcoidales
fhs-acsV-cooC2-folD-acsD-acsB-acsC-acsE- GCF 001466665.1 d Bacteria p Chloroflexota c Dehalococcoidia o Dehalococcoidales
fhs-acsV-cooC2-folD-acsD-acsB-acsC-acsE- GCF 000143165.1 d Bacteria p Chloroflexota c Dehalococcoidia o Dehalococcoidales
fhs-acsV-cooC2-folD-acsD-acsB-acsC-acsE- GCA 001005265.1 d Bacteria p Chloroflexota c Dehalococcoidia o Dehalococcoidales
fhs-acsV-cooC2- 1X acsD-acsB-acsC- GCF 000830885.1 d Bacteria p Chloroflexota c Dehalococcoidia o Dehalococcoidales
fhs-acsV-cooC2- 1X acsD-acsB-acsC- GCF 001889305.1 d Bacteria p Chloroflexota c Dehalococcoidia o Dehalococcoidales
fhs-acsV-cooC2- 1X acsD-acsB-acsC- GCF 000011905.1 d Bacteria p Chloroflexota c Dehalococcoidia o Dehalococcoidales
fhs-acsV-cooC2- 1X acsD-acsB-acsC- GCF 000011905.1 d Bacteria p Chloroflexota c Dehalococcoidia o Dehalococcoidales(2)
cooC-fhs-acsV-cooC2-folD- 1X acsD-acsA-acsB-acsC-acsE- GCA 903844845.1 d Bacteria p Chloroflexota c Dehalococcoidia o Dehalococcoidales
cooC-fhs-acsV-cooC2-folD- 1X acsD- 1X acsA-acsB-acsC-acsE- GCA 003597995.1 d Bacteria p Chloroflexota c Dehalococcoidia o Dehalococcoidales
cooC-fhs-acsV-cooC2- 2X folD-acsD-acsA-acsB-acsC- 2X acsE- GCA 009776515.1 d Bacteria p Chloroflexota c Dehalococcoidia o Dehalococcoidales
acsB-acsC-acsE- GCA 009779655.1 d Bacteria p Chloroflexota c Dehalococcoidia o Dehalococcoidales
acsV-cooC2-folD-acsD-acsB-acsC-acsE- GCA 009783135.1 d Bacteria p Chloroflexota c Dehalococcoidia o Dehalococcoidales
gcsH-pdhD- 10X fhs-acsV-cooC2-folD-acsD-acsB-acsC-acsE- GCA 009784845.1 d Bacteria p Chloroflexota c Dehalococcoidia o Dehalococcoidales
cooC-acsV-cooC2- 1X acsD-acsA-acsB- GCA 013824905.1 d Bacteria p Desulfobacterota c Syntrophia o Syntrophales
acsA-acsB-acsC-acsE- GCA 003552785.1 d Bacteria p Chloroflexota c Dehalococcoidia o Dehalococcoidales
acsB-acsC-acsE- GCA 003563865.1 d Bacteria p Chloroflexota c Dehalococcoidia o Dehalococcoidales
acsA-acsB-acsC-acsE- GCA 903890885.1 d Bacteria p Chloroflexota c Dehalococcoidia o Dehalococcoidales
acsD-acsA-acsB-acsC-acsE- GCA 011389395.1 d Bacteria p Chloroflexota c Dehalococcoidia o Dehalococcoidales
acsD-acsA-acsB-acsC-acsE- GCA 011328975.1 d Bacteria p Chloroflexota c Dehalococcoidia o Dehalococcoidales
acsA-acsB- 2X acsC-acsE- GCA 011170385.1 d Bacteria p Chloroflexota c Dehalococcoidia o Dehalococcoidales
acsA-acsB-acsC-acsE- GCA 002690375.1 d Bacteria p Chloroflexota c Dehalococcoidia o Dehalococcoidales
cooC-acsV-cooC2-folD- 1X acsD- 11X acsA-acsB-acsC-acsE-fhs- GCA 002299865.1 d Bacteria p Desulfobacterota c Desulfovibrionia o Desulfovibrionales
fhs-cooC2-folD- 5X acsB-acsC-acsE- GCA 003162335.1 d Bacteria p Chloroflexota c Dehalococcoidia o Dehalococcoidales
acsD- 3X acsB- 1X acsC-acsE- GCA 003153075.1 d Bacteria p Chloroflexota c Dehalococcoidia o Dehalococcoidales
cooC-fhs-cooC2-folD-acsD-acsA-acsB-acsC-acsE- GCA 903916765.1 d Bacteria p Chloroflexota c Dehalococcoidia o Dehalococcoidales
cooC-fhs-cooC2- 2X acsD-acsA-acsB-acsC-acsE- GCA 011170375.1 d Bacteria p Chloroflexota c Dehalococcoidia o Dehalococcoidales
acsB-acsC-acsE- GCA 011192055.1 d Bacteria p Chloroflexota c Dehalococcoidia o Dehalococcoidales
cooC-fhs-cooC2-folD- 1X acsD-acsA-acsB-acsC-acsE- GCA 011170285.1 d Bacteria p Chloroflexota c Dehalococcoidia o Dehalococcoidales
cooC-fhs-cooC2-folD-folD-acsD-acsA-acsB-acsC-acsE- GCA 011191875.1 d Bacteria p Chloroflexota c Dehalococcoidia o Dehalococcoidales
acsD-acsA-acsB-acsC-acsE- GCA 004376105.1 d Bacteria p Chloroflexota c Dehalococcoidia o Dehalococcoidales
cooC-fhs-cooC2-folD-acsD-acsA-acsB-acsC-acsE- GCA 002688105.1 d Bacteria p Chloroflexota c Dehalococcoidia o Dehalococcoidales
fhs-acsV-cooC2-folD-acsD-acsA-acsB-acsC-acsE- GCA 011174815.1 d Bacteria p Chloroflexota c Dehalococcoidia o Dehalococcoidales
acsB- GCA 013202615.1 d Bacteria p Chloroflexota c Dehalococcoidia o Dehalococcoidales
cooC-fhs-acsV-cooC2-folD-acsD-acsA-acsB-acsC-acsE- GCA 002011495.1 d Bacteria p Chloroflexota c Dehalococcoidia o Dehalococcoidales
cooC-fhs-acsV-cooC2-folD-acsD-acsA-acsB-acsC-acsE- GCA 002688435.1 d Bacteria p Chloroflexota c Dehalococcoidia o Dehalococcoidales
cooC-fhs-cooC2-folD-acsD-acsA-acsB-acsC- GCA 001796415.1 d Bacteria p Chloroflexota c Dehalococcoidia o Dehalococcoidales
cooC-fhs-cooC2-folD-acsD-acsA-acsB-acsC-acsE- 1X metF- GCA 011191975.1 d Bacteria p Chloroflexota c Dehalococcoidia o Dehalococcoidales
acsD- 2X acsA-acsB-acsC-acsE- 3X acsV GCA 002685815.1 d Bacteria p Chloroflexota c Dehalococcoidia o Dehalococcoidales
acsB-acsC-acsE-metF- GCA 011192075.1 d Bacteria p Chloroflexota c Dehalococcoidia o Dehalococcoidales
cooC-fhs-cooC2- 2X folD-acsD-acsA-acsB-acsC-acsE- 5X metV-metF- GCA 011191985.1 d Bacteria p Chloroflexota c Dehalococcoidia o Dehalococcoidales
cooC-fhs-cooC2-folD-acsD-acsA-acsB-acsC-acsE-metF- GCA 011176635.1 d Bacteria p Chloroflexota c Dehalococcoidia o Dehalococcoidales
folD-acsD-acsA-acsB-acsC-acsE-metF- 18X metV- 2X metF- GCA 004375675.1 d Bacteria p Chloroflexota c Dehalococcoidia o Dehalococcoidales
cooC-fhs-acsV-cooC2-folD-folD-acsD- 1X acsA-acsB-acsC-acsE- 4X metV-metF- GCA 002347295.1 d Bacteria p Chloroflexota c Dehalococcoidia o UBA2247
cooC-fhs-acsV-cooC2-folD-acsD-acsA-acsB-acsC-acsE-metF- GCA 003599745.1 d Bacteria p Chloroflexota c Dehalococcoidia o Dehalococcoidales
cooC-fhs-cooC2- 2X acsD-acsA-acsB-acsC-acsE- GCA 003141235.1 d Bacteria p Chloroflexota c Dehalococcoidia o Dehalococcoidales
cooC-fhs-cooC2- 2X folD-acsD-acsA-acsB- GCA 001796495.1 d Bacteria p Chloroflexota c Dehalococcoidia o Dehalococcoidales
cooC-fhs-cooC2- 2X folD-acsD-acsA-acsB-acsC-acsE- GCA 001796865.1 d Bacteria p Chloroflexota c Dehalococcoidia o Dehalococcoidales
cooC-fhs-cooC2-folD- 1X acsD-acsA-acsB-acsC-acsE- GCA 001796935.1 d Bacteria p Chloroflexota c Dehalococcoidia o Dehalococcoidales
cooC-fhs-cooC2- 1X folD- 1X acsD-acsA-acsB-acsC-acsE- GCA 011192005.1 d Bacteria p Chloroflexota c Dehalococcoidia o Dehalococcoidales
acsA-acsB-acsC-acsE- GCA 011362925.1 d Bacteria p Desulfobacterota c Desulfomonilia o Desulfomonilales
pdhD- 14X acsD-acsA-acsB-acsC-acsE- GCF 000266945.1 d Bacteria p Desulfobacterota c Desulfomonilia o Desulfomonilales
acsA-acsB-acsC-acsE- GCA 903896745.1 d Bacteria p Desulfobacterota c Desulfomonilia o Desulfomonilales
acsD 21X acsD- 1X acsA-acsB-acsC-acsE- GCA 903952205.1 d Bacteria p Desulfobacterota c Desulfomonilia o Desulfomonilales
cooC2-folD-acsD- 1X acsA-acsB-acsC-acsE- 2X metV-metF- GCA 903924805.1 d Bacteria p Desulfobacterota c SM23-61 o WTBG01
cooF- 10X acsB- GCA 011328795.1 d Bacteria p Desulfobacterota c SM23-61 o WTBG01
acsV- 2X acsD- 1X acsA-acsB-acsC-acsE- GCA 903911225.1 d Bacteria p Desulfobacterota c SM23-61 o WTBG01
fhs-acsV-cooC2-folD-acsD- 1X acsA- 1X acsB-acsC-acsE- GCA 903881505.1 d Bacteria p Desulfobacterota c SM23-61 o WTBG01
fhs-folD-cooC2-acsD-acsB-acsC- GCA 011370045.1 d Bacteria p DSWW01 c DSWW01 o DSWW01
acsD-acsA-acsB-acsC-acsE- 2X cooC- 1X metF metV 12X cooC2 acsV GCA 003598065.1 d Bacteria p Myxococcota c SURF-8 o SURF-8
acsB-acsC-acsE- GCA 011373655.1 d Bacteria p Myxococcota c Polyangia o DRWM01
acsA-acsB-acsC-acsE- 1X cooC- GCA 013152945.1 d Bacteria p Myxococcota c JAADFL01 o JAADFL01
metV-metF- 4X fhs-folD- 1X acsD-acsA-acsB-acsC-acsE- 3X metV-metF- GCA 001829495.1 d Bacteria p Spirochaetota c UBA4802 o UBA4802
fhs-cooC2-acsD-acsA-acsB-acsC-acsE- 14X metV-metF- 1X cooC- GCA 009885955.1 d Bacteria p Spirochaetota c UBA4802 o UBA4802
folD-acsV-cooC2-acsD- 1X acsA-acsB-acsC-acsE- 3X cooC- GCA 001797815.1 d Bacteria p RBG-13-61-14 c RBG-13-61-14 o RBG-13-61-14
acsB-acsC-acsE- 1X cooC- GCA 011388515.1 d Bacteria p RBG-13-61-14 c RBG-13-61-14 o RBG-13-61-14
acsD-cooF- 7X acsA-acsB-acsC-acsE- 1X cooC- GCA 011337885.1 d Bacteria p RBG-13-61-14 c RBG-13-61-14 o RBG-13-61-14
acsA-acsB-acsC-acsE- 2X cooC- GCA 903897985.1 d Bacteria p RBG-13-61-14 c RBG-13-61-14 o RBG-13-61-14

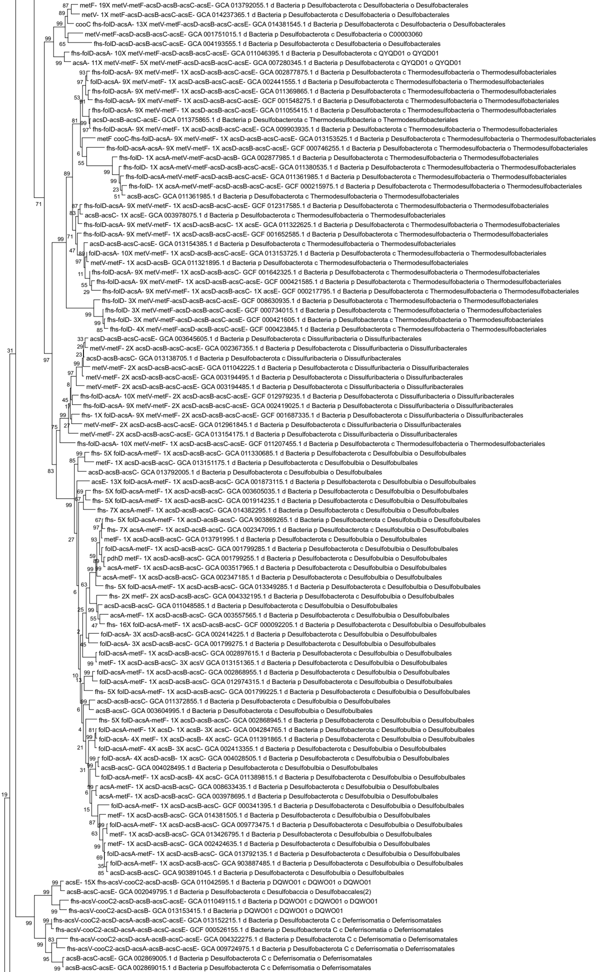
metF- 19X metV-metF-acsD-acsB-acsC-acsE- GCA 013792055.1 d Bacteria p Desulfobacterota c Desulfobacteria o Desulfobacterales
metV- 1X metF-acsD-acsB-acsC-acsE- GCA 014237365.1 d Bacteria p Desulfobacterota c Desulfobacteria o Desulfobacterales
cooC fhs-folD-acsA- 13X metV-metF-acsD-acsB-acsC-acsE- GCA 014381545.1 d Bacteria p Desulfobacterota c Desulfobacteria o Desulfobacterales
metV-metF-acsD-acsB-acsC-acsE- GCA 001751015.1 d Bacteria p Desulfobacterota c Desulfobacteria o C00003060
fhs-folD-acsD-acsB-acsC-acsE- GCA 004193555.1 d Bacteria p Desulfobacterota c Desulfobacteria o Desulfobacterales
fhs-folD-acsA- 10X metV-metF-acsD-acsB-acsC-acsE- GCA 011046395.1 d Bacteria p Desulfobacterota c QYQD01 o QYQD01
acsA- 11X metV-metF- 5X metV-metF-acsD-acsB-acsC-acsE- GCA 007280345.1 d Bacteria p Desulfobacterota c QYQD01 o QYQD01
fhs-folD-acsA- 9X metV-metF- 1X acsD-acsB-acsC-acsE- GCA 002877875.1 d Bacteria p Desulfobacterota c Thermodesulfobacteria o Thermodesulfobacteriales
folD-acsA- 9X metV-metF- 1X acsD-acsB-acsC-acsE- GCA 002441555.1 d Bacteria p Desulfobacterota c Thermodesulfobacteria o Thermodesulfobacteriales
fhs-folD-acsA- 9X metV-metF- 1X acsD-acsB-acsC-acsE- GCA 011369865.1 d Bacteria p Desulfobacterota c Thermodesulfobacteria o Thermodesulfobacteriales
fhs-folD-acsA- 9X metV-metF- 1X acsD-acsB-acsC-acsE- GCF 001548275.1 d Bacteria p Desulfobacterota c Thermodesulfobacteria o Thermodesulfobacteriales
fhs-folD-acsA- 9X metV-metF- 1X acsD-acsB-acsC-acsE- GCA 011055415.1 d Bacteria p Desulfobacterota c Thermodesulfobacteria o Thermodesulfobacteriales
acsD-acsB-acsC-acsE- GCA 011375865.1 d Bacteria p Desulfobacterota c Thermodesulfobacteria o Thermodesulfobacteriales
fhs-folD-acsA- 9X metV-metF- 1X acsD-acsB-acsC-acsE- GCA 009903935.1 d Bacteria p Desulfobacterota c Thermodesulfobacteria o Thermodesulfobacteriales
metF cooC-fhs-folD-acsA- 9X metV-metF- 1X acsD-acsB-acsC-acsE- GCA 013153525.1 d Bacteria p Desulfobacterota c Thermodesulfobacteria o Thermodesulfobacteriales
fhs-folD-acsA-acsA- 9X metV-metF- 1X acsD-acsB-acsC-acsE- GCF 000746255.1 d Bacteria p Desulfobacterota c Thermodesulfobacteria o Thermodesulfobacteriales
fhs-folD- 1X acsA-metV-metF-acsD-acsB- GCA 002877985.1 d Bacteria p Desulfobacterota c Thermodesulfobacteria o Thermodesulfobacteriales
fhs-folD- 1X acsA-metV-metF-acsD-acsB-acsC-acsE- GCA 011380535.1 d Bacteria p Desulfobacterota c Thermodesulfobacteria o Thermodesulfobacteriales
fhs-folD-acsA-metV-metF-acsD-acsB-acsC-acsE- GCA 011361985.1 d Bacteria p Desulfobacterota c Thermodesulfobacteria o Thermodesulfobacteriales
fhs-folD- 1X acsA-metV-metF-acsD-acsB-acsC-acsE- GCF 000215975.1 d Bacteria p Desulfobacterota c Thermodesulfobacteria o Thermodesulfobacteriales
acsB-acsC- GCA 011361985.1 d Bacteria p Desulfobacterota c Thermodesulfobacteria o Thermodesulfobacteriales
fhs-folD-acsA- 9X metV-metF- 1X acsD-acsB-acsC-acsE- GCF 012317585.1 d Bacteria p Desulfobacterota c Thermodesulfobacteria o Thermodesulfobacteriales
acsB-acsC- 1X acsE- GCA 003978075.1 d Bacteria p Desulfobacterota c Thermodesulfobacteria o Thermodesulfobacteriales
fhs-folD-acsA- 9X metV-metF- 1X acsD-acsB-acsC- 1X acsE- GCA 011322625.1 d Bacteria p Desulfobacterota c Thermodesulfobacteria o Thermodesulfobacteriales
fhs-folD-acsA- 9X metV-metF- 1X acsD-acsB-acsC-acsE- GCF 001652585.1 d Bacteria p Desulfobacterota c Thermodesulfobacteria o Thermodesulfobacteriales
acsD-acsB-acsC-acsE- GCA 013154385.1 d Bacteria p Desulfobacterota c Thermodesulfobacteria o Thermodesulfobacteriales
folD-acsA- 10X metV-metF- 1X acsD-acsB-acsC-acsE- GCA 013153725.1 d Bacteria p Desulfobacterota c Thermodesulfobacteria o Thermodesulfobacteriales
metV-metF- 1X acsD-acsB- GCA 011321895.1 d Bacteria p Desulfobacterota c Thermodesulfobacteria o Thermodesulfobacteriales
fhs-folD-acsA- 9X metV-metF- 1X acsD-acsB-acsC- GCF 001642325.1 d Bacteria p Desulfobacterota c Thermodesulfobacteria o Thermodesulfobacteriales
fhs-folD-acsA- 9X metV-metF- 1X acsD-acsB-acsC-acsE- GCF 000421585.1 d Bacteria p Desulfobacterota c Thermodesulfobacteria o Thermodesulfobacteriales
fhs-folD-acsA- 9X metV-metF- 1X acsD-acsB-acsC- 1X acsE- GCF 000217795.1 d Bacteria p Desulfobacterota c Thermodesulfobacteria o Thermodesulfobacteriales
fhs-folD- 3X metV-metF-acsD-acsB-acsC-acsE- GCF 008630935.1 d Bacteria p Desulfobacterota c Thermodesulfobacteria o Thermodesulfobacteriales
fhs-folD- 3X metV-metF-acsD-acsB-acsC-acsE- GCF 000734015.1 d Bacteria p Desulfobacterota c Thermodesulfobacteria o Thermodesulfobacteriales
fhs-folD- 3X metV-metF-acsD-acsB-acsC-acsE- GCF 000421605.1 d Bacteria p Desulfobacterota c Thermodesulfobacteria o Thermodesulfobacteriales
fhs-folD- 4X metV-metF-acsD-acsB-acsC-acsE- GCF 000423845.1 d Bacteria p Desulfobacterota c Thermodesulfobacteria o Thermodesulfobacteriales
acsD-acsB-acsC-acsE- GCA 003645605.1 d Bacteria p Desulfobacterota c Dissulfuribacteria o Dissulfuribacterales
metV-metF- 2X acsD-acsB-acsC-acsE- GCA 002367355.1 d Bacteria p Desulfobacterota c Dissulfuribacteria o Dissulfuribacterales
acsD-acsB-acsC- GCA 013138705.1 d Bacteria p Desulfobacterota c Dissulfuribacteria o Dissulfuribacterales
metV-metF- 2X acsD-acsB-acsC-acsE- GCA 011042225.1 d Bacteria p Desulfobacterota c Dissulfuribacteria o Dissulfuribacterales
metV-metF- 2X acsD-acsB-acsC-acsE- GCA 003194495.1 d Bacteria p Desulfobacterota c Dissulfuribacteria o Dissulfuribacterales
metV-metF- 2X acsD-acsB-acsC-acsE- GCA 003194485.1 d Bacteria p Desulfobacterota c Dissulfuribacteria o Dissulfuribacterales
fhs-folD-acsA- 10X metV-metF- 2X acsD-acsB-acsC-acsE- GCF 012979235.1 d Bacteria p Desulfobacterota c Dissulfuribacteria o Dissulfuribacterales
fhs-folD-acsA- 9X metV-metF- 2X acsD-acsB-acsC-acsE- GCA 002419025.1 d Bacteria p Desulfobacterota c Dissulfuribacteria o Dissulfuribacterales
fhs- 1X folD-acsA- 9X metV-metF- 2X acsD-acsB-acsC-acsE- GCF 001687335.1 d Bacteria p Desulfobacterota c Dissulfuribacteria o Dissulfuribacterales
metV-metF- 2X acsD-acsB-acsC-acsE- GCA 012961845.1 d Bacteria p Desulfobacterota c Dissulfuribacteria o Dissulfuribacterales
metV-metF- 2X acsD-acsB-acsC-acsE- GCA 013154175.1 d Bacteria p Desulfobacterota c Dissulfuribacteria o Dissulfuribacterales
fhs-folD-acsA- 10X metV-metF- 1X acsD-acsB-acsC-acsE- GCF 011207455.1 d Bacteria p Desulfobacterota c Thermodesulfobacteria o Thermodesulfobacteriales
fhs- 5X folD-acsA-metF- 1X acsD-acsB-acsC- GCA 011330685.1 d Bacteria p Desulfobacterota c Desulfobulbia o Desulfobulbales
metF- 1X acsD-acsB-acsC- GCA 013151175.1 d Bacteria p Desulfobacterota c Desulfobulbia o Desulfobulbales
acsD-acsB-acsC- GCA 013792005.1 d Bacteria p Desulfobacterota c Desulfobulbia o Desulfobulbales
acsE- 13X folD-acsA-metF- 1X acsD-acsB-acsC- GCA 001873115.1 d Bacteria p Desulfobacterota c Desulfobulbia o Desulfobulbales
fhs- 5X folD-acsA-metF- 1X acsD-acsB-acsC- GCA 003605035.1 d Bacteria p Desulfobacterota c Desulfobulbia o Desulfobulbales
fhs- 5X folD-acsA-metF- 1X acsD-acsB-acsC- GCA 001914235.1 d Bacteria p Desulfobacterota c Desulfobulbia o Desulfobulbales
fhs- 7X acsA-metF- 1X acsD-acsB-acsC- GCA 014382295.1 d Bacteria p Desulfobacterota c Desulfobulbia o Desulfobulbales
fhs- 5X folD-acsA-metF- 1X acsD-acsB-acsC- GCA 903869265.1 d Bacteria p Desulfobacterota c Desulfobulbia o Desulfobulbales
fhs- 7X acsA-metF- 1X acsD-acsB-acsC- GCA 002347095.1 d Bacteria p Desulfobacterota c Desulfobulbia o Desulfobulbales
metF- 1X acsD-acsB-acsC- GCA 013791995.1 d Bacteria p Desulfobacterota c Desulfobulbia o Desulfobulbales
folD-acsA-metF- 1X acsD-acsB-acsC- GCA 001799285.1 d Bacteria p Desulfobacterota c Desulfobulbia o Desulfobulbales
pdhD metF- 1X acsD-acsB-acsC- GCA 001799255.1 d Bacteria p Desulfobacterota c Desulfobulbia o Desulfobulbales
acsA-metF- 1X acsD-acsB-acsC- GCA 003517965.1 d Bacteria p Desulfobacterota c Desulfobulbia o Desulfobulbales
acsA-metF- 1X acsD-acsB-acsC- GCA 002347185.1 d Bacteria p Desulfobacterota c Desulfobulbia o Desulfobulbales
fhs- 5X folD-acsA-metF- 1X acsD-acsB-acsC- GCA 013349285.1 d Bacteria p Desulfobacterota c Desulfobulbia o Desulfobulbales
fhs- 2X metF- 2X acsD-acsB-acsC- GCA 004332195.1 d Bacteria p Desulfobacterota c Desulfobulbia o Desulfobulbales
acsD-acsB-acsC- GCA 011048585.1 d Bacteria p Desulfobacterota c Desulfobulbia o Desulfobulbales
acsA-metF- 1X acsD-acsB-acsC- GCA 003557565.1 d Bacteria p Desulfobacterota c Desulfobulbia o Desulfobulbales
fhs- 16X folD-acsA-metF- 1X acsD-acsB-acsC- GCF 000092205.1 d Bacteria p Desulfobacterota c Desulfobulbia o Desulfobulbales
folD-acsA- 3X acsD-acsB-acsC- GCA 002414225.1 d Bacteria p Desulfobacterota c Desulfobulbia o Desulfobulbales
folD-acsA- 3X acsD-acsB-acsC- GCA 001799275.1 d Bacteria p Desulfobacterota c Desulfobulbia o Desulfobulbales
folD-acsA-metF- 1X acsD-acsB-acsC- GCA 002897615.1 d Bacteria p Desulfobacterota c Desulfobulbia o Desulfobulbales
metF- 1X acsD-acsB-acsC- 3X acsV GCA 013151365.1 d Bacteria p Desulfobacterota c Desulfobulbia o Desulfobulbales
folD-acsA-metF- 1X acsD-acsB-acsC- GCA 002868955.1 d Bacteria p Desulfobacterota c Desulfobulbia o Desulfobulbales
folD-acsA-metF- 1X acsD-acsB-acsC- GCA 012974315.1 d Bacteria p Desulfobacterota c Desulfobulbia o Desulfobulbales
fhs- 5X folD-acsA-metF- 1X acsD-acsB-acsC- GCA 001799225.1 d Bacteria p Desulfobacterota c Desulfobulbia o Desulfobulbales
acsD-acsB-acsC- GCA 011372855.1 d Bacteria p Desulfobacterota c Desulfobulbia o Desulfobulbales
acsB-acsC- GCA 003604995.1 d Bacteria p Desulfobacterota c Desulfobulbia o Desulfobulbales
fhs- 5X folD-acsA-metF- 1X acsD-acsB-acsC- GCA 002868945.1 d Bacteria p Desulfobacterota c Desulfobulbia o Desulfobulbales
folD-acsA-metF- 1X acsD- 1X acsB- 3X acsC- GCA 004284765.1 d Bacteria p Desulfobacterota c Desulfobulbia o Desulfobulbales
folD-acsA- 4X metF- 1X acsD-acsB- 4X acsC- GCA 011391865.1 d Bacteria p Desulfobacterota c Desulfobulbia o Desulfobulbales
folD-acsA-metF- 4X acsB- 3X acsC- GCA 002413355.1 d Bacteria p Desulfobacterota c Desulfobulbia o Desulfobulbales
folD-acsA- 4X acsD-acsB- 1X acsC- GCA 004028505.1 d Bacteria p Desulfobacterota c Desulfobulbia o Desulfobulbales
acsB-acsC- GCA 004028495.1 d Bacteria p Desulfobacterota c Desulfobulbia o Desulfobulbales
folD-acsA-metF- 1X acsD-acsB- 4X acsC- GCA 011389815.1 d Bacteria p Desulfobacterota c Desulfobulbia o Desulfobulbales
acsA-metF- 1X acsD-acsB-acsC- GCA 008633435.1 d Bacteria p Desulfobacterota c Desulfobulbia o Desulfobulbales
acsA-metF- 1X acsD-acsB-acsC- GCA 003978695.1 d Bacteria p Desulfobacterota c Desulfobulbia o Desulfobulbales
folD-acsA-metF- 1X acsD-acsB-acsC- GCF 000341395.1 d Bacteria p Desulfobacterota c Desulfobulbia o Desulfobulbales
metF- 1X acsD-acsB-acsC- GCA 014381505.1 d Bacteria p Desulfobacterota c Desulfobulbia o Desulfobulbales
folD-acsA-metF- 1X acsD-acsB-acsC- GCA 009773475.1 d Bacteria p Desulfobacterota c Desulfobulbia o Desulfobulbales
metF- 1X acsD-acsB-acsC- GCA 013426795.1 d Bacteria p Desulfobacterota c Desulfobulbia o Desulfobulbales
metF- 1X acsD-acsB-acsC- GCA 002424635.1 d Bacteria p Desulfobacterota c Desulfobulbia o Desulfobulbales
folD-acsA-metF- 1X acsD-acsB-acsC- GCA 013792135.1 d Bacteria p Desulfobacterota c Desulfobulbia o Desulfobulbales
folD-acsA-metF- 1X acsD-acsB-acsC- GCA 903887485.1 d Bacteria p Desulfobacterota c Desulfobulbia o Desulfobulbales
acsD-acsB-acsC- GCA 903891045.1 d Bacteria p Desulfobacterota c Desulfobulbia o Desulfobulbales
acsE- 15X fhs-acsV-cooC2-acsD-acsB- GCA 011042595.1 d Bacteria p DQWO01 c DQWO01 o DQWO01
acsB-acsC-acsE- GCA 002049795.1 d Bacteria p Desulfobacterota c Desulfobaccia o Desulfobaccales(2)
fhs-acsV-cooC2-acsD-acsB-acsC-acsE- GCA 011049115.1 d Bacteria p DQWO01 c DQWO01 o DQWO01
fhs-acsV-cooC2-acsD-acsB- GCA 013153415.1 d Bacteria p DQWO01 c DQWO01 o DQWO01
fhs-acsV-cooC2-acsD-acsA-acsB-acsC-acsE- GCA 013152215.1 d Bacteria p Desulfobacterota C c Deferrisomatia o Deferrisomatales
fhs-acsV-cooC2-acsD-acsA-acsB-acsC-acsE- GCF 000526155.1 d Bacteria p Desulfobacterota C c Deferrisomatia o Deferrisomatales
fhs-acsV-cooC2-acsD-acsA-acsB-acsC-acsE- GCA 004322275.1 d Bacteria p Desulfobacterota C c Deferrisomatia o Deferrisomatales
fhs-acsV-cooC2-acsD-acsA-acsB-acsC-acsE- GCA 009724975.1 d Bacteria p Desulfobacterota C c Deferrisomatia o Deferrisomatales
acsB-acsC-acsE- GCA 002869005.1 d Bacteria p Desulfobacterota C c Deferrisomatia o Deferrisomatales
acsB-acsC-acsE- GCA 002869015.1 d Bacteria p Desulfobacterota C c Deferrisomatia o Deferrisomatales

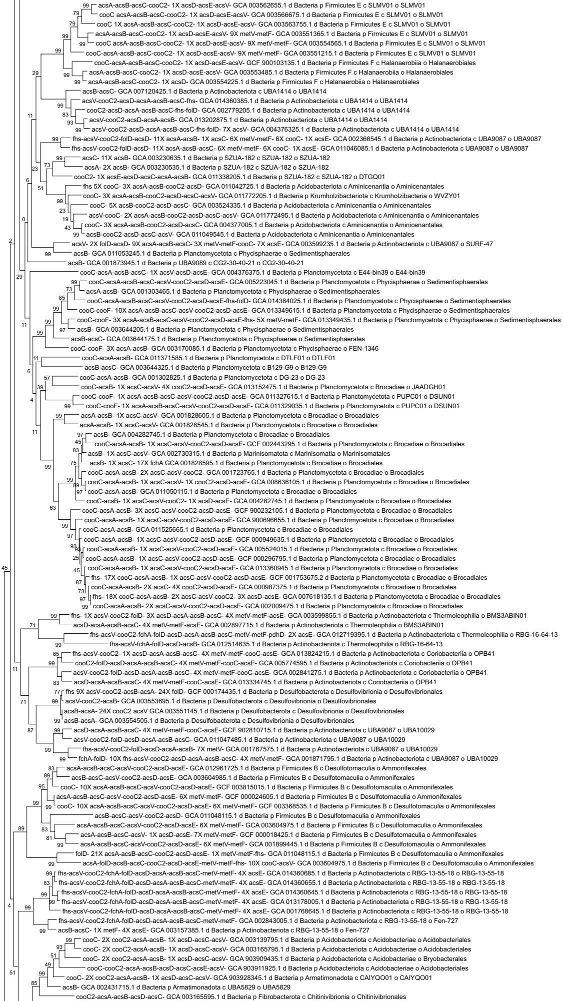

acsA-acsB-acsC-cooC2- 1X acsD-acsE-acsV- GCA 003562655.1 d Bacteria p Firmicutes E c SLMV01 o SLMV01
cooC acsA-acsB-acsC-cooC2- 1X acsD-acsE-acsV- GCA 003566675.1 d Bacteria p Firmicutes E c SLMV01 o SLMV01
cooC 1X acsA-acsB-acsC-cooC2- 1X acsD-acsE-acsV- GCA 003563755.1 d Bacteria p Firmicutes E c SLMV01 o SLMV01
acsA-acsB-acsC-cooC2- 1X acsD-acsE-acsV- 9X metV-metF- GCA 003551365.1 d Bacteria p Firmicutes E c SLMV01 o SLMV01
cooC acsA-acsB-acsC-cooC2- 1X acsD-acsE-acsV- 9X metV-metF- GCA 003554565.1 d Bacteria p Firmicutes E c SLMV01 o SLMV01
cooC-acsA-acsB-acsC-cooC2- 1X acsD-acsE-acsV- 9X metV-metF- GCA 003551215.1 d Bacteria p Firmicutes E c SLMV01 o SLMV01
cooC-acsA-acsB-acsC-cooC2- 1X acsD-acsE-acsV- GCF 900103135.1 d Bacteria p Firmicutes F c Halanaerobiia o Halanaerobiales
acsA-acsB-acsC-cooC2- 1X acsD-acsE-acsV- GCA 003553485.1 d Bacteria p Firmicutes F c Halanaerobiia o Halanaerobiales
acsA-acsB-acsC-cooC2- 1X acsD- GCA 003554225.1 d Bacteria p Firmicutes F c Halanaerobiia o Halanaerobiales
acsB-acsC- GCA 007120425.1 d Bacteria p Actinobacteriota c UBA1414 o UBA1414
acsV-cooC2-acsD-acsA-acsB-acsC-fhs- GCA 014360385.1 d Bacteria p Actinobacteriota c UBA1414 o UBA1414
cooC2-acsD-acsA-acsB-acsC-fhs-folD- GCA 002779205.1 d Bacteria p Actinobacteriota c UBA1414 o UBA1414
acsV-cooC2-acsD-acsA-acsB- GCA 013202875.1 d Bacteria p Actinobacteriota c UBA1414 o UBA1414
acsV-cooC2-acsD-acsA-acsB-acsC-fhs-folD- 7X acsV- GCA 004376325.1 d Bacteria p Actinobacteriota c UBA1414 o UBA1414
fhs-acsV-cooC2-folD-acsD- 11X acsA-acsB- 1X acsC- 6X metV-metF- 6X cooC- 1X acsE- GCA 002366545.1 d Bacteria p Actinobacteriota c UBA9087 o UBA9087
fhs-acsV-cooC2-folD-acsD- 11X acsA-acsB-acsC- 6X metV-metF- 6X cooC- 1X acsE- GCA 011046085.1 d Bacteria p Actinobacteriota c UBA9087 o UBA9087
acsC- 11X acsB- GCA 003230635.1 d Bacteria p SZUA-182 c SZUA-182 o SZUA-182
acsA- 2X acsB- GCA 003230535.1 d Bacteria p SZUA-182 c SZUA-182 o SZUA-182
cooC2- 1X acsE-acsD-acsC-acsA-acsB- GCA 011338205.1 d Bacteria p SZUA-182 c SZUA-182 o DTGQ01
fhs 5X cooC- 3X acsA-acsB-cooC2-acsD- GCA 011042725.1 d Bacteria p Acidobacteriota c Aminicenantia o Aminicenantales
cooC- 3X acsA-acsB-cooC2-acsD-acsC-acsV- GCA 011772205.1 d Bacteria p Krumholzibacteriota c Krumholzibacteria o WVZY01
cooC- 5X acsB-cooC2-acsD-acsC- GCA 003524335.1 d Bacteria p Acidobacteriota c Aminicenantia o Aminicenantales
acsV-cooC- 2X acsA-acsB-cooC2-acsD-acsC-acsV- GCA 011772495.1 d Bacteria p Acidobacteriota c Aminicenantia o Aminicenantales
cooC- 3X acsA-acsB-cooC2-acsD-acsC- GCA 004377005.1 d Bacteria p Acidobacteriota c Aminicenantia o Aminicenantales
acsB-cooC2-acsD-acsC-acsV- GCA 011049545.1 d Bacteria p Acidobacteriota c Aminicenantia o Aminicenantales
acsV- 2X folD-acsD- 9X acsA-acsB-acsC- 3X metV-metF-cooC- 7X acsE- GCA 003599235.1 d Bacteria p Actinobacteriota c UBA9087 o SURF-47
acsB- GCA 011053245.1 d Bacteria p Planctomycetota c Phycisphaerae o Sedimentisphaerales
acsB- GCA 001873945.1 d Bacteria p UBA9089 c CG2-30-40-21 o CG2-30-40-21
cooC-acsA-acsB-acsC- 1X acsV-acsD-acsE- GCA 004376375.1 d Bacteria p Planctomycetota c E44-bin39 o E44-bin39
cooC-acsA-acsB-acsC-acsV-cooC2-acsD-acsE- GCA 005223045.1 d Bacteria p Planctomycetota c Phycisphaerae o Sedimentisphaerales
acsA-acsB- GCA 001303465.1 d Bacteria p Planctomycetota c Phycisphaerae o Sedimentisphaerales
cooC-acsA-acsB-acsC-acsV-cooC2-acsD-acsE-fhs-folD- GCA 014384025.1 d Bacteria p Planctomycetota c Phycisphaerae o Sedimentisphaerales
cooC-cooF- 10X acsA-acsB-acsC-acsV-cooC2-acsD-acsE- GCA 013349615.1 d Bacteria p Planctomycetota c Phycisphaerae o Sedimentisphaerales
cooC-cooF- 3X acsA-acsB-acsC-acsV-cooC2-acsD-acsE-fhs- 5X metV-metF- GCA 013349435.1 d Bacteria p Planctomycetota c Phycisphaerae o Sedimentisphaerales
acsB- GCA 003644205.1 d Bacteria p Planctomycetota c Phycisphaerae o Sedimentisphaerales
acsB-acsC- GCA 003644175.1 d Bacteria p Planctomycetota c Phycisphaerae o Sedimentisphaerales
cooC-cooF- 3X acsA-acsB- GCA 003170085.1 d Bacteria p Planctomycetota c Phycisphaerae o FEN-1346
cooC-acsA-acsB- GCA 011371585.1 d Bacteria p Planctomycetota c DTLF01 o DTLF01
acsB-acsC- GCA 003644325.1 d Bacteria p Planctomycetota c B129-G9 o B129-G9
cooC-acsA-acsB- GCA 001302825.1 d Bacteria p Planctomycetota c DG-23 o DG-23
cooC-acsB- 1X acsC-acsV- 4X cooC2-acsD-acsE- GCA 013152475.1 d Bacteria p Planctomycetota c Brocadiae o JAADGH01
cooC-cooF- 1X acsA-acsB-acsC-acsV-cooC2-acsD-acsE- GCA 011327615.1 d Bacteria p Planctomycetota c PUPC01 o DSUN01
cooC-cooF- 1X acsA-acsB-acsC-acsV-cooC2-acsD-acsE- GCA 011329035.1 d Bacteria p Planctomycetota c PUPC01 o DSUN01
acsA-acsB- 1X acsC-acsV- GCA 001828605.1 d Bacteria p Planctomycetota c Brocadiae o Brocadiales
acsA-acsB- 1X acsC-acsV- GCA 001828545.1 d Bacteria p Planctomycetota c Brocadiae o Brocadiales
acsB- GCA 004282745.1 d Bacteria p Planctomycetota c Brocadiae o Brocadiales
cooC-acsA-acsB- 1X acsC-acsV-cooC2-acsD-acsE- GCF 002443295.1 d Bacteria p Planctomycetota c Brocadiae o Brocadiales
acsB- 1X acsC-acsV- GCA 002730315.1 d Bacteria p Marinisomatota c Marinisomatia o Marinisomatales
acsB- 1X acsC- 17X fchA GCA 001828595.1 d Bacteria p Planctomycetota c Brocadiae o Brocadiales
cooC-acsA-acsB- 2X acsC-acsV-cooC2- GCA 001723765.1 d Bacteria p Planctomycetota c Brocadiae o Brocadiales
cooC-acsA-acsB- 1X acsC-acsV- 1X cooC2-acsD-acsE- GCA 008636105.1 d Bacteria p Planctomycetota c Brocadiae o Brocadiales
cooC-acsA-acsB- GCA 011050115.1 d Bacteria p Planctomycetota c Brocadiae o Brocadiales
cooC-acsB- 1X acsC-acsV-cooC2- 1X acsD-acsE- GCA 004282745.1 d Bacteria p Planctomycetota c Brocadiae o Brocadiales
cooC-acsA-acsB- 3X acsC-acsV-cooC2-acsD-acsE- GCF 900232105.1 d Bacteria p Planctomycetota c Brocadiae o Brocadiales
cooC-acsA-acsB- 1X acsC-acsV-cooC2-acsD-acsE- GCA 900696655.1 d Bacteria p Planctomycetota c Brocadiae o Brocadiales
cooC-acsA-acsB- GCA 011525665.1 d Bacteria p Planctomycetota c Brocadiae o Brocadiales
cooC-acsA-acsB- 1X acsC-acsV-cooC2-acsD-acsE- GCF 000949635.1 d Bacteria p Planctomycetota c Brocadiae o Brocadiales
cooC-acsA-acsB- 1X acsC-acsV-cooC2-acsD-acsE- GCA 005524015.1 d Bacteria p Planctomycetota c Brocadiae o Brocadiales
cooC-acsA-acsB- 1X acsC-acsV-cooC2-acsD-acsE- GCF 000296795.1 d Bacteria p Planctomycetota c Brocadiae o Brocadiales
cooC-acsA-acsB- 1X acsC-acsV-cooC2-acsD-acsE- GCA 013360945.1 d Bacteria p Planctomycetota c Brocadiae o Brocadiales
fhs- 17X cooC-acsA-acsB- 1X acsC-acsV-cooC2-acsD-acsE- GCF 001753675.2 d Bacteria p Planctomycetota c Brocadiae o Brocadiales
cooC-acsA-acsB- 2X acsC- 4X cooC2-acsD-acsE- GCA 000987375.1 d Bacteria p Planctomycetota c Brocadiae o Brocadiales
fhs- 18X cooC-acsA-acsB- 2X acsC-acsV-cooC2- 3X acsD-acsE- GCA 007618135.1 d Bacteria p Planctomycetota c Brocadiae o Brocadiales
cooC-acsA-acsB- 2X acsC-acsV-cooC2-acsD-acsE- GCA 002009475.1 d Bacteria p Planctomycetota c Brocadiae o Brocadiales
fhs- 1X acsV-cooC2-folD- 3X acsD-acsA-acsB-acsC- 4X metV-metF-acsE- GCA 003599855.1 d Bacteria p Actinobacteriota c Thermoleophilia o BMS3ABIN01
acsD-acsA-acsB-acsC- 4X metV-metF-acsE- GCA 002897715.1 d Bacteria p Actinobacteriota c Thermoleophilia o BMS3ABIN01
fhs-acsV-cooC2-fchA-folD-acsD-acsA-acsB-acsC-metV-metF-pdhD- 2X acsE- GCA 012719395.1 d Bacteria p Actinobacteriota c Thermoleophilia o RBG-16-64-13
fhs-acsV-fchA-folD-acsD-acsB- GCA 012514635.1 d Bacteria p Actinobacteriota c Thermoleophilia o RBG-16-64-13
fhs-acsV-cooC2- 1X acsD-acsA-acsB-acsC- 4X metV-metF-cooC-acsE- GCA 013824215.1 d Bacteria p Actinobacteriota c Coriobacteriia o OPB41
cooC2-folD-acsD-acsA-acsB-acsC- 4X metV-metF-cooC-acsE- GCA 005774595.1 d Bacteria p Actinobacteriota c Coriobacteriia o OPB41
acsV-cooC2-folD-acsD-acsA-acsB-acsC- 4X metV-metF-cooC-acsE- GCA 002841275.1 d Bacteria p Actinobacteriota c Coriobacteriia o OPB41
acsD-acsA-acsB-acsC- 4X metV-metF-cooC-acsE- GCA 013334745.1 d Bacteria p Actinobacteriota c Coriobacteriia o OPB41
fhs 9X acsV-cooC2-acsB-acsA- 24X folD- GCF 000174435.1 d Bacteria p Desulfobacterota c Desulfovibrionia o Desulfovibrionales
acsV-cooC2-acsB- GCA 003553695.1 d Bacteria p Desulfobacterota c Desulfovibrionia o Desulfovibrionales
acsB-acsA- 24X cooC2 acsV GCA 003551145.1 d Bacteria p Desulfobacterota c Desulfovibrionia o Desulfovibrionales
acsB-acsA- GCA 003554505.1 d Bacteria p Desulfobacterota c Desulfovibrionia o Desulfovibrionales
acsD-acsA-acsB-acsC- 4X metV-metF-cooC-acsE- GCF 902810715.1 d Bacteria p Actinobacteriota c UBA9087 o UBA10029
acsV-cooC2-folD-acsD-acsA-acsB-acsC- GCA 011047485.1 d Bacteria p Actinobacteriota c UBA9087 o UBA10029
fhs-acsV-cooC2-folD-acsD-acsA-acsB- 7X metV- GCA 001767575.1 d Bacteria p Actinobacteriota c UBA9087 o UBA10029
fchA-folD- 10X fhs-acsV-cooC2-acsD-acsA-acsB-acsC- 4X metV-metF- GCA 001871795.1 d Bacteria p Actinobacteriota c UBA9087 o UBA10029
acsA-acsB-acsC-acsV-cooC2-acsD-acsE- GCA 012961725.1 d Bacteria p Firmicutes B c Desulfotomaculia o Ammonifexales
acsB-acsC-acsV-cooC2-acsD-acsE- GCA 003604985.1 d Bacteria p Firmicutes B c Desulfotomaculia o Ammonifexales
cooC- 10X acsA-acsB-acsC-acsV-cooC2-acsD-acsE- GCF 003815015.1 d Bacteria p Firmicutes B c Desulfotomaculia o Ammonifexales
acsA-acsB-acsC-acsV-cooC2-acsD-acsE- 6X metV-metF- GCF 000024605.1 d Bacteria p Firmicutes B c Desulfotomaculia o Ammonifexales
cooC- 10X acsA-acsB-acsC-acsV-cooC2-acsD-acsE- 6X metV-metF- GCF 003368535.1 d Bacteria p Firmicutes B c Desulfotomaculia o Ammonifexales
acsB-acsC-acsV-cooC2-acsD- GCA 011048115.1 d Bacteria p Firmicutes B c Desulfotomaculia o Ammonifexales
acsA-acsB-acsC-acsV-cooC2-acsD-acsE- 6X metV-metF- GCA 003604975.1 d Bacteria p Firmicutes B c Desulfotomaculia o Ammonifexales
acsA-acsB-acsC-acsV- 1X acsD-acsE- 7X metV-metF- GCF 000018425.1 d Bacteria p Firmicutes B c Desulfotomaculia o Ammonifexales
acsA-acsB-acsC-acsV-cooC2-acsD-acsE- 6X metV-metF- GCA 001899445.1 d Bacteria p Firmicutes B c Desulfotomaculia o Ammonifexales
folD- 21X acsA-acsB-acsC-cooC2-acsD-acsE- 1X metV-metF-fhs- GCA 011048115.1 d Bacteria p Firmicutes B c Desulfotomaculia o Ammonifexales
acsA-folD-acsB-acsC-cooC2-acsD-acsE-metV-metF-fhs- 10X cooC-acsV- GCA 003604975.1 d Bacteria p Firmicutes B c Desulfotomaculia o Ammonifexales
fhs-acsV-cooC2-fchA-folD-acsD-acsA-acsB-acsC-metV-metF- 4X acsE- GCA 014360685.1 d Bacteria p Actinobacteriota c RBG-13-55-18 o RBG-13-55-18
fhs-acsV-cooC2-fchA-folD-acsD-acsA-acsB-acsC-metV-metF- 4X acsE- GCA 014360655.1 d Bacteria p Actinobacteriota c RBG-13-55-18 o RBG-13-55-18
fhs-acsV-cooC2-fchA-folD-acsD-acsA-acsB-acsC-metV-metF- 4X acsE- GCA 014360645.1 d Bacteria p Actinobacteriota c RBG-13-55-18 o RBG-13-55-18
fhs-acsV-cooC2-fchA-folD-acsD-acsA-acsB-acsC-metV-metF- 4X acsE- GCA 013178005.1 d Bacteria p Actinobacteriota c RBG-13-55-18 o RBG-13-55-18
fhs-acsV-cooC2-fchA-folD-acsD-acsA-acsB-acsC-metV-metF- 4X acsE- GCA 001768645.1 d Bacteria p Actinobacteriota c RBG-13-55-18 o RBG-13-55-18
fhs-acsV-cooC2-fchA-folD-acsD-acsA-acsB-acsC-metV-metF- GCA 002843005.1 d Bacteria p Actinobacteriota c RBG-13-55-18 o Fen-727
acsB-acsC- 1X metF- 4X acsE- GCA 003157385.1 d Bacteria p Actinobacteriota c RBG-13-55-18 o Fen-727
cooC- 2X cooC2-acsA-acsB- 1X acsD-acsC-acsV- GCA 003139795.1 d Bacteria p Acidobacteriota c Acidobacteriae o Acidobacteriales
cooC- 2X cooC2-acsA-acsB- 1X acsD-acsC-acsV- GCA 003165795.1 d Bacteria p Acidobacteriota c Acidobacteriae o Acidobacteriales
cooC- 2X cooC2-acsA-acsB- 1X acsD-acsC-acsV- GCA 903909435.1 d Bacteria p Acidobacteriota c Acidobacteriae o Bryobacterales
cooC-cooC2-acsA-acsB-acsD-acsC-acsE-acsV- GCA 903911925.1 d Bacteria p Acidobacteriota c Acidobacteriae o Acidobacteriales
cooC- 2X cooC2-acsA-acsB- 1X acsD-acsC-acsV- GCA 903928345.1 d Bacteria p Armatimonadota c CAIYQO01 o CAIYQO01
acsB- GCA 002431715.1 d Bacteria p Armatimonadota c UBA5829 o UBA5829
cooC2-acsA-acsB-acsD-acsC- GCA 003165595.1 d Bacteria p Fibrobacterota c Chitinivibrionia o Chitinivibrionales

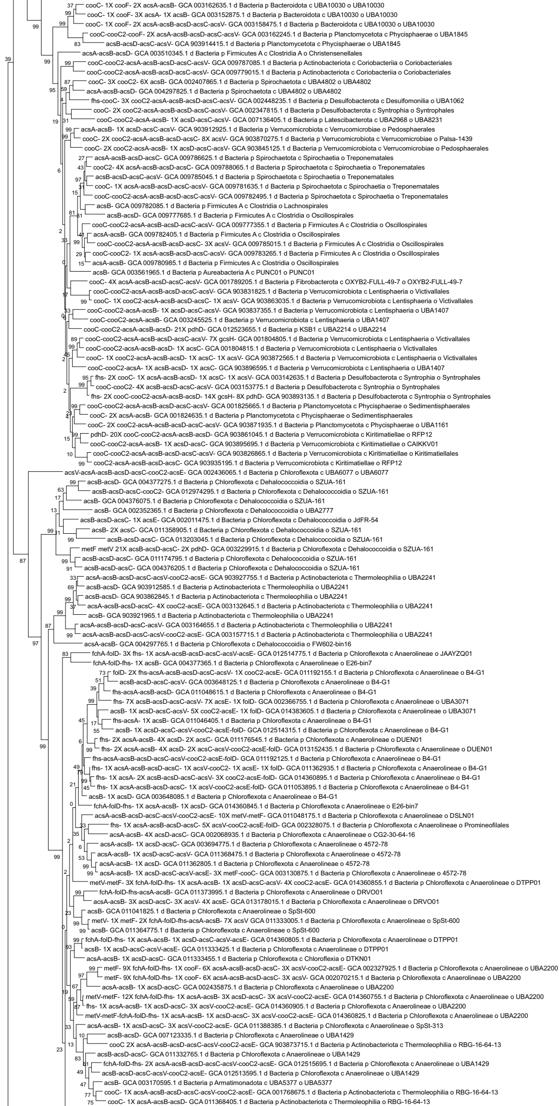

cooC- 1X cooF- 2X acsA-acsB- GCA 003162635.1 d Bacteria p Bacteroidota c UBA10030 o UBA10030
cooC- 1X cooF- 3X acsA- 1X acsB- GCA 003152875.1 d Bacteria p Bacteroidota c UBA10030 o UBA10030
cooC- 1X cooF- 2X acsA-acsB-acsD-acsC-acsV- GCA 003158475.1 d Bacteria p Bacteroidota c UBA10030 o UBA10030
cooC-cooC2-cooF- 2X acsA-acsB-acsD-acsC-acsV- GCA 003162245.1 d Bacteria p Planctomycetota c Phycisphaerae o UBA1845
acsB-acsD-acsC-acsV- GCA 903914415.1 d Bacteria p Planctomycetota c Phycisphaerae o UBA1845
acsA-acsB-acsD- GCA 003510345.1 d Bacteria p Firmicutes A c Clostridia A o Christensenellales
cooC-cooC2-acsA-acsB-acsD-acsC-acsV- GCA 009787085.1 d Bacteria p Actinobacteriota c Coriobacteriia o Coriobacteriales
cooC-cooC2-acsA-acsB-acsD-acsC-acsV- GCA 009779015.1 d Bacteria p Actinobacteriota c Coriobacteriia o Coriobacteriales
cooC- 3X cooC2- 6X acsB- GCA 002407865.1 d Bacteria p Spirochaetota c UBA4802 o UBA4802
acsA-acsB-acsD- GCA 004297825.1 d Bacteria p Spirochaetota c UBA4802 o UBA4802
fhs-cooC- 3X cooC2-acsA-acsB-acsD-acsC-acsV- GCA 002448235.1 d Bacteria p Desulfobacterota c Desulfomonilia o UBA1062
cooC- 2X cooC2-acsA-acsB-acsD-acsC-acsV- GCA 002347815.1 d Bacteria p Desulfobacterota c Syntrophia o Syntrophales
cooC-cooC2-acsA-acsB- 1X acsD-acsC-acsV- GCA 007136405.1 d Bacteria p Latescibacterota c UBA2968 o UBA8231
acsA-acsB- 1X acsD-acsC-acsV- GCA 903912925.1 d Bacteria p Verrucomicrobiota c Verrucomicrobiae o Pedosphaerales
cooC- 2X cooC2-acsA-acsB-acsD-acsC- 8X acsV- GCA 903870275.1 d Bacteria p Verrucomicrobiota c Verrucomicrobiae o Palsa-1439
cooC- 2X cooC2-acsA-acsB- 1X acsD-acsC-acsV- GCA 903845125.1 d Bacteria p Verrucomicrobiota c Verrucomicrobiae o Pedosphaerales
acsA-acsB-acsD-acsC- GCA 009786625.1 d Bacteria p Spirochaetota c Spirochaetia o Treponematales
cooC2- 4X acsA-acsB-acsD-acsC- GCA 009788065.1 d Bacteria p Spirochaetota c Spirochaetia o Treponematales
acsB-acsD-acsC-acsV- GCA 009785045.1 d Bacteria p Spirochaetota c Spirochaetia o Treponematales
cooC- 1X acsA-acsB-acsD-acsC-acsV- GCA 009781635.1 d Bacteria p Spirochaetota c Spirochaetia o Treponematales
cooC-cooC2-acsA-acsB-acsD-acsC-acsV- GCA 009782495.1 d Bacteria p Spirochaetota c Spirochaetia o Treponematales
acsB- GCA 009782085.1 d Bacteria p Firmicutes A c Clostridia o Lachnospirales
acsB-acsD- GCA 009777685.1 d Bacteria p Firmicutes A c Clostridia o Oscillospirales
cooC-cooC2-acsA-acsB-acsD-acsC-acsV- GCA 009777355.1 d Bacteria p Firmicutes A c Clostridia o Oscillospirales
acsA-acsB- GCA 009782405.1 d Bacteria p Firmicutes A c Clostridia o Oscillospirales
cooC-cooC2-acsA-acsB-acsD-acsC- 3X acsV- GCA 009785015.1 d Bacteria p Firmicutes A c Clostridia o Oscillospirales
cooC-cooC2- 1X acsA-acsB-acsD-acsC-acsV- GCA 009783265.1 d Bacteria p Firmicutes A c Clostridia o Oscillospirales
acsA-acsB- GCA 009780985.1 d Bacteria p Firmicutes A c Clostridia o Oscillospirales
acsB- GCA 003561965.1 d Bacteria p Aureabacteria A c PUNC01 o PUNC01
cooC- 4X acsA-acsB-acsD-acsC-acsV- GCA 001789205.1 d Bacteria p Fibrobacterota c OXYB2-FULL-49-7 o OXYB2-FULL-49-7
cooC-cooC2-acsA-acsB-acsD-acsC-acsV- GCA 903831825.1 d Bacteria p Verrucomicrobiota c Lentisphaeria o Victivallales
cooC- 1X cooC2-acsA-acsB-acsD-acsC- 1X acsV- GCA 903863035.1 d Bacteria p Verrucomicrobiota c Lentisphaeria o Victivallales
cooC-cooC2-acsA-acsB- 1X acsD-acsC-acsV- GCA 903837355.1 d Bacteria p Verrucomicrobiota c Lentisphaeria o UBA1407
cooC-cooC2-acsA-acsB- GCA 003245525.1 d Bacteria p Verrucomicrobiota c Lentisphaeria o UBA1407
cooC-cooC2-acsA-acsB-acsD- 21X pdhD- GCA 012523655.1 d Bacteria p KSB1 c UBA2214 o UBA2214
cooC-cooC2-acsA-acsB-acsD-acsC-acsV- 7X gcsH- GCA 001804805.1 d Bacteria p Verrucomicrobiota c Lentisphaeria o Victivallales
cooC-cooC2-acsA-acsB-acsD- 1X acsC- GCA 001804815.1 d Bacteria p Verrucomicrobiota c Lentisphaeria o Victivallales
cooC- 1X cooC2-acsA-acsB-acsD- 1X acsC- 1X acsV- GCA 903872565.1 d Bacteria p Verrucomicrobiota c Lentisphaeria o Victivallales
cooC-cooC2-acsA- 1X acsB-acsD- 1X acsC- GCA 903896595.1 d Bacteria p Verrucomicrobiota c Lentisphaeria o UBA1407
fhs- 2X cooC- 1X acsA-acsB-acsD- 1X acsC- 1X acsV- GCA 003142635.1 d Bacteria p Desulfobacterota c Syntrophia o Syntrophales
cooC-cooC2- 4X acsB-acsD-acsC-acsV- GCA 003153775.1 d Bacteria p Desulfobacterota c Syntrophia o Syntrophales
fhs- 2X cooC-cooC2-acsA-acsB-acsD- 14X gcsH- 8X pdhD- GCA 903893135.1 d Bacteria p Desulfobacterota c Syntrophia o Syntrophales
cooC-cooC2-acsA-acsB-acsD-acsC-acsV- GCA 001825665.1 d Bacteria p Planctomycetota c Phycisphaerae o Sedimentisphaerales
cooC- 2X acsA-acsB- GCA 001824635.1 d Bacteria p Planctomycetota c Phycisphaerae o Sedimentisphaerales
cooC- 2X cooC2-acsA-acsB-acsD-acsC-acsV- GCA 903871935.1 d Bacteria p Planctomycetota c Phycisphaerae o UBA1161
pdhD- 20X cooC-cooC2-acsA-acsB-acsD- GCA 903861045.1 d Bacteria p Verrucomicrobiota c Kiritimatiellae o RFP12
cooC-cooC2-acsA-acsB- 1X acsD-acsC- GCA 903895695.1 d Bacteria p Verrucomicrobiota c Kiritimatiellae o CAIKKV01
cooC-cooC2-acsA-acsB-acsD-acsC-acsV- GCA 903826865.1 d Bacteria p Verrucomicrobiota c Kiritimatiellae o Kiritimatiellales
cooC2-acsA-acsB-acsD-acsC- GCA 903935195.1 d Bacteria p Verrucomicrobiota c Kiritimatiellae o RFP12
acsV-acsA-acsB-acsD-acsC-cooC2-acsE- GCA 002436065.1 d Bacteria p Chloroflexota c UBA6077 o UBA6077
acsB-acsD- GCA 004377275.1 d Bacteria p Chloroflexota c Dehalococcoidia o SZUA-161
acsB-acsD-acsC-cooC2- GCA 012974295.1 d Bacteria p Chloroflexota c Dehalococcoidia o SZUA-161
acsB- GCA 004376075.1 d Bacteria p Chloroflexota c Dehalococcoidia o SZUA-161
acsB- GCA 002352365.1 d Bacteria p Chloroflexota c Dehalococcoidia o UBA2777
acsB-acsD-acsC- 1X acsE- GCA 002011475.1 d Bacteria p Chloroflexota c Dehalococcoidia o JdFR-54
acsB- 2X acsC- GCA 011358905.1 d Bacteria p Chloroflexota c Dehalococcoidia o SZUA-161
acsB-acsD-acsC- GCA 013203045.1 d Bacteria p Chloroflexota c Dehalococcoidia o SZUA-161
metF metV 21X acsB-acsD-acsC- 2X pdhD- GCA 003229915.1 d Bacteria p Chloroflexota c Dehalococcoidia o SZUA-161
acsB-acsD-acsC- GCA 011174795.1 d Bacteria p Chloroflexota c Dehalococcoidia o SZUA-161
acsB-acsD-acsC- GCA 004376205.1 d Bacteria p Chloroflexota c Dehalococcoidia o SZUA-161
acsA-acsB-acsD-acsC-acsV-cooC2-acsE- GCA 903927755.1 d Bacteria p Actinobacteriota c Thermoleophilia o UBA2241
acsB-acsD- GCA 903912585.1 d Bacteria p Actinobacteriota c Thermoleophilia o UBA2241
acsB-acsD- GCA 903862845.1 d Bacteria p Actinobacteriota c Thermoleophilia o UBA2241
acsA-acsB-acsD-acsC- 4X cooC2-acsE- GCA 003132645.1 d Bacteria p Actinobacteriota c Thermoleophilia o UBA2241
acsB- GCA 903921965.1 d Bacteria p Actinobacteriota c Thermoleophilia o UBA2241
acsA-acsB-acsD-acsC-acsV- GCA 003164655.1 d Bacteria p Actinobacteriota c Thermoleophilia o UBA2241
acsA-acsB-acsD-acsC-acsV-cooC2-acsE- GCA 003157715.1 d Bacteria p Actinobacteriota c Thermoleophilia o UBA2241
acsA-acsB- GCA 004297765.1 d Bacteria p Chloroflexota c Dehalococcoidia o FW602-bin16
fchA-folD- 3X fhs- 1X acsA-acsB-acsD-acsC-acsV-acsE- GCA 012514775.1 d Bacteria p Chloroflexota c Anaerolineae o JAAYZQ01
fchA-folD-fhs- 1X acsB- GCA 004377365.1 d Bacteria p Chloroflexota c Anaerolineae o E26-bin7
folD- 2X fhs-acsA-acsB-acsD-acsC-acsV- 1X cooC2-acsE- GCA 011192155.1 d Bacteria p Chloroflexota c Anaerolineae o B4-G1
acsB-acsD-acsC-acsV- GCA 003648125.1 d Bacteria p Chloroflexota c Anaerolineae o B4-G1
fhs-acsA-acsB-acsD- GCA 011048615.1 d Bacteria p Chloroflexota c Anaerolineae o B4-G1
fhs- 7X acsB-acsD-acsC-acsV- 7X acsE- 1X folD- GCA 002366755.1 d Bacteria p Chloroflexota c Anaerolineae o UBA3071
acsB- 1X acsD-acsC-acsV- 5X cooC2-acsE- 1X folD- GCA 014383605.1 d Bacteria p Chloroflexota c Anaerolineae o UBA3071
fhs-acsA- 1X acsB- GCA 011046405.1 d Bacteria p Chloroflexota c Anaerolineae o B4-G1
acsB- 1X acsD-acsC-acsV-cooC2-acsE-folD- GCA 012514315.1 d Bacteria p Chloroflexota c Anaerolineae o B4-G1
fhs- 2X acsA-acsB- 4X acsD- 2X acsC- GCA 011176545.1 d Bacteria p Chloroflexota c Anaerolineae o DUEN01
fhs- 2X acsA-acsB- 4X acsD- 2X acsC-acsV-cooC2-acsE-folD- GCA 013152435.1 d Bacteria p Chloroflexota c Anaerolineae o DUEN01
fhs-acsA-acsB-acsD-acsC-acsV-cooC2-acsE-folD- GCA 011192125.1 d Bacteria p Chloroflexota c Anaerolineae o B4-G1
fhs- 1X acsA-acsB-acsD-acsC- 1X acsV-cooC2- 1X acsE- 1X folD- GCA 011362935.1 d Bacteria p Chloroflexota c Anaerolineae o B4-G1
fhs- 1X acsA- 2X acsB-acsD-acsC-acsV- 3X cooC2-acsE-folD- GCA 014360895.1 d Bacteria p Chloroflexota c Anaerolineae o B4-G1
fhs- 1X acsA-acsB-acsD-acsC- 1X acsV-cooC2-acsE-folD- GCA 011053895.1 d Bacteria p Chloroflexota c Anaerolineae o B4-G1
acsB- 1X acsD- GCA 003648085.1 d Bacteria p Chloroflexota c Anaerolineae o B4-G1
fchA-folD-fhs- 1X acsA-acsB- 1X acsD- GCA 014360845.1 d Bacteria p Chloroflexota c Anaerolineae o E26-bin7
acsA-acsB-acsD-acsC-acsV-cooC2-acsE- 10X metV-metF- GCA 011048175.1 d Bacteria p Chloroflexota c Anaerolineae o DSLN01
fhs- 1X acsA-acsB-acsD-acsC- 5X acsV-cooC2-acsE-folD- GCA 002328075.1 d Bacteria p Chloroflexota c Anaerolineae o Promineofilales
acsA-acsB- 4X acsD-acsC- GCA 002068935.1 d Bacteria p Chloroflexota c Anaerolineae o CG2-30-64-16
acsA-acsB- 1X acsD-acsC- GCA 003694775.1 d Bacteria p Chloroflexota c Anaerolineae o 4572-78
acsA-acsB- 1X acsD-acsC-acsV- GCA 011368475.1 d Bacteria p Chloroflexota c Anaerolineae o 4572-78
acsA-acsB- 1X acsD- GCA 011362805.1 d Bacteria p Chloroflexota c Anaerolineae o 4572-78
acsA-acsB- 1X acsD-acsC-acsV-acsE- 3X metF-cooC- GCA 003130875.1 d Bacteria p Chloroflexota c Anaerolineae o 4572-78
metV-metF- 3X fchA-folD-fhs- 1X acsA-acsB- 1X acsD-acsC-acsV- 4X cooC2-acsE- GCA 014360855.1 d Bacteria p Chloroflexota c Anaerolineae o DTPP01
fchA-folD-fhs-acsA-acsB- GCA 011373995.1 d Bacteria p Chloroflexota c Anaerolineae o DRVO01
acsA-acsB- 3X acsD-acsC- 3X acsV- 4X acsE- GCA 013178015.1 d Bacteria p Chloroflexota c Anaerolineae o DRVO01
acsB- GCA 011041825.1 d Bacteria p Chloroflexota c Anaerolineae o SpSt-600
metV- 1X metF- 2X fchA-folD-fhs-acsA-acsB- 7X acsV GCA 011333005.1 d Bacteria p Chloroflexota c Anaerolineae o SpSt-600
acsB- GCA 011364775.1 d Bacteria p Chloroflexota c Anaerolineae o SpSt-600
fchA-folD-fhs- 1X acsA-acsB- 1X acsD-acsC-acsV-acsE- GCA 014360805.1 d Bacteria p Chloroflexota c Anaerolineae o DTPP01
acsB- 1X acsD-acsC-acsV-acsE- GCA 011333425.1 d Bacteria p Chloroflexota c Anaerolineae o DTPP01
acsA-acsB- 1X acsD-acsC- GCA 011333455.1 d Bacteria p Chloroflexota c Chloroflexia o DTKN01
metF- 9X fchA-folD-fhs- 1X cooF- 6X acsA-acsB-acsD-acsC- 3X acsV-cooC2-acsE- GCA 002327925.1 d Bacteria p Chloroflexota c Anaerolineae o UBA2200
metF- 9X fchA-folD-fhs- 1X cooF- 6X acsA-acsB-acsD-acsC- 3X acsV- GCA 002070215.1 d Bacteria p Chloroflexota c Anaerolineae o UBA2200
acsA-acsB- 1X acsD-acsC- GCA 002435875.1 d Bacteria p Chloroflexota c Anaerolineae o UBA2200
metV-metF- 12X fchA-folD-fhs- 1X acsA-acsB- 3X acsD-acsC- 3X acsV-cooC2-acsE- GCA 014360755.1 d Bacteria p Chloroflexota c Anaerolineae o UBA2200
fhs- 1X acsA-acsB- 1X acsD-acsC- 3X acsV-cooC2-acsE- GCA 014360905.1 d Bacteria p Chloroflexota c Anaerolineae o UBA2200
metV-metF-fchA-folD-fhs- 1X acsA-acsB- 1X acsD-acsC- 3X acsV-cooC2-acsE- GCA 014360825.1 d Bacteria p Chloroflexota c Anaerolineae o UBA2200
acsA-acsB- 1X acsD-acsC- 3X acsV-cooC2-acsE- GCA 011388385.1 d Bacteria p Chloroflexota c Anaerolineae o SpSt-313
acsB-acsD- GCA 007123335.1 d Bacteria p Chloroflexota c Anaerolineae o UBA1429
cooC 2X acsA-acsB-acsD-acsC-acsV-cooC2-acsE- GCA 903873715.1 d Bacteria p Actinobacteriota c Thermoleophilia o RBG-16-64-13
acsB-acsD-acsC- GCA 011332765.1 d Bacteria p Chloroflexota c Anaerolineae o UBA1429
fchA-folD-fhs- 2X acsA-acsB-acsD-acsC-acsV-cooC2-acsE- GCA 012515695.1 d Bacteria p Chloroflexota c Anaerolineae o UBA1429
acsB-acsD-acsC-acsV-cooC2-acsE- GCA 012513595.1 d Bacteria p Chloroflexota c Anaerolineae o UBA1429
acsB- GCA 003170595.1 d Bacteria p Armatimonadota c UBA5377 o UBA5377
cooC- 1X acsA-acsB-acsD-acsC-acsV-cooC2-acsE- GCA 001768675.1 d Bacteria p Actinobacteriota c Thermoleophilia o RBG-16-64-13
cooC- 1X acsA-acsB-acsD- GCA 011368405.1 d Bacteria p Actinobacteriota c Thermoleophilia o RBG-16-64-13

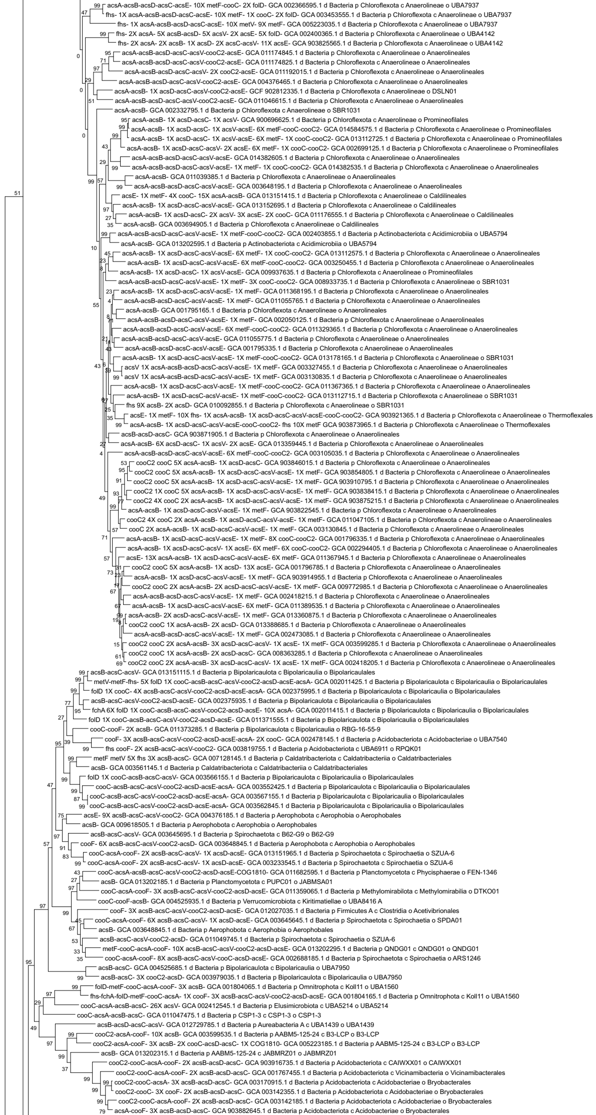
acsA-acsB-acsD-acsC-acsE- 10X metF-cooC- 2X folD- GCA 002366595.1 d Bacteria p Chloroflexota c Anaerolineae o UBA7937
fhs- 1X acsA-acsB-acsD-acsC-acsE- 10X metF- 1X cooC- 2X folD- GCA 003453555.1 d Bacteria p Chloroflexota c Anaerolineae o UBA7937
fhs- 1X acsA-acsB-acsD-acsC-acsE- 10X metV- 9X metF- GCA 005223035.1 d Bacteria p Chloroflexota c Anaerolineae o UBA7937
fhs- 2X acsA- 5X acsB-acsD- 5X acsV- 2X acsE- 5X folD- GCA 002400365.1 d Bacteria p Chloroflexota c Anaerolineae o UBA4142
fhs- 2X acsA- 2X acsB- 1X acsD- 2X acsC-acsV- 11X acsE- GCA 903825565.1 d Bacteria p Chloroflexota c Anaerolineae o UBA4142
acsA-acsB-acsD-acsC-acsV-cooC2-acsE- GCA 011174845.1 d Bacteria p Chloroflexota c Anaerolineae o Anaerolineales
acsA-acsB-acsD-acsC-acsV-cooC2-acsE- GCA 011174825.1 d Bacteria p Chloroflexota c Anaerolineae o Anaerolineales
acsA-acsB-acsD-acsC-acsV- 2X cooC2-acsE- GCA 011192015.1 d Bacteria p Chloroflexota c Anaerolineae o Anaerolineales
acsA-acsB-acsD-acsC-acsV-cooC2-acsE- GCA 004376465.1 d Bacteria p Chloroflexota c Anaerolineae o Anaerolineales
acsA-acsB- 1X acsD-acsC-acsV-cooC2-acsE- GCF 902812335.1 d Bacteria p Chloroflexota c Anaerolineae o DSLN01
acsA-acsB-acsD-acsC-acsV-cooC2-acsE- GCA 011046615.1 d Bacteria p Chloroflexota c Anaerolineae o Anaerolineales
acsA-acsB- GCA 002332795.1 d Bacteria p Chloroflexota c Anaerolineae o SBR1031
acsA-acsB- 1X acsD-acsC- 1X acsV- GCA 900696625.1 d Bacteria p Chloroflexota c Anaerolineae o Promineofilales
acsA-acsB- 1X acsD-acsC- 1X acsV-acsE- 6X metF-cooC-cooC2- GCA 014584575.1 d Bacteria p Chloroflexota c Anaerolineae o Promineofilales
acsA-acsB- 1X acsD-acsC- 1X acsV-acsE- 6X metF- 1X cooC-cooC2- GCA 013112725.1 d Bacteria p Chloroflexota c Anaerolineae o Promineofilales
acsA-acsB- 1X acsD-acsC-acsV- 2X acsE- 6X metF- 1X cooC-cooC2- GCA 002699125.1 d Bacteria p Chloroflexota c Anaerolineae o Promineofilales
acsA-acsB-acsD-acsC-acsV-acsE- GCA 014382605.1 d Bacteria p Chloroflexota c Anaerolineae o Anaerolineales
acsA-acsB-acsD-acsC-acsV-acsE- 1X metF- 1X cooC-cooC2- GCA 014382535.1 d Bacteria p Chloroflexota c Anaerolineae o Anaerolineales
acsA-acsB- GCA 011039385.1 d Bacteria p Chloroflexota c Anaerolineae o Anaerolineales
acsA-acsB-acsD-acsC-acsV-acsE- GCA 003648195.1 d Bacteria p Chloroflexota c Anaerolineae o Anaerolineales
acsE- 1X metF- 4X cooC- 15X acsA-acsB- GCA 013151415.1 d Bacteria p Chloroflexota c Anaerolineae o Caldilineales
acsA-acsB- 1X acsD-acsC-acsV-acsE- GCA 013152695.1 d Bacteria p Chloroflexota c Anaerolineae o Caldilineales
acsA-acsB- 1X acsD-acsC- 2X acsV- 3X acsE- 2X cooC- GCA 011176555.1 d Bacteria p Chloroflexota c Anaerolineae o Caldilineales
acsA-acsB- GCA 003694905.1 d Bacteria p Chloroflexota c Anaerolineae o Caldilineales
acsA-acsB-acsD-acsC-acsV-acsE- 1X metF-cooC-cooC2- GCA 002403855.1 d Bacteria p Actinobacteriota c Acidimicrobiia o UBA5794
acsA-acsB- GCA 013202595.1 d Bacteria p Actinobacteriota c Acidimicrobiia o UBA5794
acsA-acsB- 1X acsD-acsC-acsV-acsE- 6X metF- 1X cooC-cooC2- GCA 013112575.1 d Bacteria p Chloroflexota c Anaerolineae o Anaerolineales
acsA-acsB- 1X acsD-acsC-acsV-acsE- 6X metF-cooC-cooC2- GCA 003250455.1 d Bacteria p Chloroflexota c Anaerolineae o Anaerolineales
acsA-acsB- 1X acsD-acsC- 1X acsV-acsE- GCA 009937635.1 d Bacteria p Chloroflexota c Anaerolineae o Promineofilales
acsA-acsB-acsD-acsC-acsV-acsE- 1X metF- 3X cooC-cooC2- GCA 008933735.1 d Bacteria p Chloroflexota c Anaerolineae o SBR1031
acsA-acsB- 1X acsD-acsC-acsV-acsE- 1X metF- GCA 011368195.1 d Bacteria p Chloroflexota c Anaerolineae o Anaerolineales
acsA-acsB-acsD-acsC-acsV-acsE- 1X metF- GCA 011055765.1 d Bacteria p Chloroflexota c Anaerolineae o Anaerolineales
acsA-acsB- GCA 001795165.1 d Bacteria p Chloroflexota c Anaerolineae o Anaerolineales
acsA-acsB-acsD-acsC-acsV-acsE- 1X metF- GCA 002050125.1 d Bacteria p Chloroflexota c Anaerolineae o Anaerolineales
acsA-acsB-acsD-acsC-acsV-acsE- 6X metF-cooC-cooC2- GCA 011329365.1 d Bacteria p Chloroflexota c Anaerolineae o Anaerolineales
acsA-acsB-acsD-acsC-acsV-acsE- GCA 011055775.1 d Bacteria p Chloroflexota c Anaerolineae o Anaerolineales
acsA-acsB-acsD-acsC-acsV-acsE- GCA 001795335.1 d Bacteria p Chloroflexota c Anaerolineae o Anaerolineales
acsA-acsB- 1X acsD-acsC-acsV-acsE- 1X metF-cooC-cooC2- GCA 013178165.1 d Bacteria p Chloroflexota c Anaerolineae o SBR1031
acsV 1X acsA-acsB-acsD-acsC-acsV-acsE- 1X metF- GCA 003327455.1 d Bacteria p Chloroflexota c Anaerolineae o Anaerolineales
acsV 1X acsA-acsB-acsD-acsC-acsV-acsE- 1X metF- GCA 003130835.1 d Bacteria p Chloroflexota c Anaerolineae o Anaerolineales
acsA-acsB- 1X acsD-acsC-acsV-acsE- 1X metF-cooC-cooC2- GCA 011367365.1 d Bacteria p Chloroflexota c Anaerolineae o Anaerolineales
acsA-acsB- 1X acsD-acsC-acsV-acsE- 1X metF-cooC-cooC2- GCA 013112715.1 d Bacteria p Chloroflexota c Anaerolineae o SBR1031
fhs 9X acsB- 2X acsD- GCA 010092855.1 d Bacteria p Chloroflexota c Anaerolineae o SBR1031
acsE- 1X metF- 10X fhs- 1X acsA-acsB- 1X acsD-acsC-acsV-acsE-cooC-cooC2- GCA 903921365.1 d Bacteria p Chloroflexota c Anaerolineae o Thermoflexales
acsA-acsB- 1X acsD-acsC-acsV-acsE-cooC-cooC2- fhs 10X metF GCA 903873965.1 d Bacteria p Chloroflexota c Anaerolineae o Thermoflexales
acsB-acsD-acsC- GCA 903871905.1 d Bacteria p Chloroflexota c Anaerolineae o Anaerolineales
acsA-acsB- 6X acsD-acsC- 1X acsV- 2X acsE- GCA 013359445.1 d Bacteria p Chloroflexota c Anaerolineae o Anaerolineales
acsA-acsB-acsD-acsC-acsV-acsE- 6X metF-cooC-cooC2- GCA 003105035.1 d Bacteria p Chloroflexota c Anaerolineae o Anaerolineales
cooC2 cooC 5X acsA-acsB- 1X acsD-acsC- GCA 903846015.1 d Bacteria p Chloroflexota c Anaerolineae o Anaerolineales
cooC2 cooC 5X acsA-acsB- 1X acsD-acsC-acsV-acsE- 1X metF- GCA 903854805.1 d Bacteria p Chloroflexota c Anaerolineae o Anaerolineales
cooC2 cooC 5X acsA-acsB- 1X acsD-acsC-acsV-acsE- 1X metF- GCA 903910795.1 d Bacteria p Chloroflexota c Anaerolineae o Anaerolineales
cooC2 1X cooC 5X acsA-acsB- 1X acsD-acsC-acsV-acsE- 1X metF- GCA 903838415.1 d Bacteria p Chloroflexota c Anaerolineae o Anaerolineales
cooC2 4X cooC 2X acsA-acsB- 1X acsD-acsC-acsV-acsE- 1X metF- GCA 903875215.1 d Bacteria p Chloroflexota c Anaerolineae o Anaerolineales
acsA-acsB- 1X acsD-acsC-acsV-acsE- 1X metF- GCA 903822545.1 d Bacteria p Chloroflexota c Anaerolineae o Anaerolineales
cooC2 4X cooC 2X acsA-acsB- 1X acsD-acsC-acsV-acsE- 1X metF- GCA 011047105.1 d Bacteria p Chloroflexota c Anaerolineae o Anaerolineales
cooC 2X acsA-acsB- 1X acsD-acsC-acsV-acsE- 1X metF- GCA 003130845.1 d Bacteria p Chloroflexota c Anaerolineae o Anaerolineales
acsA-acsB- 1X acsD-acsC-acsV-acsE- 1X metF- 8X cooC-cooC2- GCA 001796335.1 d Bacteria p Chloroflexota c Anaerolineae o Anaerolineales
acsA-acsB- 1X acsD-acsC-acsV- 1X acsE- 6X metF- 6X cooC-cooC2- GCA 002294405.1 d Bacteria p Chloroflexota c Anaerolineae o Anaerolineales
acsE- 13X acsA-acsB- 1X acsD-acsC-acsV-acsE- 6X metF- GCA 011367945.1 d Bacteria p Chloroflexota c Anaerolineae o Anaerolineales
cooC2 cooC 5X acsA-acsB- 1X acsD- 13X acsE- GCA 001796785.1 d Bacteria p Chloroflexota c Anaerolineae o Anaerolineales
acsA-acsB- 1X acsD-acsC-acsV-acsE- 1X metF- GCA 903914955.1 d Bacteria p Chloroflexota c Anaerolineae o Anaerolineales
cooC2 cooC 2X acsA-acsB- 2X acsD-acsC-acsV-acsE- 1X metF- GCA 009772985.1 d Bacteria p Chloroflexota c Anaerolineae o Anaerolineales
acsA-acsB-acsD-acsC-acsV-acsE- 1X metF- GCA 002418215.1 d Bacteria p Chloroflexota c Anaerolineae o Anaerolineales
acsA-acsB- 1X acsD-acsC-acsV-acsE- 6X metF- GCA 011389535.1 d Bacteria p Chloroflexota c Anaerolineae o Anaerolineales
acsA-acsB- 2X acsD-acsC-acsV-acsE- 1X metF- GCA 013360875.1 d Bacteria p Chloroflexota c Anaerolineae o Anaerolineales
cooC2 cooC 1X acsA-acsB- 2X acsD- GCA 013388685.1 d Bacteria p Chloroflexota c Anaerolineae o Anaerolineales
acsA-acsB-acsD-acsC-acsV-acsE- 1X metF- GCA 002473085.1 d Bacteria p Chloroflexota c Anaerolineae o Anaerolineales
cooC2 cooC 2X acsA-acsB- 3X acsD-acsC-acsV- 1X acsE- 1X metF- GCA 003599285.1 d Bacteria p Chloroflexota c Anaerolineae o Anaerolineales
cooC2 cooC 1X acsA-acsB- 2X acsD-acsC- GCA 008363285.1 d Bacteria p Chloroflexota c Anaerolineae o Anaerolineales
cooC2 cooC 2X acsA-acsB- 3X acsD-acsC-acsV- 1X acsE- 1X metF- GCA 002418205.1 d Bacteria p Chloroflexota c Anaerolineae o Anaerolineales
acsB-acsC-acsV- GCA 013151115.1 d Bacteria p Bipolaricaulota c Bipolaricaulia o Bipolaricaulales
metV-metF-fhs- 5X folD 1X cooC-acsB-acsC-acsV-cooC2-acsD-acsE-acsA- GCA 002011425.1 d Bacteria p Bipolaricaulota c Bipolaricaulia o Bipolaricaulales
folD 1X cooC- 4X acsB-acsC-acsV-cooC2-acsD-acsE-acsA- GCA 002375995.1 d Bacteria p Bipolaricaulota c Bipolaricaulia o Bipolaricaulales
acsB-acsC-acsV-cooC2-acsD-acsE- GCA 002375935.1 d Bacteria p Bipolaricaulota c Bipolaricaulia o Bipolaricaulales
fchA 6X folD 1X cooC-acsB-acsC-acsV-cooC2-acsD-acsE- 10X acsA- GCA 002011415.1 d Bacteria p Bipolaricaulota c Bipolaricaulia o Bipolaricaulales
folD 1X cooC-acsB-acsC-acsV-cooC2-acsD-acsE- GCA 011371555.1 d Bacteria p Bipolaricaulota c Bipolaricaulia o Bipolaricaulales
cooC-cooF- 2X acsB- GCA 011373285.1 d Bacteria p Bipolaricaulota c Bipolaricaulia o RBG-16-55-9
cooF- 3X acsB-acsC-acsV-cooC2-acsD-acsE-acsA- 2X cooC- GCA 002478145.1 d Bacteria p Acidobacteriota c Acidobacteriae o UBA7540
fhs cooF- 2X acsB-acsC-acsV-cooC2- GCA 003819755.1 d Bacteria p Acidobacteriota c UBA6911 o RPQK01
metF metV 5X fhs 3X acsB-acsC- GCA 007128145.1 d Bacteria p Caldatribacteriota c Caldatribacteriia o Caldatribacteriales
acsB- GCA 003561145.1 d Bacteria p Caldatribacteriota c Caldatribacteriia o Caldatribacteriales
folD 1X cooC-acsB-acsC-acsV- GCA 003566155.1 d Bacteria p Bipolaricaulota c Bipolaricaulia o Bipolaricaulales
cooC-acsB-acsC-acsV-cooC2-acsD-acsE-acsA- GCA 003552425.1 d Bacteria p Bipolaricaulota c Bipolaricaulia o Bipolaricaulales
cooC-acsB-acsC-acsV-cooC2-acsD-acsE-acsA- GCA 003567155.1 d Bacteria p Bipolaricaulota c Bipolaricaulia o Bipolaricaulales
cooC-acsB-acsC-acsV-cooC2-acsD-acsE-acsA- GCA 003562845.1 d Bacteria p Bipolaricaulota c Bipolaricaulia o Bipolaricaulales
acsE- 9X acsB-acsC-acsV-cooC2- GCA 004376185.1 d Bacteria p Aerophobota c Aerophobia o Aerophobales
acsB- GCA 009618505.1 d Bacteria p Aerophobota c Aerophobia o Aerophobales
acsB-acsC-acsV- GCA 003645695.1 d Bacteria p Spirochaetota c B62-G9 o B62-G9
cooF- 6X acsB-acsC-acsV-cooC2-acsD- GCA 003648845.1 d Bacteria p Aerophobota c Aerophobia o Aerophobales
cooC-acsA-cooF- 2X acsB-acsC-acsV- 1X acsD-acsE- GCA 013151965.1 d Bacteria p Spirochaetota c Spirochaetia o SZUA-6
cooC-acsA-cooF- 2X acsB-acsC-acsV- 1X acsD-acsE- GCA 003233545.1 d Bacteria p Spirochaetota c Spirochaetia o SZUA-6
cooC-acsA-acsB-acsC-acsV-cooC2-acsD-acsE-COG1810- GCA 011682595.1 d Bacteria p Planctomycetota c Phycisphaerae o FEN-1346
acsB- GCA 013202185.1 d Bacteria p Planctomycetota c PUPC01 o JABMSA01
cooC-acsA-cooF- 3X acsB-acsC-acsV-cooC2-acsD-acsE- GCA 011359065.1 d Bacteria p Methylomirabilota c Methylomirabilia o DTKO01
cooC-cooF-acsB- GCA 004525935.1 d Bacteria p Verrucomicrobiota c Kiritimatiellae o UBA8416 A
cooF- 3X acsB-acsC-acsV-cooC2-acsD-acsE- GCA 012027035.1 d Bacteria p Firmicutes A c Clostridia o Acetivibrionales
cooC-acsA-cooF- 6X acsB-acsC-acsV- 1X acsD-acsE- GCA 003645645.1 d Bacteria p Spirochaetota c Spirochaetia o SPDA01
acsB- GCA 003648845.1 d Bacteria p Aerophobota c Aerophobia o Aerophobales
acsB-acsC-acsV-cooC2-acsD- GCA 011049745.1 d Bacteria p Spirochaetota c Spirochaetia o SZUA-6
metF-cooC-acsA-cooF- 10X acsB-acsC-acsV-cooC2-acsD-acsE- GCA 013202295.1 d Bacteria p QNDG01 c QNDG01 o QNDG01
cooC-acsA-cooF- 8X acsB-acsC-acsV-cooC-acsD-acsE- GCA 002688185.1 d Bacteria p Spirochaetota c Spirochaetia o ARS1246
acsB-acsC- GCA 004525685.1 d Bacteria p Bipolaricaulota c Bipolaricaulia o UBA7950
acsB-acsC- 3X cooC2-acsD- GCA 003979035.1 d Bacteria p Bipolaricaulota c Bipolaricaulia o UBA7950
folD-metF-cooC-acsA-cooF- 3X acsB- GCA 001804065.1 d Bacteria p Omnitrophota c Koll11 o UBA1560
fhs-fchA-folD-metF-cooC-acsA- 1X cooF- 3X acsB-acsC-acsV-cooC2-acsD-acsE- GCA 001804165.1 d Bacteria p Omnitrophota c Koll11 o UBA1560
cooC-acsA-acsB-acsC- 26X acsV- GCA 002412545.1 d Bacteria p Elusimicrobiota c UBA5214 o UBA5214
cooC-acsA-acsB-acsC- GCA 011047475.1 d Bacteria p CSP1-3 c CSP1-3 o CSP1-3
acsB-acsD-acsC-acsV- GCA 012729785.1 d Bacteria p Aureabacteria A c UBA1439 o UBA1439
cooC2-acsA-cooF- 10X acsB- GCA 003599535.1 d Bacteria p AABM5-125-24 c B3-LCP o B3-LCP
cooC2-acsA-cooF- 3X acsB- 2X cooC-acsD-acsC- 1X COG1810- GCA 005223185.1 d Bacteria p AABM5-125-24 c B3-LCP o B3-LCP
acsB- GCA 013202315.1 d Bacteria p AABM5-125-24 c JABMRZ01 o JABMRZ01
cooC2-cooC-acsA-cooF- 2X acsB-acsD-acsC- GCA 903916735.1 d Bacteria p Acidobacteriota c CAIWXX01 o CAIWXX01
cooC2-cooC-acsA-cooF- 2X acsB-acsD-acsC- GCA 001767455.1 d Bacteria p Acidobacteriota c Vicinamibacteria o Vicinamibacterales
cooC2-cooC-acsA- 3X acsB-acsD-acsC- GCA 003170915.1 d Bacteria p Acidobacteriota c Acidobacteriae o Bryobacterales
cooC2-cooC- 3X cooF- 2X acsB-acsD-acsC- GCA 003142355.1 d Bacteria p Acidobacteriota c Acidobacteriae o Bryobacterales
cooC2-cooC-acsA-cooF- 2X acsB-acsD-acsC- GCA 003142185.1 d Bacteria p Acidobacteriota c Acidobacteriae o Bryobacterales
acsA-cooF- 3X acsB-acsD-acsC- GCA 903882645.1 d Bacteria p Acidobacteriota c Acidobacteriae o Bryobacterales

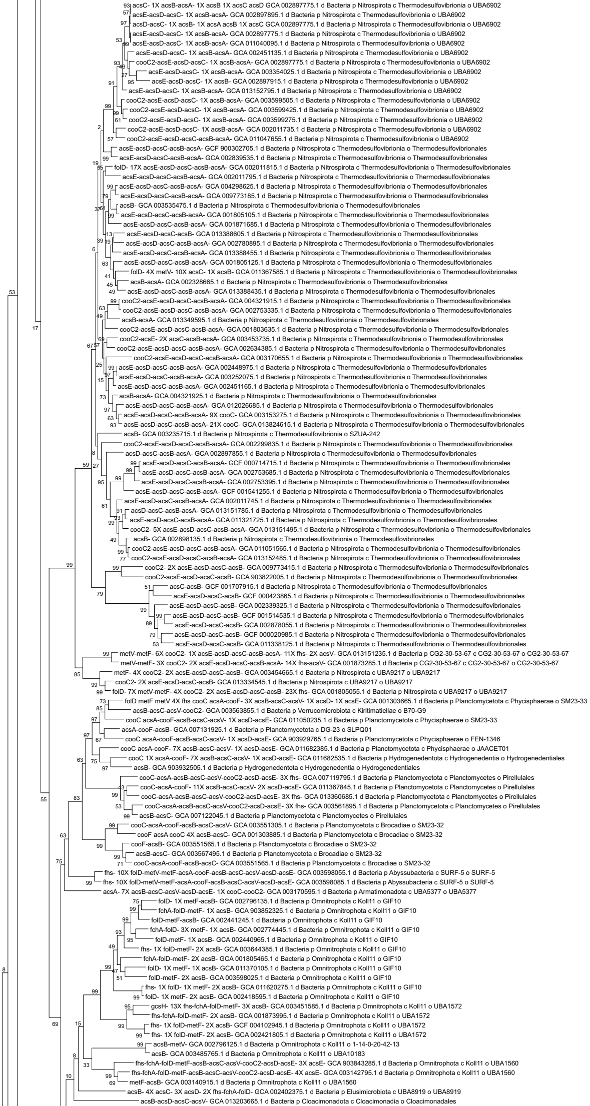
acsC- 1X acsB-acsA- 1X acsB 1X acsC acsD GCA 002897775.1 d Bacteria p Nitrospirota c Thermodesulfovibrionia o UBA6902
acsE-acsD-acsC- 1X acsB-acsA- GCA 002897895.1 d Bacteria p Nitrospirota c Thermodesulfovibrionia o UBA6902
acsD-acsC- 1X acsB- 1X acsA acsB 1X acsC GCA 002897775.1 d Bacteria p Nitrospirota c Thermodesulfovibrionia o UBA6902
acsE-acsD-acsC- 1X acsB-acsA- GCA 002897775.1 d Bacteria p Nitrospirota c Thermodesulfovibrionia o UBA6902
acsE-acsD-acsC- 1X acsB-acsA- GCA 011040095.1 d Bacteria p Nitrospirota c Thermodesulfovibrionia o UBA6902
acsE-acsD-acsC- 1X acsB-acsA- GCA 002451135.1 d Bacteria p Nitrospirota c Thermodesulfovibrionia o UBA6902
cooC2-acsE-acsD-acsC- 1X acsB-acsA- GCA 002897775.1 d Bacteria p Nitrospirota c Thermodesulfovibrionia o UBA6902
acsE-acsD-acsC- 1X acsB-acsA- GCA 003354025.1 d Bacteria p Nitrospirota c Thermodesulfovibrionia o UBA6902
acsE-acsD-acsC- 1X acsB- GCA 002897915.1 d Bacteria p Nitrospirota c Thermodesulfovibrionia o UBA6902
acsE-acsD-acsC- 1X acsB-acsA- GCA 013152795.1 d Bacteria p Nitrospirota c Thermodesulfovibrionia o UBA6902
cooC2-acsE-acsD-acsC- 1X acsB-acsA- GCA 003599505.1 d Bacteria p Nitrospirota c Thermodesulfovibrionia o UBA6902
cooC2-acsE-acsD-acsC- 1X acsB-acsA- GCA 003599425.1 d Bacteria p Nitrospirota c Thermodesulfovibrionia o UBA6902
cooC2-acsE-acsD-acsC- 1X acsB-acsA- GCA 003599275.1 d Bacteria p Nitrospirota c Thermodesulfovibrionia o UBA6902
cooC2-acsE-acsD-acsC- 1X acsB-acsA- GCA 002011735.1 d Bacteria p Nitrospirota c Thermodesulfovibrionia o UBA6902
cooC2-acsE-acsD-acsC-acsB-acsA- GCA 011047655.1 d Bacteria p Nitrospirota c Thermodesulfovibrionia o UBA6902
acsE-acsD-acsC-acsB-acsA- GCF 900302705.1 d Bacteria p Nitrospirota c Thermodesulfovibrionia o Thermodesulfovibrionales
acsE-acsD-acsC-acsB-acsA- GCA 002839535.1 d Bacteria p Nitrospirota c Thermodesulfovibrionia o Thermodesulfovibrionales
folD- 17X acsE-acsD-acsC-acsB-acsA- GCA 002011815.1 d Bacteria p Nitrospirota c Thermodesulfovibrionia o Thermodesulfovibrionales
acsE-acsD-acsC-acsB-acsA- GCA 002011795.1 d Bacteria p Nitrospirota c Thermodesulfovibrionia o Thermodesulfovibrionales
acsE-acsD-acsC-acsB-acsA- GCA 004298625.1 d Bacteria p Nitrospirota c Thermodesulfovibrionia o Thermodesulfovibrionales
acsE-acsD-acsC-acsB-acsA- GCA 009773185.1 d Bacteria p Nitrospirota c Thermodesulfovibrionia o Thermodesulfovibrionales
acsB- GCA 003535475.1 d Bacteria p Nitrospirota c Thermodesulfovibrionia o Thermodesulfovibrionales
acsE-acsD-acsC-acsB-acsA- GCA 001805105.1 d Bacteria p Nitrospirota c Thermodesulfovibrionia o Thermodesulfovibrionales
acsE-acsD-acsC-acsB-acsA- GCA 001871685.1 d Bacteria p Nitrospirota c Thermodesulfovibrionia o Thermodesulfovibrionales
acsE-acsD-acsC-acsB- GCA 013388605.1 d Bacteria p Nitrospirota c Thermodesulfovibrionia o Thermodesulfovibrionales
acsE-acsD-acsC-acsB-acsA- GCA 002780895.1 d Bacteria p Nitrospirota c Thermodesulfovibrionia o Thermodesulfovibrionales
acsE-acsD-acsC-acsB-acsA- GCA 013388455.1 d Bacteria p Nitrospirota c Thermodesulfovibrionia o Thermodesulfovibrionales
acsE-acsD-acsC-acsB-acsA- GCA 001805125.1 d Bacteria p Nitrospirota c Thermodesulfovibrionia o Thermodesulfovibrionales
folD- 4X metV- 10X acsC- 1X acsB- GCA 011367585.1 d Bacteria p Nitrospirota c Thermodesulfovibrionia o Thermodesulfovibrionales
acsB-acsA- GCA 002328665.1 d Bacteria p Nitrospirota c Thermodesulfovibrionia o Thermodesulfovibrionales
acsE-acsD-acsC-acsB-acsA- GCA 013388435.1 d Bacteria p Nitrospirota c Thermodesulfovibrionia o Thermodesulfovibrionales
cooC2-acsE-acsD-acsC-acsB-acsA- GCA 004321915.1 d Bacteria p Nitrospirota c Thermodesulfovibrionia o Thermodesulfovibrionales
cooC2-acsE-acsD-acsC-acsB-acsA- GCA 002753335.1 d Bacteria p Nitrospirota c Thermodesulfovibrionia o Thermodesulfovibrionales
acsB-acsA- GCA 013349595.1 d Bacteria p Nitrospirota c Thermodesulfovibrionia o Thermodesulfovibrionales
cooC2-acsE-acsD-acsC-acsB-acsA- GCA 001803635.1 d Bacteria p Nitrospirota c Thermodesulfovibrionia o Thermodesulfovibrionales
cooC2-acsE- 2X acsC-acsB-acsA- GCA 003453735.1 d Bacteria p Nitrospirota c Thermodesulfovibrionia o Thermodesulfovibrionales
cooC2-acsE-acsD-acsC-acsB-acsA- GCA 002634385.1 d Bacteria p Nitrospirota c Thermodesulfovibrionia o Thermodesulfovibrionales
cooC2-acsE-acsD-acsC-acsB-acsA- GCA 003170655.1 d Bacteria p Nitrospirota c Thermodesulfovibrionia o Thermodesulfovibrionales
acsE-acsD-acsC-acsB-acsA- GCA 002448975.1 d Bacteria p Nitrospirota c Thermodesulfovibrionia o Thermodesulfovibrionales
acsE-acsD-acsC-acsB-acsA- GCA 003252075.1 d Bacteria p Nitrospirota c Thermodesulfovibrionia o Thermodesulfovibrionales
acsE-acsD-acsC-acsB-acsA- GCA 002451165.1 d Bacteria p Nitrospirota c Thermodesulfovibrionia o Thermodesulfovibrionales
acsB-acsA- GCA 004321925.1 d Bacteria p Nitrospirota c Thermodesulfovibrionia o Thermodesulfovibrionales
acsE-acsD-acsC-acsB-acsA- GCA 012026685.1 d Bacteria p Nitrospirota c Thermodesulfovibrionia o Thermodesulfovibrionales
acsE-acsD-acsC-acsB-acsA- 9X cooC- GCA 003153275.1 d Bacteria p Nitrospirota c Thermodesulfovibrionia o Thermodesulfovibrionales
acsE-acsD-acsC-acsB-acsA- 21X cooC- GCA 013824615.1 d Bacteria p Nitrospirota c Thermodesulfovibrionia o Thermodesulfovibrionales
acsB- GCA 003235715.1 d Bacteria p Nitrospirota c Thermodesulfovibrionia o SZUA-242
cooC2-acsE-acsD-acsC-acsB-acsA- GCA 002299835.1 d Bacteria p Nitrospirota c Thermodesulfovibrionia o Thermodesulfovibrionales
acsD-acsC-acsB-acsA- GCA 002897855.1 d Bacteria p Nitrospirota c Thermodesulfovibrionia o Thermodesulfovibrionales
acsE-acsD-acsC-acsB-acsA- GCF 000714715.1 d Bacteria p Nitrospirota c Thermodesulfovibrionia o Thermodesulfovibrionales
acsE-acsD-acsC-acsB-acsA- GCA 002753685.1 d Bacteria p Nitrospirota c Thermodesulfovibrionia o Thermodesulfovibrionales
acsE-acsD-acsC-acsB-acsA- GCA 002753395.1 d Bacteria p Nitrospirota c Thermodesulfovibrionia o Thermodesulfovibrionales
acsE-acsD-acsC-acsB-acsA- GCF 001541255.1 d Bacteria p Nitrospirota c Thermodesulfovibrionia o Thermodesulfovibrionales
acsE-acsD-acsC-acsB-acsA- GCA 002011745.1 d Bacteria p Nitrospirota c Thermodesulfovibrionia o Thermodesulfovibrionales
acsD-acsC-acsB-acsA- GCA 013151785.1 d Bacteria p Nitrospirota c Thermodesulfovibrionia o Thermodesulfovibrionales
acsE-acsD-acsC-acsB-acsA- GCA 011321725.1 d Bacteria p Nitrospirota c Thermodesulfovibrionia o Thermodesulfovibrionales
cooC2- 5X acsE-acsD-acsC-acsB-acsA- GCA 013151495.1 d Bacteria p Nitrospirota c Thermodesulfovibrionia o Thermodesulfovibrionales
acsB- GCA 002898135.1 d Bacteria p Nitrospirota c Thermodesulfovibrionia o Thermodesulfovibrionales
cooC2-acsE-acsD-acsC-acsB-acsA- GCA 011051565.1 d Bacteria p Nitrospirota c Thermodesulfovibrionia o Thermodesulfovibrionales
cooC2-acsE-acsD-acsC-acsB-acsA- GCA 013152485.1 d Bacteria p Nitrospirota c Thermodesulfovibrionia o Thermodesulfovibrionales
cooC2- 2X acsE-acsD-acsC-acsB- GCA 009773415.1 d Bacteria p Nitrospirota c Thermodesulfovibrionia o Thermodesulfovibrionales
cooC2-acsE-acsD-acsC-acsB- GCA 903822005.1 d Bacteria p Nitrospirota c Thermodesulfovibrionia o Thermodesulfovibrionales
acsC-acsB- GCF 001707915.1 d Bacteria p Nitrospirota c Thermodesulfovibrionia o Thermodesulfovibrionales
acsE-acsD-acsC-acsB- GCF 000423865.1 d Bacteria p Nitrospirota c Thermodesulfovibrionia o Thermodesulfovibrionales
acsE-acsD-acsC-acsB- GCA 002339325.1 d Bacteria p Nitrospirota c Thermodesulfovibrionia o Thermodesulfovibrionales
acsE-acsD-acsC-acsB- GCF 001514535.1 d Bacteria p Nitrospirota c Thermodesulfovibrionia o Thermodesulfovibrionales
acsE-acsD-acsC-acsB- GCA 002878055.1 d Bacteria p Nitrospirota c Thermodesulfovibrionia o Thermodesulfovibrionales
acsE-acsD-acsC-acsB- GCF 000020985.1 d Bacteria p Nitrospirota c Thermodesulfovibrionia o Thermodesulfovibrionales
acsE-acsD-acsC-acsB- GCA 011338125.1 d Bacteria p Nitrospirota c Thermodesulfovibrionia o Thermodesulfovibrionales
metV-metF- 6X cooC2- 1X acsE-acsD-acsC-acsB-acsA- 11X fhs- 2X acsV- GCA 013151235.1 d Bacteria p CG2-30-53-67 c CG2-30-53-67 o CG2-30-53-67
metV-metF- 3X cooC2- 2X acsE-acsD-acsC-acsB-acsA- 14X fhs-acsV- GCA 001873285.1 d Bacteria p CG2-30-53-67 c CG2-30-53-67 o CG2-30-53-67
metF- 4X cooC2- 2X acsE-acsD-acsC-acsB- GCA 003454665.1 d Bacteria p Nitrospirota c UBA9217 o UBA9217
cooC2- 2X acsE-acsD-acsC-acsB- GCA 013334545.1 d Bacteria p Nitrospirota c UBA9217 o UBA9217
folD- 7X metV-metF- 4X cooC2- 2X acsE-acsD-acsC-acsB- 23X fhs- GCA 001805055.1 d Bacteria p Nitrospirota c UBA9217 o UBA9217
folD metF metV 4X fhs cooC acsA-cooF- 3X acsB-acsC-acsV- 1X acsD- 1X acsE- GCA 001303665.1 d Bacteria p Planctomycetota c Phycisphaerae o SM23-33
acsB-acsC-acsV-cooC2- GCA 003563855.1 d Bacteria p Verrucomicrobiota c Kiritimatiellae o B70-G9
cooC acsA-cooF-acsB-acsC-acsV- 1X acsD-acsE- GCA 011050235.1 d Bacteria p Planctomycetota c Phycisphaerae o SM23-33
acsA-cooF-acsB- GCA 007131925.1 d Bacteria p Planctomycetota c DG-23 o SLPQ01
cooC acsA-cooF-acsB-acsC-acsV- 1X acsD-acsE- GCA 903929765.1 d Bacteria p Planctomycetota c Phycisphaerae o FEN-1346
cooC acsA-cooF- 7X acsB-acsC-acsV- 1X acsD-acsE- GCA 011682385.1 d Bacteria p Planctomycetota c Phycisphaerae o JAACET01
cooC 1X acsA-cooF- 7X acsB-acsC-acsV- 1X acsD-acsE- GCA 011682535.1 d Bacteria p Hydrogenedentota c Hydrogenedentia o Hydrogenedentiales
acsB- GCA 903932505.1 d Bacteria p Hydrogenedentota c Hydrogenedentia o Hydrogenedentiales
cooC-acsA-acsB-acsC-acsV-cooC2-acsD-acsE- 3X fhs- GCA 007119795.1 d Bacteria p Planctomycetota c Planctomycetes o Pirellulales
cooC-acsA-cooF- 11X acsB-acsC-acsV- 2X acsD-acsE- GCA 011367845.1 d Bacteria p Planctomycetota c Planctomycetes o Pirellulales
cooC-acsA-acsB-acsC-acsV-cooC2-acsD-acsE- 3X fhs- GCA 013360685.1 d Bacteria p Planctomycetota c Planctomycetes o Pirellulales
cooC-acsA-acsB-acsC-acsV-cooC2-acsD-acsE- 3X fhs- GCA 003561895.1 d Bacteria p Planctomycetota c Planctomycetes o Pirellulales
acsB-acsC- GCA 007122045.1 d Bacteria p Planctomycetota c Planctomycetes o Pirellulales
cooC-acsA-cooF-acsB-acsC-acsV- GCA 003551305.1 d Bacteria p Planctomycetota c Brocadiae o SM23-32
cooF acsA cooC 4X acsB-acsC- GCA 001303885.1 d Bacteria p Planctomycetota c Brocadiae o SM23-32
cooF-acsB- GCA 003551565.1 d Bacteria p Planctomycetota c Brocadiae o SM23-32
acsB-acsC- GCA 003567495.1 d Bacteria p Planctomycetota c Brocadiae o SM23-32
cooC-acsA-cooF-acsB-acsC- GCA 003551565.1 d Bacteria p Planctomycetota c Brocadiae o SM23-32
fhs- 10X folD-metV-metF-acsA-cooF-acsB-acsC-acsV-acsD-acsE- GCA 003598055.1 d Bacteria p Abyssubacteria c SURF-5 o SURF-5
fhs- 10X folD-metV-metF-acsA-cooF-acsB-acsC-acsV-acsD-acsE- GCA 003598085.1 d Bacteria p Abyssubacteria c SURF-5 o SURF-5
acsA- 7X acsB-acsC-acsV-acsD-acsE- 1X cooC-cooC2- GCA 003170595.1 d Bacteria p Armatimonadota c UBA5377 o UBA5377
folD- 1X metF-acsB- GCA 002796135.1 d Bacteria p Omnitrophota c Koll11 o GIF10
fchA-folD-metF- 1X acsB- GCA 903852325.1 d Bacteria p Omnitrophota c Koll11 o GIF10
folD-metF-acsB- GCA 002441245.1 d Bacteria p Omnitrophota c Koll11 o GIF10
fchA-folD- 3X metF- 1X acsB- GCA 002774445.1 d Bacteria p Omnitrophota c Koll11 o GIF10
folD-metF- 1X acsB- GCA 002440965.1 d Bacteria p Omnitrophota c Koll11 o GIF10
fhs- 1X folD-metF- 2X acsB- GCA 003644385.1 d Bacteria p Omnitrophota c Koll11 o GIF10
fchA-folD-metF- 2X acsB- GCA 001805465.1 d Bacteria p Omnitrophota c Koll11 o GIF10
folD- 1X metF- 1X acsB- GCA 011370105.1 d Bacteria p Omnitrophota c Koll11 o GIF10
folD-metF- 2X acsB- GCA 003598025.1 d Bacteria p Omnitrophota c Koll11 o GIF10
fhs- 1X folD- 1X metF- 2X acsB- GCA 011620275.1 d Bacteria p Omnitrophota c Koll11 o GIF10
folD- 1X metF- 2X acsB- GCA 002418595.1 d Bacteria p Omnitrophota c Koll11 o GIF10
gcsH- 13X fhs-fchA-folD-metF- 3X acsB- GCA 003451585.1 d Bacteria p Omnitrophota c Koll11 o UBA1572
fhs-fchA-folD-metF- 2X acsB- GCA 001873995.1 d Bacteria p Omnitrophota c Koll11 o UBA1572
fhs- 1X folD-metF- 2X acsB- GCF 004102945.1 d Bacteria p Omnitrophota c Koll11 o UBA1572
fhs- 1X folD-metF- 2X acsB- GCA 002421805.1 d Bacteria p Omnitrophota c Koll11 o UBA1572
acsB-metV- GCA 002796125.1 d Bacteria p Omnitrophota c Koll11 o 1-14-0-20-42-13
acsB- GCA 003485765.1 d Bacteria p Omnitrophota c Koll11 o UBA10183
fhs-fchA-folD-metF-acsB-acsC-acsV-cooC2-acsD-acsE- 3X acsE- GCA 903843285.1 d Bacteria p Omnitrophota c Koll11 o UBA1560
fhs-fchA-folD-metF-acsB-acsC-acsV-cooC2-acsD-acsE- 4X acsE- GCA 003142795.1 d Bacteria p Omnitrophota c Koll11 o UBA1560
metF-acsB- GCA 003140915.1 d Bacteria p Omnitrophota c Koll11 o UBA1560
acsB- 4X acsC- 3X acsD- 2X fhs-fchA-folD- GCA 002402375.1 d Bacteria p Elusimicrobiota c UBA8919 o UBA8919
acsB-acsD-acsC-acsV- GCA 013203665.1 d Bacteria p Cloacimonadota c Cloacimonadia o Cloacimonadales

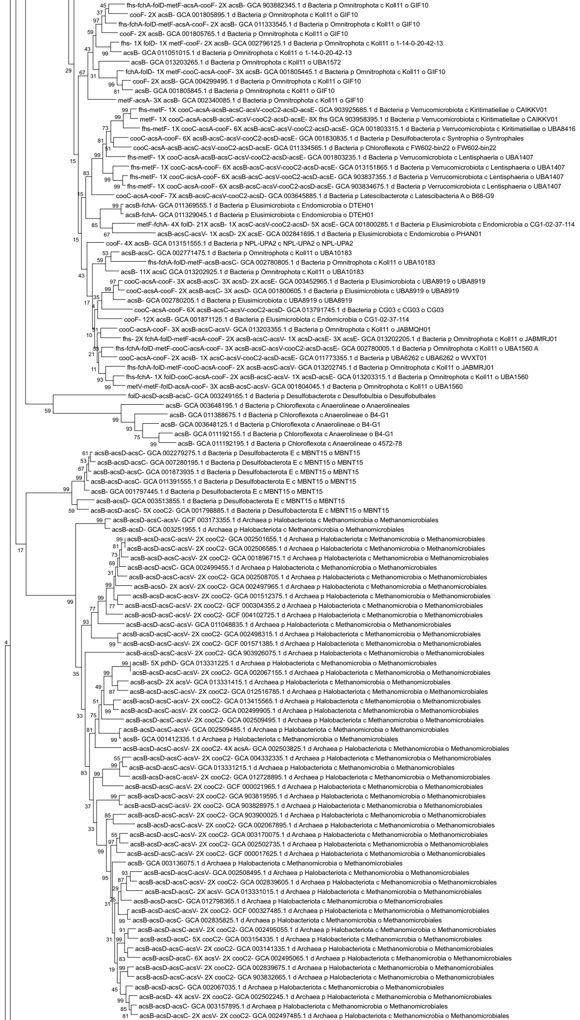
fhs-fchA-folD-metF-acsA-cooF- 2X acsB- GCA 903882345.1 d Bacteria p Omnitrophota c Koll11 o GIF10
cooF- 2X acsB- GCA 001805895.1 d Bacteria p Omnitrophota c Koll11 o GIF10
fhs-fchA-folD-metF-acsA-cooF- 2X acsB- GCA 011333545.1 d Bacteria p Omnitrophota c Koll11 o GIF10
cooF- 2X acsB- GCA 001805765.1 d Bacteria p Omnitrophota c Koll11 o GIF10
fhs- 1X folD- 1X metF-cooF- 2X acsB- GCA 002796125.1 d Bacteria p Omnitrophota c Koll11 o 1-14-0-20-42-13
acsB- GCA 011051015.1 d Bacteria p Omnitrophota c Koll11 o 1-14-0-20-42-13
acsB- GCA 013203265.1 d Bacteria p Omnitrophota c Koll11 o UBA1572
fchA-folD- 1X metF-cooC-acsA-cooF- 3X acsB- GCA 001805445.1 d Bacteria p Omnitrophota c Koll11 o GIF10
cooF- 2X acsB- GCA 004299495.1 d Bacteria p Omnitrophota c Koll11 o GIF10
acsB- GCA 001805845.1 d Bacteria p Omnitrophota c Koll11 o GIF10
metF-acsA- 3X acsB- GCA 002340085.1 d Bacteria p Omnitrophota c Koll11 o GIF10
fhs-metF- 1X cooC-acsA-acsB-acsC-acsV-cooC2-acsD-acsE- GCA 903925685.1 d Bacteria p Verrucomicrobiota c Kiritimatiellae o CAIKKV01
metF- 1X cooC-acsA-acsB-acsC-acsV-cooC2-acsD-acsE- 8X fhs GCA 903958395.1 d Bacteria p Verrucomicrobiota c Kiritimatiellae o CAIKKV01
fhs-metF- 1X cooC-acsA-cooF- 6X acsB-acsC-acsV-cooC2-acsD-acsE- GCA 001803315.1 d Bacteria p Verrucomicrobiota c Kiritimatiellae o UBA8416
cooC-acsA-cooF- 6X acsB-acsC-acsV-cooC2-acsD-acsE- GCA 001830835.1 d Bacteria p Desulfobacterota c Syntrophia o Syntrophales
cooC-acsA-acsB-acsC-acsV-cooC2-acsD-acsE- GCA 011334565.1 d Bacteria p Chloroflexota c FW602-bin22 o FW602-bin22
fhs-metF- 1X cooC-acsA-acsB-acsC-acsV-cooC2-acsD-acsE- GCA 001803235.1 d Bacteria p Verrucomicrobiota c Lentisphaeria o UBA1407
fhs-metF- 1X cooC-acsA-cooF- 6X acsB-acsC-acsV-cooC2-acsD-acsE- GCA 013151865.1 d Bacteria p Verrucomicrobiota c Lentisphaeria o UBA1407
fhs-metF- 1X cooC-acsA-cooF- 6X acsB-acsC-acsV-cooC2-acsD-acsE- GCA 903837355.1 d Bacteria p Verrucomicrobiota c Lentisphaeria o UBA1407
fhs-metF- 1X cooC-acsA-cooF- 6X acsB-acsC-acsV-cooC2-acsD-acsE- GCA 903834675.1 d Bacteria p Verrucomicrobiota c Lentisphaeria o UBA1407
cooC-acsA-cooF- 7X acsB-acsC-acsV-cooC2-acsD- GCA 003645885.1 d Bacteria p Latescibacterota c Latescibacteria A o B68-G9
acsB-fchA- GCA 011369555.1 d Bacteria p Elusimicrobiota c Endomicrobia o DTEH01
acsB-fchA- GCA 011329045.1 d Bacteria p Elusimicrobiota c Endomicrobia o DTEH01
metF-fchA- 4X folD- 21X acsB- 1X acsC-acsV-cooC2-acsD- 5X acsE- GCA 001800285.1 d Bacteria p Elusimicrobiota c Endomicrobia o CG1-02-37-114
acsB-acsC-acsV- 1X acsD- 2X acsE- GCA 002841695.1 d Bacteria p Elusimicrobiota c Endomicrobia o PHAN01
cooF- 4X acsB- GCA 013151555.1 d Bacteria p NPL-UPA2 c NPL-UPA2 o NPL-UPA2
acsB-acsC- GCA 002771475.1 d Bacteria p Omnitrophota c Koll11 o UBA10183
fhs-fchA-folD-metF-acsB-acsC- GCA 002780805.1 d Bacteria p Omnitrophota c Koll11 o UBA10183
acsB- 11X acsC GCA 013202925.1 d Bacteria p Omnitrophota c Koll11 o UBA10183
cooC-acsA-cooF- 3X acsB-acsC- 3X acsD- 2X acsE- GCA 003452965.1 d Bacteria p Elusimicrobiota c UBA8919 o UBA8919
cooC-acsA-cooF- 2X acsB-acsC- 3X acsD- GCA 001800605.1 d Bacteria p Elusimicrobiota c UBA8919 o UBA8919
acsB- GCA 002780205.1 d Bacteria p Elusimicrobiota c UBA8919 o UBA8919
cooC-acsA-cooF- 6X acsB-acsC-acsV-cooC2-acsD- GCA 013791745.1 d Bacteria p CG03 c CG03 o CG03
cooF- 12X acsB- GCA 001871125.1 d Bacteria p Elusimicrobiota c Endomicrobia o CG1-02-37-114
cooC-acsA-cooF- 3X acsB-acsC-acsV- GCA 013203355.1 d Bacteria p Omnitrophota c Koll11 o JABMQH01
fhs- 2X fchA-folD-metF-acsA-cooF- 2X acsB-acsC-acsV- 1X acsD-acsE- 3X acsE- GCA 013202205.1 d Bacteria p Omnitrophota c Koll11 o JABMRJ01
fhs-fchA-folD-metF-cooC-acsA-cooF- 3X acsB-acsC-acsV-cooC2-acsD-acsE- GCA 002780005.1 d Bacteria p Omnitrophota c Koll11 o UBA1560 A
cooC-acsA-cooF- 2X acsB- 1X acsC-acsV-cooC2-acsD-acsE- GCA 011773355.1 d Bacteria p UBA6262 c UBA6262 o WVXT01
fhs-fchA-folD-metF-cooC-acsA-cooF- 2X acsB-acsC-acsV- GCA 013202745.1 d Bacteria p Omnitrophota c Koll11 o JABMRJ01
fhs-fchA- 1X folD-cooC-acsA-cooF- 2X acsB-acsC-acsV- 1X acsD-acsE- GCA 013203315.1 d Bacteria p Omnitrophota c Koll11 o UBA1560
metV-metF-folD-acsA-cooF- 3X acsB-acsC-acsV- GCA 001804045.1 d Bacteria p Omnitrophota c Koll11 o UBA1560
folD-acsD-acsB-acsC- GCA 003249165.1 d Bacteria p Desulfobacterota c Desulfobulbia o Desulfobulbales
acsB- GCA 003648195.1 d Bacteria p Chloroflexota c Anaerolineae o Anaerolineales
acsB- GCA 011388675.1 d Bacteria p Chloroflexota c Anaerolineae o B4-G1
acsB- GCA 003648125.1 d Bacteria p Chloroflexota c Anaerolineae o B4-G1
acsB- GCA 011192155.1 d Bacteria p Chloroflexota c Anaerolineae o B4-G1
acsB- GCA 011192195.1 d Bacteria p Chloroflexota c Anaerolineae o 4572-78
acsB-acsD-acsC- GCA 002279275.1 d Bacteria p Desulfobacterota E c MBNT15 o MBNT15
acsB-acsD-acsC- GCA 007280195.1 d Bacteria p Desulfobacterota E c MBNT15 o MBNT15
acsB-acsD-acsC- GCA 001873935.1 d Bacteria p Desulfobacterota E c MBNT15 o MBNT15
acsB-acsD-acsC- GCA 011391555.1 d Bacteria p Desulfobacterota E c MBNT15 o MBNT15
acsB- GCA 001797445.1 d Bacteria p Desulfobacterota E c MBNT15 o MBNT15
acsB-acsD- GCA 003513855.1 d Bacteria p Desulfobacterota E c MBNT15 o MBNT15
acsB-acsD-acsC- 5X cooC2- GCA 001798885.1 d Bacteria p Desulfobacterota E c MBNT15 o MBNT15
acsB-acsD-acsC-acsV- GCF 003173355.1 d Archaea p Halobacteriota c Methanomicrobia o Methanomicrobiales
acsB-acsD- GCA 003251955.1 d Archaea p Halobacteriota c Methanomicrobia o Methanomicrobiales
acsB-acsD-acsC-acsV- 2X cooC2- GCA 002501655.1 d Archaea p Halobacteriota c Methanomicrobia o Methanomicrobiales
acsB-acsD-acsC-acsV- 2X cooC2- GCA 002506585.1 d Archaea p Halobacteriota c Methanomicrobia o Methanomicrobiales
acsB-acsD-acsC-acsV- 2X cooC2- GCA 001896715.1 d Archaea p Halobacteriota c Methanomicrobia o Methanomicrobiales
acsB-acsD-acsC- GCA 002499455.1 d Archaea p Halobacteriota c Methanomicrobia o Methanomicrobiales
acsB-acsD-acsC-acsV- 2X cooC2- GCA 002508705.1 d Archaea p Halobacteriota c Methanomicrobia o Methanomicrobiales
acsB-acsD- 2X acsV- 2X cooC2- GCA 002497965.1 d Archaea p Halobacteriota c Methanomicrobia o Methanomicrobiales
acsB-acsD-acsC-acsV- 2X cooC2- GCA 001512375.1 d Archaea p Halobacteriota c Methanomicrobia o Methanomicrobiales
acsB-acsD-acsC-acsV- 2X cooC2- GCF 000304355.2 d Archaea p Halobacteriota c Methanomicrobia o Methanomicrobiales
acsB-acsD-acsC-acsV- 2X cooC2- GCF 004102725.1 d Archaea p Halobacteriota c Methanomicrobia o Methanomicrobiales
acsB-acsD-acsC-acsV- GCA 011048835.1 d Archaea p Halobacteriota c Methanomicrobia o Methanomicrobiales
acsB-acsD-acsC-acsV- 2X cooC2- GCA 002498315.1 d Archaea p Halobacteriota c Methanomicrobia o Methanomicrobiales
acsB-acsD-acsC-acsV- 2X cooC2- GCF 001571385.1 d Archaea p Halobacteriota c Methanomicrobia o Methanomicrobiales
acsB-acsD-acsC-acsV- 2X cooC2- GCA 903926075.1 d Archaea p Halobacteriota c Methanomicrobia o Methanomicrobiales
acsB- 5X pdhD- GCA 013331225.1 d Archaea p Halobacteriota c Methanomicrobia o Methanomicrobiales
acsB-acsD-acsC-acsV- 2X cooC2- GCA 002067155.1 d Archaea p Halobacteriota c Methanomicrobia o Methanomicrobiales
acsB-acsD- 2X acsV- GCA 013331415.1 d Archaea p Halobacteriota c Methanomicrobia o Methanomicrobiales
acsB-acsD-acsC-acsV- 2X cooC2- GCA 012516785.1 d Archaea p Halobacteriota c Methanomicrobia o Methanomicrobiales
acsB-acsD-acsC-acsV- 2X cooC2- GCA 013415565.1 d Archaea p Halobacteriota c Methanomicrobia o Methanomicrobiales
acsB-acsD-acsC-acsV- 2X cooC2- GCA 002499905.1 d Archaea p Halobacteriota c Methanomicrobia o Methanomicrobiales
acsB-acsD-acsC-acsV- 2X cooC2- GCA 002509495.1 d Archaea p Halobacteriota c Methanomicrobia o Methanomicrobiales
acsB-acsD-acsC-acsV- GCA 002509485.1 d Archaea p Halobacteriota c Methanomicrobia o Methanomicrobiales
acsB- GCA 001412335.1 d Archaea p Halobacteriota c Methanomicrobia o Methanomicrobiales
acsB-acsD-acsC-acsV- 2X cooC2- 4X acsA- GCA 002503825.1 d Archaea p Halobacteriota c Methanomicrobia o Methanomicrobiales
acsB-acsD-acsC-acsV- 2X cooC2- GCA 004332335.1 d Archaea p Halobacteriota c Methanomicrobia o Methanomicrobiales
acsB-acsD-acsC-acsV- GCA 013331215.1 d Archaea p Halobacteriota c Methanomicrobia o Methanomicrobiales
acsB-acsD-acsC-acsV- 2X cooC2- GCA 012728895.1 d Archaea p Halobacteriota c Methanomicrobia o Methanomicrobiales
acsB-acsD-acsC-acsV- 2X cooC2- GCF 000021965.1 d Archaea p Halobacteriota c Methanomicrobia o Methanomicrobiales
acsB-acsD-acsC-acsV- 2X cooC2- GCA 903819595.1 d Archaea p Halobacteriota c Methanomicrobia o Methanomicrobiales
acsB-acsD-acsC-acsV- 2X cooC2- GCA 903828975.1 d Archaea p Halobacteriota c Methanomicrobia o Methanomicrobiales
acsB-acsD-acsC-acsV- 2X cooC2- GCA 903900025.1 d Archaea p Halobacteriota c Methanomicrobia o Methanomicrobiales
acsB-acsD-acsC-acsV- 2X cooC2- GCA 002067895.1 d Archaea p Halobacteriota c Methanomicrobia o Methanomicrobiales
acsB-acsD-acsC-acsV- 2X cooC2- GCA 003170075.1 d Archaea p Halobacteriota c Methanomicrobia o Methanomicrobiales
acsB-acsD-acsC-acsV- 2X cooC2- GCA 002502735.1 d Archaea p Halobacteriota c Methanomicrobia o Methanomicrobiales
acsB-acsD-acsC-acsV- 2X cooC2- GCF 000017625.1 d Archaea p Halobacteriota c Methanomicrobia o Methanomicrobiales
acsB- GCA 003136075.1 d Archaea p Halobacteriota c Methanomicrobia o Methanomicrobiales
acsB-acsD-acsC-acsV- GCA 002508495.1 d Archaea p Halobacteriota c Methanomicrobia o Methanomicrobiales
acsB-acsD-acsC-acsV- 2X cooC2- GCA 002839605.1 d Archaea p Halobacteriota c Methanomicrobia o Methanomicrobiales
acsB-acsD-acsC- 2X acsV- GCA 013331015.1 d Archaea p Halobacteriota c Methanomicrobia o Methanomicrobiales
acsB-acsD-acsC- GCA 012798365.1 d Archaea p Halobacteriota c Methanomicrobia o Methanomicrobiales
acsB-acsD-acsC-acsV- 2X cooC2- GCF 000327485.1 d Archaea p Halobacteriota c Methanomicrobia o Methanomicrobiales
acsB-acsD-acsC- GCA 002835825.1 d Archaea p Halobacteriota c Methanomicrobia o Methanomicrobiales
acsB-acsD-acsC-acsV- 2X cooC2- GCA 002495055.1 d Archaea p Halobacteriota c Methanomicrobia o Methanomicrobiales
acsB-acsD-acsC- 5X cooC2- GCA 003154335.1 d Archaea p Halobacteriota c Methanomicrobia o Methanomicrobiales
acsB-acsD-acsC-acsV- 2X cooC2- GCA 003141335.1 d Archaea p Halobacteriota c Methanomicrobia o Methanomicrobiales
acsB-acsD-acsC- 6X acsV- 2X cooC2- GCA 002495065.1 d Archaea p Halobacteriota c Methanomicrobia o Methanomicrobiales
acsB-acsD-acsC-acsV- 2X cooC2- GCA 002839675.1 d Archaea p Halobacteriota c Methanomicrobia o Methanomicrobiales
acsB-acsD-acsC-acsV- 2X cooC2- GCA 903832665.1 d Archaea p Halobacteriota c Methanomicrobia o Methanomicrobiales
acsB-acsD-acsC- GCA 002067035.1 d Archaea p Halobacteriota c Methanomicrobia o Methanomicrobiales
acsB-acsD- 4X acsV- 2X cooC2- GCA 002502245.1 d Archaea p Halobacteriota c Methanomicrobia o Methanomicrobiales
acsB-acsD-acsC- GCA 003157895.1 d Archaea p Halobacteriota c Methanomicrobia o Methanomicrobiales
acsB-acsD-acsC- 2X acsV- 2X cooC2- GCA 002497485.1 d Archaea p Halobacteriota c Methanomicrobia o Methanomicrobiales

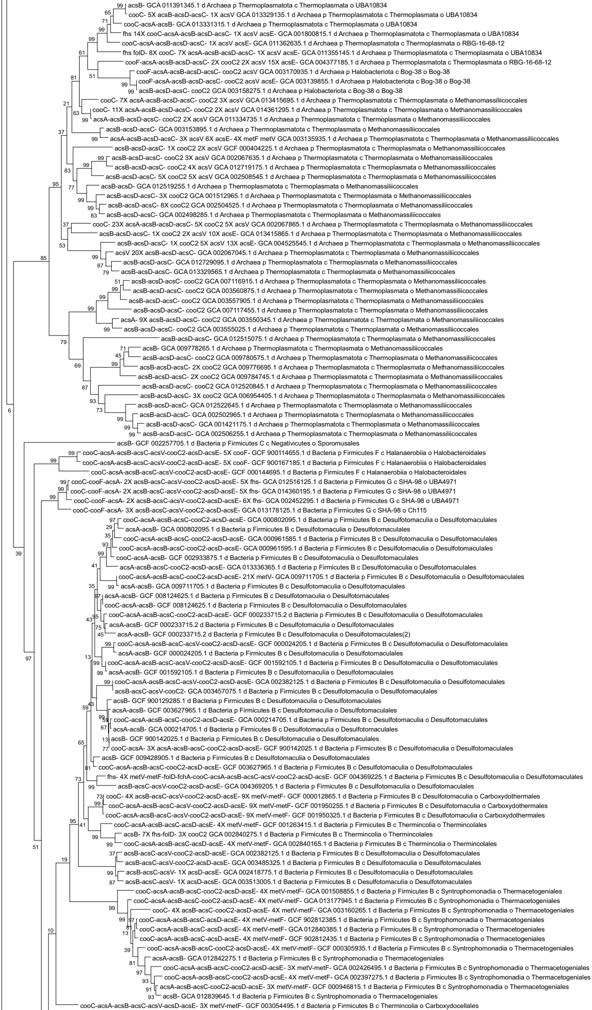
acsB- GCA 011391345.1 d Archaea p Thermoplasmatota c Thermoplasmata o UBA10834
cooC- 5X acsB-acsD-acsC- 1X acsV GCA 013329135.1 d Archaea p Thermoplasmatota c Thermoplasmata o UBA10834
cooC-acsA-acsB- GCA 013331315.1 d Archaea p Thermoplasmatota c Thermoplasmata o UBA10834
fhs 14X cooC-acsA-acsB-acsD-acsC- 1X acsV acsE- GCA 001800815.1 d Archaea p Thermoplasmatota c Thermoplasmata o UBA10834
cooC-acsA-acsB-acsD-acsC- 1X acsV acsE- GCA 011362635.1 d Archaea p Thermoplasmatota c Thermoplasmata o RBG-16-68-12
fhs folD- 8X cooC- 7X acsA-acsB-acsD-acsC- 1X acsV acsE- GCA 011355145.1 d Archaea p Thermoplasmatota c Thermoplasmata o UBA10834
cooF-acsA-acsB-acsD-acsC- 2X cooC2 2X acsV 15X acsE- GCA 004377185.1 d Archaea p Thermoplasmatota c Thermoplasmata o RBG-16-68-12
cooF-acsA-acsB-acsD-acsC- cooC2 acsV GCA 003170935.1 d Archaea p Halobacteriota c Bog-38 o Bog-38
cooF-acsA-acsB-acsD-acsC- cooC2 acsV acsE- GCA 003139855.1 d Archaea p Halobacteriota c Bog-38 o Bog-38
acsB-acsD-acsC- cooC2 GCA 003158275.1 d Archaea p Halobacteriota c Bog-38 o Bog-38
cooC- 7X acsA-acsB-acsD-acsC- cooC2 3X acsV GCA 013415695.1 d Archaea p Thermoplasmatota c Thermoplasmata o Methanomassiliicoccales
cooC- 11X acsA-acsB-acsD-acsC- cooC2 2X acsV GCA 014361295.1 d Archaea p Thermoplasmatota c Thermoplasmata o Methanomassiliicoccales
acsA-acsB-acsD-acsC- cooC2 2X acsV GCA 011334735.1 d Archaea p Thermoplasmatota c Thermoplasmata o Methanomassiliicoccales
acsB-acsD-acsC- GCA 003153895.1 d Archaea p Thermoplasmatota c Thermoplasmata o Methanomassiliicoccales
acsA-acsB-acsD-acsC- 3X acsV 6X acsE- 4X metF metV GCA 003135935.1 d Archaea p Thermoplasmatota c Thermoplasmata o Methanomassiliicoccales
acsB-acsD-acsC- 1X cooC2 2X acsV GCF 000404225.1 d Archaea p Thermoplasmatota c Thermoplasmata o Methanomassiliicoccales
acsB-acsD-acsC- cooC2 3X acsV GCA 002067635.1 d Archaea p Thermoplasmatota c Thermoplasmata o Methanomassiliicoccales
acsB-acsD-acsC- cooC2 4X acsV GCA 012719175.1 d Archaea p Thermoplasmatota c Thermoplasmata o Methanomassiliicoccales
acsB-acsD-acsC- 5X cooC2 5X acsV GCA 002508545.1 d Archaea p Thermoplasmatota c Thermoplasmata o Methanomassiliicoccales
acsB-acsD- GCA 012519255.1 d Archaea p Thermoplasmatota c Thermoplasmata o Methanomassiliicoccales
acsB-acsD-acsC- 3X cooC2 GCA 001512965.1 d Archaea p Thermoplasmatota c Thermoplasmata o Methanomassiliicoccales
acsB-acsD-acsC- 8X cooC2 GCA 002504525.1 d Archaea p Thermoplasmatota c Thermoplasmata o Methanomassiliicoccales
acsB-acsD-acsC- GCA 002498285.1 d Archaea p Thermoplasmatota c Thermoplasmata o Methanomassiliicoccales
cooC- 23X acsA-acsB-acsD-acsC- 5X cooC2 5X acsV GCA 002067865.1 d Archaea p Thermoplasmatota c Thermoplasmata o Methanomassiliicoccales
acsB-acsD-acsC- 1X cooC2 2X acsV 10X acsE- GCA 013415865.1 d Archaea p Thermoplasmatota c Thermoplasmata o Methanomassiliicoccales
acsB-acsD-acsC- 1X cooC2 5X acsV 13X acsE- GCA 004525545.1 d Archaea p Thermoplasmatota c Thermoplasmata o Methanomassiliicoccales
acsV 20X acsB-acsD-acsC- GCA 002067045.1 d Archaea p Thermoplasmatota c Thermoplasmata o Methanomassiliicoccales
acsB-acsD-acsC- GCA 012729095.1 d Archaea p Thermoplasmatota c Thermoplasmata o Methanomassiliicoccales
acsB-acsD-acsC- GCA 013329565.1 d Archaea p Thermoplasmatota c Thermoplasmata o Methanomassiliicoccales
acsB-acsD-acsC- cooC2 GCA 007116915.1 d Archaea p Thermoplasmatota c Thermoplasmata o Methanomassiliicoccales
acsB-acsD-acsC- cooC2 GCA 003560875.1 d Archaea p Thermoplasmatota c Thermoplasmata o Methanomassiliicoccales
acsB-acsD-acsC- cooC2 GCA 003557905.1 d Archaea p Thermoplasmatota c Thermoplasmata o Methanomassiliicoccales
acsB-acsD-acsC- cooC2 GCA 007117455.1 d Archaea p Thermoplasmatota c Thermoplasmata o Methanomassiliicoccales
acsA- 9X acsB-acsD-acsC- cooC2 GCA 003550345.1 d Archaea p Thermoplasmatota c Thermoplasmata o Methanomassiliicoccales
acsB-acsD-acsC- cooC2 GCA 003555025.1 d Archaea p Thermoplasmatota c Thermoplasmata o Methanomassiliicoccales
acsB-acsD-acsC- GCA 012515075.1 d Archaea p Thermoplasmatota c Thermoplasmata o Methanomassiliicoccales
acsB- GCA 009778265.1 d Archaea p Thermoplasmatota c Thermoplasmata o Methanomassiliicoccales
acsB-acsD-acsC- cooC2 GCA 009780575.1 d Archaea p Thermoplasmatota c Thermoplasmata o Methanomassiliicoccales
acsB-acsD-acsC- 2X cooC2 GCA 009776695.1 d Archaea p Thermoplasmatota c Thermoplasmata o Methanomassiliicoccales
acsB-acsD-acsC- 2X cooC2 GCA 009784745.1 d Archaea p Thermoplasmatota c Thermoplasmata o Methanomassiliicoccales
acsB-acsD-acsC- cooC2 GCA 012520845.1 d Archaea p Thermoplasmatota c Thermoplasmata o Methanomassiliicoccales
acsB-acsD-acsC- 3X cooC2 GCA 006954405.1 d Archaea p Thermoplasmatota c Thermoplasmata o Methanomassiliicoccales
acsB-acsD-acsC- GCA 012522645.1 d Archaea p Thermoplasmatota c Thermoplasmata o Methanomassiliicoccales
acsB-acsD-acsC- GCA 002502965.1 d Archaea p Thermoplasmatota c Thermoplasmata o Methanomassiliicoccales
acsB-acsD-acsC- GCA 001421175.1 d Archaea p Thermoplasmatota c Thermoplasmata o Methanomassiliicoccales
acsB-acsD-acsC- GCA 002506255.1 d Archaea p Thermoplasmatota c Thermoplasmata o Methanomassiliicoccales
acsB- GCF 002257705.1 d Bacteria p Firmicutes C c Negativicutes o Sporomusales
cooC-acsA-acsB-acsC-acsV-cooC2-acsD-acsE- 5X cooF- GCF 900114655.1 d Bacteria p Firmicutes F c Halanaerobiia o Halobacteroidales
cooC-acsA-acsB-acsC-acsV-cooC2-acsD-acsE- 5X cooF- GCF 900167185.1 d Bacteria p Firmicutes F c Halanaerobiia o Halobacteroidales
cooC-acsA-acsB-acsC-acsV-cooC2-acsD-acsE- GCF 000144695.1 d Bacteria p Firmicutes F c Halanaerobiia o Halobacteroidales
cooC-cooF-acsA- 2X acsB-acsC-acsV-cooC2-acsD-acsE- 5X fhs- GCA 012516125.1 d Bacteria p Firmicutes G c SHA-98 o UBA4971
cooC-cooF-acsA- 2X acsB-acsC-acsV-cooC2-acsD-acsE- 5X fhs- GCA 014360195.1 d Bacteria p Firmicutes G c SHA-98 o UBA4971
cooC-cooF-acsA- 2X acsB-acsC-acsV-cooC2-acsD-acsE- 6X fhs- GCA 002452295.1 d Bacteria p Firmicutes G c SHA-98 o UBA4971
cooC-cooF-acsA- 3X acsB-acsC-acsV-cooC2-acsD-acsE- GCA 013178125.1 d Bacteria p Firmicutes G c SHA-98 o Ch115
cooC-acsA-acsB-acsC-cooC2-acsD-acsE- GCA 000802095.1 d Bacteria p Firmicutes B c Desulfotomaculia o Desulfotomaculales
acsA-acsB- GCA 000802095.1 d Bacteria p Firmicutes B c Desulfotomaculia o Desulfotomaculales
cooC-acsA-acsB-acsC-cooC2-acsD-acsE- GCA 000961585.1 d Bacteria p Firmicutes B c Desulfotomaculia o Desulfotomaculales
cooC-acsA-acsB-acsC-cooC2-acsD-acsE- GCA 000961595.1 d Bacteria p Firmicutes B c Desulfotomaculia o Desulfotomaculales
cooC-acsA-acsB- GCF 002933875.1 d Bacteria p Firmicutes B c Desulfotomaculia o Desulfotomaculales
acsA-acsB-acsC-cooC2-acsD-acsE- GCA 013336365.1 d Bacteria p Firmicutes B c Desulfotomaculia o Desulfotomaculales
cooC-acsA-acsB-acsC-cooC2-acsD-acsE- 21X metV- GCA 009711705.1 d Bacteria p Firmicutes B c Desulfotomaculia o Desulfotomaculales
acsA-acsB- GCA 009711705.1 d Bacteria p Firmicutes B c Desulfotomaculia o Desulfotomaculales
acsA-acsB- GCF 008124625.1 d Bacteria p Firmicutes B c Desulfotomaculia o Desulfotomaculales
cooC-acsA-acsB- GCF 008124625.1 d Bacteria p Firmicutes B c Desulfotomaculia o Desulfotomaculales
cooC-acsA-acsB-acsC-cooC2-acsD-acsE- GCF 000233715.2 d Bacteria p Firmicutes B c Desulfotomaculia o Desulfotomaculales
acsA-acsB- GCF 000233715.2 d Bacteria p Firmicutes B c Desulfotomaculia o Desulfotomaculales
acsA-acsB- GCF 000233715.2 d Bacteria p Firmicutes B c Desulfotomaculia o Desulfotomaculales(2)
cooC-acsA-acsB-acsC-acsV-cooC2-acsD-acsE- GCF 000024205.1 d Bacteria p Firmicutes B c Desulfotomaculia o Desulfotomaculales
acsA-acsB- GCF 000024205.1 d Bacteria p Firmicutes B c Desulfotomaculia o Desulfotomaculales
cooC-acsA-acsB-acsC-acsV-cooC2-acsD-acsE- GCF 001592105.1 d Bacteria p Firmicutes B c Desulfotomaculia o Desulfotomaculales
acsA-acsB- GCF 001592105.1 d Bacteria p Firmicutes B c Desulfotomaculia o Desulfotomaculales
cooC-acsA-acsB-acsC-acsV-cooC2-acsD-acsE- GCA 002382125.1 d Bacteria p Firmicutes B c Desulfotomaculia o Desulfotomaculales
acsB-acsC-acsV-cooC2- GCA 003457075.1 d Bacteria p Firmicutes B c Desulfotomaculia o Desulfotomaculales
acsB- GCF 900129285.1 d Bacteria p Firmicutes B c Desulfotomaculia o Desulfotomaculales
acsA-acsB- GCF 003627965.1 d Bacteria p Firmicutes B c Desulfotomaculia o Desulfotomaculales
cooC-acsA-acsB-acsC-cooC2-acsD-acsE- GCA 000214705.1 d Bacteria p Firmicutes B c Desulfotomaculia o Desulfotomaculales
acsA-acsB- GCA 000214705.1 d Bacteria p Firmicutes B c Desulfotomaculia o Desulfotomaculales
acsB- GCF 900142025.1 d Bacteria p Firmicutes B c Desulfotomaculia o Desulfotomaculales
cooC-acsA- 3X acsA-acsB-acsC-cooC2-acsD-acsE- GCF 900142025.1 d Bacteria p Firmicutes B c Desulfotomaculia o Desulfotomaculales
acsB- GCF 009428905.1 d Bacteria p Firmicutes B c Desulfotomaculia o Desulfotomaculales
cooC-acsA-acsB-acsC-cooC2-acsD-acsE- GCF 003627965.1 d Bacteria p Firmicutes B c Desulfotomaculia o Desulfotomaculales
fhs- 4X metV-metF-folD-fchA-cooC-acsA-acsB-acsC-acsV-cooC2-acsD-acsE- GCF 004369225.1 d Bacteria p Firmicutes B c Desulfotomaculia o Desulfotomaculales
acsB-acsC-acsV-cooC2-acsD-acsE- GCA 004369205.1 d Bacteria p Firmicutes B c Desulfotomaculia o Desulfotomaculales
cooC- 4X acsB-acsC-acsV-cooC2-acsD-acsE- 9X metV-metF- GCF 000012865.1 d Bacteria p Firmicutes B c Desulfotomaculia o Carboxydothermales
cooC-acsA-acsB-acsC-acsV-cooC2-acsD-acsE- 9X metV-metF- GCF 001950255.1 d Bacteria p Firmicutes B c Desulfotomaculia o Carboxydothermales
cooC-acsA-acsB-acsC-acsV-cooC2-acsD-acsE- 9X metV-metF- GCF 001950325.1 d Bacteria p Firmicutes B c Desulfotomaculia o Carboxydothermales
cooC-acsA-acsB-acsC-acsD-acsE- 4X metV-metF- GCF 001263415.1 d Bacteria p Firmicutes B c Thermincolia o Thermincolales
acsB- 7X fhs-folD- 3X cooC2 GCA 002840275.1 d Bacteria p Firmicutes B c Thermincolia o Thermincolales
cooC-acsA-acsB-acsC-acsD-acsE- 4X metV-metF- GCA 002840165.1 d Bacteria p Firmicutes B c Thermincolia o Thermincolales
acsB-acsC-acsV-cooC2-acsD-acsE- GCA 002382125.1 d Bacteria p Firmicutes B c Desulfotomaculia o Desulfotomaculales
acsB-acsC-acsV-cooC2-acsD-acsE- GCA 003485325.1 d Bacteria p Firmicutes B c Desulfotomaculia o Desulfotomaculales
acsB-acsC-acsV- 1X acsD-acsE- GCA 002418775.1 d Bacteria p Firmicutes B c Desulfotomaculia o Desulfotomaculales
acsB-acsC-acsV- 1X acsD-acsE- GCA 003513005.1 d Bacteria p Firmicutes B c Desulfotomaculia o Desulfotomaculales
cooC-acsA-acsB-acsC-cooC2-acsD-acsE- 4X metV-metF- GCA 001508855.1 d Bacteria p Firmicutes B c Syntrophomonadia o Thermacetogeniales
cooC-acsA-acsB-acsC-cooC2-acsD-acsE- 4X metV-metF- GCA 013177945.1 d Bacteria p Firmicutes B c Syntrophomonadia o Thermacetogeniales
cooC- 4X acsB-acsC-cooC2-acsD-acsE- 4X metV-metF- GCA 003160265.1 d Bacteria p Firmicutes B c Syntrophomonadia o Thermacetogeniales
cooC-acsA-acsB-acsC-acsD-acsE- 4X metV-metF- GCF 902812385.1 d Bacteria p Firmicutes B c Syntrophomonadia o Thermacetogeniales
cooC-acsA-acsB-acsC-acsD-acsE- 4X metV-metF- GCA 012840385.1 d Bacteria p Firmicutes B c Syntrophomonadia o Thermacetogeniales
cooC-acsA-acsB-acsC-acsD-acsE- 4X metV-metF- GCF 902812435.1 d Bacteria p Firmicutes B c Syntrophomonadia o Thermacetogeniales
cooC-acsA-acsB-acsC-cooC2-acsD-acsE- 4X metV-metF- GCF 000305935.1 d Bacteria p Firmicutes B c Syntrophomonadia o Thermacetogeniales
acsA-acsB- GCA 012842275.1 d Bacteria p Firmicutes B c Syntrophomonadia o Thermacetogeniales
cooC-acsA-acsB-acsC-cooC2-acsD-acsE- 3X metV-metF- GCA 002426495.1 d Bacteria p Firmicutes B c Syntrophomonadia o Thermacetogeniales
cooC-acsA-acsB-acsC-cooC2-acsD-acsE- 4X metV-metF- GCA 002397275.1 d Bacteria p Firmicutes B c Syntrophomonadia o Thermacetogeniales
acsA-acsB-acsC-cooC2-acsD-acsE- 3X metV-metF- GCF 000946815.1 d Bacteria p Firmicutes B c Syntrophomonadia o Thermacetogeniales
acsB- GCA 012839645.1 d Bacteria p Firmicutes B c Syntrophomonadia o Thermacetogeniales
cooC-acsA-acsB-acsC-acsV-acsD-acsE- 3X metV-metF- GCF 003054495.1 d Bacteria p Firmicutes B c Thermincolia o Carboxydocellales

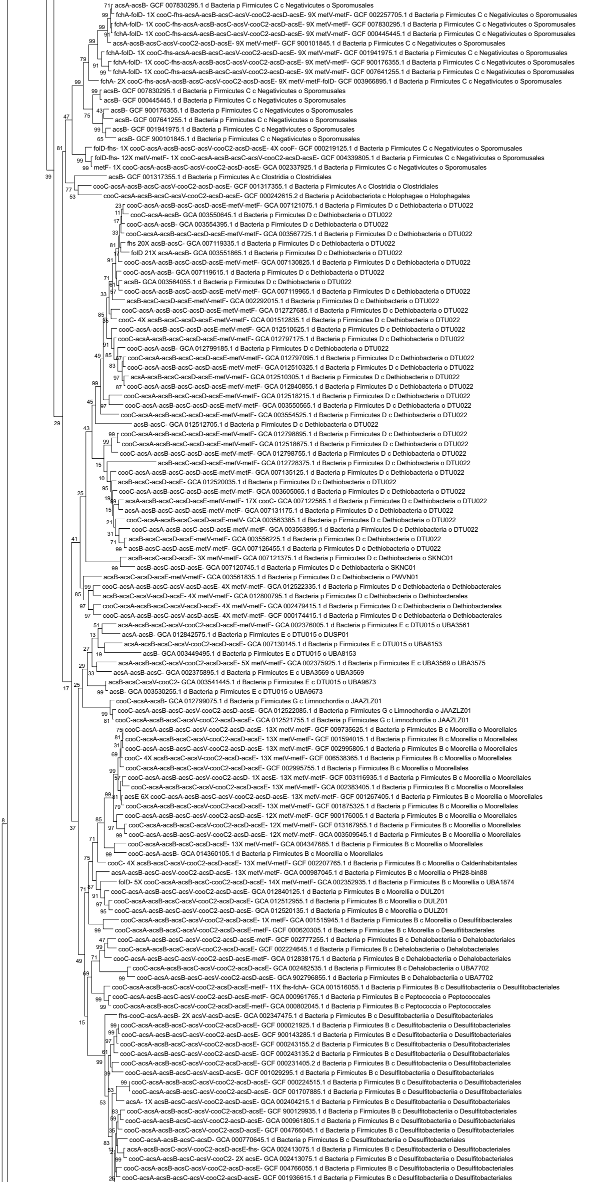
acsA-acsB- GCF 007830295.1 d Bacteria p Firmicutes C c Negativicutes o Sporomusales
fchA-folD- 1X cooC-fhs-acsA-acsB-acsC-acsV-cooC2-acsD-acsE- 9X metV-metF- GCF 002257705.1 d Bacteria p Firmicutes C c Negativicutes o Sporomusales
fchA-folD- 1X cooC-fhs-acsA-acsB-acsC-acsV-cooC2-acsD-acsE- 9X metV-metF- GCF 007830295.1 d Bacteria p Firmicutes C c Negativicutes o Sporomusales
fchA-folD- 1X cooC-fhs-acsA-acsB-acsC-acsV-cooC2-acsD-acsE- 9X metV-metF- GCF 000445445.1 d Bacteria p Firmicutes C c Negativicutes o Sporomusales
acsA-acsB-acsC-acsV-cooC2-acsD-acsE- 9X metV-metF- GCF 900101845.1 d Bacteria p Firmicutes C c Negativicutes o Sporomusales
fchA-folD- 1X cooC-fhs-acsA-acsB-acsC-acsV-cooC2-acsD-acsE- 9X metV-metF- GCF 001941975.1 d Bacteria p Firmicutes C c Negativicutes o Sporomusales
fchA-folD- 1X cooC-fhs-acsA-acsB-acsC-acsV-cooC2-acsD-acsE- 9X metV-metF- GCF 900176355.1 d Bacteria p Firmicutes C c Negativicutes o Sporomusales
fchA-folD- 1X cooC-fhs-acsA-acsB-acsC-acsV-cooC2-acsD-acsE- 9X metV-metF- GCF 007641255.1 d Bacteria p Firmicutes C c Negativicutes o Sporomusales
fchA- 2X cooC-fhs-acsA-acsB-acsC-acsV-cooC2-acsD-acsE- 9X metV-metF-folD- GCF 003966895.1 d Bacteria p Firmicutes C c Negativicutes o Sporomusales
acsB- GCF 007830295.1 d Bacteria p Firmicutes C c Negativicutes o Sporomusales
acsB- GCF 000445445.1 d Bacteria p Firmicutes C c Negativicutes o Sporomusales
acsB- GCF 900176355.1 d Bacteria p Firmicutes C c Negativicutes o Sporomusales
acsB- GCF 007641255.1 d Bacteria p Firmicutes C c Negativicutes o Sporomusales
acsB- GCF 001941975.1 d Bacteria p Firmicutes C c Negativicutes o Sporomusales
acsB- GCF 900101845.1 d Bacteria p Firmicutes C c Negativicutes o Sporomusales
folD-fhs- 1X cooC-acsA-acsB-acsC-acsV-cooC2-acsD-acsE- 4X cooF- GCF 000219125.1 d Bacteria p Firmicutes C c Negativicutes o Sporomusales
folD-fhs- 12X metV-metF- 1X cooC-acsA-acsB-acsC-acsV-cooC2-acsD-acsE- GCF 004339805.1 d Bacteria p Firmicutes C c Negativicutes o Sporomusales
metF- 1X cooC-acsA-acsB-acsC-acsV-cooC2-acsD-acsE- GCA 002337925.1 d Bacteria p Firmicutes C c Negativicutes o Sporomusales
acsB- GCF 001317355.1 d Bacteria p Firmicutes A c Clostridia o Clostridiales
cooC-acsA-acsB-acsC-acsV-cooC2-acsD-acsE- GCF 001317355.1 d Bacteria p Firmicutes A c Clostridia o Clostridiales
cooC-acsA-acsB-acsC-acsV-cooC2-acsD-acsE- GCF 000242615.2 d Bacteria p Acidobacteriota c Holophagae o Holophagales
cooC-acsA-acsB-acsC-acsD-acsE-metV-metF- GCA 007121075.1 d Bacteria p Firmicutes D c Dethiobacteria o DTU022
cooC-acsA-acsB- GCA 003550645.1 d Bacteria p Firmicutes D c Dethiobacteria o DTU022
cooC-acsA-acsB- GCA 003554395.1 d Bacteria p Firmicutes D c Dethiobacteria o DTU022
cooC-acsA-acsB-acsC-acsD-acsE-metV-metF- GCA 003567725.1 d Bacteria p Firmicutes D c Dethiobacteria o DTU022
fhs 20X acsB-acsC- GCA 007119335.1 d Bacteria p Firmicutes D c Dethiobacteria o DTU022
folD 21X acsA-acsB- GCA 003551865.1 d Bacteria p Firmicutes D c Dethiobacteria o DTU022
cooC-acsA-acsB-acsC-acsD-acsE-metV-metF- GCA 007130825.1 d Bacteria p Firmicutes D c Dethiobacteria o DTU022
cooC-acsA-acsB- GCA 007119615.1 d Bacteria p Firmicutes D c Dethiobacteria o DTU022
acsB- GCA 003564055.1 d Bacteria p Firmicutes D c Dethiobacteria o DTU022
cooC-acsA-acsB-acsC-acsD-acsE-metV-metF- GCA 007119965.1 d Bacteria p Firmicutes D c Dethiobacteria o DTU022
acsB-acsC-acsD-acsE-metV-metF- GCA 002292015.1 d Bacteria p Firmicutes D c Dethiobacteria o DTU022
cooC-acsA-acsB-acsC-acsD-acsE-metV-metF- GCA 012727685.1 d Bacteria p Firmicutes D c Dethiobacteria o DTU022
cooC- 4X acsB-acsC-acsD-acsE-metV-metF- GCA 001512835.1 d Bacteria p Firmicutes D c Dethiobacteria o DTU022
cooC-acsA-acsB-acsC-acsD-acsE-metV-metF- GCA 012510625.1 d Bacteria p Firmicutes D c Dethiobacteria o DTU022
cooC-acsA-acsB-acsC-acsD-acsE-metV-metF- GCA 012797175.1 d Bacteria p Firmicutes D c Dethiobacteria o DTU022
cooC-acsA-acsB- GCA 012799185.1 d Bacteria p Firmicutes D c Dethiobacteria o DTU022
cooC-acsA-acsB-acsC-acsD-acsE-metV-metF- GCA 012797095.1 d Bacteria p Firmicutes D c Dethiobacteria o DTU022
cooC-acsA-acsB-acsC-acsD-acsE-metV-metF- GCA 012510325.1 d Bacteria p Firmicutes D c Dethiobacteria o DTU022
acsA-acsB-acsC-acsD-acsE-metV-metF- GCA 012510305.1 d Bacteria p Firmicutes D c Dethiobacteria o DTU022
cooC-acsA-acsB-acsC-acsD-acsE-metV-metF- GCA 012840855.1 d Bacteria p Firmicutes D c Dethiobacteria o DTU022
cooC-acsA-acsB-acsC-acsD-acsE-metV-metF- GCA 012518215.1 d Bacteria p Firmicutes D c Dethiobacteria o DTU022
cooC-acsA-acsB-acsC-acsD-acsE-metV-metF- GCA 003550565.1 d Bacteria p Firmicutes D c Dethiobacteria o DTU022
cooC-acsA-acsB-acsC-acsD-acsE-metV-metF- GCA 003554525.1 d Bacteria p Firmicutes D c Dethiobacteria o DTU022
acsB-acsC- GCA 012512705.1 d Bacteria p Firmicutes D c Dethiobacteria o DTU022
cooC-acsA-acsB-acsC-acsD-acsE-metV-metF- GCA 012798895.1 d Bacteria p Firmicutes D c Dethiobacteria o DTU022
cooC-acsA-acsB-acsC-acsD-acsE-metV-metF- GCA 012518675.1 d Bacteria p Firmicutes D c Dethiobacteria o DTU022
cooC-acsA-acsB-acsC-acsD-acsE-metV-metF- GCA 012798755.1 d Bacteria p Firmicutes D c Dethiobacteria o DTU022
acsB-acsC-acsD-acsE-metV-metF- GCA 012728375.1 d Bacteria p Firmicutes D c Dethiobacteria o DTU022
cooC-acsA-acsB-acsC-acsD-acsE-metV-metF- GCA 007135125.1 d Bacteria p Firmicutes D c Dethiobacteria o DTU022
acsB-acsC-acsD-acsE- GCA 012520035.1 d Bacteria p Firmicutes D c Dethiobacteria o DTU022
cooC-acsA-acsB-acsC-acsD-acsE-metV-metF- GCA 003605065.1 d Bacteria p Firmicutes D c Dethiobacteria o DTU022
acsA-acsB-acsC-acsD-acsE-metV-metF- 17X cooC- GCA 007122565.1 d Bacteria p Firmicutes D c Dethiobacteria o DTU022
acsA-acsB-acsC-acsD-acsE-metV-metF- GCA 007131175.1 d Bacteria p Firmicutes D c Dethiobacteria o DTU022
cooC-acsA-acsB-acsC-acsD-acsE-metV- GCA 003563385.1 d Bacteria p Firmicutes D c Dethiobacteria o DTU022
cooC-acsA-acsB-acsC-acsD-acsE-metV-metF- GCA 003563895.1 d Bacteria p Firmicutes D c Dethiobacteria o DTU022
acsB-acsC-acsD-acsE-metV-metF- GCA 003556225.1 d Bacteria p Firmicutes D c Dethiobacteria o DTU022
acsB-acsC-acsD-acsE-metV-metF- GCA 007126455.1 d Bacteria p Firmicutes D c Dethiobacteria o DTU022
acsB-acsC-acsD-acsE- 3X metV-metF- GCA 007121375.1 d Bacteria p Firmicutes D c Dethiobacteria o SKNC01
acsB-acsC-acsD-acsE- GCA 007120745.1 d Bacteria p Firmicutes D c Dethiobacteria o SKNC01
acsB-acsC-acsD-acsE-metV-metF- GCA 003561835.1 d Bacteria p Firmicutes D c Dethiobacteria o PWVN01
cooC-acsA-acsB-acsC-acsV-acsD-acsE- 4X metV-metF- GCA 012522335.1 d Bacteria p Firmicutes D c Dethiobacteria o Dethiobacterales
acsB-acsC-acsV-acsD-acsE- 4X metV-metF- GCA 012800795.1 d Bacteria p Firmicutes D c Dethiobacteria o Dethiobacterales
cooC-acsA-acsB-acsC-acsV-acsD-acsE- 4X metV-metF- GCA 002479415.1 d Bacteria p Firmicutes D c Dethiobacteria o Dethiobacterales
cooC-acsA-acsB-acsC-acsV-acsD-acsE- 4X metV-metF- GCF 000174415.1 d Bacteria p Firmicutes D c Dethiobacteria o Dethiobacterales
acsA-acsB-acsC-acsV-cooC2-acsD-acsE-metV-metF- GCA 002376005.1 d Bacteria p Firmicutes E c DTU015 o UBA3561
acsA-acsB- GCA 012842575.1 d Bacteria p Firmicutes E c DTU015 o DUSP01
acsA-acsB-acsC-acsV-cooC2-acsD-acsE- GCA 007130145.1 d Bacteria p Firmicutes E c DTU015 o UBA8153
acsB- GCA 003449495.1 d Bacteria p Firmicutes E c DTU015 o UBA8153
acsA-acsB-acsC-acsV-cooC2-acsD-acsE- 5X metV-metF- GCA 002375925.1 d Bacteria p Firmicutes E c UBA3569 o UBA3575
acsA-acsB-acsC- GCA 002375895.1 d Bacteria p Firmicutes E c UBA3569 o UBA3569
acsB-acsC-acsV-cooC2- GCA 003541445.1 d Bacteria p Firmicutes E c DTU015 o UBA9673
acsB- GCA 003530255.1 d Bacteria p Firmicutes E c DTU015 o UBA9673
cooC-acsA-acsB- GCA 012799075.1 d Bacteria p Firmicutes G c Limnochordia o JAAZLZ01
cooC-acsA-acsB-acsC-acsV-cooC2-acsD-acsE- GCA 012522085.1 d Bacteria p Firmicutes G c Limnochordia o JAAZLZ01
cooC-acsA-acsB-acsC-acsV-cooC2-acsD-acsE- GCA 012521755.1 d Bacteria p Firmicutes G c Limnochordia o JAAZLZ01
cooC-acsA-acsB-acsC-acsV-cooC2-acsD-acsE- 13X metV-metF- GCF 009735625.1 d Bacteria p Firmicutes B c Moorellia o Moorellales
cooC-acsA-acsB-acsC-acsV-cooC2-acsD-acsE- 13X metV-metF- GCF 001594015.1 d Bacteria p Firmicutes B c Moorellia o Moorellales
cooC-acsA-acsB-acsC-acsV-cooC2-acsD-acsE- 13X metV-metF- GCF 002995805.1 d Bacteria p Firmicutes B c Moorellia o Moorellales
cooC- 4X acsB-acsC-acsV-cooC2-acsD-acsE- 13X metV-metF- GCF 006538365.1 d Bacteria p Firmicutes B c Moorellia o Moorellales
cooC-acsA-acsB-acsC-acsV-cooC2-acsD-acsE- GCF 002995755.1 d Bacteria p Firmicutes B c Moorellia o Moorellales
cooC-acsA-acsB-acsC-acsV-cooC2-acsD- 1X acsE- 13X metV-metF- GCF 003116935.1 d Bacteria p Firmicutes B c Moorellia o Moorellales
cooC-acsA-acsB-acsC-acsV-cooC2-acsD-acsE- 13X metV-metF- GCA 002383405.1 d Bacteria p Firmicutes B c Moorellia o Moorellales
acsE 6X cooC-acsA-acsB-acsC-acsV-cooC2-acsD-acsE- 13X metV-metF- GCF 001267405.1 d Bacteria p Firmicutes B c Moorellia o Moorellales
cooC-acsA-acsB-acsC-acsV-cooC2-acsD-acsE- 13X metV-metF- GCF 001875325.1 d Bacteria p Firmicutes B c Moorellia o Moorellales
cooC-acsA-acsB-acsC-acsV-cooC2-acsD-acsE- 12X metV-metF- GCF 900176005.1 d Bacteria p Firmicutes B c Moorellia o Moorellales
cooC-acsA-acsB-acsC-acsV-cooC2-acsD-acsE- 12X metV-metF- GCF 013167955.1 d Bacteria p Firmicutes B c Moorellia o Moorellales
cooC-acsA-acsB-acsC-acsV-cooC2-acsD-acsE- 12X metV-metF- GCA 003509545.1 d Bacteria p Firmicutes B c Moorellia o Moorellales
cooC-acsA-acsB-acsC-acsD-acsE- 13X metV-metF- GCA 004347685.1 d Bacteria p Firmicutes B c Moorellia o Moorellales
cooC-acsA-acsB- GCA 014360105.1 d Bacteria p Firmicutes B c Moorellia o Moorellales
cooC- 4X acsB-acsC-acsV-cooC2-acsD-acsE- 13X metV-metF- GCF 002207765.1 d Bacteria p Firmicutes B c Moorellia o Calderihabitantales
acsA-acsB-acsC-acsV-cooC2-acsD-acsE- 13X metV-metF- GCA 000987045.1 d Bacteria p Firmicutes B c Moorellia o PH28-bin88
folD- 5X cooC-acsA-acsB-acsC-cooC2-acsD-acsE- 14X metV-metF- GCA 002352935.1 d Bacteria p Firmicutes B c Moorellia o UBA1874
cooC-acsA-acsB-acsC-acsV-cooC2-acsD-acsE- GCA 012840125.1 d Bacteria p Firmicutes B c Moorellia o DULZ01
cooC-acsA-acsB-acsC-acsV-cooC2-acsD-acsE- GCA 012512955.1 d Bacteria p Firmicutes B c Moorellia o DULZ01
cooC-acsA-acsB-acsC-acsV-cooC2-acsD-acsE- GCA 012520135.1 d Bacteria p Firmicutes B c Moorellia o DULZ01
cooC-acsA-acsB-acsC-acsV-cooC2-acsD-acsE- 1X metF- GCA 001515945.1 d Bacteria p Firmicutes B c Moorellia o Desulfitibacterales
cooC-acsA-acsB-acsC-acsV-cooC2-acsD-acsE-metF- GCF 000620305.1 d Bacteria p Firmicutes B c Moorellia o Desulfitibacterales
cooC-acsA-acsB-acsC-acsV-cooC2-acsD-acsE-metF- GCF 002777255.1 d Bacteria p Firmicutes B c Dehalobacteriia o Dehalobacteriales
cooC-acsA-acsB-acsC-acsV-cooC2-acsD-acsE- GCF 002224645.1 d Bacteria p Firmicutes B c Dehalobacteriia o Dehalobacteriales
cooC-acsA-acsB-acsC-acsV-cooC2-acsD-acsE-metF- GCA 012838175.1 d Bacteria p Firmicutes B c Dehalobacteriia o Dehalobacteriales
cooC-acsA-acsB-acsC-acsV-cooC2-acsD-acsE- GCA 002482535.1 d Bacteria p Firmicutes B c Dehalobacteriia o UBA7702
cooC-acsA-acsB-acsC-acsV-cooC2-acsD-acsE- GCA 902796855.1 d Bacteria p Firmicutes B c Dehalobacteriia o UBA7702
cooC-acsA-acsB-acsC-acsV-cooC2-acsD-acsE-metF- 11X fhs-fchA- GCA 001516055.1 d Bacteria p Firmicutes B c Desulfitobacteriia o Desulfitobacteriales
cooC-acsA-acsB-acsC-acsV-cooC2-acsD-acsE-metF- GCA 000961765.1 d Bacteria p Firmicutes B c Peptococcia o Peptococcales
cooC-acsA-acsB-acsC-acsV-cooC2-acsD-acsE-metF- GCA 000802045.1 d Bacteria p Firmicutes B c Peptococcia o Peptococcales
fhs-cooC-acsA-acsB- 2X acsV-acsD-acsE- GCA 002347475.1 d Bacteria p Firmicutes B c Desulfitobacteriia o Desulfitobacteriales
cooC-acsA-acsB-acsC-acsV-cooC2-acsD-acsE- GCF 000021925.1 d Bacteria p Firmicutes B c Desulfitobacteriia o Desulfitobacteriales
cooC-acsA-acsB-acsC-acsV-cooC2-acsD-acsE- GCF 900143285.1 d Bacteria p Firmicutes B c Desulfitobacteriia o Desulfitobacteriales
cooC-acsA-acsB-acsC-acsV-cooC2-acsD-acsE- GCF 000243155.2 d Bacteria p Firmicutes B c Desulfitobacteriia o Desulfitobacteriales
cooC-acsA-acsB-acsC-acsV-cooC2-acsD-acsE- GCF 000243135.2 d Bacteria p Firmicutes B c Desulfitobacteriia o Desulfitobacteriales
cooC-acsA-acsB-acsC-acsV-cooC2-acsD-acsE- GCF 000231405.2 d Bacteria p Firmicutes B c Desulfitobacteriia o Desulfitobacteriales
cooC-acsA-acsB-acsC-acsV-acsD-acsE- GCF 001029295.1 d Bacteria p Firmicutes B c Desulfitobacteriia o Desulfitobacteriales
cooC-acsA-acsB-acsC-acsV-cooC2-acsD-acsE- GCF 000224515.1 d Bacteria p Firmicutes B c Desulfitobacteriia o Desulfitobacteriales
cooC-acsA-acsB-acsC-acsV-cooC2-acsD-acsE- GCF 001707885.1 d Bacteria p Firmicutes B c Desulfitobacteriia o Desulfitobacteriales
acsA- 1X acsB-acsC-acsV-cooC2-acsD-acsE- GCA 002404215.1 d Bacteria p Firmicutes B c Desulfitobacteriia o Desulfitobacteriales
cooC-acsA-acsB-acsC-acsV-cooC2-acsD-acsE- GCF 900129935.1 d Bacteria p Firmicutes B c Desulfitobacteriia o Desulfitobacteriales
cooC-acsA-acsB-acsC-acsV-cooC2-acsD-acsE- GCA 000961805.1 d Bacteria p Firmicutes B c Desulfitobacteriia o Desulfitobacteriales
cooC-acsA-acsB-acsC-acsV-cooC2-acsD-acsE- GCF 004766045.1 d Bacteria p Firmicutes B c Desulfitobacteriia o Desulfitobacteriales
cooC-acsA-acsB-acsC-acsD- GCA 000770645.1 d Bacteria p Firmicutes B c Desulfitobacteriia o Desulfitobacteriales
acsA-acsB-acsC-acsV-cooC2-acsD-acsE-fhs- GCA 002413075.1 d Bacteria p Firmicutes B c Desulfitobacteriia o Desulfitobacteriales
cooC-acsA-acsB-acsC-acsV-cooC2- 2X acsE- GCA 002413075.1 d Bacteria p Firmicutes B c Desulfitobacteriia o Desulfitobacteriales
cooC-acsA-acsB-acsC-acsV-cooC2-acsD-acsE- GCF 004766055.1 d Bacteria p Firmicutes B c Desulfitobacteriia o Desulfitobacteriales
cooC-acsA-acsB-acsC-acsV-cooC2-acsD-acsE- GCF 001936615.1 d Bacteria p Firmicutes B c Desulfitobacteriia o Desulfitobacteriales

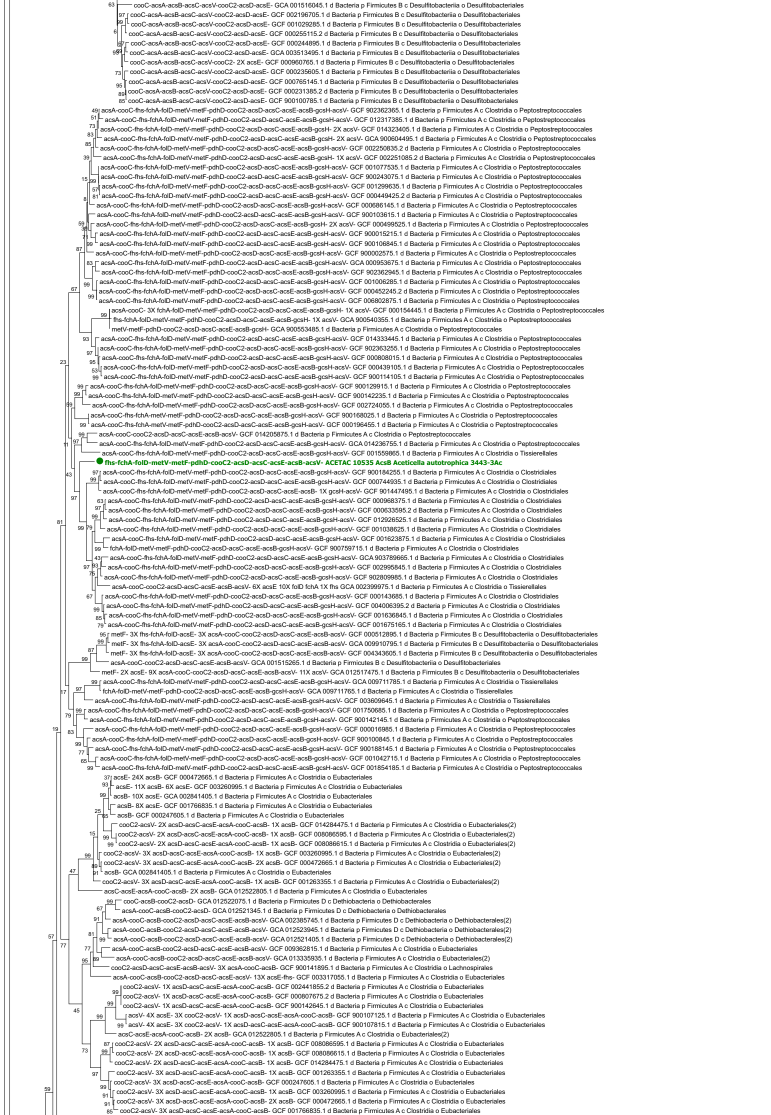

cooC-acsA-acsB-acsC-acsV-cooC2-acsD-acsE- GCA 001516045.1 d Bacteria p Firmicutes B c Desulfitobacteriia o Desulfitobacteriales
cooC-acsA-acsB-acsC-acsV-cooC2-acsD-acsE- GCF 002196705.1 d Bacteria p Firmicutes B c Desulfitobacteriia o Desulfitobacteriales
cooC-acsA-acsB-acsC-acsV-cooC2-acsD-acsE- GCF 001029285.1 d Bacteria p Firmicutes B c Desulfitobacteriia o Desulfitobacteriales
cooC-acsA-acsB-acsC-acsV-cooC2-acsD-acsE- GCF 000255115.2 d Bacteria p Firmicutes B c Desulfitobacteriia o Desulfitobacteriales
cooC-acsA-acsB-acsC-acsV-cooC2-acsD-acsE- GCF 000244895.1 d Bacteria p Firmicutes B c Desulfitobacteriia o Desulfitobacteriales
cooC-acsA-acsB-acsC-acsV-cooC2-acsD-acsE- GCA 003513495.1 d Bacteria p Firmicutes B c Desulfitobacteriia o Desulfitobacteriales
cooC-acsA-acsB-acsC-acsV-cooC2- 2X acsE- GCF 000960765.1 d Bacteria p Firmicutes B c Desulfitobacteriia o Desulfitobacteriales
cooC-acsA-acsB-acsC-acsV-cooC2-acsD-acsE- GCF 000235605.1 d Bacteria p Firmicutes B c Desulfitobacteriia o Desulfitobacteriales
cooC-acsA-acsB-acsC-acsV-cooC2-acsD-acsE- GCF 000765145.1 d Bacteria p Firmicutes B c Desulfitobacteriia o Desulfitobacteriales
cooC-acsA-acsB-acsC-acsV-cooC2-acsD-acsE- GCF 000231385.2 d Bacteria p Firmicutes B c Desulfitobacteriia o Desulfitobacteriales
cooC-acsA-acsB-acsC-acsV-cooC2-acsD-acsE- GCF 900100785.1 d Bacteria p Firmicutes B c Desulfitobacteriia o Desulfitobacteriales
acsA-cooC-fhs-fchA-folD-metV-metF-pdhD-cooC2-acsD-acsC-acsE-acsB-gcsH-acsV- GCF 902362365.1 d Bacteria p Firmicutes A c Clostridia o Peptostreptococcales
acsA-cooC-fhs-fchA-folD-metV-metF-pdhD-cooC2-acsD-acsC-acsE-acsB-gcsH-acsV- GCF 012317385.1 d Bacteria p Firmicutes A c Clostridia o Peptostreptococcales
acsA-cooC-fhs-fchA-folD-metV-metF-pdhD-cooC2-acsD-acsC-acsE-acsB-gcsH- 2X acsV- GCF 014323405.1 d Bacteria p Firmicutes A c Clostridia o Peptostreptococcales
acsA-cooC-fhs-fchA-folD-metV-metF-pdhD-cooC2-acsD-acsC-acsE-acsB-gcsH- 2X acsV- GCA 900604495.1 d Bacteria p Firmicutes A c Clostridia o Peptostreptococcales
acsA-cooC-fhs-fchA-folD-metV-metF-pdhD-cooC2-acsD-acsC-acsE-acsB-gcsH-acsV- GCF 002250835.2 d Bacteria p Firmicutes A c Clostridia o Peptostreptococcales
acsA-cooC-fhs-fchA-folD-metV-metF-pdhD-cooC2-acsD-acsC-acsE-acsB-gcsH- 1X acsV- GCF 002251085.2 d Bacteria p Firmicutes A c Clostridia o Peptostreptococcales
acsA-cooC-fhs-fchA-folD-metV-metF-pdhD-cooC2-acsD-acsC-acsE-acsB-gcsH-acsV- GCF 001077535.1 d Bacteria p Firmicutes A c Clostridia o Peptostreptococcales
acsA-cooC-fhs-fchA-folD-metV-metF-pdhD-cooC2-acsD-acsC-acsE-acsB-gcsH-acsV- GCF 900243075.1 d Bacteria p Firmicutes A c Clostridia o Peptostreptococcales
acsA-cooC-fhs-fchA-folD-metV-metF-pdhD-cooC2-acsD-acsC-acsE-acsB-gcsH-acsV- GCF 001299635.1 d Bacteria p Firmicutes A c Clostridia o Peptostreptococcales
acsA-cooC-fhs-fchA-folD-metV-metF-pdhD-cooC2-acsD-acsC-acsE-acsB-gcsH-acsV- GCF 000449425.2 d Bacteria p Firmicutes A c Clostridia o Peptostreptococcales
acsA-cooC-fhs-fchA-folD-metV-metF-pdhD-cooC2-acsD-acsC-acsE-acsB-gcsH-acsV- GCF 000686145.1 d Bacteria p Firmicutes A c Clostridia o Peptostreptococcales
acsA-cooC-fhs-fchA-folD-metV-metF-pdhD-cooC2-acsD-acsC-acsE-acsB-gcsH-acsV- GCF 900103615.1 d Bacteria p Firmicutes A c Clostridia o Peptostreptococcales
acsA-cooC-fhs-fchA-folD-metV-metF-pdhD-cooC2-acsD-acsC-acsE-acsB-gcsH- 2X acsV- GCF 000499525.1 d Bacteria p Firmicutes A c Clostridia o Peptostreptococcales
acsA-cooC-fhs-fchA-folD-metV-metF-pdhD-cooC2-acsD-acsC-acsE-acsB-gcsH-acsV- GCF 900015215.1 d Bacteria p Firmicutes A c Clostridia o Peptostreptococcales
acsA-cooC-fhs-fchA-folD-metV-metF-pdhD-cooC2-acsD-acsC-acsE-acsB-gcsH-acsV- GCF 900106845.1 d Bacteria p Firmicutes A c Clostridia o Peptostreptococcales
acsA-cooC-fhs-fchA-folD-metV-metF-pdhD-cooC2-acsD-acsC-acsE-acsB-gcsH-acsV- GCF 900002575.1 d Bacteria p Firmicutes A c Clostridia o Peptostreptococcales
acsA-cooC-fhs-fchA-folD-metV-metF-pdhD-cooC2-acsD-acsC-acsE-acsB-gcsH-acsV- GCA 000953675.1 d Bacteria p Firmicutes A c Clostridia o Peptostreptococcales
acsA-cooC-fhs-fchA-folD-metV-metF-pdhD-cooC2-acsD-acsC-acsE-acsB-gcsH-acsV- GCF 902362945.1 d Bacteria p Firmicutes A c Clostridia o Peptostreptococcales
acsA-cooC-fhs-fchA-folD-metV-metF-pdhD-cooC2-acsD-acsC-acsE-acsB-gcsH-acsV- GCF 001006285.1 d Bacteria p Firmicutes A c Clostridia o Peptostreptococcales
acsA-cooC-fhs-fchA-folD-metV-metF-pdhD-cooC2-acsD-acsC-acsE-acsB-gcsH-acsV- GCF 000452245.2 d Bacteria p Firmicutes A c Clostridia o Peptostreptococcales
acsA-cooC-fhs-fchA-folD-metV-metF-pdhD-cooC2-acsD-acsC-acsE-acsB-gcsH-acsV- GCF 006802875.1 d Bacteria p Firmicutes A c Clostridia o Peptostreptococcales
acsA-cooC- 3X fchA-folD-metV-metF-pdhD-cooC2-acsD-acsC-acsE-acsB-gcsH- 1X acsV- GCF 000154445.1 d Bacteria p Firmicutes A c Clostridia o Peptostreptococcales
fhs-fchA-folD-metV-metF-pdhD-cooC2-acsD-acsC-acsE-acsB-gcsH- 1X acsV- GCA 900540355.1 d Bacteria p Firmicutes A c Clostridia o Peptostreptococcales
metV-metF-pdhD-cooC2-acsD-acsC-acsE-acsB-gcsH- GCA 900553485.1 d Bacteria p Firmicutes A c Clostridia o Peptostreptococcales
acsA-cooC-fhs-fchA-folD-metV-metF-pdhD-cooC2-acsD-acsC-acsE-acsB-gcsH-acsV- GCF 014333445.1 d Bacteria p Firmicutes A c Clostridia o Peptostreptococcales
acsA-cooC-fhs-fchA-folD-metV-metF-pdhD-cooC2-acsD-acsC-acsE-acsB-gcsH-acsV- GCF 902363255.1 d Bacteria p Firmicutes A c Clostridia o Peptostreptococcales
acsA-cooC-fhs-fchA-folD-metV-metF-pdhD-cooC2-acsD-acsC-acsE-acsB-gcsH-acsV- GCF 000808015.1 d Bacteria p Firmicutes A c Clostridia o Peptostreptococcales
acsA-cooC-fhs-fchA-folD-metV-metF-pdhD-cooC2-acsD-acsC-acsE-acsB-gcsH-acsV- GCF 000439105.1 d Bacteria p Firmicutes A c Clostridia o Peptostreptococcales
acsA-cooC-fhs-fchA-folD-metV-metF-pdhD-cooC2-acsD-acsC-acsE-acsB-gcsH-acsV- GCF 900114105.1 d Bacteria p Firmicutes A c Clostridia o Peptostreptococcales
acsA-cooC-fhs-fchA-folD-metV-metF-pdhD-cooC2-acsD-acsC-acsE-acsB-gcsH-acsV- GCF 900129915.1 d Bacteria p Firmicutes A c Clostridia o Peptostreptococcales
acsA-cooC-fhs-fchA-folD-metV-metF-pdhD-cooC2-acsD-acsC-acsE-acsB-gcsH-acsV- GCF 900142235.1 d Bacteria p Firmicutes A c Clostridia o Peptostreptococcales
acsA-cooC-fhs-fchA-folD-metV-metF-pdhD-cooC2-acsD-acsC-acsE-acsB-gcsH-acsV- GCF 002724055.1 d Bacteria p Firmicutes A c Clostridia o Peptostreptococcales
acsA-cooC-fhs-fchA-metV-metF-pdhD-cooC2-acsD-acsC-acsE-acsB-gcsH-acsV- GCF 900168025.1 d Bacteria p Firmicutes A c Clostridia o Peptostreptococcales
acsA-cooC-fhs-fchA-metV-metF-pdhD-cooC2-acsD-acsC-acsE-acsB-gcsH-acsV- GCF 900196455.1 d Bacteria p Firmicutes A c Clostridia o Peptostreptococcales
acsA-cooC-cooC2-acsD-acsC-acsE-acsB-acsV- GCF 014205875.1 d Bacteria p Firmicutes A c Clostridia o Peptostreptococcales
acsA-cooC-fhs-fchA-folD-metV-metF-pdhD-cooC2-acsD-acsC-acsE-acsB-gcsH-acsV- GCA 014236755.1 d Bacteria p Firmicutes A c Clostridia o Peptostreptococcales
acsA-cooC-fhs-fchA-folD-metV-metF-pdhD-cooC2-acsD-acsC-acsE-acsB-gcsH-acsV- GCF 001559865.1 d Bacteria p Firmicutes A c Clostridia o Tissierellales
fhs-fchA-folD-metV-metF-pdhD-cooC2-acsD-acsC-acsE-acsB-acsV- ACETAC 10535 AcsB Aceticella autotrophica 3443-3Ac
acsA-cooC-fhs-fchA-folD-metV-metF-pdhD-cooC2-acsD-acsC-acsE-acsB-gcsH-acsV- GCF 900184255.1 d Bacteria p Firmicutes A c Clostridia o Clostridiales
acsA-cooC-fhs-fchA-folD-metV-metF-pdhD-cooC2-acsD-acsC-acsE-acsB-gcsH-acsV- GCF 000744935.1 d Bacteria p Firmicutes A c Clostridia o Clostridiales
acsA-cooC-fhs-fchA-folD-metV-metF-pdhD-cooC2-acsD-acsC-acsE-acsB- 1X gcsH-acsV- GCF 901447495.1 d Bacteria p Firmicutes A c Clostridia o Clostridiales
acsA-cooC-fhs-fchA-folD-metV-metF-pdhD-cooC2-acsD-acsC-acsE-acsB-gcsH-acsV- GCF 000968375.1 d Bacteria p Firmicutes A c Clostridia o Clostridiales
acsA-cooC-fhs-fchA-folD-metV-metF-pdhD-cooC2-acsD-acsC-acsE-acsB-gcsH-acsV- GCF 000633595.2 d Bacteria p Firmicutes A c Clostridia o Clostridiales
acsA-cooC-fhs-fchA-folD-metV-metF-pdhD-cooC2-acsD-acsC-acsE-acsB-gcsH-acsV- GCF 012926525.1 d Bacteria p Firmicutes A c Clostridia o Clostridiales
acsA-cooC-fhs-fchA-folD-metV-metF-pdhD-cooC2-acsD-acsC-acsE-acsB-gcsH-acsV- GCF 001038625.1 d Bacteria p Firmicutes A c Clostridia o Clostridiales
acsA-cooC-fhs-fchA-folD-metV-metF-pdhD-cooC2-acsD-acsC-acsE-acsB-gcsH-acsV- GCF 001623875.1 d Bacteria p Firmicutes A c Clostridia o Clostridiales
fchA-folD-metV-metF-pdhD-cooC2-acsD-acsC-acsE-acsB-gcsH-acsV- GCF 900759715.1 d Bacteria p Firmicutes A c Clostridia o Clostridiales
acsA-cooC-fhs-fchA-folD-metV-metF-pdhD-cooC2-acsD-acsC-acsE-acsB-gcsH-acsV- GCA 903789665.1 d Bacteria p Firmicutes A c Clostridia o Clostridiales
acsA-cooC-fhs-fchA-folD-metV-metF-pdhD-cooC2-acsD-acsC-acsE-acsB-gcsH-acsV- GCF 002995845.1 d Bacteria p Firmicutes A c Clostridia o Clostridiales
acsA-cooC-fhs-fchA-folD-metV-metF-pdhD-cooC2-acsD-acsC-acsE-acsB-gcsH-acsV- GCF 902809985.1 d Bacteria p Firmicutes A c Clostridia o Clostridiales
acsA-cooC-cooC2-acsD-acsC-acsE-acsB-acsV- 6X acsE 10X folD fchA 1X fhs GCA 002399975.1 d Bacteria p Firmicutes A c Clostridia o Tissierellales
acsA-cooC-fhs-fchA-folD-metV-metF-pdhD-cooC2-acsD-acsC-acsE-acsB-gcsH-acsV- GCF 000143685.1 d Bacteria p Firmicutes A c Clostridia o Clostridiales
acsA-cooC-fhs-fchA-folD-metV-metF-pdhD-cooC2-acsD-acsC-acsE-acsB-gcsH-acsV- GCF 004006395.2 d Bacteria p Firmicutes A c Clostridia o Clostridiales
acsA-cooC-fhs-fchA-folD-metV-metF-pdhD-cooC2-acsD-acsC-acsE-acsB-gcsH-acsV- GCF 001636845.1 d Bacteria p Firmicutes A c Clostridia o Clostridiales
acsA-cooC-fhs-fchA-folD-metV-metF-pdhD-cooC2-acsD-acsC-acsE-acsB-gcsH-acsV- GCF 001675165.1 d Bacteria p Firmicutes A c Clostridia o Clostridiales
metF- 3X fhs-fchA-folD-acsE- 3X acsA-cooC-cooC2-acsD-acsC-acsE-acsB-acsV- GCF 000512895.1 d Bacteria p Firmicutes B c Desulfitobacteriia o Desulfitobacteriales
metF- 3X fhs-fchA-folD-acsE- 3X acsA-cooC-cooC2-acsD-acsC-acsE-acsB-acsV- GCA 009910795.1 d Bacteria p Firmicutes B c Desulfitobacteriia o Desulfitobacteriales
metF- 3X fhs-fchA-folD-acsE- 3X acsA-cooC-cooC2-acsD-acsC-acsE-acsB-acsV- GCF 004343605.1 d Bacteria p Firmicutes B c Desulfitobacteriia o Desulfitobacteriales
acsA-cooC-cooC2-acsD-acsC-acsE-acsB-acsV- GCA 001515265.1 d Bacteria p Firmicutes B c Desulfitobacteriia o Desulfitobacteriales
metF- 2X acsE- 9X acsA-cooC-cooC2-acsD-acsC-acsE-acsB-acsV- 11X acsV- GCA 012517475.1 d Bacteria p Firmicutes B c Desulfitobacteriia o Desulfitobacteriales
acsA-cooC-fhs-fchA-folD-metV-metF-pdhD-cooC2-acsD-acsC-acsE-acsB-gcsH-acsV- GCA 009711785.1 d Bacteria p Firmicutes A c Clostridia o Tissierellales
fchA-folD-metV-metF-pdhD-cooC2-acsD-acsC-acsE-acsB-gcsH-acsV- GCA 009711765.1 d Bacteria p Firmicutes A c Clostridia o Tissierellales
acsA-cooC-fhs-fchA-folD-metV-metF-pdhD-cooC2-acsD-acsC-acsE-acsB-gcsH-acsV- GCF 003609645.1 d Bacteria p Firmicutes A c Clostridia o Tissierellales
acsA-cooC-fhs-fchA-folD-metV-metF-pdhD-cooC2-acsD-acsC-acsE-acsB-gcsH-acsV- GCF 001750685.1 d Bacteria p Firmicutes A c Clostridia o Peptostreptococcales
acsA-cooC-fhs-fchA-folD-metV-metF-pdhD-cooC2-acsD-acsC-acsE-acsB-gcsH-acsV- GCF 900142145.1 d Bacteria p Firmicutes A c Clostridia o Peptostreptococcales
acsA-cooC-fhs-fchA-folD-metV-metF-pdhD-cooC2-acsD-acsC-acsE-acsB-gcsH-acsV- GCF 000016985.1 d Bacteria p Firmicutes A c Clostridia o Peptostreptococcales
acsA-cooC-fhs-fchA-folD-metV-metF-pdhD-cooC2-acsD-acsC-acsE-acsB-gcsH-acsV- GCF 900100845.1 d Bacteria p Firmicutes A c Clostridia o Peptostreptococcales
acsA-cooC-fhs-fchA-folD-metV-metF-pdhD-cooC2-acsD-acsC-acsE-acsB-gcsH-acsV- GCF 900188145.1 d Bacteria p Firmicutes A c Clostridia o Peptostreptococcales
acsA-cooC-fhs-fchA-folD-metV-metF-pdhD-cooC2-acsD-acsC-acsE-acsB-gcsH-acsV- GCF 001042715.1 d Bacteria p Firmicutes A c Clostridia o Peptostreptococcales
acsA-cooC-fhs-fchA-folD-metV-metF-pdhD-cooC2-acsD-acsC-acsE-acsB-gcsH-acsV- GCF 001854185.1 d Bacteria p Firmicutes A c Clostridia o Peptostreptococcales
acsE- 24X acsB- GCF 000472665.1 d Bacteria p Firmicutes A c Clostridia o Eubacteriales
acsE- 11X acsB- 6X acsE- GCF 003260995.1 d Bacteria p Firmicutes A c Clostridia o Eubacteriales
acsB- 10X acsE- GCA 002841405.1 d Bacteria p Firmicutes A c Clostridia o Eubacteriales
acsB- 8X acsE- GCF 001766835.1 d Bacteria p Firmicutes A c Clostridia o Eubacteriales
acsB- GCF 000247605.1 d Bacteria p Firmicutes A c Clostridia o Eubacteriales
cooC2-acsV- 2X acsD-acsC-acsE-acsA-cooC-acsB- 1X acsB- GCF 014284475.1 d Bacteria p Firmicutes A c Clostridia o Eubacteriales(2)
cooC2-acsV- 2X acsD-acsC-acsE-acsA-cooC-acsB- 1X acsB- GCF 008086595.1 d Bacteria p Firmicutes A c Clostridia o Eubacteriales(2)
cooC2-acsV- 2X acsD-acsC-acsE-acsA-cooC-acsB- 1X acsB- GCF 008086615.1 d Bacteria p Firmicutes A c Clostridia o Eubacteriales(2)
cooC2-acsV- 3X acsD-acsC-acsE-acsA-cooC-acsB- 1X acsB- GCF 003260995.1 d Bacteria p Firmicutes A c Clostridia o Eubacteriales(2)
cooC2-acsV- 3X acsD-acsC-acsE-acsA-cooC-acsB- 2X acsB- GCF 000472665.1 d Bacteria p Firmicutes A c Clostridia o Eubacteriales(2)
acsB- GCA 002841405.1 d Bacteria p Firmicutes A c Clostridia o Eubacteriales
cooC2-acsV- 3X acsD-acsC-acsE-acsA-cooC-acsB- 1X acsB- GCF 001263355.1 d Bacteria p Firmicutes A c Clostridia o Eubacteriales(2)
acsC-acsE-acsA-cooC-acsB- 2X acsB- GCA 012522805.1 d Bacteria p Firmicutes A c Clostridia o Eubacteriales
cooC-acsB-cooC2-acsD- GCA 012522075.1 d Bacteria p Firmicutes D c Dethiobacteria o Dethiobacterales
acsA-cooC-acsB-cooC2-acsD- GCA 012521345.1 d Bacteria p Firmicutes D c Dethiobacteria o Dethiobacterales
acsA-cooC-acsB-cooC2-acsD-acsC-acsE-acsB-acsV- GCA 002385745.1 d Bacteria p Firmicutes D c Dethiobacteria o Dethiobacterales(2)
acsA-cooC-acsB-cooC2-acsD-acsC-acsE-acsB-acsV- GCA 012523945.1 d Bacteria p Firmicutes D c Dethiobacteria o Dethiobacterales(2)
acsA-cooC-acsB-cooC2-acsD-acsC-acsE-acsB-acsV- GCA 012521405.1 d Bacteria p Firmicutes D c Dethiobacteria o Dethiobacterales(2)
acsA-cooC-acsB-cooC2-acsD-acsC-acsE-acsB-acsV- GCF 009362815.1 d Bacteria p Firmicutes A c Clostridia o Eubacteriales
acsA-cooC-acsB-cooC2-acsD-acsC-acsE-acsB-acsV- GCA 013335935.1 d Bacteria p Firmicutes A c Clostridia o Eubacteriales(2)
cooC2-acsD-acsC-acsE-acsB-acsV- 3X acsA-cooC-acsB- GCF 900141895.1 d Bacteria p Firmicutes A c Clostridia o Lachnospirales
acsA-cooC-acsB-cooC2-acsD-acsC-acsE-acsV- 13X acsE-fhs- GCF 003317055.1 d Bacteria p Firmicutes A c Clostridia o Eubacteriales
cooC2-acsV- 1X acsD-acsC-acsE-acsA-cooC-acsB- GCF 002441855.2 d Bacteria p Firmicutes A c Clostridia o Eubacteriales
cooC2-acsV- 1X acsD-acsC-acsE-acsA-cooC-acsB- GCF 000807675.2 d Bacteria p Firmicutes A c Clostridia o Eubacteriales
cooC2-acsV- 1X acsD-acsC-acsE-acsA-cooC-acsB- GCF 900142645.1 d Bacteria p Firmicutes A c Clostridia o Eubacteriales
acsV- 4X acsE- 3X cooC2-acsV- 1X acsD-acsC-acsE-acsA-cooC-acsB- GCF 900107125.1 d Bacteria p Firmicutes A c Clostridia o Eubacteriales
acsV- 4X acsE- 3X cooC2-acsV- 1X acsD-acsC-acsE-acsA-cooC-acsB- GCF 900107815.1 d Bacteria p Firmicutes A c Clostridia o Eubacteriales
acsC-acsE-acsA-cooC-acsB- 2X acsB- GCA 012522805.1 d Bacteria p Firmicutes A c Clostridia o Eubacteriales(2)
cooC2-acsV- 2X acsD-acsC-acsE-acsA-cooC-acsB- 1X acsB- GCF 008086595.1 d Bacteria p Firmicutes A c Clostridia o Eubacteriales
cooC2-acsV- 2X acsD-acsC-acsE-acsA-cooC-acsB- 1X acsB- GCF 008086615.1 d Bacteria p Firmicutes A c Clostridia o Eubacteriales
cooC2-acsV- 2X acsD-acsC-acsE-acsA-cooC-acsB- 1X acsB- GCF 014284475.1 d Bacteria p Firmicutes A c Clostridia o Eubacteriales
cooC2-acsV- 3X acsD-acsC-acsE-acsA-cooC-acsB- 1X acsB- GCF 001263355.1 d Bacteria p Firmicutes A c Clostridia o Eubacteriales
cooC2-acsV- 3X acsD-acsC-acsE-acsA-cooC-acsB- GCF 000247605.1 d Bacteria p Firmicutes A c Clostridia o Eubacteriales
cooC2-acsV- 3X acsD-acsC-acsE-acsA-cooC-acsB- 1X acsB- GCF 003260995.1 d Bacteria p Firmicutes A c Clostridia o Eubacteriales
cooC2-acsV- 3X acsD-acsC-acsE-acsA-cooC-acsB- 2X acsB- GCF 000472665.1 d Bacteria p Firmicutes A c Clostridia o Eubacteriales
cooC2-acsV- 3X acsD-acsC-acsE-acsA-cooC-acsB- GCF 001766835.1 d Bacteria p Firmicutes A c Clostridia o Eubacteriales

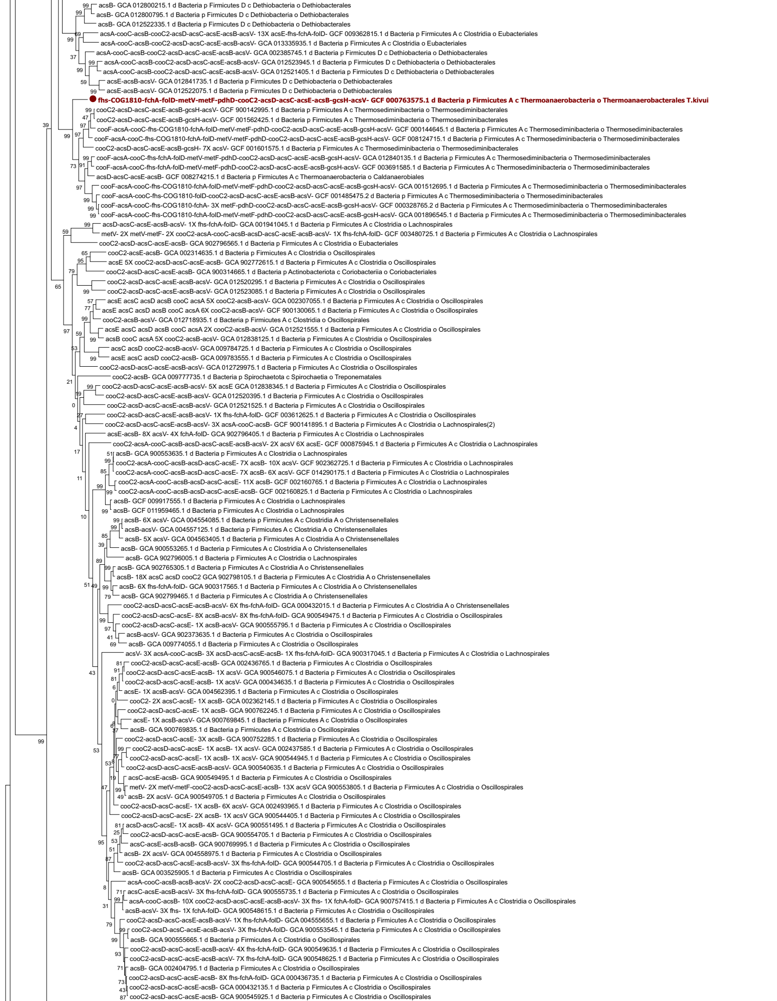

acsB- GCA 012800215.1 d Bacteria p Firmicutes D c Dethiobacteria o Dethiobacterales
acsB- GCA 012800795.1 d Bacteria p Firmicutes D c Dethiobacteria o Dethiobacterales
acsB- GCA 012522335.1 d Bacteria p Firmicutes D c Dethiobacteria o Dethiobacterales
acsA-cooC-acsB-cooC2-acsD-acsC-acsE-acsB-acsV- 13X acsE-fhs-fchA-folD- GCF 009362815.1 d Bacteria p Firmicutes A c Clostridia o Eubacteriales
acsA-cooC-acsB-cooC2-acsD-acsC-acsE-acsB-acsV- GCA 013335935.1 d Bacteria p Firmicutes A c Clostridia o Eubacteriales
acsA-cooC-acsB-cooC2-acsD-acsC-acsE-acsB-acsV- GCA 002385745.1 d Bacteria p Firmicutes D c Dethiobacteria o Dethiobacterales
acsA-cooC-acsB-cooC2-acsD-acsC-acsE-acsB-acsV- GCA 012523945.1 d Bacteria p Firmicutes D c Dethiobacteria o Dethiobacterales
acsA-cooC-acsB-cooC2-acsD-acsC-acsE-acsB-acsV- GCA 012521405.1 d Bacteria p Firmicutes D c Dethiobacteria o Dethiobacterales
acsE-acsB-acsV- GCA 012841735.1 d Bacteria p Firmicutes D c Dethiobacteria o Dethiobacterales
acsE-acsB-acsV- GCA 012522075.1 d Bacteria p Firmicutes D c Dethiobacteria o Dethiobacterales
fhs-COG1810-fchA-folD-metV-metF-pdhD-cooC2-acsD-acsC-acsE-acsB-gcsH-acsV- GCF 000763575.1 d Bacteria p Firmicutes A c Thermoanaerobacteria o Thermoanaerobacterales T.kivui
cooC2-acsD-acsC-acsE-acsB-gcsH-acsV- GCF 900142995.1 d Bacteria p Firmicutes A c Thermosediminibacteria o Thermosediminibacterales
cooC2-acsD-acsC-acsE-acsB-gcsH-acsV- GCF 001562425.1 d Bacteria p Firmicutes A c Thermosediminibacteria o Thermosediminibacterales
cooF-acsA-cooC-fhs-fchA-folD-metV-metF-pdhD-cooC2-acsD-acsC-acsE-acsB-gcsH-acsV- GCF 000144645.1 d Bacteria p Firmicutes A c Thermosediminibacteria o Thermosediminibacterales
cooF-acsA-cooC-fhs-COG1810-fchA-folD-metV-metF-pdhD-cooC2-acsD-acsC-acsE-acsB-gcsH-acsV- GCF 008124715.1 d Bacteria p Firmicutes A c Thermosediminibacteria o Thermosediminibacterales
cooC2-acsD-acsC-acsE-acsB-gcsH- 7X acsV- GCF 001601575.1 d Bacteria p Firmicutes A c Thermosediminibacteria o Thermosediminibacterales
cooF-acsA-cooC-fhs-fchA-folD-metV-metF-pdhD-cooC2-acsD-acsC-acsE-acsB-gcsH-acsV- GCA 012840135.1 d Bacteria p Firmicutes A c Thermosediminibacteria o Thermosediminibacterales
cooF-acsA-cooC-fhs-fchA-folD-metV-metF-pdhD-cooC2-acsD-acsC-acsE-acsB-gcsH-acsV- GCF 003691585.1 d Bacteria p Firmicutes A c Thermosediminibacteria o Thermosediminibacterales
acsD-acsC-acsE-acsB- GCF 008274215.1 d Bacteria p Firmicutes A c Thermoanaerobacteria o Caldanaerobiales
cooF-acsA-cooC-fhs-COG1810-fchA-folD-metV-metF-pdhD-cooC2-acsD-acsC-acsE-acsB-gcsH-acsV- GCA 001512695.1 d Bacteria p Firmicutes A c Thermosediminibacteria o Thermosediminibacterales
cooF-acsA-cooC-fhs-COG1810-folD-cooC2-acsD-acsC-acsE-acsB-acsV- GCF 001485475.2 d Bacteria p Firmicutes A c Thermosediminibacteria o Thermosediminibacterales
cooF-acsA-cooC-fhs-COG1810-fchA- 3X metF-pdhD-cooC2-acsD-acsC-acsE-acsB-gcsH-acsV- GCF 000328765.2 d Bacteria p Firmicutes A c Thermosediminibacteria o Thermosediminibacterales
cooF-acsA-cooC-fhs-COG1810-fchA-folD-metV-metF-pdhD-cooC2-acsD-acsC-acsE-acsB-gcsH-acsV- GCA 001896545.1 d Bacteria p Firmicutes A c Thermosediminibacteria o Thermosediminibacterales
acsD-acsC-acsE-acsB-acsV- 1X fhs-fchA-folD- GCA 001941045.1 d Bacteria p Firmicutes A c Clostridia o Lachnospirales
metV- 2X metV-metF- 2X cooC2-acsA-cooC-acsB-acsD-acsC-acsE-acsB-acsV- 1X fhs-fchA-folD- GCF 003480725.1 d Bacteria p Firmicutes A c Clostridia o Lachnospirales
cooC2-acsD-acsC-acsE-acsB- GCA 902796565.1 d Bacteria p Firmicutes A c Clostridia o Eubacteriales
cooC2-acsE-acsB- GCA 002314635.1 d Bacteria p Firmicutes A c Clostridia o Oscillospirales
acsE 5X cooC2-acsD-acsC-acsE-acsB- GCA 902772615.1 d Bacteria p Firmicutes A c Clostridia o Oscillospirales
cooC2-acsD-acsC-acsE-acsB- GCA 900314665.1 d Bacteria p Actinobacteriota c Coriobacteriia o Coriobacteriales
cooC2-acsD-acsC-acsE-acsB-acsV- GCA 012520295.1 d Bacteria p Firmicutes A c Clostridia o Oscillospirales
cooC2-acsD-acsC-acsE-acsB-acsV- GCA 012523085.1 d Bacteria p Firmicutes A c Clostridia o Oscillospirales
acsE acsC acsD acsB cooC acsA 5X cooC2-acsB-acsV- GCA 002307055.1 d Bacteria p Firmicutes A c Clostridia o Oscillospirales
acsE acsC acsD acsB cooC acsA 6X cooC2-acsB-acsV- GCF 900130065.1 d Bacteria p Firmicutes A c Clostridia o Oscillospirales
cooC2-acsB-acsV- GCA 012718935.1 d Bacteria p Firmicutes A c Clostridia o Oscillospirales
acsE acsC acsD acsB cooC acsA 2X cooC2-acsB-acsV- GCA 012521555.1 d Bacteria p Firmicutes A c Clostridia o Oscillospirales
acsB cooC acsA 5X cooC2-acsB-acsV- GCA 012838125.1 d Bacteria p Firmicutes A c Clostridia o Oscillospirales
acsC acsD cooC2-acsB-acsV- GCA 009784725.1 d Bacteria p Firmicutes A c Clostridia o Oscillospirales
acsE acsC acsD cooC2-acsB- GCA 009783555.1 d Bacteria p Firmicutes A c Clostridia o Oscillospirales
cooC2-acsD-acsC-acsE-acsB-acsV- GCA 012729975.1 d Bacteria p Firmicutes A c Clostridia o Oscillospirales
cooC2-acsB- GCA 009777735.1 d Bacteria p Spirochaetota c Spirochaetia o Treponematales
cooC2-acsD-acsC-acsE-acsB-acsV- 5X acsE GCA 012838345.1 d Bacteria p Firmicutes A c Clostridia o Oscillospirales
cooC2-acsD-acsC-acsE-acsB-acsV- GCA 012520395.1 d Bacteria p Firmicutes A c Clostridia o Oscillospirales
cooC2-acsD-acsC-acsE-acsB-acsV- GCA 012521525.1 d Bacteria p Firmicutes A c Clostridia o Oscillospirales
cooC2-acsD-acsC-acsE-acsB-acsV- 1X fhs-fchA-folD- GCF 003612625.1 d Bacteria p Firmicutes A c Clostridia o Oscillospirales
cooC2-acsD-acsC-acsE-acsB-acsV- 3X acsA-cooC-acsB- GCF 900141895.1 d Bacteria p Firmicutes A c Clostridia o Lachnospirales(2)
acsE-acsB- 8X acsV- 4X fchA-folD- GCA 902796405.1 d Bacteria p Firmicutes A c Clostridia o Lachnospirales
cooC2-acsA-cooC-acsB-acsD-acsC-acsE-acsB-acsV- 2X acsV 6X acsE- GCF 000875945.1 d Bacteria p Firmicutes A c Clostridia o Lachnospirales
acsB- GCA 900553635.1 d Bacteria p Firmicutes A c Clostridia o Lachnospirales
cooC2-acsA-cooC-acsB-acsD-acsC-acsE- 7X acsB- 10X acsV- GCF 902362725.1 d Bacteria p Firmicutes A c Clostridia o Lachnospirales
cooC2-acsA-cooC-acsB-acsD-acsC-acsE- 7X acsB- 6X acsV- GCF 014290175.1 d Bacteria p Firmicutes A c Clostridia o Lachnospirales
cooC2-acsA-cooC-acsB-acsD-acsC-acsE- 11X acsB- GCF 002160765.1 d Bacteria p Firmicutes A c Clostridia o Lachnospirales
cooC2-acsA-cooC-acsB-acsD-acsC-acsE-acsB- GCF 002160825.1 d Bacteria p Firmicutes A c Clostridia o Lachnospirales
acsB- GCF 009917555.1 d Bacteria p Firmicutes A c Clostridia o Lachnospirales
acsB- GCF 011959465.1 d Bacteria p Firmicutes A c Clostridia o Lachnospirales
acsB- 6X acsV- GCA 004554085.1 d Bacteria p Firmicutes A c Clostridia A o Christensenellales
acsB-acsV- GCA 004557125.1 d Bacteria p Firmicutes A c Clostridia A o Christensenellales
acsB- 5X acsV- GCA 004563405.1 d Bacteria p Firmicutes A c Clostridia A o Christensenellales
acsB- GCA 900553265.1 d Bacteria p Firmicutes A c Clostridia A o Christensenellales
acsB- GCA 902796005.1 d Bacteria p Firmicutes A c Clostridia o Lachnospirales
acsB- GCA 902765305.1 d Bacteria p Firmicutes A c Clostridia A o Christensenellales
acsB- 18X acsC acsD cooC2 GCA 902798105.1 d Bacteria p Firmicutes A c Clostridia A o Christensenellales
acsB- 6X fhs-fchA-folD- GCA 900317565.1 d Bacteria p Firmicutes A c Clostridia A o Christensenellales
acsB- GCA 902799465.1 d Bacteria p Firmicutes A c Clostridia A o Christensenellales
cooC2-acsD-acsC-acsE-acsB-acsV- 6X fhs-fchA-folD- GCA 000432015.1 d Bacteria p Firmicutes A c Clostridia A o Christensenellales
cooC2-acsD-acsC-acsE- 8X acsB-acsV- 8X fhs-fchA-folD- GCA 900549475.1 d Bacteria p Firmicutes A c Clostridia o Oscillospirales
cooC2-acsD-acsC-acsE- 1X acsB-acsV- GCA 900555795.1 d Bacteria p Firmicutes A c Clostridia o Oscillospirales
acsB-acsV- GCA 902373635.1 d Bacteria p Firmicutes A c Clostridia o Oscillospirales
acsB- GCA 009774055.1 d Bacteria p Firmicutes A c Clostridia o Oscillospirales
acsV- 3X acsA-cooC-acsB- 3X acsD-acsC-acsE-acsB- 1X fhs-fchA-folD- GCA 900317045.1 d Bacteria p Firmicutes A c Clostridia o Lachnospirales
cooC2-acsD-acsC-acsE-acsB- GCA 002436765.1 d Bacteria p Firmicutes A c Clostridia o Oscillospirales
cooC2-acsD-acsC-acsE-acsB- 1X acsV- GCA 900546075.1 d Bacteria p Firmicutes A c Clostridia o Oscillospirales
cooC2-acsD-acsC-acsE-acsB- 1X acsV- GCA 000434635.1 d Bacteria p Firmicutes A c Clostridia o Oscillospirales
acsE- 1X acsB-acsV- GCA 004562395.1 d Bacteria p Firmicutes A c Clostridia o Oscillospirales
cooC2- 2X acsC-acsE- 1X acsB- GCA 002362145.1 d Bacteria p Firmicutes A c Clostridia o Oscillospirales
cooC2-acsD-acsC-acsE- 1X acsB- GCA 900762245.1 d Bacteria p Firmicutes A c Clostridia o Oscillospirales
acsE- 1X acsB-acsV- GCA 900769845.1 d Bacteria p Firmicutes A c Clostridia o Oscillospirales
acsB- GCA 900769835.1 d Bacteria p Firmicutes A c Clostridia o Oscillospirales
cooC2-acsD-acsC-acsE- 3X acsB- GCA 900752285.1 d Bacteria p Firmicutes A c Clostridia o Oscillospirales
cooC2-acsD-acsC-acsE- 1X acsB- 1X acsV- GCA 002437585.1 d Bacteria p Firmicutes A c Clostridia o Oscillospirales
cooC2-acsD-acsC-acsE- 1X acsB- 1X acsV- GCA 900544945.1 d Bacteria p Firmicutes A c Clostridia o Oscillospirales
cooC2-acsD-acsC-acsE-acsB-acsV- GCA 900540635.1 d Bacteria p Firmicutes A c Clostridia o Oscillospirales
acsC-acsE-acsB- GCA 900549495.1 d Bacteria p Firmicutes A c Clostridia o Oscillospirales
metV- 2X metV-metF-cooC2-acsD-acsC-acsE-acsB- 13X acsV GCA 900553805.1 d Bacteria p Firmicutes A c Clostridia o Oscillospirales
acsB- 2X acsV- GCA 900549705.1 d Bacteria p Firmicutes A c Clostridia o Oscillospirales
cooC2-acsD-acsC-acsE- 1X acsB- 6X acsV- GCA 002493965.1 d Bacteria p Firmicutes A c Clostridia o Oscillospirales
cooC2-acsD-acsC-acsE- 2X acsB- 1X acsV GCA 900544405.1 d Bacteria p Firmicutes A c Clostridia o Oscillospirales
acsD-acsC-acsE- 1X acsB- 4X acsV- GCA 900551495.1 d Bacteria p Firmicutes A c Clostridia o Oscillospirales
cooC2-acsD-acsC-acsE-acsB- GCA 900554705.1 d Bacteria p Firmicutes A c Clostridia o Oscillospirales
acsC-acsE-acsB-acsB- GCA 900769995.1 d Bacteria p Firmicutes A c Clostridia o Oscillospirales
acsB- 2X acsV- GCA 004558975.1 d Bacteria p Firmicutes A c Clostridia o Oscillospirales
cooC2-acsD-acsC-acsE-acsB-acsV- 3X fhs-fchA-folD- GCA 900544705.1 d Bacteria p Firmicutes A c Clostridia o Oscillospirales
acsB- GCA 003525905.1 d Bacteria p Firmicutes A c Clostridia o Oscillospirales
acsA-cooC-acsB-acsB-acsV- 2X cooC2-acsD-acsC-acsE- GCA 900545655.1 d Bacteria p Firmicutes A c Clostridia o Oscillospirales
acsC-acsE-acsB-acsV- 3X fhs-fchA-folD- GCA 900555735.1 d Bacteria p Firmicutes A c Clostridia o Oscillospirales
acsA-cooC-acsB- 10X cooC2-acsD-acsC-acsE-acsB-acsV- 3X fhs- 1X fchA-folD- GCA 900757415.1 d Bacteria p Firmicutes A c Clostridia o Oscillospirales
acsB-acsV- 3X fhs- 1X fchA-folD- GCA 900548615.1 d Bacteria p Firmicutes A c Clostridia o Oscillospirales
cooC2-acsD-acsC-acsE-acsB-acsV- 1X fhs-fchA-folD- GCA 004555655.1 d Bacteria p Firmicutes A c Clostridia o Oscillospirales
cooC2-acsD-acsC-acsE-acsB-acsV- 3X fhs-fchA-folD- GCA 900553545.1 d Bacteria p Firmicutes A c Clostridia o Oscillospirales
acsB- GCA 900555665.1 d Bacteria p Firmicutes A c Clostridia o Oscillospirales
cooC2-acsD-acsC-acsE-acsB-acsV- 4X fhs-fchA-folD- GCA 900549635.1 d Bacteria p Firmicutes A c Clostridia o Oscillospirales
cooC2-acsD-acsC-acsE-acsB-acsV- 7X fhs-fchA-folD- GCA 900548625.1 d Bacteria p Firmicutes A c Clostridia o Oscillospirales
acsB- GCA 002404795.1 d Bacteria p Firmicutes A c Clostridia o Oscillospirales
cooC2-acsD-acsC-acsE-acsB- 8X fhs-fchA-folD- GCA 000436735.1 d Bacteria p Firmicutes A c Clostridia o Oscillospirales
cooC2-acsD-acsC-acsE-acsB- GCA 000432135.1 d Bacteria p Firmicutes A c Clostridia o Oscillospirales
cooC2-acsD-acsC-acsE-acsB- GCA 900545925.1 d Bacteria p Firmicutes A c Clostridia o Oscillospirales

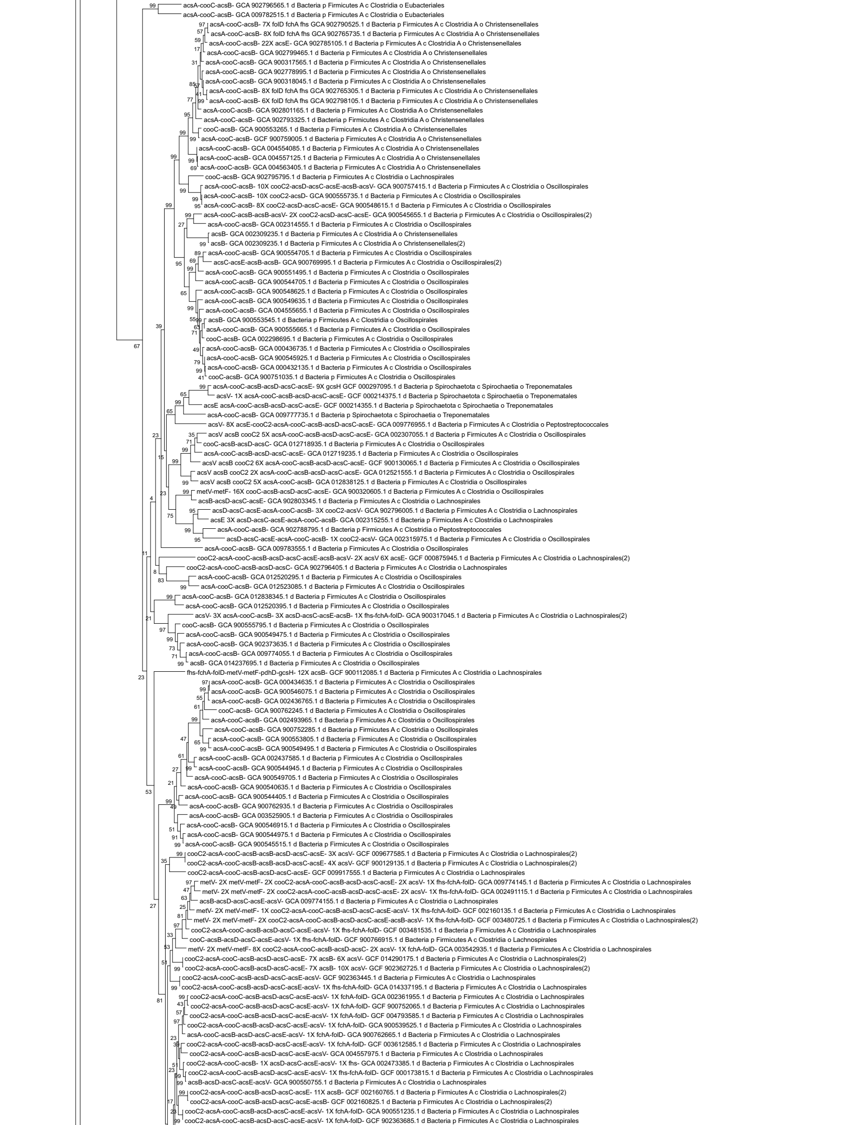
acsA-cooC-acsB- GCA 902796565.1 d Bacteria p Firmicutes A c Clostridia o Eubacteriales
acsA-cooC-acsB- GCA 009782515.1 d Bacteria p Firmicutes A c Clostridia o Eubacteriales
acsA-cooC-acsB- 7X folD fchA fhs GCA 902790525.1 d Bacteria p Firmicutes A c Clostridia A o Christensenellales
acsA-cooC-acsB- 8X folD fchA fhs GCA 902765735.1 d Bacteria p Firmicutes A c Clostridia A o Christensenellales
acsA-cooC-acsB- 22X acsE- GCA 902785105.1 d Bacteria p Firmicutes A c Clostridia A o Christensenellales
acsA-cooC-acsB- GCA 902799465.1 d Bacteria p Firmicutes A c Clostridia A o Christensenellales
acsA-cooC-acsB- GCA 900317565.1 d Bacteria p Firmicutes A c Clostridia A o Christensenellales
acsA-cooC-acsB- GCA 902778995.1 d Bacteria p Firmicutes A c Clostridia A o Christensenellales
acsA-cooC-acsB- GCA 900318045.1 d Bacteria p Firmicutes A c Clostridia A o Christensenellales
acsA-cooC-acsB- 8X folD fchA fhs GCA 902765305.1 d Bacteria p Firmicutes A c Clostridia A o Christensenellales
acsA-cooC-acsB- 6X folD fchA fhs GCA 902798105.1 d Bacteria p Firmicutes A c Clostridia A o Christensenellales
acsA-cooC-acsB- GCA 902801165.1 d Bacteria p Firmicutes A c Clostridia A o Christensenellales
acsA-cooC-acsB- GCA 902793325.1 d Bacteria p Firmicutes A c Clostridia A o Christensenellales
cooC-acsB- GCA 900553265.1 d Bacteria p Firmicutes A c Clostridia A o Christensenellales
acsA-cooC-acsB- GCF 900759005.1 d Bacteria p Firmicutes A c Clostridia A o Christensenellales
acsA-cooC-acsB- GCA 004554085.1 d Bacteria p Firmicutes A c Clostridia A o Christensenellales
acsA-cooC-acsB- GCA 004557125.1 d Bacteria p Firmicutes A c Clostridia A o Christensenellales
acsA-cooC-acsB- GCA 004563405.1 d Bacteria p Firmicutes A c Clostridia A o Christensenellales
cooC-acsB- GCA 902795795.1 d Bacteria p Firmicutes A c Clostridia o Lachnospirales
acsA-cooC-acsB- 10X cooC2-acsD-acsC-acsE-acsB-acsV- GCA 900757415.1 d Bacteria p Firmicutes A c Clostridia o Oscillospirales
acsA-cooC-acsB- 10X cooC2-acsD- GCA 900555735.1 d Bacteria p Firmicutes A c Clostridia o Oscillospirales
acsA-cooC-acsB- 8X cooC2-acsD-acsC-acsE- GCA 900548615.1 d Bacteria p Firmicutes A c Clostridia o Oscillospirales
acsA-cooC-acsB-acsB-acsV- 2X cooC2-acsD-acsC-acsE- GCA 900545655.1 d Bacteria p Firmicutes A c Clostridia o Oscillospirales(2)
acsA-cooC-acsB- GCA 002314555.1 d Bacteria p Firmicutes A c Clostridia o Oscillospirales
acsB- GCA 002309235.1 d Bacteria p Firmicutes A c Clostridia A o Christensenellales
acsB- GCA 002309235.1 d Bacteria p Firmicutes A c Clostridia A o Christensenellales(2)
acsA-cooC-acsB- GCA 900554705.1 d Bacteria p Firmicutes A c Clostridia o Oscillospirales
acsC-acsE-acsB-acsB- GCA 900769995.1 d Bacteria p Firmicutes A c Clostridia o Oscillospirales(2)
acsA-cooC-acsB- GCA 900551495.1 d Bacteria p Firmicutes A c Clostridia o Oscillospirales
acsA-cooC-acsB- GCA 900544705.1 d Bacteria p Firmicutes A c Clostridia o Oscillospirales
acsA-cooC-acsB- GCA 900548625.1 d Bacteria p Firmicutes A c Clostridia o Oscillospirales
acsA-cooC-acsB- GCA 900549635.1 d Bacteria p Firmicutes A c Clostridia o Oscillospirales
acsA-cooC-acsB- GCA 004555655.1 d Bacteria p Firmicutes A c Clostridia o Oscillospirales
acsB- GCA 900553545.1 d Bacteria p Firmicutes A c Clostridia o Oscillospirales
acsA-cooC-acsB- GCA 900555665.1 d Bacteria p Firmicutes A c Clostridia o Oscillospirales
cooC-acsB- GCA 002298695.1 d Bacteria p Firmicutes A c Clostridia o Oscillospirales
acsA-cooC-acsB- GCA 000436735.1 d Bacteria p Firmicutes A c Clostridia o Oscillospirales
acsA-cooC-acsB- GCA 900545925.1 d Bacteria p Firmicutes A c Clostridia o Oscillospirales
acsA-cooC-acsB- GCA 000432135.1 d Bacteria p Firmicutes A c Clostridia o Oscillospirales
cooC-acsB- GCA 900751035.1 d Bacteria p Firmicutes A c Clostridia o Oscillospirales
acsA-cooC-acsB-acsD-acsC-acsE- 9X gcsH GCF 000297095.1 d Bacteria p Spirochaetota c Spirochaetia o Treponematales
acsV- 1X acsA-cooC-acsB-acsD-acsC-acsE- GCF 000214375.1 d Bacteria p Spirochaetota c Spirochaetia o Treponematales
acsE acsA-cooC-acsB-acsD-acsC-acsE- GCF 000214355.1 d Bacteria p Spirochaetota c Spirochaetia o Treponematales
acsA-cooC-acsB- GCA 009777735.1 d Bacteria p Spirochaetota c Spirochaetia o Treponematales
acsV- 8X acsE-cooC2-acsA-cooC-acsB-acsD-acsC-acsE- GCA 009776955.1 d Bacteria p Firmicutes A c Clostridia o Peptostreptococcales
acsV acsB cooC2 5X acsA-cooC-acsB-acsD-acsC-acsE- GCA 002307055.1 d Bacteria p Firmicutes A c Clostridia o Oscillospirales
cooC-acsB-acsD-acsC- GCA 012718935.1 d Bacteria p Firmicutes A c Clostridia o Oscillospirales
acsA-cooC-acsB-acsD-acsC-acsE- GCA 012719235.1 d Bacteria p Firmicutes A c Clostridia o Oscillospirales
acsV acsB cooC2 6X acsA-cooC-acsB-acsD-acsC-acsE- GCF 900130065.1 d Bacteria p Firmicutes A c Clostridia o Oscillospirales
acsV acsB cooC2 2X acsA-cooC-acsB-acsD-acsC-acsE- GCA 012521555.1 d Bacteria p Firmicutes A c Clostridia o Oscillospirales
acsV acsB cooC2 5X acsA-cooC-acsB- GCA 012838125.1 d Bacteria p Firmicutes A c Clostridia o Oscillospirales
metV-metF- 16X cooC-acsB-acsD-acsC-acsE- GCA 900320605.1 d Bacteria p Firmicutes A c Clostridia o Oscillospirales
acsB-acsD-acsC-acsE- GCA 902803345.1 d Bacteria p Firmicutes A c Clostridia o Lachnospirales
acsD-acsC-acsE-acsA-cooC-acsB- 3X cooC2-acsV- GCA 902796005.1 d Bacteria p Firmicutes A c Clostridia o Lachnospirales
acsE 3X acsD-acsC-acsE-acsA-cooC-acsB- GCA 002315255.1 d Bacteria p Firmicutes A c Clostridia o Lachnospirales
acsA-cooC-acsB- GCA 902788795.1 d Bacteria p Firmicutes A c Clostridia o Peptostreptococcales
acsD-acsC-acsE-acsA-cooC-acsB- 1X cooC2-acsV- GCA 002315975.1 d Bacteria p Firmicutes A c Clostridia o Oscillospirales
acsA-cooC-acsB- GCA 009783555.1 d Bacteria p Firmicutes A c Clostridia o Oscillospirales
cooC2-acsA-cooC-acsB-acsD-acsC-acsE-acsB-acsV- 2X acsV 6X acsE- GCF 000875945.1 d Bacteria p Firmicutes A c Clostridia o Lachnospirales(2)
cooC2-acsA-cooC-acsB-acsD-acsC- GCA 902796405.1 d Bacteria p Firmicutes A c Clostridia o Lachnospirales
acsA-cooC-acsB- GCA 012520295.1 d Bacteria p Firmicutes A c Clostridia o Oscillospirales
acsA-cooC-acsB- GCA 012523085.1 d Bacteria p Firmicutes A c Clostridia o Oscillospirales
acsA-cooC-acsB- GCA 012838345.1 d Bacteria p Firmicutes A c Clostridia o Oscillospirales
acsA-cooC-acsB- GCA 012520395.1 d Bacteria p Firmicutes A c Clostridia o Oscillospirales
acsV- 3X acsA-cooC-acsB- 3X acsD-acsC-acsE-acsB- 1X fhs-fchA-folD- GCA 900317045.1 d Bacteria p Firmicutes A c Clostridia o Lachnospirales(2)
cooC-acsB- GCA 900555795.1 d Bacteria p Firmicutes A c Clostridia o Oscillospirales
acsA-cooC-acsB- GCA 900549475.1 d Bacteria p Firmicutes A c Clostridia o Oscillospirales
acsA-cooC-acsB- GCA 902373635.1 d Bacteria p Firmicutes A c Clostridia o Oscillospirales
acsA-cooC-acsB- GCA 009774055.1 d Bacteria p Firmicutes A c Clostridia o Oscillospirales
acsB- GCA 014237695.1 d Bacteria p Firmicutes A c Clostridia o Oscillospirales
fhs-fchA-folD-metV-metF-pdhD-gcsH- 12X acsB- GCF 900112085.1 d Bacteria p Firmicutes A c Clostridia o Lachnospirales
acsA-cooC-acsB- GCA 000434635.1 d Bacteria p Firmicutes A c Clostridia o Oscillospirales
acsA-cooC-acsB- GCA 900546075.1 d Bacteria p Firmicutes A c Clostridia o Oscillospirales
acsA-cooC-acsB- GCA 002436765.1 d Bacteria p Firmicutes A c Clostridia o Oscillospirales
cooC-acsB- GCA 900762245.1 d Bacteria p Firmicutes A c Clostridia o Oscillospirales
acsA-cooC-acsB- GCA 002493965.1 d Bacteria p Firmicutes A c Clostridia o Oscillospirales
acsA-cooC-acsB- GCA 900752285.1 d Bacteria p Firmicutes A c Clostridia o Oscillospirales
acsA-cooC-acsB- GCA 900553805.1 d Bacteria p Firmicutes A c Clostridia o Oscillospirales
acsA-cooC-acsB- GCA 900549495.1 d Bacteria p Firmicutes A c Clostridia o Oscillospirales
acsA-cooC-acsB- GCA 002437585.1 d Bacteria p Firmicutes A c Clostridia o Oscillospirales
acsA-cooC-acsB- GCA 900544945.1 d Bacteria p Firmicutes A c Clostridia o Oscillospirales
acsA-cooC-acsB- GCA 900549705.1 d Bacteria p Firmicutes A c Clostridia o Oscillospirales
acsA-cooC-acsB- GCA 900540635.1 d Bacteria p Firmicutes A c Clostridia o Oscillospirales
acsA-cooC-acsB- GCA 900544405.1 d Bacteria p Firmicutes A c Clostridia o Oscillospirales
acsA-cooC-acsB- GCA 900762935.1 d Bacteria p Firmicutes A c Clostridia o Oscillospirales
acsA-cooC-acsB- GCA 003525905.1 d Bacteria p Firmicutes A c Clostridia o Oscillospirales
acsA-cooC-acsB- GCA 900546915.1 d Bacteria p Firmicutes A c Clostridia o Oscillospirales
acsA-cooC-acsB- GCA 900544975.1 d Bacteria p Firmicutes A c Clostridia o Oscillospirales
acsA-cooC-acsB- GCA 900545515.1 d Bacteria p Firmicutes A c Clostridia o Oscillospirales
cooC2-acsA-cooC-acsB-acsB-acsD-acsC-acsE- 3X acsV- GCF 009677585.1 d Bacteria p Firmicutes A c Clostridia o Lachnospirales(2)
cooC2-acsA-cooC-acsB-acsB-acsD-acsC-acsE- 4X acsV- GCF 900129135.1 d Bacteria p Firmicutes A c Clostridia o Lachnospirales(2)
cooC2-acsA-cooC-acsB-acsD-acsC-acsE- GCF 009917555.1 d Bacteria p Firmicutes A c Clostridia o Lachnospirales
metV- 2X metV-metF- 2X cooC2-acsA-cooC-acsB-acsD-acsC-acsE- 2X acsV- 1X fhs-fchA-folD- GCA 009774145.1 d Bacteria p Firmicutes A c Clostridia o Lachnospirales
metV- 2X metV-metF- 2X cooC2-acsA-cooC-acsB-acsD-acsC-acsE- 2X acsV- 1X fhs-fchA-folD- GCA 002491115.1 d Bacteria p Firmicutes A c Clostridia o Lachnospirales
acsB-acsD-acsC-acsE-acsV- GCA 009774155.1 d Bacteria p Firmicutes A c Clostridia o Lachnospirales
metV- 2X metV-metF- 1X cooC2-acsA-cooC-acsB-acsD-acsC-acsE-acsV- 1X fhs-fchA-folD- GCF 002160135.1 d Bacteria p Firmicutes A c Clostridia o Lachnospirales
metV- 2X metV-metF- 2X cooC2-acsA-cooC-acsB-acsD-acsC-acsE-acsB-acsV- 1X fhs-fchA-folD- GCF 003480725.1 d Bacteria p Firmicutes A c Clostridia o Lachnospirales(2)
cooC2-acsA-cooC-acsB-acsD-acsC-acsE-acsV- 1X fhs-fchA-folD- GCF 003481535.1 d Bacteria p Firmicutes A c Clostridia o Lachnospirales
cooC-acsB-acsD-acsC-acsE-acsV- 1X fhs-fchA-folD- GCF 900766915.1 d Bacteria p Firmicutes A c Clostridia o Lachnospirales
metV- 2X metV-metF- 8X cooC2-acsA-cooC-acsB-acsD-acsC- 2X acsV- 1X fchA-folD- GCA 003542935.1 d Bacteria p Firmicutes A c Clostridia o Lachnospirales
cooC2-acsA-cooC-acsB-acsD-acsC-acsE- 7X acsB- 6X acsV- GCF 014290175.1 d Bacteria p Firmicutes A c Clostridia o Lachnospirales(2)
cooC2-acsA-cooC-acsB-acsD-acsC-acsE- 7X acsB- 10X acsV- GCF 902362725.1 d Bacteria p Firmicutes A c Clostridia o Lachnospirales(2)
cooC2-acsA-cooC-acsB-acsD-acsC-acsE-acsV- GCF 902363445.1 d Bacteria p Firmicutes A c Clostridia o Lachnospirales
cooC2-acsA-cooC-acsB-acsD-acsC-acsE-acsV- 1X fhs-fchA-folD- GCA 014337195.1 d Bacteria p Firmicutes A c Clostridia o Lachnospirales
cooC2-acsA-cooC-acsB-acsD-acsC-acsE-acsV- 1X fchA-folD- GCA 002361955.1 d Bacteria p Firmicutes A c Clostridia o Lachnospirales
cooC2-acsA-cooC-acsB-acsD-acsC-acsE-acsV- 1X fchA-folD- GCF 900752065.1 d Bacteria p Firmicutes A c Clostridia o Lachnospirales
cooC2-acsA-cooC-acsB-acsD-acsC-acsE-acsV- 1X fchA-folD- GCF 004793585.1 d Bacteria p Firmicutes A c Clostridia o Lachnospirales
cooC2-acsA-cooC-acsB-acsD-acsC-acsE-acsV- 1X fchA-folD- GCA 900539525.1 d Bacteria p Firmicutes A c Clostridia o Lachnospirales
acsA-cooC-acsB-acsD-acsC-acsE-acsV- 1X fchA-folD- GCA 900762665.1 d Bacteria p Firmicutes A c Clostridia o Lachnospirales
cooC2-acsA-cooC-acsB-acsD-acsC-acsE-acsV- 1X fchA-folD- GCF 003612585.1 d Bacteria p Firmicutes A c Clostridia o Lachnospirales
cooC2-acsA-cooC-acsB-acsD-acsC-acsE-acsV- GCA 004557975.1 d Bacteria p Firmicutes A c Clostridia o Lachnospirales
cooC2-acsA-cooC-acsB- 1X acsD-acsC-acsE-acsV- 1X fhs- GCA 002473385.1 d Bacteria p Firmicutes A c Clostridia o Lachnospirales
cooC2-acsA-cooC-acsB-acsD-acsC-acsE-acsV- 1X fhs-fchA-folD- GCF 000173815.1 d Bacteria p Firmicutes A c Clostridia o Lachnospirales
acsB-acsD-acsC-acsE-acsV- GCA 900550755.1 d Bacteria p Firmicutes A c Clostridia o Lachnospirales
cooC2-acsA-cooC-acsB-acsD-acsC-acsE- 11X acsB- GCF 002160765.1 d Bacteria p Firmicutes A c Clostridia o Lachnospirales(2)
cooC2-acsA-cooC-acsB-acsD-acsC-acsE-acsB- GCF 002160825.1 d Bacteria p Firmicutes A c Clostridia o Lachnospirales(2)
cooC2-acsA-cooC-acsB-acsD-acsC-acsE-acsV- 1X fchA-folD- GCA 900551235.1 d Bacteria p Firmicutes A c Clostridia o Lachnospirales
cooC2-acsA-cooC-acsB-acsD-acsC-acsE-acsV- 1X fchA-folD- GCF 902363685.1 d Bacteria p Firmicutes A c Clostridia o Lachnospirales

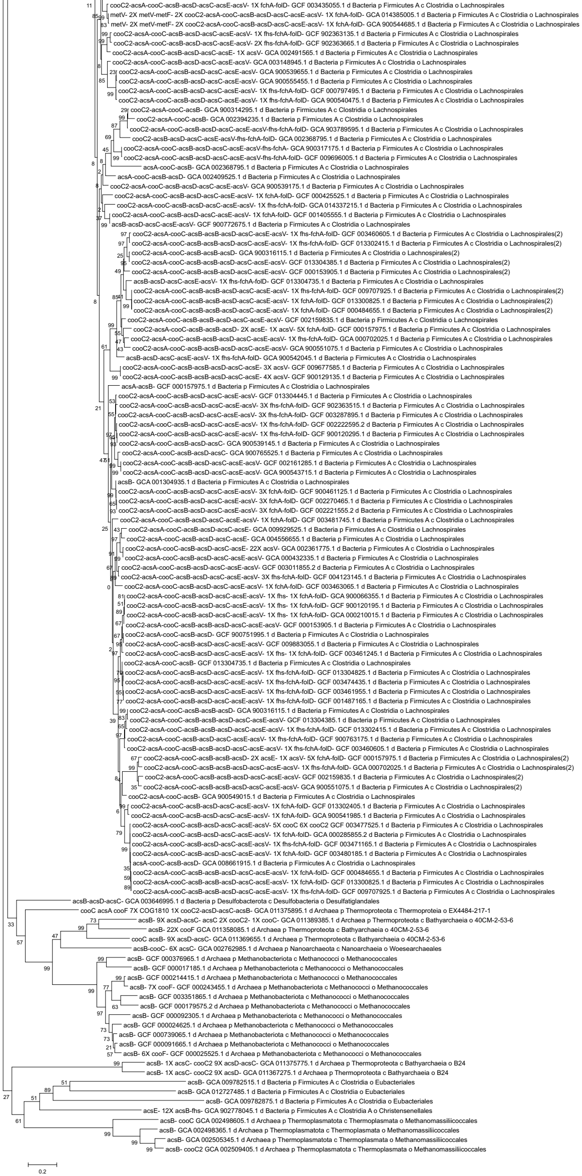

cooC2-acsA-cooC-acsB-acsD-acsC-acsE-acsV- 1X fchA-folD- GCF 003435055.1 d Bacteria p Firmicutes A c Clostridia o Lachnospirales
metV- 2X metV-metF- 2X cooC2-acsA-cooC-acsB-acsD-acsC-acsE-acsV- 1X fchA-folD- GCA 014385005.1 d Bacteria p Firmicutes A c Clostridia o Lachnospirales
metV- 2X metV-metF- 2X cooC2-acsA-cooC-acsB-acsD-acsC-acsE-acsV- 1X fchA-folD- GCA 900544685.1 d Bacteria p Firmicutes A c Clostridia o Lachnospirales
cooC2-acsA-cooC-acsB-acsD-acsC-acsE-acsV- 1X fhs-fchA-folD- GCF 902363135.1 d Bacteria p Firmicutes A c Clostridia o Lachnospirales
cooC2-acsA-cooC-acsB-acsD-acsC-acsE-acsV- 2X fhs-fchA-folD- GCF 902363665.1 d Bacteria p Firmicutes A c Clostridia o Lachnospirales
cooC2-acsA-cooC-acsB-acsD-acsC-acsE- 1X acsV- GCA 002491565.1 d Bacteria p Firmicutes A c Clostridia o Lachnospirales
cooC2-acsA-cooC-acsB-acsD-acsC-acsE-acsV- GCA 003148945.1 d Bacteria p Firmicutes A c Clostridia o Lachnospirales
cooC2-acsA-cooC-acsB-acsD-acsC-acsE-acsV- GCA 900539655.1 d Bacteria p Firmicutes A c Clostridia o Lachnospirales
cooC2-acsA-cooC-acsB-acsD-acsC-acsE-acsV- GCA 900555455.1 d Bacteria p Firmicutes A c Clostridia o Lachnospirales
cooC2-acsA-cooC-acsB-acsD-acsC-acsE-acsV- 1X fhs-fchA-folD- GCF 000797495.1 d Bacteria p Firmicutes A c Clostridia o Lachnospirales
cooC2-acsA-cooC-acsB-acsD-acsC-acsE-acsV- 1X fhs-fchA-folD- GCA 900540475.1 d Bacteria p Firmicutes A c Clostridia o Lachnospirales
cooC2-acsA-cooC-acsB- GCA 900314295.1 d Bacteria p Firmicutes A c Clostridia o Lachnospirales
cooC2-acsA-cooC-acsB- GCA 002394235.1 d Bacteria p Firmicutes A c Clostridia o Lachnospirales
cooC2-acsA-cooC-acsB-acsD-acsC-acsE-acsV-fhs-fchA-folD- GCA 903789595.1 d Bacteria p Firmicutes A c Clostridia o Lachnospirales
cooC2-acsB-acsD-acsC-acsE-acsV-fhs-fchA-folD- GCA 002368795.1 d Bacteria p Firmicutes A c Clostridia o Lachnospirales
cooC2-acsA-cooC-acsB-acsD-acsC-acsE-acsV-fhs-fchA- GCA 900317175.1 d Bacteria p Firmicutes A c Clostridia o Lachnospirales
cooC2-acsA-cooC-acsB-acsD-acsC-acsE-acsV-fhs-fchA-folD- GCF 009696005.1 d Bacteria p Firmicutes A c Clostridia o Lachnospirales
acsA-cooC-acsB- GCA 002368795.1 d Bacteria p Firmicutes A c Clostridia o Lachnospirales
acsA-cooC-acsB-acsD- GCA 002409525.1 d Bacteria p Firmicutes A c Clostridia o Lachnospirales
cooC2-acsA-cooC-acsB-acsD-acsC-acsE-acsV- GCA 900539175.1 d Bacteria p Firmicutes A c Clostridia o Lachnospirales
cooC2-acsA-cooC-acsB-acsD-acsC-acsE-acsV- 1X fchA-folD- GCF 000425525.1 d Bacteria p Firmicutes A c Clostridia o Lachnospirales
cooC2-acsA-cooC-acsB-acsD-acsC-acsE-acsV- 1X fhs-fchA-folD- GCA 014337215.1 d Bacteria p Firmicutes A c Clostridia o Lachnospirales
cooC2-acsA-cooC-acsB-acsD-acsC-acsE-acsV- 1X fchA-folD- GCF 001405555.1 d Bacteria p Firmicutes A c Clostridia o Lachnospirales
acsB-acsD-acsC-acsE-acsV- GCF 900772675.1 d Bacteria p Firmicutes A c Clostridia o Lachnospirales
cooC2-acsA-cooC-acsB-acsB-acsD-acsC-acsE-acsV- 1X fhs-fchA-folD- GCF 003460605.1 d Bacteria p Firmicutes A c Clostridia o Lachnospirales(2)
cooC2-acsA-cooC-acsB-acsB-acsD-acsC-acsE-acsV- 1X fhs-fchA-folD- GCF 013302415.1 d Bacteria p Firmicutes A c Clostridia o Lachnospirales(2)
cooC2-acsA-cooC-acsB-acsB-acsD- GCA 900316115.1 d Bacteria p Firmicutes A c Clostridia o Lachnospirales(2)
cooC2-acsA-cooC-acsB-acsB-acsD-acsC-acsE-acsV- GCF 013304385.1 d Bacteria p Firmicutes A c Clostridia o Lachnospirales(2)
cooC2-acsA-cooC-acsB-acsB-acsD-acsC-acsE-acsV- GCF 000153905.1 d Bacteria p Firmicutes A c Clostridia o Lachnospirales(2)
acsB-acsD-acsC-acsE-acsV- 1X fhs-fchA-folD- GCF 013304735.1 d Bacteria p Firmicutes A c Clostridia o Lachnospirales
cooC2-acsA-cooC-acsB-acsB-acsD-acsC-acsE-acsV- 1X fhs-fchA-folD- GCF 009707925.1 d Bacteria p Firmicutes A c Clostridia o Lachnospirales(2)
cooC2-acsA-cooC-acsB-acsB-acsD-acsC-acsE-acsV- 1X fchA-folD- GCF 013300825.1 d Bacteria p Firmicutes A c Clostridia o Lachnospirales(2)
cooC2-acsA-cooC-acsB-acsB-acsD-acsC-acsE-acsV- 1X fchA-folD- GCF 000484655.1 d Bacteria p Firmicutes A c Clostridia o Lachnospirales(2)
cooC2-acsA-cooC-acsB-acsB-acsD-acsC-acsE-acsV- GCF 002159835.1 d Bacteria p Firmicutes A c Clostridia o Lachnospirales
cooC2-acsA-cooC-acsB-acsB-acsD- 2X acsE- 1X acsV- 5X fchA-folD- GCF 000157975.1 d Bacteria p Firmicutes A c Clostridia o Lachnospirales
cooC2-acsA-cooC-acsB-acsB-acsD-acsC-acsE-acsV- 1X fhs-fchA-folD- GCA 000702025.1 d Bacteria p Firmicutes A c Clostridia o Lachnospirales
cooC2-acsA-cooC-acsB-acsB-acsD-acsC-acsE-acsV- GCA 900551075.1 d Bacteria p Firmicutes A c Clostridia o Lachnospirales
acsB-acsD-acsC-acsE-acsV- 1X fhs-fchA-folD- GCA 900542045.1 d Bacteria p Firmicutes A c Clostridia o Lachnospirales
cooC2-acsA-cooC-acsB-acsB-acsD-acsC-acsE- 3X acsV- GCF 009677585.1 d Bacteria p Firmicutes A c Clostridia o Lachnospirales
cooC2-acsA-cooC-acsB-acsB-acsD-acsC-acsE- 4X acsV- GCF 900129135.1 d Bacteria p Firmicutes A c Clostridia o Lachnospirales
acsA-acsB- GCF 000157975.1 d Bacteria p Firmicutes A c Clostridia o Lachnospirales
cooC2-acsA-cooC-acsB-acsD-acsC-acsE-acsV- GCF 013304445.1 d Bacteria p Firmicutes A c Clostridia o Lachnospirales
cooC2-acsA-cooC-acsB-acsD-acsC-acsE-acsV- 3X fhs-fchA-folD- GCF 902363515.1 d Bacteria p Firmicutes A c Clostridia o Lachnospirales
cooC2-acsA-cooC-acsB-acsD-acsC-acsE-acsV- 3X fhs-fchA-folD- GCF 003287895.1 d Bacteria p Firmicutes A c Clostridia o Lachnospirales
cooC2-acsA-cooC-acsB-acsD-acsC-acsE-acsV- 1X fhs-fchA-folD- GCF 002222595.2 d Bacteria p Firmicutes A c Clostridia o Lachnospirales
cooC2-acsA-cooC-acsB-acsD-acsC-acsE-acsV- 1X fhs-fchA-folD- GCF 900120295.1 d Bacteria p Firmicutes A c Clostridia o Lachnospirales
cooC2-acsA-cooC-acsB-acsD-acsC- GCA 900539145.1 d Bacteria p Firmicutes A c Clostridia o Lachnospirales
cooC2-acsA-cooC-acsB-acsD-acsC- GCA 900765525.1 d Bacteria p Firmicutes A c Clostridia o Lachnospirales
cooC2-acsA-cooC-acsB-acsD-acsC-acsE-acsV- GCF 002161285.1 d Bacteria p Firmicutes A c Clostridia o Lachnospirales
cooC2-acsA-cooC-acsB-acsD-acsC-acsE-acsV- GCA 900543715.1 d Bacteria p Firmicutes A c Clostridia o Lachnospirales
acsB- GCA 001304935.1 d Bacteria p Firmicutes A c Clostridia o Lachnospirales
cooC2-acsA-cooC-acsB-acsD-acsC-acsE-acsV- 3X fchA-folD- GCF 900461125.1 d Bacteria p Firmicutes A c Clostridia o Lachnospirales
cooC2-acsA-cooC-acsB-acsD-acsC-acsE-acsV- 3X fchA-folD- GCF 002270465.1 d Bacteria p Firmicutes A c Clostridia o Lachnospirales
cooC2-acsA-cooC-acsB-acsD-acsC-acsE-acsV- 3X fchA-folD- GCF 002221555.2 d Bacteria p Firmicutes A c Clostridia o Lachnospirales
cooC2-acsA-cooC-acsB-acsD-acsC-acsE-acsV- 1X fchA-folD- GCF 003481745.1 d Bacteria p Firmicutes A c Clostridia o Lachnospirales
cooC2-acsA-cooC-acsB-acsD-acsC-acsE- GCA 009929525.1 d Bacteria p Firmicutes A c Clostridia o Lachnospirales
cooC2-acsA-cooC-acsB-acsD-acsC-acsE- GCA 004556655.1 d Bacteria p Firmicutes A c Clostridia o Lachnospirales
cooC2-acsA-cooC-acsB-acsD-acsC-acsE- 22X acsV- GCA 002361775.1 d Bacteria p Firmicutes A c Clostridia o Lachnospirales
cooC2-acsA-cooC-acsB-acsD-acsC-acsE-acsV- GCA 000432335.1 d Bacteria p Firmicutes A c Clostridia o Lachnospirales
cooC2-acsA-cooC-acsB-acsD-acsC-acsE-acsV- GCF 003011855.2 d Bacteria p Firmicutes A c Clostridia o Lachnospirales
cooC2-acsA-cooC-acsB-acsD-acsC-acsE-acsV- 3X fhs-fchA-folD- GCF 004123145.1 d Bacteria p Firmicutes A c Clostridia o Lachnospirales
cooC2-acsA-cooC-acsB-acsD-acsC-acsE-acsV- 1X fchA-folD- GCF 003463065.1 d Bacteria p Firmicutes A c Clostridia o Lachnospirales
cooC2-acsA-cooC-acsB-acsD-acsC-acsE-acsV- 1X fhs- 1X fchA-folD- GCA 900066355.1 d Bacteria p Firmicutes A c Clostridia o Lachnospirales
cooC2-acsA-cooC-acsB-acsD-acsC-acsE-acsV- 1X fhs- 1X fchA-folD- GCF 900120195.1 d Bacteria p Firmicutes A c Clostridia o Lachnospirales
cooC2-acsA-cooC-acsB-acsD-acsC-acsE-acsV- 1X fhs- 1X fchA-folD- GCA 000210015.1 d Bacteria p Firmicutes A c Clostridia o Lachnospirales
cooC2-acsA-cooC-acsB-acsD-acsC-acsE-acsV- GCF 000153905.1 d Bacteria p Firmicutes A c Clostridia o Lachnospirales
cooC2-acsA-cooC-acsB-acsD- GCF 900751995.1 d Bacteria p Firmicutes A c Clostridia o Lachnospirales
cooC2-acsA-cooC-acsB-acsD-acsC-acsE-acsV- GCF 009883055.1 d Bacteria p Firmicutes A c Clostridia o Lachnospirales
cooC2-acsA-cooC-acsB-acsD-acsC-acsE-acsV- 1X fhs- 1X fchA-folD- GCF 003461245.1 d Bacteria p Firmicutes A c Clostridia o Lachnospirales
cooC2-acsA-cooC-acsB- GCF 013304735.1 d Bacteria p Firmicutes A c Clostridia o Lachnospirales
cooC2-acsA-cooC-acsB-acsD-acsC-acsE-acsV- 1X fhs-fchA-folD- GCF 013304825.1 d Bacteria p Firmicutes A c Clostridia o Lachnospirales
cooC2-acsA-cooC-acsB-acsD-acsC-acsE-acsV- 1X fhs-fchA-folD- GCF 003474435.1 d Bacteria p Firmicutes A c Clostridia o Lachnospirales
cooC2-acsA-cooC-acsB-acsD-acsC-acsE-acsV- 1X fhs-fchA-folD- GCF 003461955.1 d Bacteria p Firmicutes A c Clostridia o Lachnospirales
cooC2-acsA-cooC-acsB-acsD-acsC-acsE-acsV- 1X fhs-fchA-folD- GCF 001487165.1 d Bacteria p Firmicutes A c Clostridia o Lachnospirales
cooC2-acsA-cooC-acsB-acsB-acsD- GCA 900316115.1 d Bacteria p Firmicutes A c Clostridia o Lachnospirales
cooC2-acsA-cooC-acsB-acsB-acsD-acsC-acsE-acsV- GCF 013304385.1 d Bacteria p Firmicutes A c Clostridia o Lachnospirales
cooC2-acsA-cooC-acsB-acsB-acsD-acsC-acsE-acsV- 1X fhs-fchA-folD- GCF 013302415.1 d Bacteria p Firmicutes A c Clostridia o Lachnospirales
cooC2-acsA-cooC-acsB-acsD-acsC-acsE-acsV- 1X fhs-fchA-folD- GCF 900763175.1 d Bacteria p Firmicutes A c Clostridia o Lachnospirales
cooC2-acsA-cooC-acsB-acsB-acsD-acsC-acsE-acsV- 1X fhs-fchA-folD- GCF 003460605.1 d Bacteria p Firmicutes A c Clostridia o Lachnospirales
cooC2-acsA-cooC-acsB-acsB-acsD- 2X acsE- 1X acsV- 5X fchA-folD- GCF 000157975.1 d Bacteria p Firmicutes A c Clostridia o Lachnospirales(2)
cooC2-acsA-cooC-acsB-acsB-acsD-acsC-acsE-acsV- 1X fhs-fchA-folD- GCA 000702025.1 d Bacteria p Firmicutes A c Clostridia o Lachnospirales(2)
cooC2-acsA-cooC-acsB-acsB-acsD-acsC-acsE-acsV- GCF 002159835.1 d Bacteria p Firmicutes A c Clostridia o Lachnospirales(2)
cooC2-acsA-cooC-acsB-acsB-acsD-acsC-acsE-acsV- GCA 900551075.1 d Bacteria p Firmicutes A c Clostridia o Lachnospirales(2)
cooC2-acsA-cooC-acsB- GCA 900549015.1 d Bacteria p Firmicutes A c Clostridia o Lachnospirales
cooC2-acsA-cooC-acsB-acsD-acsC-acsE-acsV- 1X fchA-folD- GCF 013302405.1 d Bacteria p Firmicutes A c Clostridia o Lachnospirales
cooC2-acsA-cooC-acsB-acsD-acsC-acsE-acsV- 1X fchA-folD- GCA 900541985.1 d Bacteria p Firmicutes A c Clostridia o Lachnospirales
cooC2-acsA-cooC-acsB-acsD-acsC-acsE-acsV- 5X cooC 6X cooC2 GCF 003477525.1 d Bacteria p Firmicutes A c Clostridia o Lachnospirales
cooC2-acsA-cooC-acsB-acsD-acsC-acsE-acsV- 1X fchA-folD- GCA 000285855.2 d Bacteria p Firmicutes A c Clostridia o Lachnospirales
cooC2-acsA-cooC-acsB-acsD-acsC-acsE-acsV- 1X fhs-fchA-folD- GCF 003471165.1 d Bacteria p Firmicutes A c Clostridia o Lachnospirales
cooC2-acsA-cooC-acsB-acsD-acsC-acsE-acsV- 1X fchA-folD- GCF 003480185.1 d Bacteria p Firmicutes A c Clostridia o Lachnospirales
acsA-cooC-acsB-acsD- GCA 008661915.1 d Bacteria p Firmicutes A c Clostridia o Lachnospirales
cooC2-acsA-cooC-acsB-acsB-acsD-acsC-acsE-acsV- 1X fchA-folD- GCF 000484655.1 d Bacteria p Firmicutes A c Clostridia o Lachnospirales
cooC2-acsA-cooC-acsB-acsB-acsD-acsC-acsE-acsV- 1X fchA-folD- GCF 013300825.1 d Bacteria p Firmicutes A c Clostridia o Lachnospirales
cooC2-acsA-cooC-acsB-acsB-acsD-acsC-acsE-acsV- 1X fhs-fchA-folD- GCF 009707925.1 d Bacteria p Firmicutes A c Clostridia o Lachnospirales
acsB-acsD-acsC- GCA 003646995.1 d Bacteria p Desulfobacterota c Desulfobacteria o Desulfatiglandales
cooC acsA cooF 7X COG1810 1X cooC2-acsD-acsC-acsB- GCA 011375895.1 d Archaea p Thermoproteota c Thermoproteia o EX4484-217-1
acsB- 9X acsD-acsC- acsC 2X cooC2- 1X cooC- GCA 011389385.1 d Archaea p Thermoproteota c Bathyarchaeia o 40CM-2-53-6
acsB- 22X cooF GCA 011358085.1 d Archaea p Thermoproteota c Bathyarchaeia o 40CM-2-53-6
cooC acsB- 9X acsD-acsC- GCA 011369655.1 d Archaea p Thermoproteota c Bathyarchaeia o 40CM-2-53-6
acsB-cooC- 6X acsC- GCA 002762985.1 d Archaea p Nanoarchaeota c Nanoarchaeia o Woesearchaeales
acsB- GCF 000376965.1 d Archaea p Methanobacteriota c Methanococci o Methanococcales
acsB- GCF 000017185.1 d Archaea p Methanobacteriota c Methanococci o Methanococcales
acsB- GCF 000214415.1 d Archaea p Methanobacteriota c Methanococci o Methanococcales
acsB- 7X cooF- GCF 000243455.1 d Archaea p Methanobacteriota c Methanococci o Methanococcales
acsB- GCF 003351865.1 d Archaea p Methanobacteriota c Methanococci o Methanococcales
acsB- GCF 000179575.2 d Archaea p Methanobacteriota c Methanococci o Methanococcales
acsB- GCF 000092305.1 d Archaea p Methanobacteriota c Methanococci o Methanococcales
acsB- GCF 000024625.1 d Archaea p Methanobacteriota c Methanococci o Methanococcales
acsB- GCF 000739065.1 d Archaea p Methanobacteriota c Methanococci o Methanococcales
acsB- GCF 000091665.1 d Archaea p Methanobacteriota c Methanococci o Methanococcales
acsB- 6X cooF- GCF 000025525.1 d Archaea p Methanobacteriota c Methanococci o Methanococcales
acsB- 1X acsC- cooC2 9X acsD-acsC- GCA 011375775.1 d Archaea p Thermoproteota c Bathyarchaeia o B24
acsB- 1X acsC- cooC2 9X acsD- GCA 011367275.1 d Archaea p Thermoproteota c Bathyarchaeia o B24
acsB- GCA 009782515.1 d Bacteria p Firmicutes A c Clostridia o Eubacteriales
acsB- GCA 012727485.1 d Bacteria p Firmicutes A c Clostridia o Eubacteriales
acsB- GCA 009782875.1 d Bacteria p Firmicutes A c Clostridia o Eubacteriales
acsE- 12X acsB-fhs- GCA 902778045.1 d Bacteria p Firmicutes A c Clostridia A o Christensenellales
acsB- cooC GCA 002498605.1 d Archaea p Thermoplasmatota c Thermoplasmata o Methanomassiliicoccales
acsB- GCA 002498365.1 d Archaea p Thermoplasmatota c Thermoplasmata o Methanomassiliicoccales
acsB- GCA 002505345.1 d Archaea p Thermoplasmatota c Thermoplasmata o Methanomassiliicoccales
acsB- cooC2 GCA 002509405.1 d Archaea p Thermoplasmatota c Thermoplasmata o Methanomassiliicoccales
0.2
