# Supplementary material for: Obligate autotrophy at the thermodynamic limit of life in a new acetogenic bacterium
Source: Front Microbiol. 2023 May 12;14:1185739. doi: 10.3389/fmicb.2023.1185739 (PMC10213532; doi:10.3389/fmicb.2023.1185739)
Supplement: Supplementary file 6 [file Data_Sheet_6.PDF]

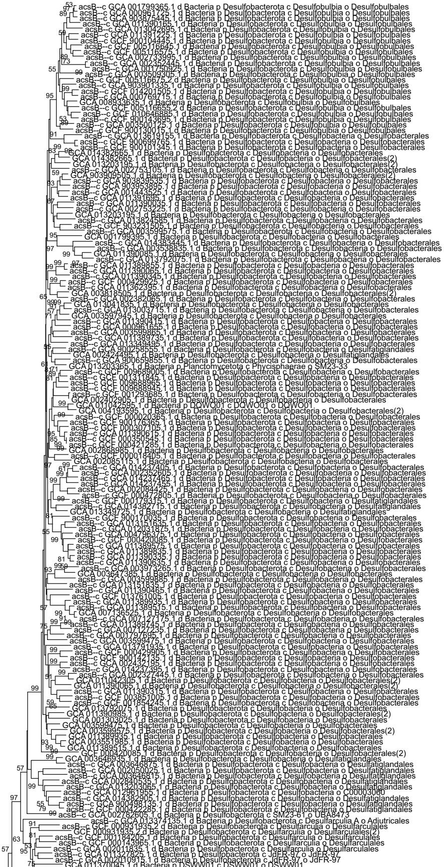

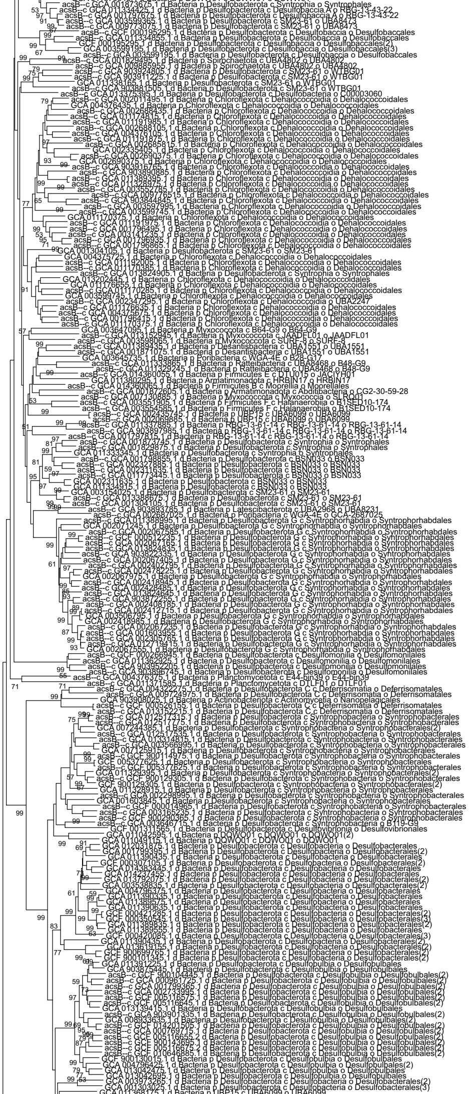

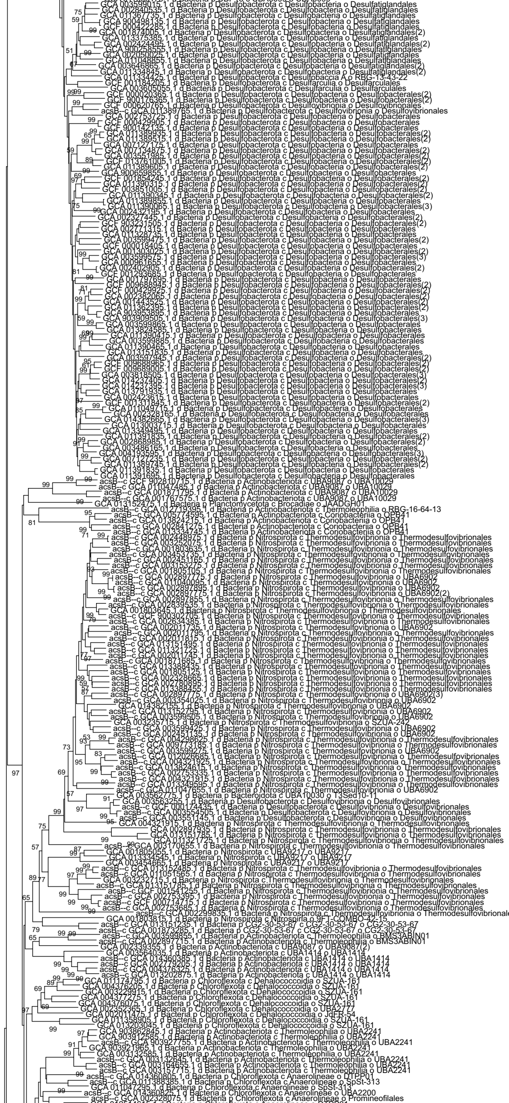

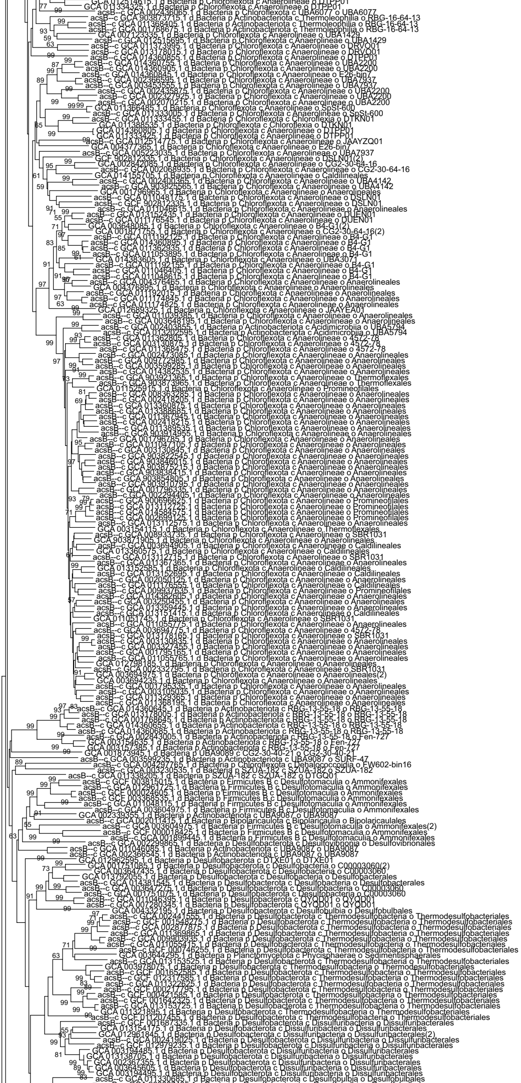

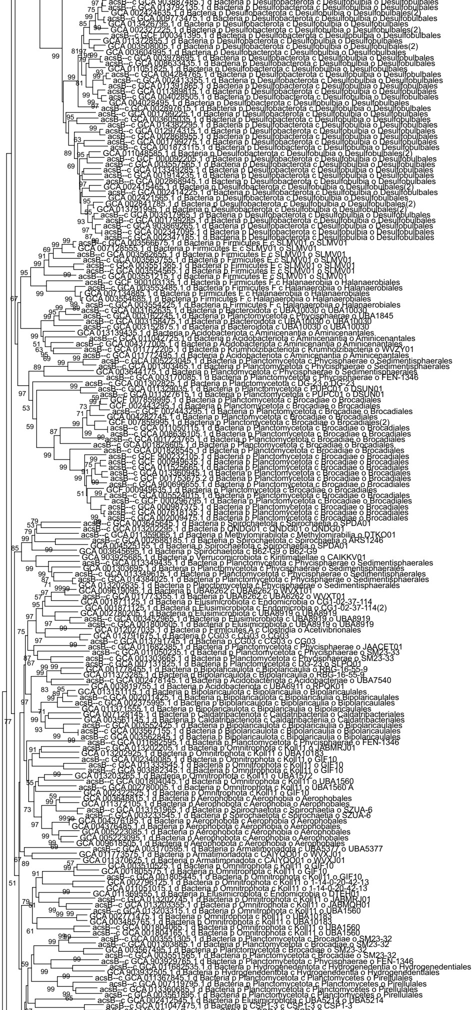

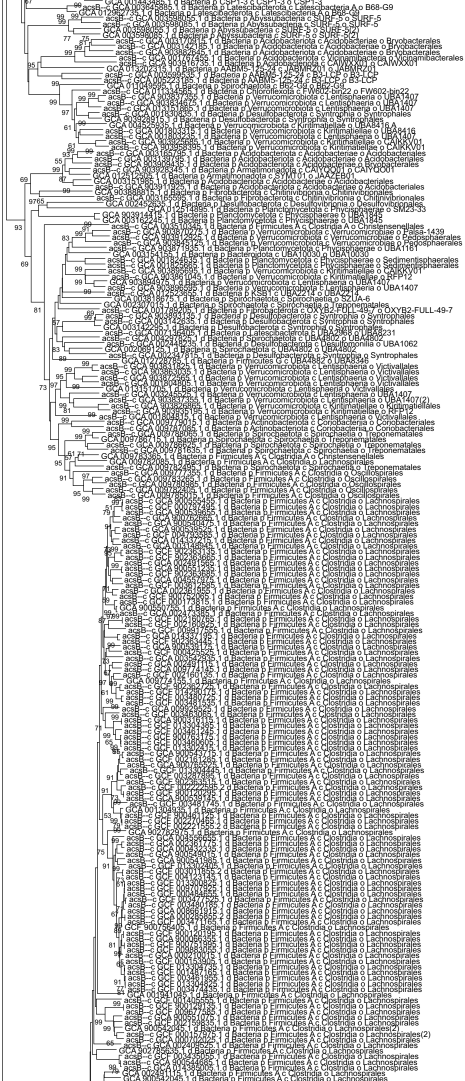

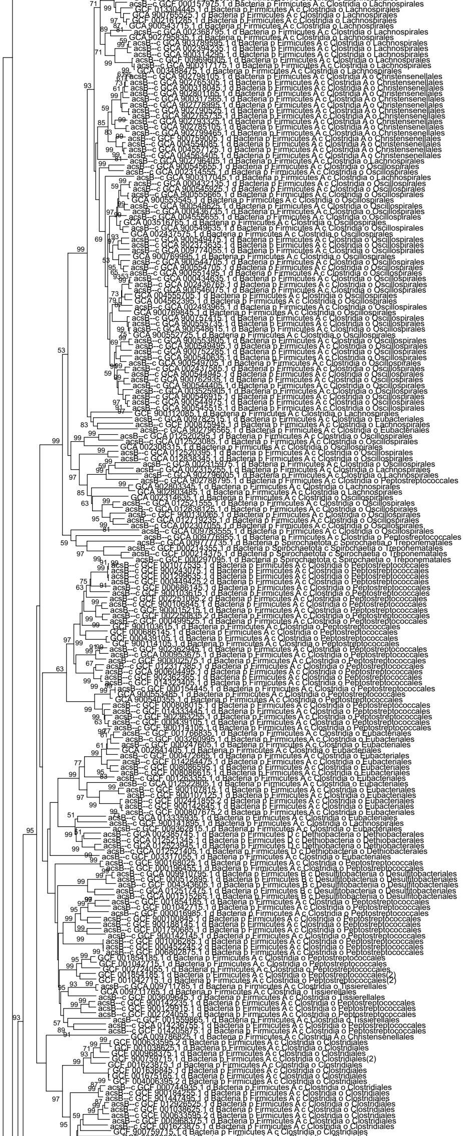

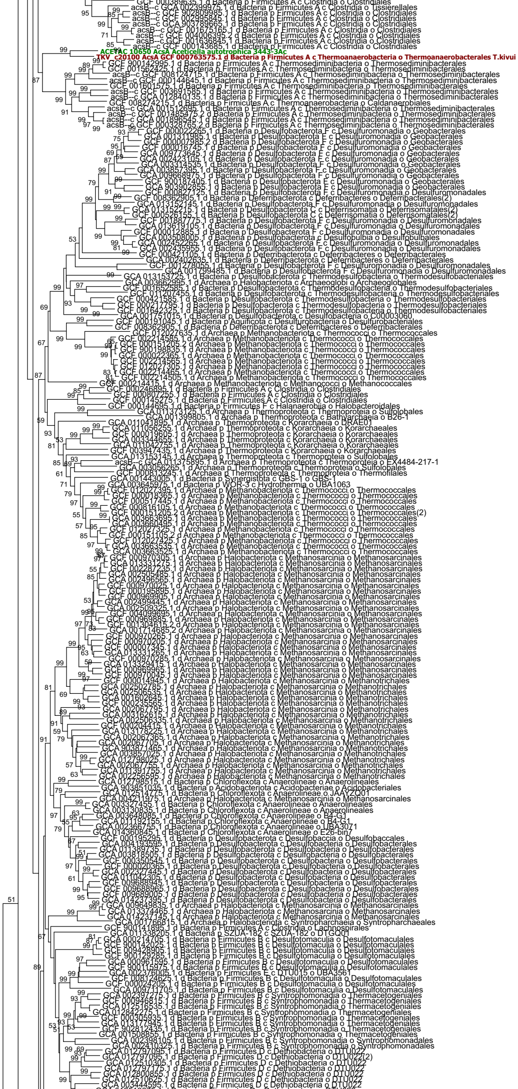
ACETAC 10650 AcsA Aceticella autotrophica 3443-3Ac
TKV_c20100 AcsA GCF 000763575.1 d Bacteria p Firmicutes A c Thermoanaerobacteria o Thermoanaerobacterales T.kivui

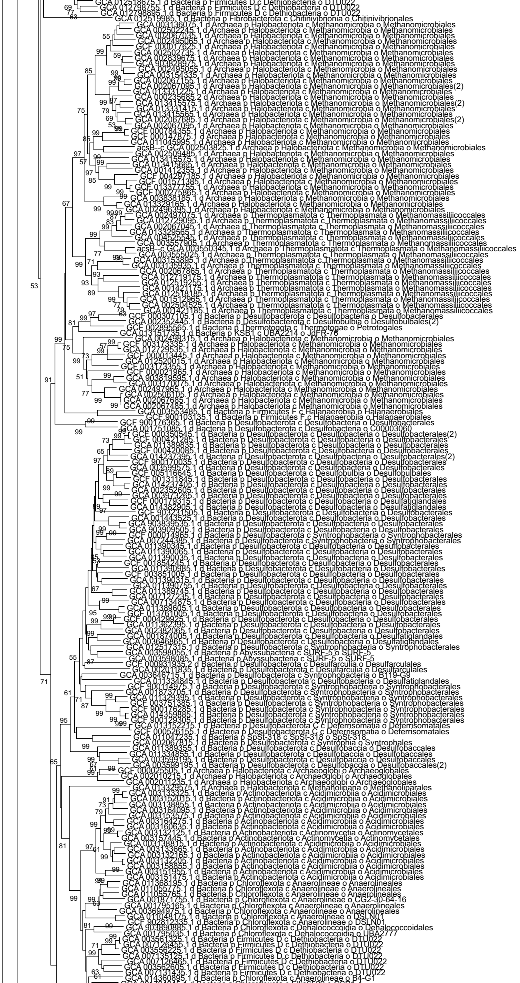

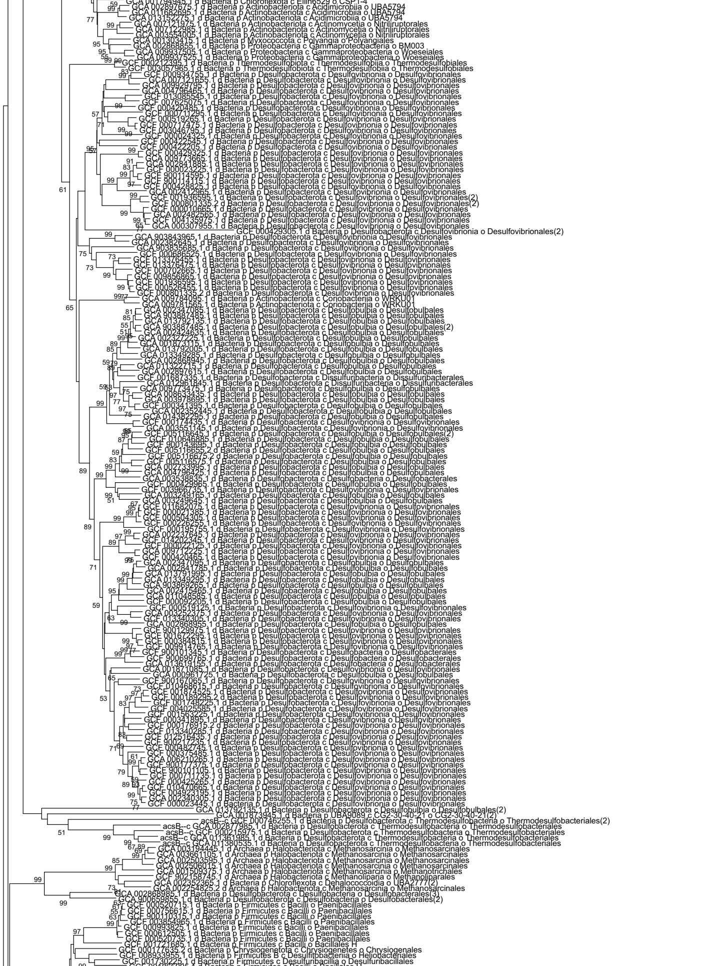

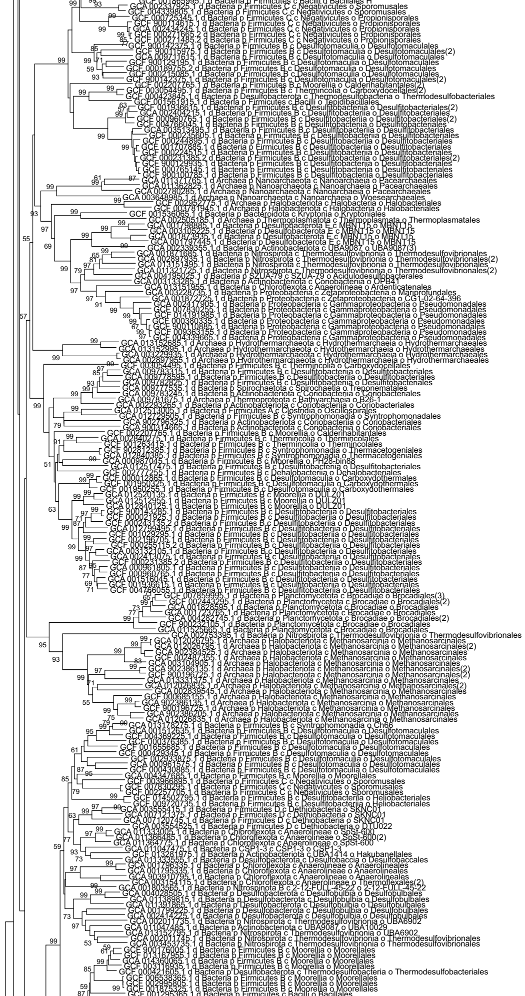
GCA 002337925.1 d Bacteria p Firmicutes C c Negativicutes o Sporomusales
GCF 004339805.1 d Bacteria p Firmicutes C c Negativicutes o Sporomusales
GCF 000725345.1 d Bacteria p Firmicutes C c Negativicutes o Propionisporales
GCF 900114615.1 d Bacteria p Firmicutes C c Negativicutes o Propionisporales
GCA 002434245.1 d Bacteria p Firmicutes C c Negativicutes o Propionisporales
GCF 900142375.1 d Bacteria p Firmicutes B c Desulfotomaculia o Desulfotomaculales
GCF 900115975.1 d Bacteria p Firmicutes B c Desulfotomaculia o Desulfotomaculales(2)
GCF 900129195.1 d Bacteria p Firmicutes B c Desulfotomaculia o Desulfotomaculales
GCF 000189755.2 d Bacteria p Firmicutes B c Desulfotomaculia o Desulfotomaculales
GCF 000215085.1 d Bacteria p Firmicutes B c Desulfotomaculia o Desulfotomaculales
GCF 900142375.1 d Bacteria p Firmicutes B c Desulfotomaculia o Desulfotomaculales(2)
GCF 002207765.1 d Bacteria p Firmicutes B c Moorellia o Calderihabitantales(2)
GCF 003054495.1 d Bacteria p Firmicutes B c Thermincolia o Carboxydocellales(2)
GCF 000423845.1 d Bacteria p Desulfobacterota c Thermodesulfobacteria o Thermodesulfobacteriales
GCF 001561915.1 d Bacteria p Firmicutes c Bacilli o Tepidibacillales
GCF 001936615.1 d Bacteria p Firmicutes B c Desulfitobacteriia o Desulfitobacteriales(2)
GCA 002404215.1 d Bacteria p Firmicutes B c Desulfitobacteriia o Desulfitobacteriales
GCF 000960765.1 d Bacteria p Firmicutes B c Desulfitobacteriia o Desulfitobacteriales(2)
GCA 003513495.1 d Bacteria p Firmicutes B c Desulfitobacteriia o Desulfitobacteriales
GCF 000235605.1 d Bacteria p Firmicutes B c Desulfitobacteriia o Desulfitobacteriales
GCF 000244895.1 d Bacteria p Firmicutes B c Desulfitobacteriia o Desulfitobacteriales
GCF 001707885.1 d Bacteria p Firmicutes B c Desulfitobacteriia o Desulfitobacteriales
GCF 000231385.2 d Bacteria p Firmicutes B c Desulfitobacteriia o Desulfitobacteriales(2)
GCF 900129935.1 d Bacteria p Firmicutes B c Desulfitobacteriia o Desulfitobacteriales
GCF 000765145.1 d Bacteria p Firmicutes B c Desulfitobacteriia o Desulfitobacteriales
GCA 011362825.1 d Archaea p Nanoarchaeota c Nanoarchaeia o Pacearchaeales
GCA 002780285.1 d Archaea p Nanoarchaeota c Nanoarchaeia o Pacearchaeales
GCA 003648985.1 d Archaea p Nanoarchaeota c Nanoarchaeia o Woesearchaeales
GCF 001536065.1 d Bacteria p Bacteroidota c Kryptonia o Kryptoniales
GCA 002505185.1 d Archaea p Thermoplasmatota c Thermoplasmata o Thermoplasmatales
GCA 002339355.1 d Bacteria p Actinobacteriota c UBA9087 o UBA9087(3)
GCA 001871685.1 d Bacteria p Nitrospirota c Thermodesulfovibrionia o Thermodesulfovibrionales
GCA 002897935.1 d Bacteria p Nitrospirota c Thermodesulfovibrionia o Thermodesulfovibrionales(2)
GCA 011321725.1 d Bacteria p Nitrospirota c Thermodesulfovibrionia o Thermodesulfovibrionales(2)
GCA 004195025.1 d Bacteria p SZUA-79 c SZUA-79 o Acidulodesulfobacterales
GCA 003133285.1 d Bacteria p Actinobacteriota c Coriobacteriia o OPB41
GCA 001872725.1 d Bacteria p Proteobacteria c Zetaproteobacteria o CG1-02-64-396
GCA 002417905.1 d Bacteria p Proteobacteria c Gammaproteobacteria o Pseudomonadales
GCF 007830255.1 d Bacteria p Proteobacteria c Gammaproteobacteria o Pseudomonadales
GCF 014191985.1 d Bacteria p Proteobacteria c Gammaproteobacteria o Pseudomonadales
GCF 000380335.1 d Bacteria p Proteobacteria c Gammaproteobacteria o Pseudomonadales
GCF 900110885.1 d Bacteria p Proteobacteria c Gammaproteobacteria o Pseudomonadales
GCF 009363155.1 d Bacteria p Proteobacteria c Gammaproteobacteria o Pseudomonadales
GCF 004339665.1 d Bacteria p Proteobacteria c Gammaproteobacteria o Pseudomonadales
GCA 013152685.1 d Archaea p Hydrothermarchaeota c Hydrothermarchaeia o Hydrothermarchaeales
GCA 003229935.1 d Archaea p Hydrothermarchaeota c Hydrothermarchaeia o Hydrothermarchaeales
GCA 002897955.1 d Archaea p Hydrothermarchaeota c Hydrothermarchaeia o Hydrothermarchaeales
GCF 003054495.1 d Bacteria p Firmicutes B c Thermincolia o Carboxydocellales
GCA 009777535.1 d Bacteria p Spirochaetota c Spirochaetia o Treponematales
GCA 009783245.1 d Bacteria p Actinobacteriota c Coriobacteriia o Coriobacteriales
GCA 009781675.1 d Archaea p Thermoproteota c Bathyarchaeia o B26-1
GCA 012513005.1 d Bacteria p Firmicutes A c Clostridia o Oscillospirales
GCA 012729505.1 d Bacteria p Firmicutes B c Syntrophomonadia o Syntrophomonadales
GCA 902796325.1 d Bacteria p Actinobacteriota c Coriobacteriia o Coriobacteriales
GCF 002207765.1 d Bacteria p Firmicutes B c Moorellia o Calderihabitantales
GCF 001263415.1 d Bacteria p Firmicutes B c Thermincolia o Thermincolales
GCF 902812385.1 d Bacteria p Firmicutes B c Syntrophomonadia o Thermacetogeniales
GCA 012517475.1 d Bacteria p Firmicutes B c Desulfitobacteriia o Desulfitobacteriales
GCF 002777255.1 d Bacteria p Firmicutes B c Dehalobacteriia o Dehalobacteriales
GCA 012520135.1 d Bacteria p Firmicutes B c Moorellia o DULZ01
GCA 012512955.1 d Bacteria p Firmicutes B c Moorellia o DULZ01
GCA 012840125.1 d Bacteria p Firmicutes B c Moorellia o DULZ01
GCF 000243135.2 d Bacteria p Firmicutes B c Desulfitobacteriia o Desulfitobacteriales
GCA 012799495.1 d Bacteria p Firmicutes B c Desulfitobacteriia o Desulfitobacteriales
GCA 003132105.1 d Bacteria p Firmicutes B c Desulfitobacteriia o Desulfitobacteriales
GCA 002413075.1 d Bacteria p Firmicutes B c Desulfitobacteriia o Desulfitobacteriales
GCF 000231385.2 d Bacteria p Firmicutes B c Desulfitobacteriia o Desulfitobacteriales
GCA 000961805.1 d Bacteria p Firmicutes B c Desulfitobacteriia o Desulfitobacteriales
GCF 001936615.1 d Bacteria p Firmicutes B c Desulfitobacteriia o Desulfitobacteriales
GCF 004766055.1 d Bacteria p Firmicutes B c Desulfitobacteriia o Desulfitobacteriales
GCF 007859995.1 d Bacteria p Planctomycetota c Brocadiae o Brocadiales(3)
GCF 002443295.1 d Bacteria p Planctomycetota c Brocadiae o Brocadiales(2)
GCA 001828595.1 d Bacteria p Planctomycetota c Brocadiae o Brocadiales
GCA 001723765.1 d Bacteria p Planctomycetota c Brocadiae o Brocadiales
GCA 004282745.1 d Bacteria p Planctomycetota c Brocadiae o Brocadiales(2)
GCF 900232105.1 d Bacteria p Planctomycetota c Brocadiae o Brocadiales
GCA 002753395.1 d Bacteria p Nitrospirota c Thermodesulfovibrionia o Thermodesulfovibrionales
GCA 012026795.1 d Archaea p Halobacteriota c Methanosarcinia o Methanosarcinales
GCA 012026795.1 d Archaea p Halobacteriota c Methanosarcinia o Methanosarcinales(2)
GCA 902384525.1 d Archaea p Halobacteriota c Methanosarcinia o Methanosarcinales
GCA 902386135.1 d Archaea p Halobacteriota c Methanosarcinia o Methanosarcinales(2)
GCF 900196725.1 d Archaea p Halobacteriota c Methanosarcinia o Methanosarcinales(2)
GCA 013331375.1 d Archaea p Halobacteriota c Methanosarcinia o Methanosarcinales
GCA 002839545.1 d Archaea p Halobacteriota c Methanosarcinia o Methanosarcinales
GCF 000685155.1 d Archaea p Halobacteriota c Methanosarcinia o Methanosarcinales
GCA 902386135.1 d Archaea p Halobacteriota c Methanosarcinia o Methanosarcinales
GCF 900196725.1 d Archaea p Halobacteriota c Methanosarcinia o Methanosarcinales
GCA 012026835.1 d Archaea p Halobacteriota c Methanosarcinia o Methanosarcinales
GCA 013178275.1 d Bacteria p Firmicutes B c Syntrophomonadia o Ch66
GCA 001512635.1 d Bacteria p Firmicutes B c Desulfotomaculia o Desulfotomaculales
GCF 004369225.1 d Bacteria p Firmicutes B c Desulfotomaculia o Desulfotomaculales
GCF 000429345.1 d Bacteria p Firmicutes B c Desulfotomaculia o Desulfotomaculales
GCF 002933875.1 d Bacteria p Firmicutes B c Desulfotomaculia o Desulfotomaculales
GCA 004347685.1 d Bacteria p Firmicutes B c Moorellia o Moorellales
GCF 003966895.1 d Bacteria p Firmicutes C c Negativicutes o Sporomusales
GCF 007830295.1 d Bacteria p Firmicutes C c Negativicutes o Sporomusales
GCF 002257705.1 d Bacteria p Firmicutes C c Negativicutes o Sporomusales
GCF 014502795.1 d Bacteria p Firmicutes B c Desulfitobacteriia o Heliobacteriales
GCF 009720735.1 d Bacteria p Firmicutes B c Desulfitobacteriia o Heliobacteriales
GCA 003555415.1 d Bacteria p Firmicutes D c Dethiobacteria o SKNC01
GCA 007121375.1 d Bacteria p Firmicutes D c Dethiobacteria o SKNC01
GCA 007120745.1 d Bacteria p Firmicutes D c Dethiobacteria o SKNC01
GCA 003554525.1 d Bacteria p Firmicutes D c Dethiobacteria o DTU022
GCA 011366485.1 d Bacteria p Chloroflexota c Anaerolineae o SpSt-600(2)
GCA 011364775.1 d Bacteria p Chloroflexota c Anaerolineae o SpSt-600
GCA 011047475.1 d Bacteria p CSP1-3 c CSP1-3 o CSP1-3
GCF 013281975.1 d Bacteria p Actinobacteriota c UBA1414 o Hakubanellales
GCA 001796335.1 d Bacteria p Chloroflexota c Anaerolineae o Anaerolineales
GCA 001795335.1 d Bacteria p Chloroflexota c Anaerolineae o Anaerolineales
GCA 903910795.1 d Bacteria p Chloroflexota c Anaerolineae o Anaerolineales
GCA 004028505.1 d Bacteria p Desulfobacterota c Desulfobulbia o Desulfobulbales
GCA 011389815.1 d Bacteria p Desulfobacterota c Desulfobulbia o Desulfobulbales
GCA 011391865.1 d Bacteria p Desulfobacterota c Desulfobulbia o Desulfobulbales
GCA 002011735.1 d Bacteria p Nitrospirota c Thermodesulfovibrionia o UBA6902
GCA 011047485.1 d Bacteria p Actinobacteriota c UBA9087 o UBA10029
GCA 013152795.1 d Bacteria p Nitrospirota c Thermodesulfovibrionia o UBA6902
GCF 900176005.1 d Bacteria p Firmicutes B c Moorellia o Moorellales
GCF 013167955.1 d Bacteria p Firmicutes B c Moorellia o Moorellales
GCA 014360065.1 d Bacteria p Firmicutes B c Moorellia o Moorellales
GCF 006538365.1 d Bacteria p Firmicutes B c Moorellia o Moorellales
GCF 002995805.1 d Bacteria p Firmicutes B c Moorellia o Moorellales
GCF 001875325.1 d Bacteria p Firmicutes B c Moorellia o Moorellales
GCF 001295365.1 d Bacteria p Firmicutes c Bacilli o Bacillales

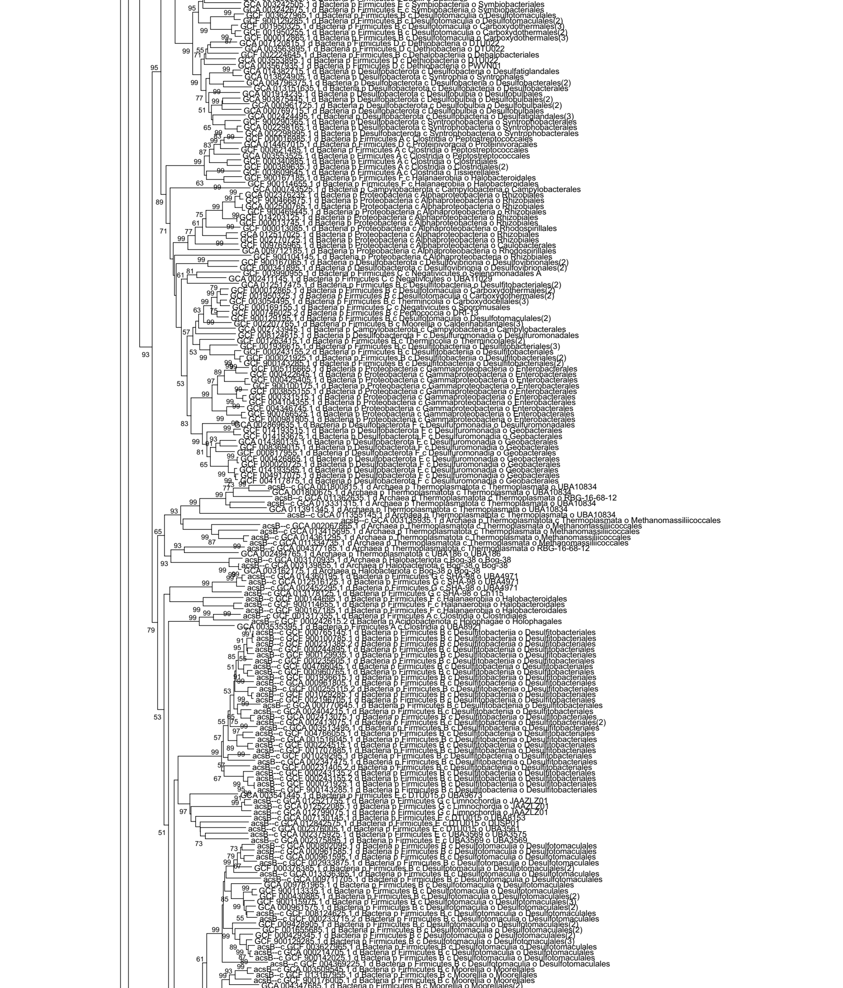

GCA 003242675.1 d Bacteria p Firmicutes E c Symbiobacteriia o Symbiobacteriales
GCF 003627965.1 d Bacteria p Firmicutes B c Desulfotomaculia o Desulfotomaculales
GCF 900129285.1 d Bacteria p Firmicutes B c Desulfotomaculia o Desulfotomaculales(2)
GCF 001950255.1 d Bacteria p Firmicutes B c Desulfotomaculia o Carboxydothermales(2)
GCF 000012865.1 d Bacteria p Firmicutes B c Desulfotomaculia o Carboxydothermales(3)
GCA 007120815.1 d Bacteria p Firmicutes D c Dethiobacteria o DTU022
GCA 003553895.1 d Bacteria p Firmicutes D c Dethiobacteria o DTU022
GCF 002224645.1 d Bacteria p Firmicutes B c Dehalobacteriia o Dehalobacteriales
GCA 003567935.1 d Bacteria p Firmicutes D c Dethiobacteria o PWVN01
GCA 014382715.1 d Bacteria p Desulfobacterota c Desulfobacteria o Desulfatiglandales
GCA 013824905.1 d Bacteria p Desulfobacterota c Syntrophia o Syntrophales
GCA 013151635.1 d Bacteria p Desulfobacterota c Desulfobacteria o Desulfobacterales
GCA 001914235.1 d Bacteria p Desulfobacterota c Desulfobulbia o Desulfobulbales
GCA 903875445.1 d Bacteria p Desulfobacterota c Desulfobulbia o Desulfobulbales(2)
GCA 000961725.1 d Bacteria p Desulfobacterota c Desulfobulbia o Desulfobulbales(2)
GCA 002424495.1 d Bacteria p Desulfobacterota c Desulfobacteria o Desulfatiglandales(3)
GCF 900290365.1 d Bacteria p Desulfobacterota c Syntrophobacteria o Syntrophobacterales
GCA 002298165.1 d Bacteria p Desulfobacterota c Syntrophobacteria o Syntrophobacterales
GCA 002298995.1 d Bacteria p Desulfobacterota c Syntrophobacteria o Syntrophobacterales
GCA 014467015.1 d Bacteria p Firmicutes D c Proteinivoracia o Proteinivoracales
GCF 000621485.1 d Bacteria p Firmicutes A c Clostridia o Peptostreptococcales
GCA 003553525.1 d Bacteria p Firmicutes A c Clostridia o Peptostreptococcales
GCF 000340885.1 d Bacteria p Firmicutes A c Clostridia o Clostridiales
GCF 003609645.1 d Bacteria p Firmicutes A c Clostridia o Tissierellales
GCF 900167185.1 d Bacteria p Firmicutes F c Halanaerobiia o Halobacteroidales
GCF 900114655.1 d Bacteria p Firmicutes F c Halanaerobiia o Halobacteroidales
GCA 000743525.1 d Bacteria p Campylobacterota c Campylobacteria o Campylobacterales
GCA 002500765.1 d Bacteria p Proteobacteria c Alphaproteobacteria o Rhizobiales
GCF 900469445.1 d Bacteria p Proteobacteria c Alphaproteobacteria o Rhizobiales
GCF 000013085.1 d Bacteria p Proteobacteria c Alphaproteobacteria o Rhodospirillales
GCA 012517025.1 d Bacteria p Proteobacteria c Alphaproteobacteria o Rhizobiales
GCF 002770725.1 d Bacteria p Proteobacteria c Alphaproteobacteria o Rhizobiales
GCA 009712185.1 d Bacteria p Proteobacteria c Alphaproteobacteria o Rhodospirillales
GCF 900104145.1 d Bacteria p Proteobacteria c Alphaproteobacteria o Rhizobiales
GCF 900167065.1 d Bacteria p Desulfobacterota c Desulfovibrionia o Desulfovibrionales(2)
GCF 000341895.1 d Bacteria p Desulfobacterota c Desulfovibrionia o Desulfovibrionales(2)
GCF 003990955.1 d Bacteria p Firmicutes C c Negativicutes o Selenomonadales A
GCA 002411145.1 d Bacteria p Firmicutes C c Negativicutes o UBA11029
GCA 012517475.1 d Bacteria p Firmicutes B c Desulfitobacteriia o Desulfitobacteriales(2)
GCF 000012865.1 d Bacteria p Firmicutes B c Desulfotomaculia o Carboxydothermales(2)
GCF 001950325.1 d Bacteria p Firmicutes B c Desulfotomaculia o Carboxydothermales(2)
GCF 003054495.1 d Bacteria p Firmicutes B c Thermincolia o Carboxydocellales(3)
GCF 900129195.1 d Bacteria p Firmicutes B c Desulfotomaculia o Desulfotomaculales(2)
GCF 002207765.1 d Bacteria p Firmicutes B c Moorellia o Calderihabitantales(3)
GCA 002733945.1 d Bacteria p Campylobacterota c Campylobacteria o Campylobacterales
GCF 001263415.1 d Bacteria p Firmicutes B c Thermincolia o Thermincolales(2)
GCF 001936615.1 d Bacteria p Firmicutes B c Desulfitobacteriia o Desulfitobacteriales(3)
GCF 000243155.2 d Bacteria p Firmicutes B c Desulfitobacteriia o Desulfitobacteriales
GCF 000021925.1 d Bacteria p Firmicutes B c Desulfitobacteriia o Desulfitobacteriales(2)
GCF 000422645.1 d Bacteria p Proteobacteria c Gammaproteobacteria o Enterobacterales
GCF 000425405.1 d Bacteria p Proteobacteria c Gammaproteobacteria o Enterobacterales
GCF 900100175.1 d Bacteria p Proteobacteria c Gammaproteobacteria o Enterobacterales
GCF 003855155.1 d Bacteria p Proteobacteria c Gammaproteobacteria o Enterobacterales
GCF 004104355.1 d Bacteria p Proteobacteria c Gammaproteobacteria o Enterobacterales
GCF 004346745.1 d Bacteria p Proteobacteria c Gammaproteobacteria o Enterobacterales
GCF 900766525.1 d Bacteria p Proteobacteria c Gammaproteobacteria o Enterobacterales
GCF 000981805.1 d Bacteria p Proteobacteria c Gammaproteobacteria o Enterobacterales
GCF 014193515.1 d Bacteria p Desulfobacterota F c Desulfuromonadia o Geobacterales
GCF 014193675.1 d Bacteria p Desulfobacterota F c Desulfuromonadia o Geobacterales
GCA 014380135.1 d Bacteria p Desulfobacterota F c Desulfuromonadia o Geobacterales
GCF 000426865.1 d Bacteria p Desulfobacterota F c Desulfuromonadia o Geobacterales
GCF 000020725.1 d Bacteria p Desulfobacterota F c Desulfuromonadia o Geobacterales
GCF 014193585.1 d Bacteria p Desulfobacterota F c Desulfuromonadia o Geobacterales
GCF 004117875.1 d Bacteria p Desulfobacterota F c Desulfuromonadia o Geobacterales
acsB--c GCA 001800815.1 d Archaea p Thermoplasmatota c Thermoplasmata o UBA10834
GCA 001800675.1 d Archaea p Thermoplasmatota c Thermoplasmata o UBA10834
acsB--c GCA 011626635.1 d Archaea p Thermoplasmatota c Thermoplasmata o RBG-16-68-12
GCA 011391345.1 d Archaea p Thermoplasmatota c Thermoplasmata o UBA10834
acsB--c GCA 011355145.1 d Archaea p Thermoplasmatota c Thermoplasmata o UBA10834
acsB--c GCA 003135935.1 d Archaea p Thermoplasmatota c Thermoplasmata o Methanomassiliicoccales
acsB--c GCA 002067865.1 d Archaea p Thermoplasmatota c Thermoplasmata o Methanomassiliicoccales
acsB--c GCA 011334735.1 d Archaea p Thermoplasmatota c Thermoplasmata o Methanomassiliicoccales
acsB--c GCA 004377185.1 d Archaea p Thermoplasmatota c Thermoplasmata o RBG-16-68-12
GCA 002494765.1 d Archaea p Thermoplasmatota c UBA186 o UBA186
acsB--c GCA 003170935.1 d Archaea p Halobacteriota c Bog-38 o Bog-38
GCA 003162175.1 d Archaea p Halobacteriota c Bog-38 o Bog-38
acsB--c GCA 014360195.1 d Bacteria p Firmicutes G c SHA-98 o UBA4971
acsB--c GCA 012516125.1 d Bacteria p Firmicutes G c SHA-98 o UBA4971
acsB--c GCA 002452295.1 d Bacteria p Firmicutes G c SHA-98 o UBA4971
acsB--c GCF 000144695.1 d Bacteria p Firmicutes F c Halanaerobiia o Halobacteroidales
acsB--c GCF 900114655.1 d Bacteria p Firmicutes F c Halanaerobiia o Halobacteroidales
acsB--c GCF 900167185.1 d Bacteria p Firmicutes F c Halanaerobiia o Halobacteroidales
acsB--c GCF 002442615.2 d Bacteria p Acidobacteriota c Holophagae o Holophagales
GCA 003535395.1 d Bacteria p Firmicutes A c Clostridia o UBA8821
acsB--c GCF 000765145.1 d Bacteria p Firmicutes B c Desulfitobacteriia o Desulfitobacteriales
acsB--c GCF 900100785.1 d Bacteria p Firmicutes B c Desulfitobacteriia o Desulfitobacteriales
acsB--c GCF 900129935.1 d Bacteria p Firmicutes B c Desulfitobacteriia o Desulfitobacteriales
acsB--c GCF 000235605.1 d Bacteria p Firmicutes B c Desulfitobacteriia o Desulfitobacteriales
acsB--c GCF 004766045.1 d Bacteria p Firmicutes B c Desulfitobacteriia o Desulfitobacteriales
acsB--c GCA 000961805.1 d Bacteria p Firmicutes B c Desulfitobacteriia o Desulfitobacteriales
acsB--c GCF 000255115.2 d Bacteria p Firmicutes B c Desulfitobacteriia o Desulfitobacteriales
acsB--c GCA 002404215.1 d Bacteria p Firmicutes B c Desulfitobacteriia o Desulfitobacteriales
acsB--c GCA 002413075.1 d Bacteria p Firmicutes B c Desulfitobacteriia o Desulfitobacteriales(2)
acsB--c GCA 001516045.1 d Bacteria p Firmicutes B c Desulfitobacteriia o Desulfitobacteriales
acsB--c GCF 000224515.1 d Bacteria p Firmicutes B c Desulfitobacteriia o Desulfitobacteriales
acsB--c GCF 001707885.1 d Bacteria p Firmicutes B c Desulfitobacteriia o Desulfitobacteriales
acsB--c GCF 000231405.2 d Bacteria p Firmicutes B c Desulfitobacteriia o Desulfitobacteriales
acsB--c GCF 000243135.2 d Bacteria p Firmicutes B c Desulfitobacteriia o Desulfitobacteriales
acsB--c GCF 000243155.2 d Bacteria p Firmicutes B c Desulfitobacteriia o Desulfitobacteriales
acsB--c GCF 900143285.1 d Bacteria p Firmicutes B c Desulfitobacteriia o Desulfitobacteriales
GCA 003541445.1 d Bacteria p Firmicutes E c DTU015 o UBA9673
acsB--c GCA 012521755.1 d Bacteria p Firmicutes G c Limnochordia o JAAZLZ01
acsB--c GCA 012522085.1 d Bacteria p Firmicutes G c Limnochordia o JAAZLZ01
acsB--c GCA 012799075.1 d Bacteria p Firmicutes G c Limnochordia o JAAZLZ01
acsB--c GCA 012842575.1 d Bacteria p Firmicutes E c DTU015 o DUSP01
acsB--c GCA 002376005.1 d Bacteria p Firmicutes E c DTU015 o UBA3561
acsB--c GCA 002375925.1 d Bacteria p Firmicutes E c UBA3569 o UBA3575
acsB--c GCA 002375895.1 d Bacteria p Firmicutes E c UBA3569 o UBA3569
acsB--c GCA 000961585.1 d Bacteria p Firmicutes B c Desulfotomaculia o Desulfotomaculales
acsB--c GCA 000961595.1 d Bacteria p Firmicutes B c Desulfotomaculia o Desulfotomaculales
acsB--c GCF 002933875.1 d Bacteria p Firmicutes B c Desulfotomaculia o Desulfotomaculales
GCF 000376385.1 d Bacteria p Firmicutes B c Desulfotomaculia o Desulfotomaculales(2)
acsB--c GCA 009711705.1 d Bacteria p Firmicutes B c Desulfotomaculia o Desulfotomaculales
GCA 009781965.1 d Bacteria p Firmicutes B c Desulfotomaculia o Desulfotomaculales
GCF 900113335.1 d Bacteria p Firmicutes B c Desulfotomaculia o Desulfotomaculales
GCF 000430885.1 d Bacteria p Firmicutes B c Desulfotomaculia o Desulfotomaculales(2)
GCA 000961575.1 d Bacteria p Firmicutes B c Desulfotomaculia o Desulfotomaculales(2)
acsB--c GCF 008124625.1 d Bacteria p Firmicutes B c Desulfotomaculia o Desulfotomaculales
acsB--c GCF 000233715.2 d Bacteria p Firmicutes B c Desulfotomaculia o Desulfotomaculales
GCF 000429345.1 d Bacteria p Firmicutes B c Desulfotomaculia o Desulfotomaculales(2)
GCF 900129285.1 d Bacteria p Firmicutes B c Desulfotomaculia o Desulfotomaculales(3)
acsB--c GCF 003627965.1 d Bacteria p Firmicutes B c Desulfotomaculia o Desulfotomaculales
acsB--c GCF 004369225.1 d Bacteria p Firmicutes B c Desulfotomaculia o Desulfotomaculales
acsB--c GCA 003509545.1 d Bacteria p Firmicutes B c Moorellia o Moorellales
acsB--c GCF 013167955.1 d Bacteria p Firmicutes B c Moorellia o Moorellales
acsB--c GCF 900176005.1 d Bacteria p Firmicutes B c Moorellia o Moorellales
GCA 004347685.1 d Bacteria p Firmicutes B c Moorellia o Moorellales(2)

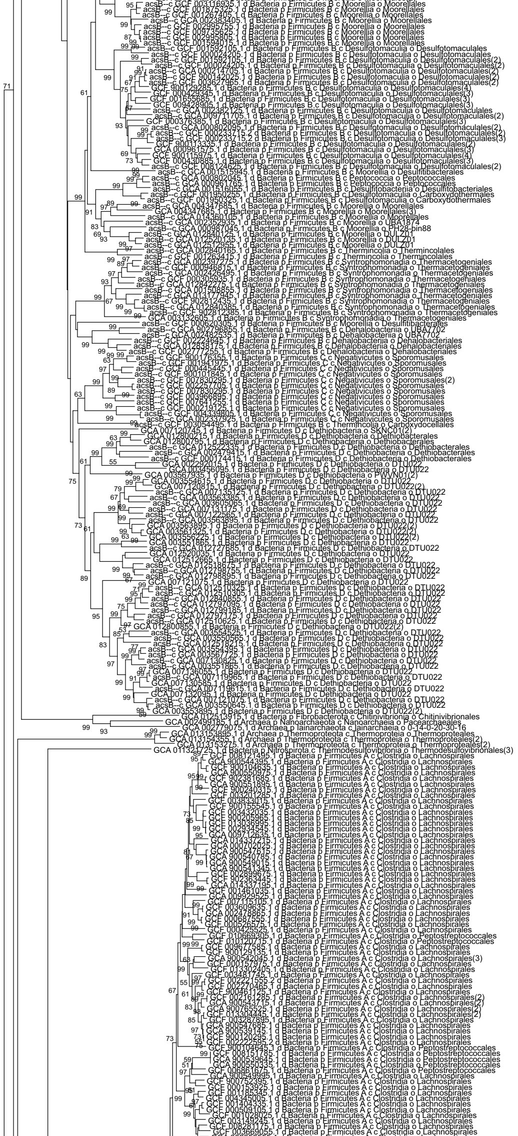

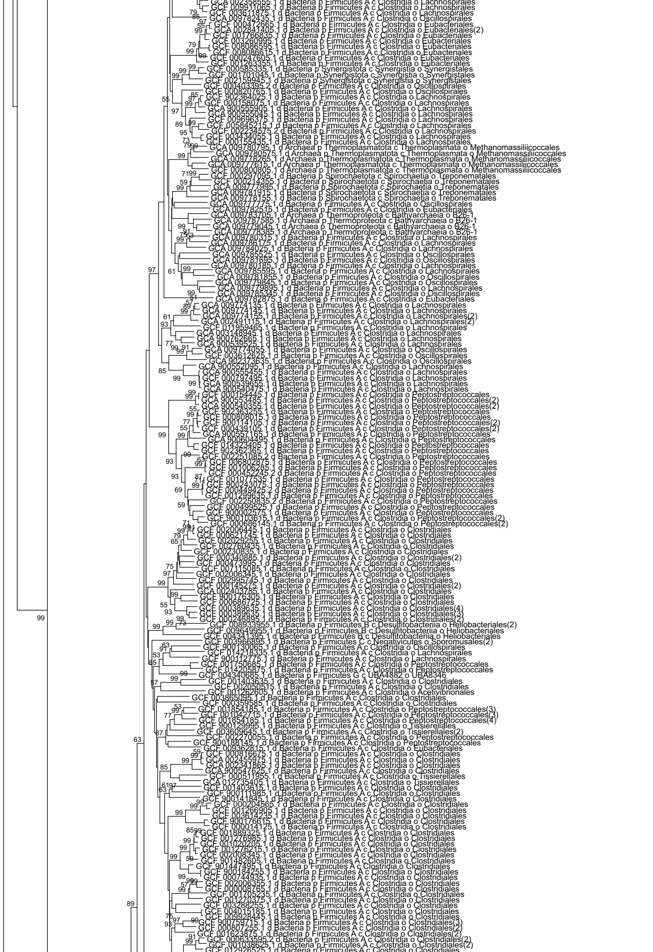

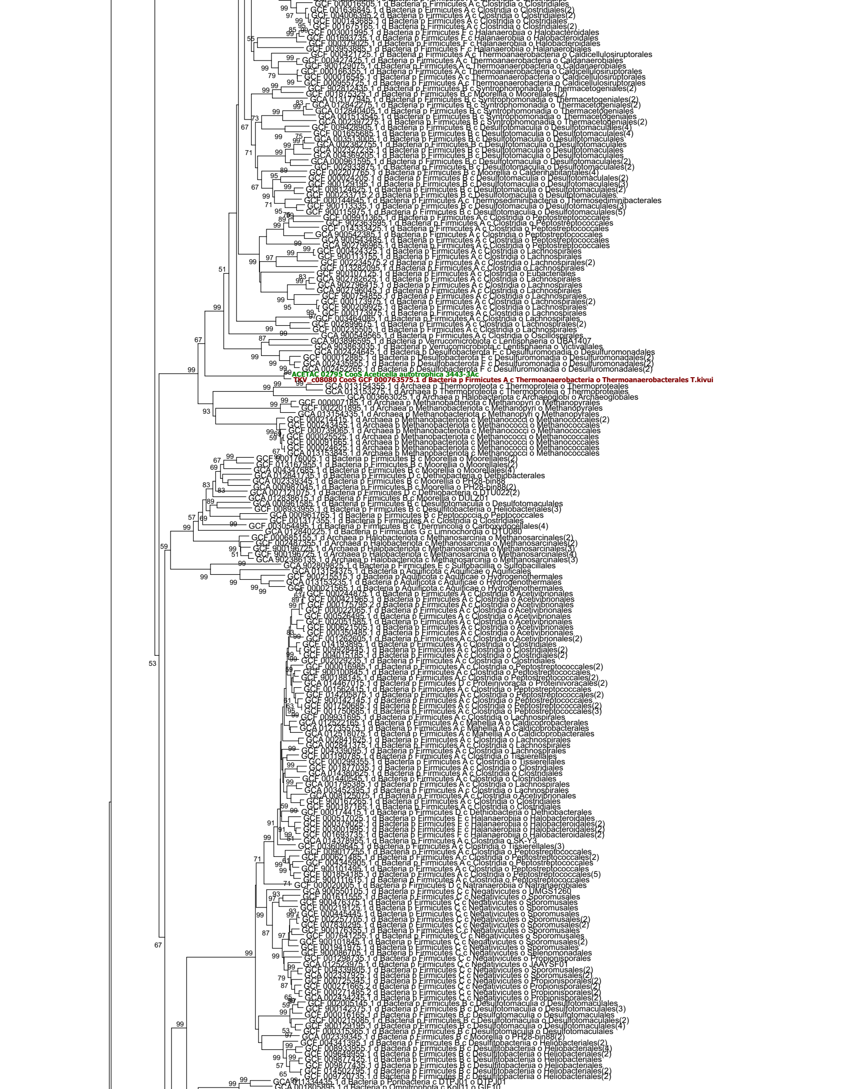
ACETAC_02795 CooS Aceticella autotrophica 3443-3Ac
TKV_c08080 CooS GCF 000763575.1 d Bacteria p Firmicutes A c Thermoanaerobacteria o Thermoanaerobacterales T.kivui

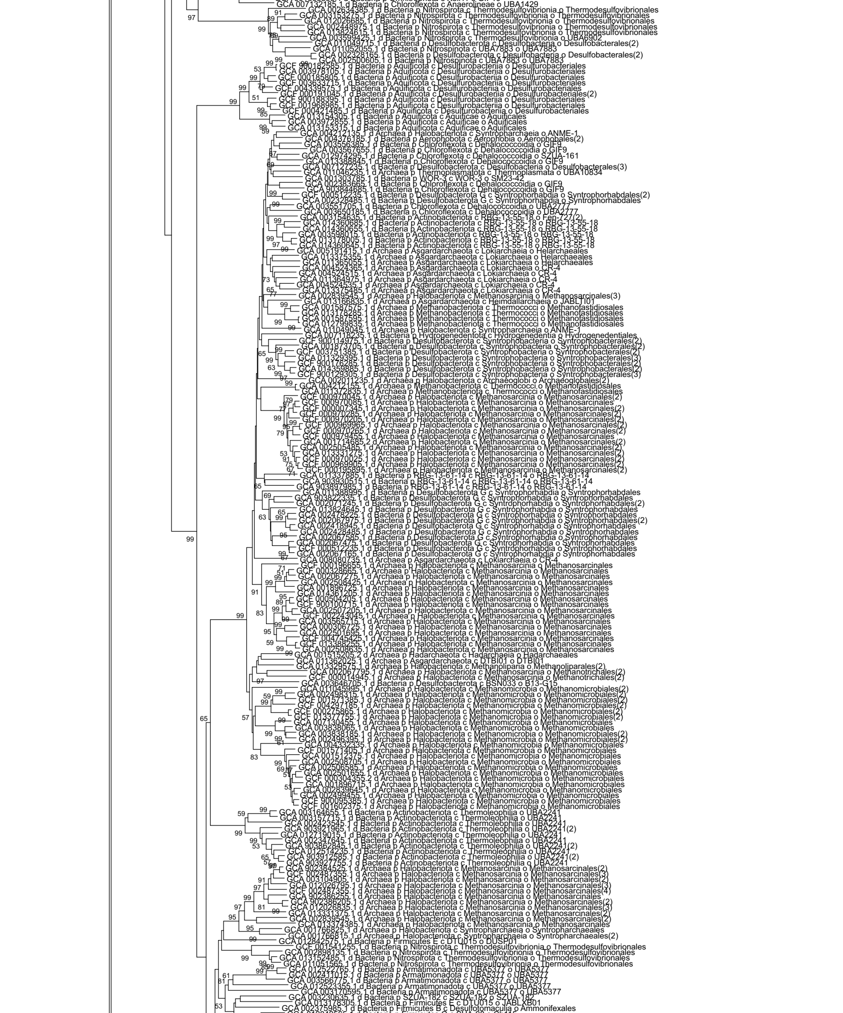

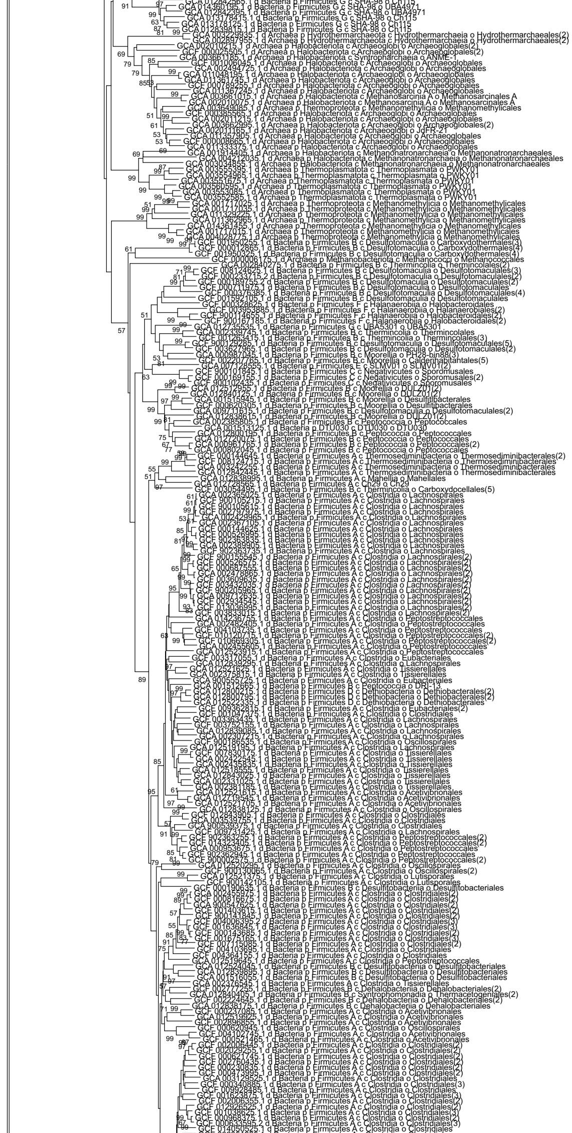

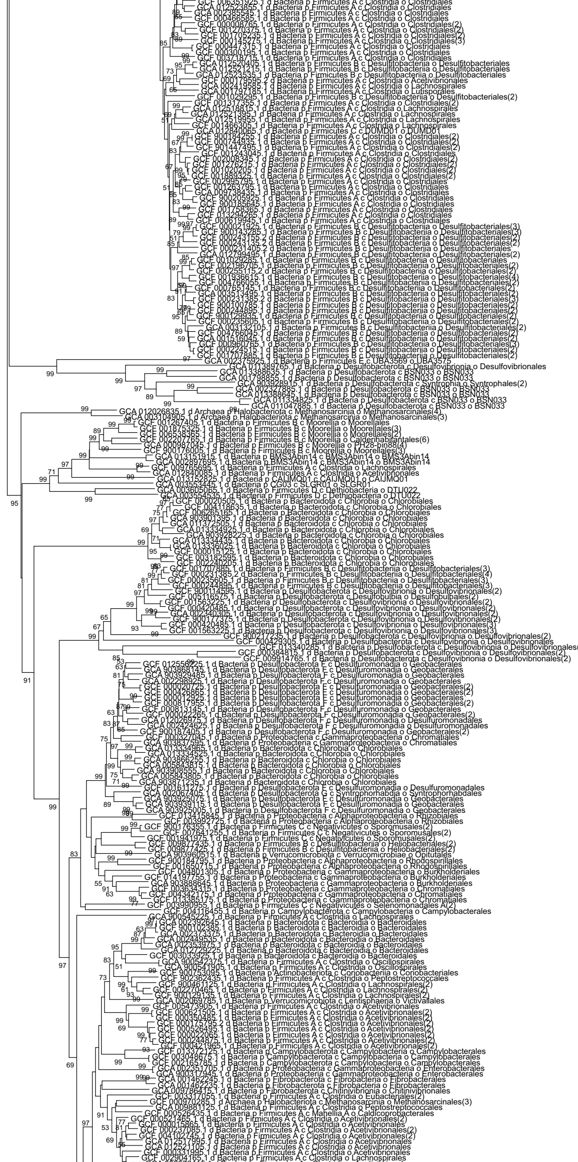

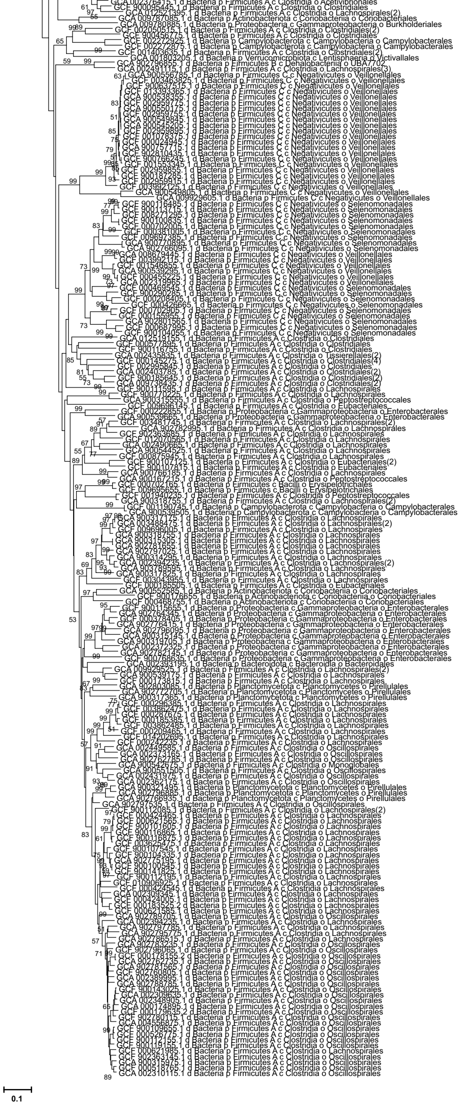
0.1
